# Metal and Activating Group Free C-4 Alkylation of Isoquinolines via a Temporary Dearomatization Strategy

**DOI:** 10.1021/acs.orglett.2c04149

**Published:** 2023-01-23

**Authors:** Aaron J. Day, Timothy C. Jenkins, Marvin Kischkewitz, Kirsten E. Christensen, Darren L. Poole, Timothy J. Donohoe

**Affiliations:** † Department of Chemistry, University of Oxford, Chemistry Research Laboratory, Mansfield Road, Oxford, OX1 3TA, United Kingdom; ‡ Discovery High-Throughput Chemistry, Medicinal Chemistry, GlaxoSmithKline Medicines Research Centre, Gunnels Wood Road, Stevenage, Hertfordshire SG1 2NY, United Kingdom

## Abstract

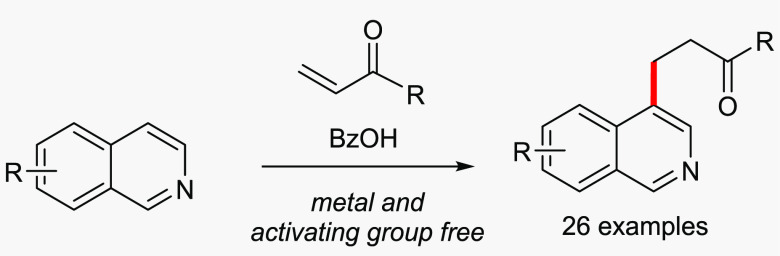

A simple method for the C-4 alkylation of isoquinolines is described using benzoic acid as a nucleophilic reagent and vinyl ketones as an electrophile. The reaction shows tolerance for substitution at C-3, and C-5–C-8 positions as well as allowing some variation of the vinyl ketone electrophiles. The products contain a carbonyl that can act as a synthetic handle for further manipulations giving esters, amines, or simple alkyl products.

The functionalization of aromatic heterocycles is an area of continuous interest for synthetic chemists. Given the ubiquitous presence of nitrogen-containing heterocycles in natural products, pharmaceuticals, and agrochemicals,^1^ the development of efficient methods for the preparation and manipulation of these moieties are important goals in the field of synthetic organic chemistry. Currently, access to C-4 alkylated isoquinolines is limited. The main preparatory methods are via the de novo construction of the isoquinoline core already bearing a C-4 substituent,^2^ or cross-coupling reactions of C-4 halo- or boronate prefunctionalized isoquinolines.^3^ However, limited examples of the direct C-4 functionalization of isoquinolines do exist and include the procedure of Minter and Re, where one equivalent of NaEt_3_BH was premixed with isoquinoline and the resultant 1,2-dihydroisoquinoline intermediate was then reacted with several aryl aldehydes. After elimination of water, the aromatic product was obtained (Figure 1a).^4^ A similar strategy was employed by Mamane and co-workers where alkyllithium nucleophiles were used and the 1,2-dihydroisoquinoline intermediate was trapped with alkyl halide electrophiles to enable the 1,4-dialkylation of isoquinolines.^5^ Very recently, Studer and co-workers reported an elegant strategy for the *meta*-CH functionalization of nitrogen-containing electron-deficient arenes that utilize a DMAD-derived oxazino group to accomplish temporary dearomatization.^6^

**Figure 1:**
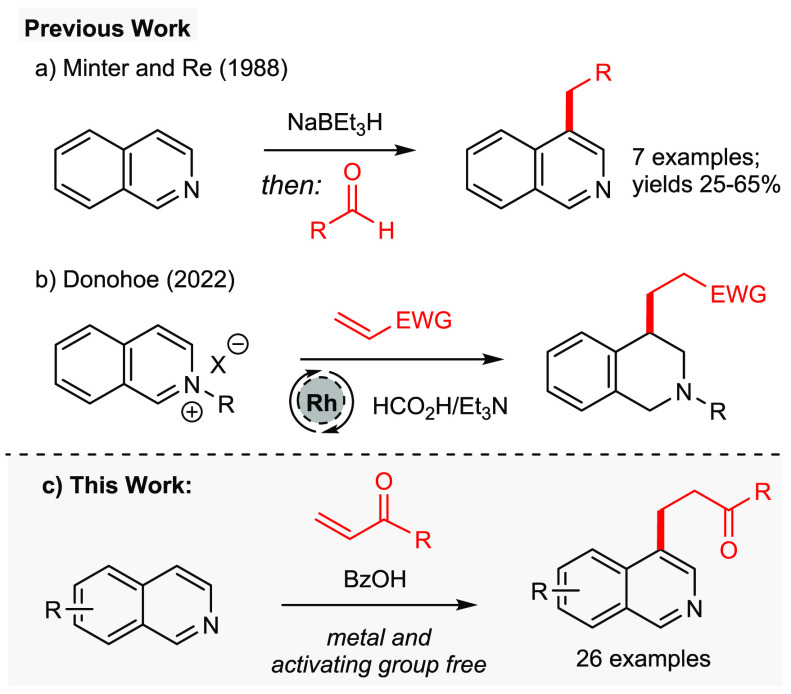
C-4 alkylation of isoquinolines.

We have disclosed procedures for the Ir- or Rh-catalyzed hydroxymethylenation of pyridinium, quinolinium, and isoquinolinium salts,^7^ and recently this methodology has been expanded to encompass a range of carbon electrophiles using acidic conditions (Figure 1b): some of these reductive reactions also operate without the use of a metal catalyst.^8^ In this work, we present a complementary acid-catalyzed reaction in which isoquinoline is alkylated at C-4 by a vinyl ketone but which retains the aromaticity of the isoquinoline ring (Figure 1c). Notably, in this process, the isoquinoline nitrogen does not require preactivation by quaternization in order to enable the desired reactivity.

During our earlier investigations on the rhodium-catalyzed reductive alkylation of isoquinolines, the importance of an *N*-activating group was probed. Surprisingly, in the absence of an activating group, isoquinoline (**1a**) still gave some reaction with methyl vinyl ketone (MVK, **2a**), albeit with no overall reduction occurring (Table 1, entry 1). Removing the [Rh] catalyst improved the yield, presumably by minimizing reduction of the starting materials (Table 1, entry 2). A potential hydride source was then shown to be unnecessary when we replaced 5:2 HCO_2_H/Et_3_N with AcOH (Table 1, entry 3). Adding too much acid proved detrimental to the yield (Table 1, entry 4); however, increasing the temperature to 65 °C was favorable (Table 1, entry 5). At this point, a wide range of acids and nucleophiles were screened, with benzoic acid proving to be the best (Table 1, entry 6; for a full list of nucleophiles screened and additional optimization tables, see the Supporting Information). With these conditions, the C-4 alkylation reaction could be performed on a gram-scale (Table 1, entry 7). Note that (i) using a large excess of the electrophile completely stopped the desired reaction (Table 1, entry 8) and (ii) the yield decreased when smaller amounts of benzoic acid were used (Table 1, entry 9).

**Table 1:**
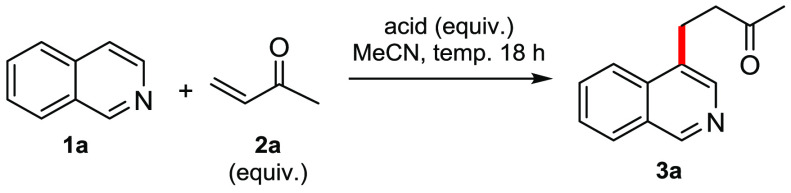
Optimization of the C-4 Alkylation of Isoquinoline (**1a**) with Methyl Vinyl Ketone (**2a**)^a^

entry	temperature (°C)	acid (equiv)	**2a** (equiv)	yield of **3a**^b^ (%)
1^c^	45	5:2 HCO_2_H/Et_3_N (4.0)	2	20
2	45	5:2 HCO_2_H/Et_3_N (4.0)	2	32
3	45	AcOH (3.0)	2	43
4	45	AcOH (6.0)	2	17
5	65	AcOH (3.0)	2	53
6	80	BzOH (3.0)	4	73
7^d^	80	BzOH (3.0)	4	61^e^
8	80	BzOH (3.0)	10	0
9	80	BzOH (1.0)	4	46

a Reactions were performed on 0.125 mmol scale.

b qNMR yields are reported.

c Contains 0.03 mol % [RhCp*Cl_2_]_2_ catalyst.

d Reaction was performed on 8.00 mmol scale.

e Isolated yield.

We presume the mechanism is related to that of the C-4 bromination of isoquinoline, first reported by Edinger and Bossung.^9^ In our proposal (Scheme 1), we suggest that **1a** can combine with benzoic acid at C-1 to give 1,2-dihydroisoquinoline intermediate **A**, which can subsequently react as a nucleophile with MVK to give **B**, which can then eliminate BzOH to furnish the aromatic 4-substituted isoquinoline **3a**. Consistent with this mechanism, reactions of C-1-substituted isoquinolines (Me, Ph, or Cl) afforded none of the desired 4-alkylated products—presumably because of a steric impediment to addition at this position. Additionally, under our optimized conditions, no reaction was observed on preformed *N*-alkylated isoquinolinium salts.^10^ To probe the mechanism, the reaction was performed in acetonitrile-*d*_3_, but no intermediate **A** was observed by NMR spectroscopy. Interestingly, we were able to observe the reaction of **2a** acting as a conjugate acceptor with the carboxylic acid and also with the isoquinolines **1a** and **3a** (reacting via nitrogen; see the Supporting Information). Resubjection of product **3a** to the reaction conditions but in the presence of a different electrophile (ethyl vinyl ketone) only gave recovered starting material and no crossover product **5a**.

**Scheme 1:**
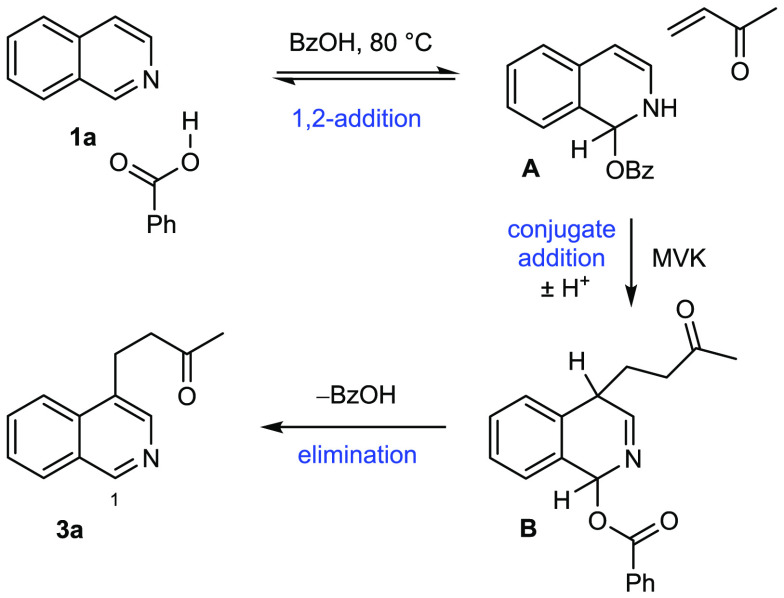
Proposed Mechanism for the Formation of **3a**

Using our optimized conditions consisting of MVK (4.0 equiv) and BzOH (3.0 equiv) in MeCN at 80 °C, the isoquinoline scope was explored.^11^ As previously stated, 1-substituted isoquinolines afforded none of the desired C-4 alkylated products. As expected, blocking the C-4 position with a Me or Cl/Br also prevented any of the desired alkylation. Of the C-3 substituted isoquinolines that were examined, only 3-methylisoquinoline underwent alkylation to give **3b** (Scheme 2). Pleasingly, the isoquinoline C-5–C-8 positions tolerated methyl, phenyl, and bromo substituents. Note that C-5 substitutions **3c**–**3g** generally led to poorer yields, perhaps due to an unfavorable *peri* interaction. Of the C-6 (**3h**–**3k**) and C-7 (**3l**–**3n**) substituents that were examined, there was no clear trend regarding reactivity. The C-8 substituents **3o**–**3r** were the most successful; all C-8-substituted isoquinolines gave better yields than that of the unsubstituted isoquinoline **1a**. The structure of the 8-Ph-substituted product **3p** was confirmed by its X-ray structure.^12^

**Scheme 2:**
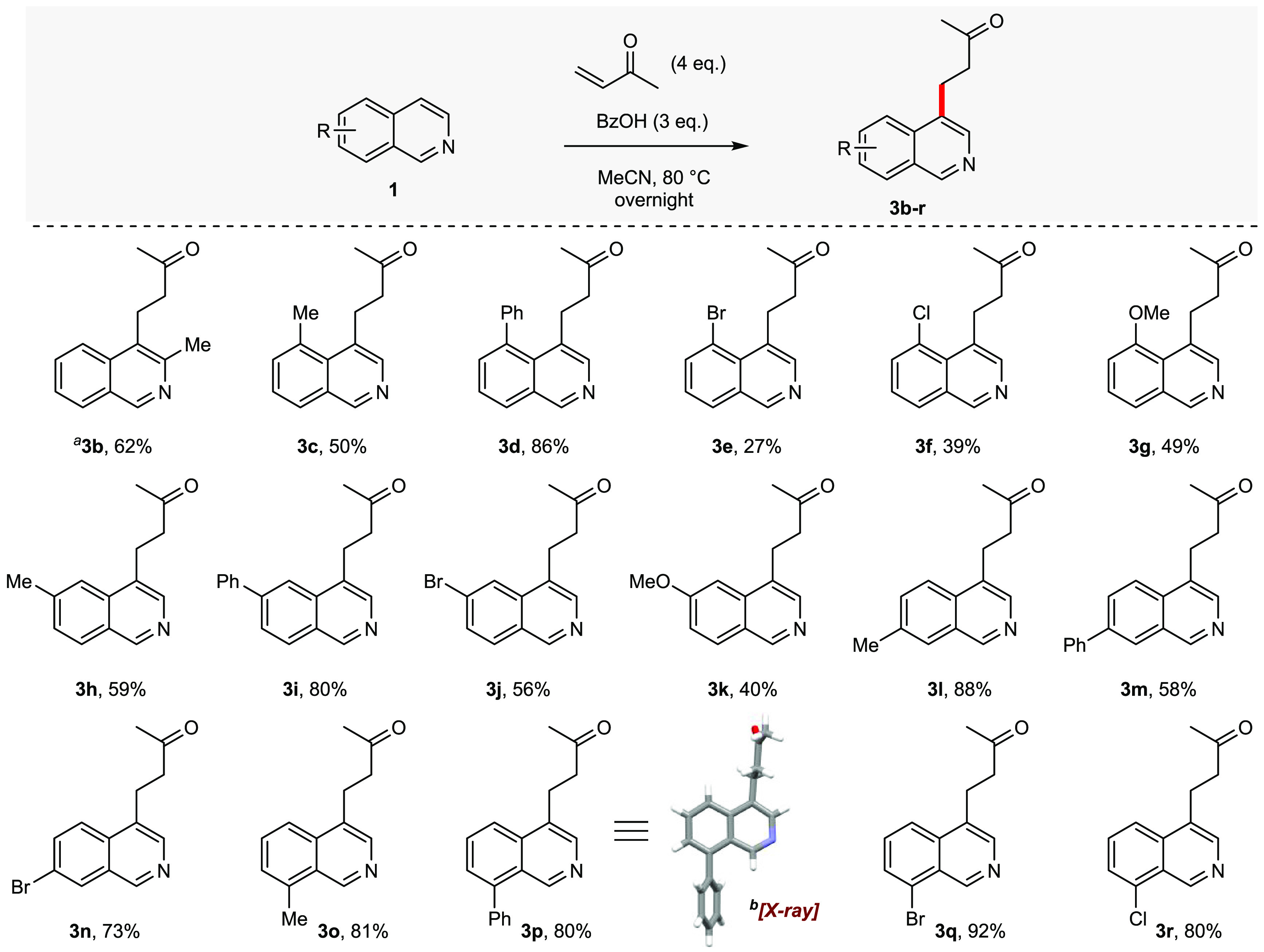
Scope of Isoquinolines for C-4 Alkylation with MVK Reactions were performed on a 0.250 mmol scale, unless otherwise stated. Isolated yields are reported. Overnight is ∼16 h. Reaction was performed on a 2.00 mmol scale. Determined by single crystal X-ray diffraction.^12^

Our attention then moved to examine the scope of electrophiles that could be used in the reaction (Scheme 3). We found that only vinyl ketones participated easily in this metal and activating group-free procedure (cf ref (8); see the Supporting Information for a full list of electrophiles screened). Moving from methyl vinyl ketone to ethyl, *iso*-propyl, or benzyl vinyl ketone, the reaction performed similarly giving **5a**–**5c**, **6**, and **7** in reasonable yields. Reactions with the commercially available ethyl vinyl ketone (**5a**–**5c**) were performed on gram-scale. However, reactions with *tert*-butyl, cyclopropyl, or cyclohexyl vinyl ketone gave no product; additionally, substitution on the vinyl group of the electrophile gave poor yields (<25%). For the more precious, noncommercial benzyl vinyl ketone, reducing the equivalents of the electrophile to 2.0 still afforded **7** in 56% yield. Aryl vinyl ketones proved more challenging and higher temperatures were required for good conversion (100 °C). Interestingly, the para-CF_3_, and furoyl vinyl ketone gave the best yields for isoquinolines **9** and **10**.

**Scheme 3:**
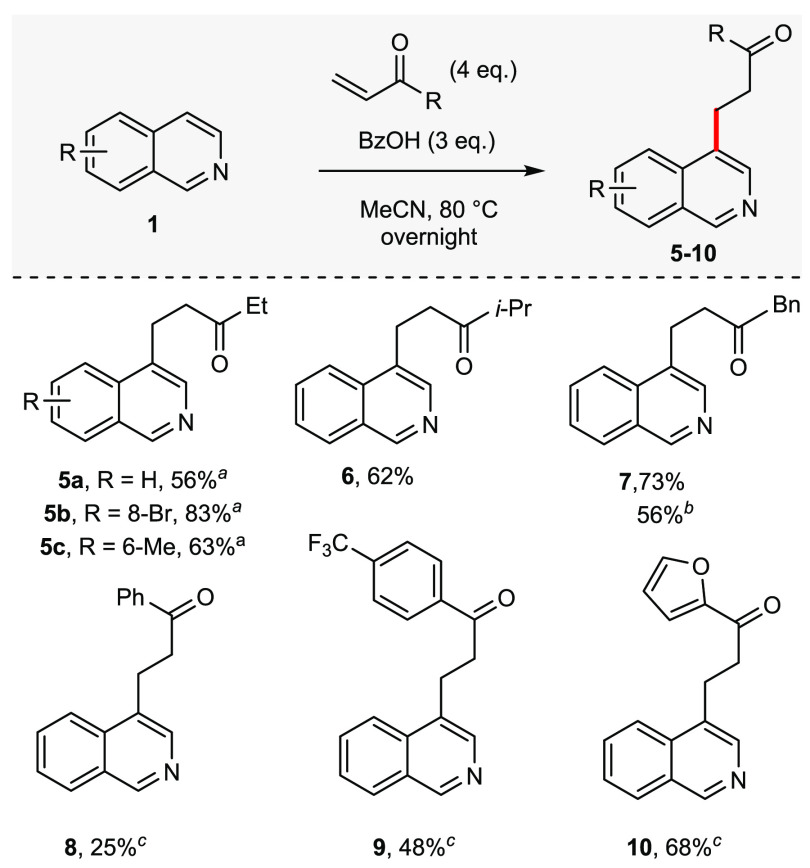
Scope of Vinyl Ketone Electrophiles Reactions were performed on a 0.250 mmol scales, unless otherwise stated. Isolated yields are reported. Overnight is ∼16 h. Reaction was performed on a 10 mmol scale. 2 equiv of electrophile was used. Reaction was at 100 °C.

With ready access to C-4 alkylated isoquinolines, we were interested in demonstrating several synthetically useful reactions which are possible in order to maximize product diversity (Scheme 4). Simple Grignard addition to **3a** with methylmagnesium bromide afforded **11**, while Corey–Chaykovsky epoxidation gave **13** in 70% yield. Wolf–Kishner reduction of **3a** afforded the simple alkylated product **14** in 61% yield. Baeyer–Villiger oxidation of the benzylated adduct **7a** afforded the ester product with complete regioselectivity for the migrating benzyl group, but with overoxidation to the *N*-oxide. However, subsequent reduction with B_2_pin_2_, according to Lakshman’s protocol,^13^ afforded the isoquinoline ester **12**. Lastly, a reductive amination of **3a** with benzylamine gave **15** in 66% yield.

**Scheme 4:**
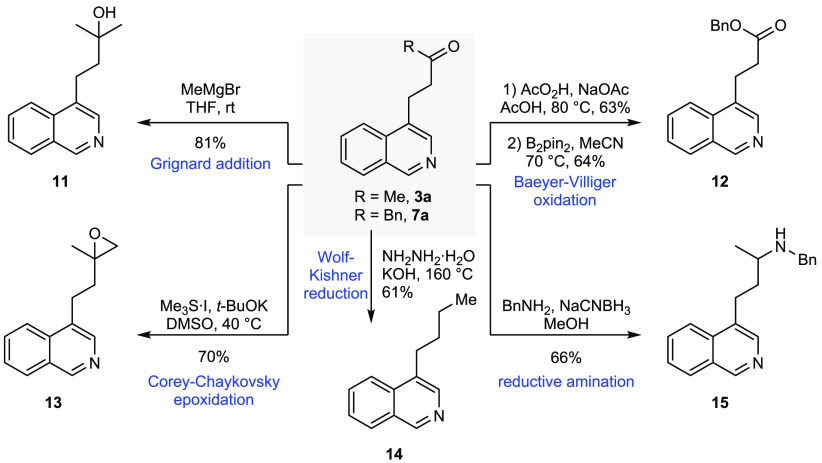
Derivatization of Alkylated Isoquinolines

In summary, we have described a new method for the C-4 alkylation of isoquinolines. This method retains aromaticity in the products and is complementary to our previously described methods, which accomplish reductive functionalization at C-4. Moreover, the procedure is straightforward to carry out and does not require an *N*-activating group on the arene substrate.

## Data Availability

The data underlying this study are available in the published article and its Supporting Information.

## Supporting Information Available

Experimental procedures and characterization for all new compounds; expanded optimization tables (PDF)
