# Supplementary material for: Metal and Activating Group Free C-4 Alkylation of Isoquinolines via a Temporary Dearomatization Strategy
Source: Org Lett. 2023 Jan 23;25(4):614–8. doi: 10.1021/acs.orglett.2c04149 (PMC9903316; doi:10.1021/acs.orglett.2c04149)
Supplement: Supplementary file 1 — ol2c04149_si_001.pdf [file ol2c04149_si_001.pdf]

Supporting Information for

Metal and Activating Group Free C-4 Alkylation of Isoquinolines via a Temporary Dearomatization Strategy

*Aaron J. Day,[a] Timothy C. Jenkins,[a] Marvin Kischkewitz,[a] Kirsten E. Christensen,[a] Darren L. Poole[b] and Timothy J. Donohoe [a]**

[a] Department of Chemistry, University of Oxford, Chemistry Research Laboratory, Mansfield Road, Oxford, OX1 3TA, UK. E-mail: timothy.donohoe@chem.ox.ac.uk

[b] Discovery High-Throughput Chemistry, Medicinal Chemistry, GlaxoSmithKline Medicines Research Centre, Gunnels Wood Road, Stevenage, Hertfordshire, SG1 2NY, UK.

Contents

**1.**	**General Information**	**S2**
**2.**	**Experimental Procedures**	**S4**
**2.1**	**General Procedures**	**S4**
**2.2**	**Synthesis of Isoquinolines**	**S7**
**2.3**	**Synthesis of Vinyl Ketones**	**S11**
**2.4**	**C-4 Alkylation of Isoquinolines**	**S14**
**2.5**	**Derivatisation Reactions**	**S30**
**3.**	**Screening and Optimisation**	**S35**
**4.**	**NMR Experiment**	**S40**
**5.**	**NMR Spectra**	**S42**
**6.**	**References**	**S148**

1. General Information

Reactions were carried out in standard glassware under an atmosphere of air unless stated otherwise. Room temperature (rt) refers to 20-25 °C. Temperatures of 0 °C were obtained using an ice/water bath. High temperatures were obtained using an oil bath equipped with a contact thermometer.

Diethyl ether, CH_2Cl_2, DMF, and tetrahydrofuran were purified by filtration through activated alumina columns employing the method of Grubbs and co-workers.[1] All other solvents and reagents were used as supplied without prior purification. All other reagents were used directly as supplied by major chemical suppliers, or following purification procedures described by Perrin and Armarego.[2]

Thin layer chromatography was performed on Merck Kieselgel 60 F_{254} 0.25 mm pre-coated aluminium plates. Product spots were visualized under UV light (λ = 254 nm) and/or by staining with potassium permanganate solution. Flash column chromatography was performed using VWR silica gel 60 (40-63 μm particle size) using head pressure by means of a nitrogen line.

NMR spectroscopy was carried out using Bruker 400, 500, or 600 MHz spectrometers in the deuterated solvent stated, using the residual non-deuterated solvent signal as an internal reference. Chemical shifts are quoted in ppm with signal splittings recorded as singlet (s), doublet (d), triplet (t), quartet (q), quintet (qn), sextet (sext), septet (sept), octet (oct), nonet (non) and multiplet (m). Higher multiplicities are indicated by combinations of these abbreviations, for example: doublet of doublets (dd). The abbreviation br denotes broad. Coupling constants, *J*, are measured to the nearest 0.1 Hz and are presented as observed. The numbering of compounds for assignment of NMR spectra is arbitrary and not based on IUPAC numbering. Naming of chemical structures was done using Chemdraw software.

Infrared spectra were recorded neat on a Bruker Tensor 27 spectrometer equipped with an attenuated total reflectance attachment with internal calibration. Absorption maxima (λ_{max}) are quoted in wavenumbers (cm^{-1}). The abbreviation br denotes broad.

Electrospray ionisation (ESI) HRMS were recorded on a Thermo Exactive orbitrap spectrometer equipped with a Waters Equity LC system, with a flow rate of 0.2 mL/min using water:methanol:formic acid (10:89.9:0.1) as eluent. The system uses a heated electrospray ionisation (HESI-II) probe for ESI^+ and has a resolution of 50,000 FWHM under conditions for maximum sensitivity, with an accuracy of better than 5 ppm for 24 h following external calibration on the day of analysis. The mass reported is that containing the most abundant isotopes, with each value rounded to 4 decimal places and within 5 ppm of the calculated mass. Electron impact ionisation (EI) HRMS were performed on an Agilent 7200 quadrupole time of flight (Q-ToF) instrument equipped with a direct insertion probe supplied by Scientific Instrument Manufacturer (SIM) GmbH. Instrument control and data processing were performed using Agilent MassHunter software. The mass reported is that containing the most abundant isotopes, with each value to 4 decimal places and within 5 ppm of the calculated mass.

2. *Experimental Procedures*

2.1 General Procedures

General Procedure A: For the preparation of methyl and phenyl substituted isoquinolines via Suzuki cross coupling reactions.

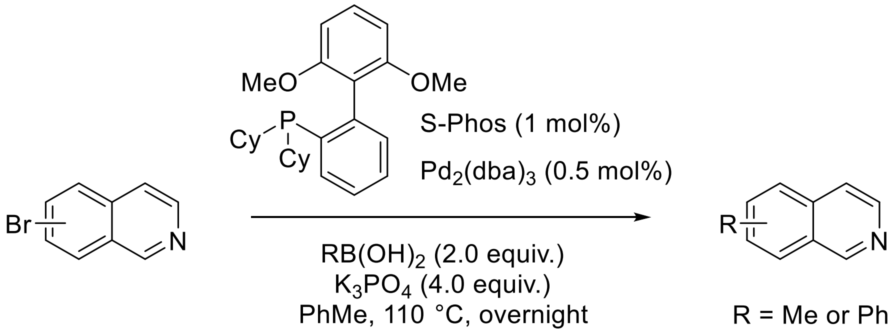

In a 2-necked round bottom flask, bromoisoquinoline (1.00 equiv.), boronic acid (2.00 equiv.), K_3PO_4 (4.00 equiv.) and S-Phos (1 mol%) were combined and taken up in PhMe (0.1 M). Argon was bubbled through for 10 min, then $Pd_2(dba)_3$ (0.5 mol%) was added and the mixture was heated to 110 °C for 8 – 18 h. The reaction was cooled to room temperature and water was added. The layers were separated and the aqueous phase was extracted with EtOAc once. The combined organic phases were dried over $MgSO_4$, filtered, and concentrated *in vacuo*. The residue was purified by flash column chromatography on SiO_2 (pentane/EtOAc) to afford the substituted isoquinoline.

General Procedure B: For the preparation of vinyl ketones via addition of vinylmagnesium bromide to a Weinreb amide.

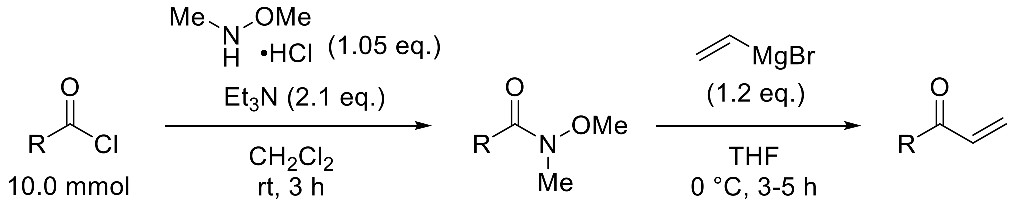

This method has been adapted from the procedure of Marsden and Nelson.[3]

To a solution of acid chloride (10.0 mmol, 1.0 equiv.) in dry CH_2Cl_2 (50 mL) was added *N,O*-dimethylhydroxylamine hydrochloride (1.02 g, 10.5 mmol, 1.05 equiv.) Et_3N (2.9 mL, 21 mmol, 2.1 equiv.) was added dropwise over 5-10 min and the mixture was stirred at room temperature for a further 3 h. The reaction was quenched with 1 M HCl (20 mL) and the layers were separated. The aqueous phase was extracted with CH_2Cl_2 (20 mL) and the combined organic extracts were dried over Na_2SO_4, filtered and concentrated *in vacuo*. This crude Weinreb amide was either used directly without purification, or after chromatography on SiO_2 (pentane/Et_2O). Typically, the overall yield was improved if the intermediate amide was purified prior to Grignard addition.

A solution of Weinreb amide (1.0 equiv.) in dry THF (50 mL) was cooled to 0 °C and vinylmagnesium bromide (1.0 M in THF, 1.2 equiv.) was added dropwise over 10 min. The reaction mixture was then warmed to rt and stirred until starting material was consumed by TLC (typically 3-5 h). The reaction was cooled again to 0 °C, and quenched by slow addition of 1 M HCl (20 mL), and extracted with Et_2O (15 mL × 5). The combined organic extracts were washed with brine (20 mL), dried over $MgSO_4$, filtered, and concentrated *in vacuo*. The crude residue was purified by flash column chromatography of SiO_2 (pentane/Et_2O) to afford the vinyl ketone.

N.B. These compounds are close analogues of methyl vinyl ketone, which is highly toxic. Care should be taken when handling. All vinyl ketones were stored in the freezer (–20 °C) and used shortly after their preparation.

General Procedure C: Metal-Free C-4 alkylation of isoquinolines

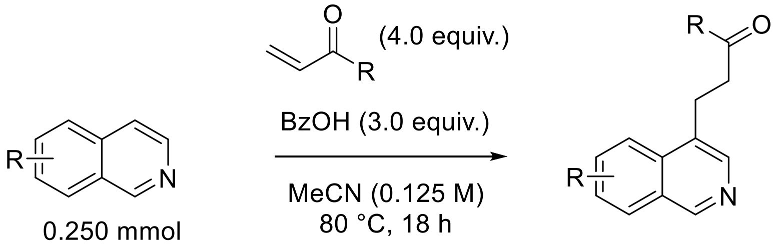

In a 1.5 mL GC vial equipped with a magnetic stir bar, the substrate isoquinoline (0.250 mmol, 1.0 equiv.), BzOH (91.6 mg, 0.750 mmol, 3.0 equiv.), vinyl ketone (1.00 mmol, 4.0 equiv.), and MeCN (0.2 mL, 0.125 mmol) were combined. The vial was placed in a pre-heated oil bath and heated at 80 °C for 18 h. The reaction mixture was taken up in CH_2Cl_2 (15 mL) and quenched with 0.1 M K_2CO_3 solution (20 mL). The layers were separated, and the aqueous phase was further extracted with CH_2Cl_2 (15 mL × 2). The combined organic extracts were dried over $MgSO_4$, filtered, and concentrated *in vacuo*. The residue was purified by flash column chromatography on SiO_2 (Et_2O/acetone, CH_2Cl_2/MeOH, or pentane/EtOAc/acetone) to afford the product.

A common by-product in these reactions is the adduct between benzoic acid and the vinyl ketone. This were separated during chromatography and discarded.

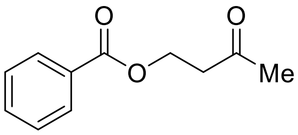

Data for the MVK-BzOH adduct:

^{1}H NMR (400 MHz, $CDCl_3$) δ 8.04 – 7.96 (2H, m), 7.59 – 7.51 (1H, m), 7.47 – 7.38 (2H, m), 4.58 (2H, t, *J* = 6.3 Hz), 2.90 (2H, t, *J* = 6.3 Hz), 2.23 (3H, s)

^{13}C NMR (101 MHz, $CDCl_3$) δ 205.7, 166.5, 133.2, 130.1, 129.7, 128.5, 60.0, 42.5, 30.4

Spectroscopic data were consistent with the literature data for this compound.[4]

2.2 Synthesis of Isoquinolines

5-methylisoquinoline (SI-1c):

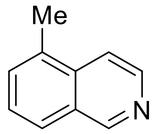

SI-1c

Prepared according to **General Procedure A**, using 5-bromoisoquinoline (416 mg, 2.00 mmol) and $MeB(OH)_2$ (239 mg, 4.00 mmol), leaving the reaction overnight. Purification by flash column chromatography on SiO_2 (5:1 → 4:1 pentane/EtOAc) afforded the product **SI-1c** as a clear colourless oil which slowly crystallised over time (249 mg, 87%). Spectroscopic data for **SI-1c** was consistent with that which has been reported in the literature.[5]

Data for **SI-1c**:

^{1}H NMR (400 MHz, $CDCl_3$) δ 9.24 (d, *J* = 1.0 Hz, 1H), 8.56 (d, *J* = 6.0 Hz), 7.82 (dd, *J* = 8.1, 1.5 Hz, 1H), 7.76 (d, *J* = 6.0 Hz, 1H), 7.56 – 7.46 (m, 2H), 2.68 (s, 3H).
^{13}C NMR (101 MHz, $CDCl_3$) δ 153.1, 143.2, 135.4, 133.7, 130.7, 128.9, 127.1, 125.9, 117.2, 18.6.

5-phenylisoquinoline (SI-1d):

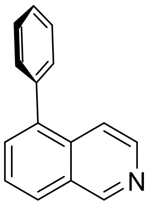

SI-1d

Prepared according to **General Procedure A**, using 5-bromoisoquinoline (416 mg, 2.00 mmol) and $PhB(OH)_2$ (488 mg, 4.00 mmol), leaving the reaction overnight. Purification by flash column chromatography on SiO_2 (8:1 → 4:1 pentane/EtOAc) afforded the product **SI-1d** as a light yellow oil (344 mg, 83%). Spectroscopic data for **SI-1d** was consistent with that which has been reported in the literature.[6]

Data for **SI-1d**:

^{1}H NMR (400 MHz, $CDCl_3$) δ 9.31 (s, 1H), 8.49 (d, *J* = 6.0 Hz, 1H), 8.03 – 7.96 (m, 1H), 7.72 (d, *J* = 6.0 Hz, 1H), 7.68 – 7.65 (m, 2H), 7.56 – 7.44 (m, 5H).
^{13}C NMR (101 MHz, $CDCl_3$) δ 153.0, 143.5, 139.4, 139.2, 134.3, 131.0, 130.0, 129.1, 128.7, 127.9, 127.3, 127.0, 118.7.

5-methoxyisoquinoline (SI-1g):

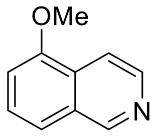

SI-1g

NaH (60% dispersion in parafins, 96 mg, 2.4 mmol, 2.2 equiv.) was washed with pentane (5 mL × 3). The NaH was taken up in DMF (6 mL) and cooled to 0 °C, then 5-hydroxyisoquinoline (290 mg, 2.00 mmol) was added slowly and stirred for 10 min. To the resultant salt, MeI (0.14 mL, 2.2 mmol) was added and the mixture was stirred at 0 °C for a further 1.5 h. The reaction was quenched by pouring into water (100 mL) and was then extracted into EtOAc (40 mL × 5). The combined organic extracts were then washed with water (100 mL) and brine (100 mL), then dried over $MgSO_4$, filtered, and concentrated *in vacuo*. The residue was purified by flash column chromatography on SiO_2 (10:1 Et_2O/acetone) to afford **SI-1g** as a brown oil (278 mg, 87%). Spectroscopic data for **SI-1g** was consistent with that which has been reported in the literature.[7]

Data for **SI-1g**:

^{1}H NMR (600 MHz, $CDCl_3$) δ 9.20 (s, 1H), 8.52 (d, *J* = 5.8 Hz, 1H), 8.00 (dd, *J* = 5.8, 1.0 Hz, 1H), 7.53 (dd, *J* = 8.2, 1.1 Hz, 1H), 7.50 (dd, *J* = 8.2, 7.4 Hz, 1H), 6.99 (d, *J* = 7.3 Hz, 1H), 4.01 (s, 3H).
^{13}C NMR (151 MHz, $CDCl_3$) δ 154.6, 151.9, 142.8, 129.6, 128.6, 127.5, 119.4, 115.2, 107.7, 55.8.

6-phenylisoquinoline (SI-1i):

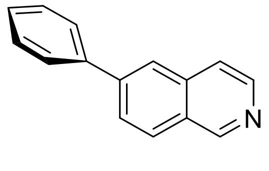

SI-1i

Prepared according to **General Procedure A**, using 6-bromoisoquinoline (208 mg, 1.00 mmol) and $PhB(OH)_2$ (57.8 mg, 4.00 mmol), leaving the reaction overnight. Purification by flash column chromatography on SiO_2 (4:1 → 2:1 pentane/EtOAc) afforded the product **SI-1i** as a white solid (210 mg, 102%, quant.). Spectroscopic data for **SI-1i** was consistent with that which has been reported in the literature.[6]

Data for **SI-1i**:

^{1}H NMR (400 MHz, $CDCl_3$) δ 9.29 (s, 1H), 8.58 – 8.53 (m, 1H), 8.07 – 8.02 (m, 1H), 8.00 (d, *J* = 1.9 Hz, 1H), 7.87 (dt, *J* = 8.5, 1.4 Hz, 1H), 7.76 – 7.69 (m, 3H), 7.56 – 7.48 (m, 2H), 7.44 (dd, *J* = 6.8, 1.5 Hz, 1H).

^{13}C NMR (101 MHz, $CDCl_3$) δ 152.3, 143.5, 143.3, 140.3, 136.3, 129.2, 128.3, 127.9, 127.7, 127.3, 124.3, 120.8.

7-methylisoquinoline (SI-1l):

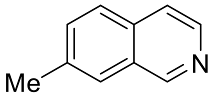

SI-1l

Prepared according to **General Procedure A**, using 7-bromoisoquinoline (416 mg, 2.00 mmol) and $MeB(OH)_2$ (239 mg, 4.00 mmol), leaving the reaction overnight. Purification by flash column chromatography on SiO_2 (8:1 → 4:1 pentane/EtOAc) afforded the product **SI-1l** as a white solid (261 mg, 91%). Spectroscopic data for **SI-1i** was consistent with that which has been reported in the literature.[8]

Data for **SI-1l**:

^{1}H NMR (400 MHz, $CDCl_3$) δ 9.18 (s, 1H), 8.47 (d, *J* = 5.7 Hz, 1H), 7.76 – 7.71 (m, 2H), 7.61 (d, *J* = 5.7 Hz, 1H), 7.54 (dd, *J* = 8.8, 1.5 Hz, 1H), 2.56 (s, 3H).
^{13}C NMR (101 MHz, $CDCl_3$) δ 151.9, 142.3, 137.2, 134.1, 132.7, 128.3, 126.4, 126.3, 120.2, 21.8.

7-phenylisoquinoline (SI-1m):

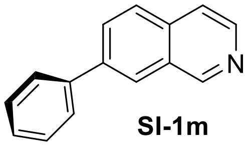

SI-1m

Prepared according to **General Procedure A**, using 7-bromoisoquinoline (416 mg, 2.00 mmol) and $PhB(OH)_2$ (488 mg, 4.00 mmol), leaving the reaction overnight. Purification by flash column chromatography on SiO_2 (4:1 pentane/EtOAc) afforded the product **SI-1m** as a light-yellow oil (372 mg, 91%).

Data for **SI-1m**:

^{1}H NMR (400 MHz, $CDCl_3$) δ 9.34 (s, 1H), 8.56 (d, *J* = 5.8 Hz, 1H), 8.18 – 8.13 (m, 1H), 7.97 (dd, *J* = 8.6, 1.8 Hz, 1H), 7.89 (br d, *J* = 8.5 Hz, 1H), 7.75 – 7.67 (m, 2H), 7.68 (d, *J* = 5.9 Hz, 1H), 7.54 – 7.48 (m, 2H), 7.44 – 7.40 (m, 1H).
^{13}C NMR (101 MHz, $CDCl_3$) δ 152.7, 142.8, 140.3, 140.2, 135.0, 130.3, 129.2, 129.1, 128.0, 127.5, 127.2, 125.4, 120.5.

8-methylisoquinoline (SI-1o):

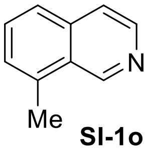
SI-1o

Prepared according to **General Procedure A**, using 8-bromoisoquinoline (832 mg, 4.00 mmol) and $PhB(OH)_2$ (479 mg, 8.00 mmol), leaving the reaction overnight. Purification by flash column chromatography on SiO_2 (4:1 → 3:1 pentane/EtOAc) afforded the product **SI-1o** as a yellow oil (374 mg, 65%).

Data for **SI-1o**:

^{1}H NMR (400 MHz, $CDCl_3$) δ 9.46 (d, *J* = 1.0 Hz, 1H), 8.55 (d, *J* = 5.7 Hz, 1H), 7.67 (d, *J* = 8.3 Hz, 1H), 7.64 (dd, *J* = 5.7, 1.0 Hz, 1H), 7.57 (dd, *J* = 8.3, 7.0 Hz, 1H), 7.39 (dq, *J* = 7.0, 1.0 Hz, 1H), 2.80 (s, 3H).
^{13}C NMR (101 MHz, $CDCl_3$) δ 149.5, 143.0, 136.3, 135.6, 130.3, 128.2, 127.8, 125.0, 121.0, 18.6.

8-phenylisoquinoline (SI-1p):

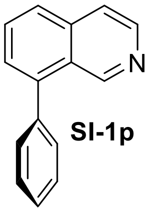
SI-1p

8-Bromoisoquinoline (208 mg, 1.00 mmol, 1.0 equiv.), $PhB(OH)_2$ (183 mg, 1.50 mmol, 1.5 equiv.), Na_2CO_3 (190 mg, 1.80 mmol, 1.8 equiv.) and $Pd(PPh_3)_4$ (57.8 mg, 0.0500 mmol, 5.0 mol%) were combined and taken up in 2:1 dioxane/water (6 mL). The mixture was sparged with N_2 then heated to reflux overnight. The reaction was cooled to ambient temperature and water (20 mL) was added and the mixture was extracted with EtOAc (15 mL × 2). The combined organic extracts were dried over $MgSO_4$, filtered, and concentrated *in vacuo*. The residue was purified by flash column chromatography on SiO_2 (4:1 → 2:1 pentane/EtOAc) to afford the product **SI-1p** as a white solid (179 mg, 87%). Spectroscopic data for **SI-1i** was consistent with that which has been reported in the literature.[9]

Data for **SI-1p**:

^{1}H NMR (400 MHz, $CDCl_3$) δ 9.32 (s, 1H), 8.56 (d, *J* = 5.7 Hz, 1H), 7.82 (d, *J* = 8.3 Hz, 1H), 7.74 (d, *J* = 7.1 Hz, 1H), 7.70 (d, *J* = 5.4 Hz, 1H), 7.56 – 7.45 (m, 6H).
^{13}C NMR (101 MHz, $CDCl_3$) δ 151.3, 143.0, 141.2, 138.8, 136.3, 130.2, 130.0, 128.6, 128.3, 128.0, 126.8, 126.1, 120.6.

2.3 Synthesis of Vinyl Ketones

4-methylpent-1-en-3-one (SI-2)

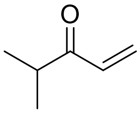

SI-2

Prepared according to **General Procedure B** with no purification of the Weinreb amide intermediate. The crude vinyl ketone was purified by flash column chromatography on SiO_2 (10:1 pentane/Et_2O) to afford the *i*-propyl vinyl ketone (**SI-2**) as a clear colourless oil (444 mg, 45% over 2 steps). Spectroscopic data for **SI-2** was consistent with that which has been reported in the literature.[10]

N.B. **SI-2** is volatile, care must be taken when concentrating *in vacuo* as well as with handling as it is presumably toxic.

Data for **SI-2**:

^{1}H NMR (400 MHz, $CDCl_3$) δ 6.44 (ddd, *J* = 17.5, 10.5, 0.8 Hz, 1H), 6.27 (ddd, *J* = 17.5, 1.5, 0.8 Hz, 1H), 5.77 (ddd, *J* = 10.5, 1.5, 0.8 Hz, 1H), 2.89 (pd, *J* = 6.9, 0.8 Hz, 1H), 1.12 (dt, *J* = 6.9, 0.8 Hz, 6H).

^{13}C NMR (101 MHz, $CDCl_3$) δ 204.3, 134.9, 128.1, 38.3, 18.4.

1-phenylbut-3-en-2-one (SI-3):

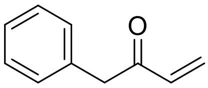

SI-3

Prepared according to **General Procedure B** using phenylacetyl chloride (3.09 g, 20.0 mmol). The intermediate Weinreb amide was purified by flash column chromatography on SiO_2 (4:1 pentane/EtOAc). The crude vinyl ketone product was purified by flash column chromatography on SiO_2 (10:1 pentane/Et_2O) to afford the benzyl vinyl ketone (**SI-3**) as a clear colourless oil (1.57 g, 54% over 2 steps). Spectroscopic data for **SI-3** was consistent with that which has been reported in the literature.[11]

Data for **SI-3**:

^{1}H NMR (400 MHz, $CDCl_3$) δ 7.41 – 7.34 (m, 1H), 7.32 – 7.27 (m, 1H), 7.27 – 7.23 (m, 2H), 6.45 (dd, *J* = 17.6, 10.2 Hz, 1H), 6.34 (dd, *J* = 17.6, 1.4 Hz, 1H), 5.86 (dd, *J* = 10.2, 1.4 Hz, 1H), 3.92 (s, 2H).

^{13}C NMR (101 MHz, $CDCl_3$) δ 197.9, 135.7, 134.2, 129.6, 129.2, 128.9, 127.2, 47.3.

1-phenylprop-2-en-1-one (SI-4):

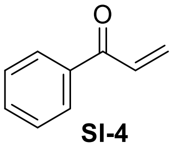

SI-4

A 100 mL flask was flame dried then charged with anhydrous THF (30 mL). Commercially available Weinreb amide of benzoic acid (2.00 g, 12.0 mmol, 1.0 equiv.) was added and the solution cooled to 0 °C. Vinyl magnesium bromide (14.4 mL, 14.4 mmol, 1.0 M in THF, 1.2 equiv.) was added dropwise and the solution stirred at rt for 1 h. The reaction was quenched with 2.0 M aq. HCl, diluted with Et_2O, the layers partitioned and the aqueous layer extracted twice more with Et_2O. The combined organic extracts were dried over $MgSO_4$, filtered under gravity and concentrated *in vacuo*. Purification by flash column chromatography on SiO_2 (10% Et_2O in pentane) afforded **SI-4** as a pale-yellow oil (0.62 g, 78%). Spectroscopic data for **SI-4** was consistent with that which has been reported in the literature.[11]

Data for **SI-4**:

^{1}H NMR (400 MHz, $CDCl_3$) δ 7.99 – 7.92 (2H, m), 7.63 – 7.54 (m, 1H), 7.54 – 7.44 (m, 2H), 7.16 (dd, *J* = 17.2, 10.6 Hz, 1H), 6.44 (dd, *J* = 17.1, 1.7 Hz, 1H), 5.94 (1H, dd, *J* = 10.5, 1.7 Hz, 1H).
^{13}C NMR (101 MHz, $CDCl_3$) δ 191.2, 137.4, 133.1, 132.6, 130.3, 128.9, 128.8.

1-(4-(trifluoromethyl)phenyl)prop-2-en-1-one (SI-5):

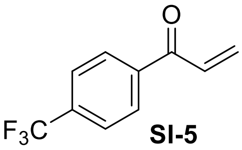

SI-5

Prepared according to **General Procedure B** using *p*-trifluoromethylbenzoyl chloride (2.10 g, 10.0 mmol) The crude vinyl ketone product was purified by flash column chromatography on SiO_2 (10% Et_2O in pentane) afford **SI-5** as a clear colourless oil (630 mg, 32%). Spectroscopic data for **SI-4** was consistent with that which has been reported in the literature.[12]

Data for **SI-5**:

^{1}H NMR (600 MHz, $CDCl_3$) δ 8.06 – 7.99 (m, 2H), 7.75 (dt, *J* = 8.1, 0.8 Hz, 1H), 7.13 (dd, *J* = 17.2, 10.6 Hz, 1H), 6.47 (dd, *J* = 17.2, 1.5 Hz, 1H), 6.02 (dd, *J* = 10.6, 1.4 Hz, 1H).

^{13}C NMR (151 MHz, $CDCl_3$) δ 190.4, 140.2, 134.4 (q, *J* = 32.7 Hz), 132.3, 131.6, 129.1, 125.8 (q, *J* = 3.7 Hz), 123.8 (q, *J* = 272.7 Hz).

^{19}F NMR (377 MHz, $CDCl_3$): –63.1.

1-(furan-2-yl)prop-2-en-1-one (SI-6):

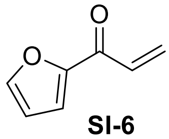

SI-6

Prepared according to **General Procedure A** with no purification of the intermediate Weinreb amide. The crude furoyl vinyl ketone **SI-6** was uses without purification by chromatography and was a pale yellow oil (1.10 g, 88%). Data for **SI-6** matched what had previously been described in the literature.[11]

Data for **SI-6**:

^{1}H NMR (400 MHz, $CDCl_3$) δ 7.63 (dd, *J* = 1.7, 0.8 Hz, 1H), 7.27 (dd, *J* = 3.6, 0.8 Hz, 1H), 7.06 (dd, *J* = 17.2, 10.5 Hz, 1H), 6.56 (d, *J* = 1.6 Hz, 1H), 6.55 (dd, *J* = 20.5, 1.8 Hz, 1H), 5.87 (1H, dd, *J* = 10.5, 1.7 Hz, 1H)

^{13}C NMR (101 MHz, $CDCl_3$) 178.2, 153.1, 147.1, 131.5, 129.6, 118.4, 112.6.

2.4 C-4 Alkylation of Isoquinolines

4-(isoquinolin-4-yl)butan-2-one[13,14] (3a)

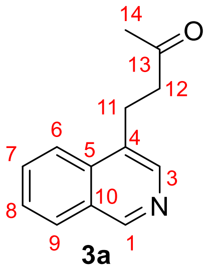

3a

Prepared according to a scaled-down version of **General Procedure C**, using isoquinoline (16.2 mg, 0.125 mmol, 1.00 equiv.) and methyl vinyl ketone (42 µL, 0.50 mmol, 4.0 equiv.) and after work up was analysed by qNMR using trimethoxybenzene (1/3 mol equiv.) as an internal standard which showed **3a** was formed in 73% yield.

Additionally, this reaction was performed on a gram-scale using isoquinoline (**1a**) (1.03 g, 8.00 mmol) and methyl vinyl ketone (2.61 mL, 32.0 mmol). Purification using flash column chromatography on SiO_2 (0.5 → 2% MeOH in CH_2Cl_2) afforded **3a** as a yellow oil (970 mg, 61%).

Data for **3a**:

^{1}H NMR (600 MHz, $CDCl_3$): 9.12 (1H, s, C^1**H**), 8.37 (1H, s, C^3**H**), 7.99 – 7.91 (2H, m, 2×$C^{6,9}$**H**), 7.72 (1H, dddt, J = 8.2, 6.9, 2.5, 1.3 Hz, C^7**H**), 7.59 (1H, dddt, J = 8.1, 6.9, 2.4, 1.2 Hz, C^8**H**), 3.28 (2H, td, J = 7.7, 3.3 Hz, C^{11}**H**$_2$), 2.87 (2H, td, J = 7.8, 2.9 Hz, C^{12}**H**$_2$), 2.16 (3H, s, C^{14}**H**$_3$).
^{13}C NMR (101 MHz, $CDCl_3$) δ 207.2 (**C**^{13}), 151.6 (**C**^{1}H), 142.5 (**C**^{3}H), 134.4 (C^5), 130.5 (**C**^{7}H), 130.2 (**C**^{4}H), 128.5 (2×**C**^{9,10}H), 127.0 (**C**^{8}H), 122.6 (**C**^{6}H), 44.0 (**C**^{12}H_2), 30.1 (**C**^{14}H_3), 23.7 (**C**^{11}H_2)
IR (neat): ν 1713, 1653, 1623, 1585, 1504, 1411, 1391, 1366, 1293, 1231, 1165, 1109 cm^{-1}.
HRMS (ESI^+) m/z calc. for $C_{13}H_{14}NO$ $[M+H]^+$ 200.1070; found 200.1067, Δ = –1.50 ppm

4-(3-methylisoquinolin-4-yl)butan-2-one (3b):

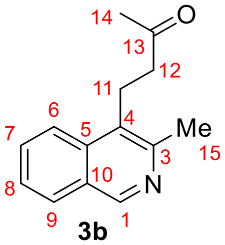

3b

Prepared according to a scaled-up version of **General Procedure C**, using 3-methylisoquinoline (286 mg, 2.00 mmol) and methyl vinyl ketone (0.66 mL, 8.00 mmol) and was purified by flash column chromatography on SiO_2 (20:1→10:1 Et_2O/acetone) to afford **3b** as a yellow oil, which slowly solidified (266 mg, 62%).

Data for **3b**:

^{1}H NMR (600 MHz, $CDCl_3$) δ 9.06 (s, 1H, C^{1}**H**), 7.93 (d, *J* = 8.1 Hz, 1H, C^{9}**H**), 7.90 (d, *J* = 8.6 Hz, 1H, C^{6}**H**), 7.70 (t, *J* = 7.7 Hz, 1H, C^{8}**H**), 7.52 (t, *J* = 7.5 Hz, 1H, C^{7}**H**), 3.32 (t, *J* = 8.0 Hz, 2H, C^{13}**H**$_2$), 2.74 (t, *J* = 8.4 Hz, 2H, C^{12}**H**$_2$), 2.70 (s, 3H, C^{15}**H**$_3$), 2.19 (s, 3H, C^{11}**H**$_3$).
^{13}C NMR (150 MHz, $CDCl_3$) δ 207.7 (**C**^{13}), 150.5 (**C**^{1}H), 149.3 (**C**^{3}), 135.0 (**C**^{10}), 130.6 (**C**^{8}H), 128.5 (**C**^{9}H), 127.4 (**C**^{5}), 126.9 (**C**^{4}), 126.0 (**C**^{7}H), 122.3 (**C**^{6}H), 43.3 (**C**^{12}H), 30.1 (**C**^{14}H$_3$), 22.4 (**C**^{15}H$_3$), 22.0 (**C**^{11}H$_2$).
HRMS (ESI$^+$) calc. for $C_{14}H_{16}NO$ $[M+H]^+$ 214.1226; found 214.1224, Δ = –0.93 ppm.
IR (neat) ν 3657, 2981, 2889, 2360, 2341, 1710, 1624, 1383, 1250, 1155 cm^{-1}.

4-(5-methylisoquinolin-4-yl)butan-2-one (3c):

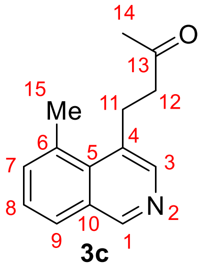

3c

Prepared according to **General Procedure C**, using 5-methylisoquinoline (**SI-1c**) (35.8 mg, 0.250 mmol) and methyl vinyl ketone (82 μL, 1.00 mmol) and was purified by flash column chromatography on SiO_2 (20:1 → 10:1 Et_2O/acetone) to afford **3c** as a white solid (26.4 mg, 50%).

Data for **3c**:

^{1}H NMR (600 MHz, $CDCl_3$) δ 9.06 (s, 1H, C^{1}**H**), 8.30 (s, 1H, C^{3}**H**), 7.81 (d, *J* = 7.7 Hz, 1H, C^{9}**H**), 7.49 (d, *J* = 6.2 Hz, 1H, C^{7}**H**), 7.45 (t, *J* = 7.5 Hz, 1H, C^{8}**H**), 3.47 (t, *J* = 7.8 Hz, 2H, C^{11}**H**$_2$), 2.85 (s, 3H, C^{15}**H**), 2.78 (t, *J* = 8.0 Hz, 2H, C^{12}**H**$_2$), 2.17 (s, 3H, C^{14}**H**$_3$).
^{13}C NMR (151 MHz, $CDCl_3$) δ 207.0 (**C**^{13}), 153.0 (**C**^{1}H), 145.1 (**C**^{3}H), 134.6 (**C**^{5}), 134.0 (**C**^{7}H), 133.7 (**C**^{6}), 131.2 (**C**^{4}), 130.1 (**C**^{10}), 127.8 (**C**^{9}H), 126.7 (**C**^{8}H), 46.5 (**C**^{12}H$_2$), 30.2 (**C**^{14}H$_3$), 27.4 (**C**^{11}H$_2$), 24.5 (**C**^{15}H$_3$).
IR (neat): ν 1714, 1612, 1585, 1326 cm^{-1}.
HRMS (ESI$^+$) m/z calc. for $C_{14}H_{15}NONa$ $[M+Na]^+$ 236.1046; found 236.1051, Δ = 2.16 ppm.
Mp = 55-56 °C (amorphous).

4-(5-phenylisoquinolin-4-yl)butan-2-one (3d):

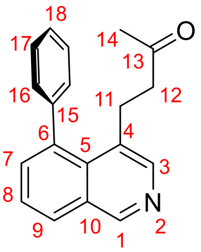

3d

Prepared according to **General Procedure C**, using 5-phenylisoquinoline (**SI-1d**) (51.3 mg, 0.250 mmol) and methyl vinyl ketone (82 μL, 1.00 mmol) and was purified by flash column chromatography on SiO_2 (20:1 Et_2O/acetone) to afford **3d** as a white solid (59.5 mg, 86%).

Data for **3d**:

^{1}H NMR (500 MHz, $CDCl_3$) δ 9.18 (s, 1H, C^1**H**), 8.31 (s, 1H, C^3**H**), 8.00 (dd, J = 8.1, 1.5 Hz, 1H, C^9**H**), 7.61 (dd, J = 8.1, 7.1 Hz, 1H, C^8**H**), 7.53 (dd, J = 7.0, 1.4 Hz, 1H, C^7**H**), 7.44 – 7.40 (m, 3H, 3×C_{Ph}**H**), 7.39 – 7.34 (m, 2H, 2×C_{Ph}**H**), 2.70 (t, J = 7.9 Hz, 2H, C^{11}**H**$_2$), 2.33 (t, J = 8.5 Hz, 2H, C^{12}**H**), 1.87 (s, 3H, C^{14}**H**$_3$).

^{13}C NMR (126 MHz, $CDCl_3$) δ 207.0 (**C**^{13}), 152.7 (**C**^{1}H), 145.5 (**C**^{3}), 142.9 (**C**^{15}), 139.0 (**C**^{6}), 134.2 (**C**^{7}H), 132.7 (**C**^{5}), 131.1 (**C**^{4}), 129.8 (**C**^{10}), 129.5 (2×**C**$^{16/17}$H), 128.9 (**C**^{9}H), 128.2 (2×**C**$^{16/17}$H), 127.9 (**C**^{18}H), 126.1 (**C**^{8}H), 45.5 (**C**^{12}H$_2$), 29.6 (**C**^{14}H$_3$), 26.9 (**C**^{11}H$_2$).

IR ν 1715, 1579, 1362, 1162, 767 cm^{-1}.

HRMS (ESI$^+$) m/z calc. for $C_{19}H_{17}NONa$ [M+Na]$^+$ 298.1202; found 298.1217, Δ = 4.90 ppm.

Mp = 83-85 °C (amorphous).

4-(5-bromoisoquinolin-4-yl)butan-2-one (3e):

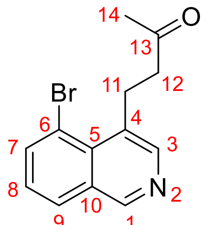

3e

Prepared according to **General Procedure C**, using 5-bromoisoquinoline (52.0 mg, 0.250 mmol) and methyl vinyl ketone (82 μL, 1.00 mmol) and was purified by flash column chromatography on SiO_2 (20:1 Et_2O/acetone) to afford **3e** as a pale yellow oil (18.9 mg, 27%).

Data for **3e**:

^{1}H NMR (600 MHz, $CDCl_3$) δ 9.12 (s, 1H, C^1**H**), 8.42 (s, 1H, C^3**H**), 8.03 (dd, J = 7.5, 1.3 Hz, 1H, C^9**H**), 7.95 (dd, J = 8.1, 1.3 Hz, 1H, C^7**H**), 7.41 (t, J = 7.8 Hz, 1H, C^8**H**), 3.71 (t, J = 7.9 Hz, 2H, C^{11}**H**$_2$), 2.89 (t, J = 7.9 Hz, 2H, C^{12}**H**$_2$), 2.18 (s, 3H, C^{14}**H**$_3$).

^{13}**C NMR** (151 MHz, $CDCl_3$) δ 207.2 (**C**^{13}), 153.2 (**C**^{1}H), 147.0 (**C**^{3}H), 137.8 (**C**^{7}H), 133.3 (**C**^{5}), 131.0 (**C**^{10}), 130.5 (**C**^{4}), 129.4 (**C**^{9}H), 127.4 (**C**^{8}H), 117.9 (**C**^{6}), 47.0 (**C**^{12}H_2), 30.2 (**C**^{14}H_3), 26.9 (**C**^{11}H_2).

IR (neat) ν 1714, 1372, 1202, 1164 cm^{-1}.

HRMS (ESI^+) m/z calc. for $C_{13}H_{13}BrNO$ $[M+H]^+$ 278.0175; found 278.0181, Δ = 2.14 ppm.

4-(5-chloroisoquinolin-4-yl)butan-2-one (3f):

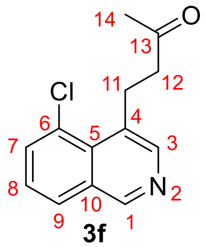

3f

Prepared according to **General Procedure C**, using 5-chloroisoquinoline (40.9 mg, 0.250 mmol) and methyl vinyl ketone (82 µL, 1.00 mmol) and was purified by flash column chromatography on SiO_2 (15:5:2 pentane/EtOAc/acetone) to afford **3f** as a pale yellow oil (23.0 mg, 39%).

Data for **3f**:

^{1}**H NMR** (600 MHz, $CDCl_3$) δ 9.11 (s, 1H, C^{1}**H**), 8.40 (s, 1H, C^{3}**H**), 7.90 (dd, *J* = 8.1, 1.3 Hz, 1H, C^{9}**H**), 7.77 (dd, *J* = 7.5, 1.3 Hz, 1H C^{7}**H**), 7.49 (t, *J* = 7.8 Hz, 1H, C^{8}**H**), 3.64 (t, *J* = 7.8 Hz, 2H, C^{11}**H**$_2$), 2.88 (t, *J* = 8.2 Hz, 2H, C^{12}**H**$_2$), 2.17 (s, 3H, C^{14}**H**$_3$).

^{13}**C NMR** (151 MHz, $CDCl_3$) δ 207.3 (**C**^{13}), 152.9 (**C**^{1}H), 146.8 (**C**^{3}H), 133.5 (**C**^{7}H), 132.2 (**C**^{5}), 130.9 (**C**^{6}), 130.3 (**C**^{4}), 129.9 (**C**^{10}), 128.6 (**C**^{9}H), 127.0 (**C**^{8}H), 46.7 (**C**^{12}H_2), 30.1 (**C**^{14}H_3), 27.4 (**C**^{11}H_2).

HRMS (ESI^+) m/z calc. for $C_{13}H_{14}ClNO$ $[M+H]^+$ 234.0680; found 234.0685, Δ = 2.04 ppm.

IR (neat) ν 1714, 1579, 1375, 1204, 1163, 1087 cm^{-1}.

4-(5-methoxyisoquinolin-4-yl)butan-2-one (3g):

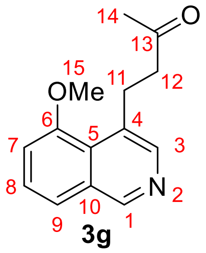

3g

Prepared according to **General Procedure C**, using 5-methoxyisoquinoline (**SI-1g**) (39.8 mg, 0.250 mmol) and methyl vinyl ketone (82 µL, 1.00 mmol) and was purified by flash column chromatography on SiO_2 (20:1 Et_2O/acetone) to afford **3g** as an orange/brown oil (28.4 mg, 49%).

Data for **3g**:

^{1}**H NMR** (400 MHz, $CDCl_3$) δ 9.02 (s, 1H, C^{1}**H**), 8.26 (s, 1H, C^{3}**H**), 7.56 – 7.45 (m, 2H), 7.02 (dd, *J* = 7.4, 1.5 Hz, 1H), 3.94 (s, 3H, C^{15}**H**$_3$), 3.43 (dd, *J* = 8.5, 7.0 Hz, 2H, C^{11}**H**$_2$), 2.79 (dd, *J* = 8.6, 6.9 Hz, 2H, C^{12}**H**$_2$), 2.16 (s, 3H, C^{14}**H**$_3$).

^{13}C NMR (100 MHz, $CDCl_3$) δ 208.2 (**C**^{13}), 156.1 (**C**^{6}), 151.5 (**C**^{1}H), 144.7 (**C**^{3}H), 130.6 (**C**$_q$), 127.3 (**C**^{8}H), 127.1 (**C**$_q$), 120.7 (**C**^{9}H), 109.2 (**C**^{7}H), 55.6 (**C**^{15}H_3), 46.5 (**C**^{12}H_2), 30.1 (**C**^{14}H_3), 28.4 (**C**^{11}H_2).

IR (neat) ν 1716, 1613, 1356, 1072 cm^{-1}.

HRMS (ESI$^+$) m/z calc. for $C_{14}H_{15}NO_2Na$ [M+Na]$^+$ 252.0995; found 252.1006, Δ = 4.35 ppm.

4-(6-methylisoquinolin-4-yl)butan-2-one (3h):

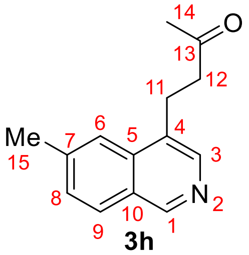

3h

Prepared according to **General Procedure C**, using 6-methylisoquinoline (39.8 mg, 0.250 mmol) and methyl vinyl ketone (82 µL, 1.0 mmol) and was purified by flash column chromatography on SiO_2 (20:1 Et_2O/acetone) to afford **3h** as a pale yellow oil (31.7 mg, 59%).

Data for **3h**:

^{1}H NMR (500 MHz, $CDCl_3$) δ 9.06 (s, 1H, C^{1}**H**), 8.33 (s, 1H, C^{3}**H**), 7.87 (d, *J* = 8.3 Hz, 1H, C^{9}**H**), 7.70 (br s, 1H, C^{6}**H**), 7.44 (dd, *J* = 8.3, 1.5 Hz, 1H, C^{8}**H**), 3.27 (t, *J* = 7.7 Hz, 2H, C^{11}**H**$_2$), 2.88 (t, *J* = 8.1 Hz, 2H, C^{12}**H**$_2$), 2.58 (s, 3H, C^{15}**H**$_3$), 2.19 (s, 3H, C^{14}**H**$_3$).

^{13}C NMR (126 MHz, $CDCl_3$) δ 207.5 (**C**^{13}), 151.2 (**C**^{1}H), 142.5 (**C**^{3}H), 141.1 (**C**^{7}), 134.8 (**C**^{5}), 129.7 (**C**^{4}), 129.4 (**C**^{8}H), 128.4 (**C**^{9}H), 127.0 (**C**^{10}), 121.6 (**C**^{6}H), 44.1 (**C**^{12}H_2), 30.2 (**C**^{14}H_3), 23.8 (**C**^{11}H_2), 22.6 (**C**^{15}H_3).

IR ν 1716, 1628, 1499, 1365, 669 cm^{-1}.

HRMS (ESI$^+$) m/z calc. for $C_{14}H_{15}NONa$ [M+Na]$^+$ 236.1046; found 236.1036, Δ = –4.19 ppm.

4-(6-phenylisoquinolin-4-yl)butan-2-one (3i):

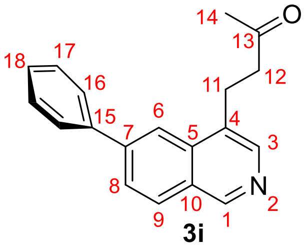

3i

Prepared according to **General Procedure C**, using 6-phenylisoquinoline (**SI-1i**) (51.3 mg, 0.250 mmol) and methyl vinyl ketone (82 µL, 1.0 mmol) and was purified by flash column chromatography on SiO_2 (20:1 →10:1 Et_2O/acetone) to afford **3i** as an orange/brown oil (55.2 mg, 80%).

Data for **3i**:

^{1}H NMR (600 MHz, $CDCl_3$) δ 9.15 (s, 1H, C^{1}**H**), 8.41 (s, 1H, C^{3}**H**), 8.10 (s, 1H, C^{6}**H**), 8.05 (d, J = 8.5 Hz, 1H, C^{9}**H**), 7.86 (dd, J = 8.4, 1.7 Hz, 1H, C^{8}**H**), 7.71 (d, J = 7.1 Hz, 2H, 2×C^{16}**H**), 7.53 (t, J = 7.6 Hz, 2H, 2×C^{17}**H**), 7.45 (t, J = 7.4 Hz, 1H, C^{18}**H**), 3.36 (t, J = 7.8 Hz, 2H, C^{11}**H_2**), 2.93 (t, J = 7.7 Hz, 2H, C^{12}**H_2**), 2.18 (s, 3H, C^{14}**H_3**).

^{13}C NMR (151 MHz, $CDCl_3$) δ 207.3 (**C^{13}**), 151.5 (**C^{1}**H), 143.4 (**C^{7}**), 143.1 (**C^{3}**H), 140.6 (**C^{15}**), 134.9 (**C^{5}**), 130.4 (**C^{4}**), 129.2 (2×**C^{17}**H), 129.1 (**C^{9}**H), 128.4 (**C^{18}**H), 127.9 (2×**C^{16}**H), 127.6 (**C^{10}**), 127.0 (**C^{8}**H), 120.5 (**C^{6}**H), 44.1 (**C^{12}**H_2), 30.3 (**C^{14}**H_3), 23.8 (**C^{11}**H_2).

IR (neat) ν 1715, 1623, 1488, 1361, 1163 cm^{-1}.

HRMS m/z calc. for $C_{19}H_{17}NONa$ $[M+Na]^+$ 298.1202; found 298.1212, Δ = 3.22 ppm.

4-(6-bromoisoquinolin-4-yl)butan-2-one (3j):

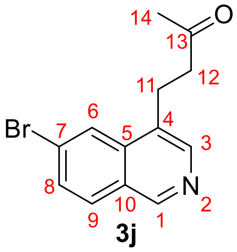

3j

Prepared according to **General Procedure C**, using 6-bromoisoquinoline (52.0 mg, 0.250 mmol) and methyl vinyl ketone (82 µL, 1.0 mmol) and was purified by flash column chromatography on SiO_2 (20:1 →10:1 Et_2O/acetone) to afford **3j** as clear colourless oil (39.0 mg, 56%).

Data for **3j**:

^{1}H NMR (600 MHz, $CDCl_3$) δ 9.08 (s, 1H, C^{1}**H**), 8.39 (s, 1H, C^{3}**H**), 8.10 (s, 1H, C^{6}**H**), 7.83 (d, J = 8.7 Hz, 1H, C^{9}**H**), 7.67 (dd, J = 8.7, 1.8 Hz, 1H, C^{8}**H**), 3.23 (t, J = 7.7 Hz, 2H, C^{11}**H_2**), 2.87 (t, J = 7.7 Hz, 2H, C^{12}**H_2**), 2.18 (s, 3H, C^{14}**H_3**).

^{13}C NMR (151 MHz, $CDCl_3$) δ 206.9 (**C^{13}**), 151.4 (**C^{1}**H), 143.6 (**C^{3}**H), 135.7 (**C^{10}**), 130.8 (**C^{8}**H), 130.1 (**C^{9}**H), 129.3 (**C^{4}**), 126.8 (**C^{5}**), 125.7 (**C^{7}**), 125.3 (**C^{6}**H), 43.8 (**C^{12}**H_2), 30.2 (**C^{14}**H_3), 23.5 (**C^{11}**H_2).

IR 1716, 1613, 1356, 1072.

HRMS m/z calc. for $C_{13}H_{14}BrNO$ $[M+H]^+$ 278.0175; found 278.0185, Δ = 3.58 ppm.

4-(6-methoxyisoquinolin-4-yl)butan-2-one (3k):

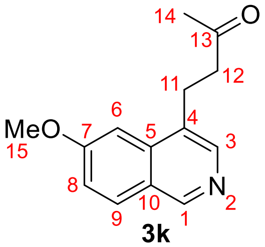

3k

Prepared according to **General Procedure C**, using 6-methoxyisoquinoline (39.8 mg, 0.250 mmol) and methyl vinyl ketone (82 µL, 1.0 mmol) and was purified by flash column chromatography on SiO_2 (10:1 Et_2O/acetone) to afford **3k** as clear colourless oil (23.1 mg, 40%).

Data for **3k**:

^{1}H NMR (600 MHz, $CDCl_3$) δ 9.00 (s, 1H, C^1**H**), 8.31 (s, 1H, C^3**H**), 7.88 (d, J = 8.9 Hz, 1H, C^9**H**), 7.24 (dd, J = 8.9, 2.4 Hz, 1H, C^8**H**), 7.17 (d, J = 2.4 Hz, 1H, C^6**H**), 3.97 (s, 3H, C^{15}**H**$_3$), 3.26 (t, J = 7.7 Hz, 2H, C^{11}**H**$_2$), 2.89 (t, J = 7.9 Hz, 2H, C^{12}**H**$_2$), 2.19 (s, 3H, C^{14}**H**$_3$).

^{13}C NMR (151 MHz, $CDCl_3$) δ 207.6 (**C**^{13}), 161.3 (**C**^{7}), 150.8 (**C**^{1}H), 143.0 (**C**^{3}H), 136.5 (**C**^{5}), 130.4 (**C**^{9}H), 129.3 (**C**^{4}), 124.4 (**C**^{10}), 119.8 (**C**^{8}H), 101.0 (**C**^{6}), 55.7 (**C**^{15}H$_3$), 43.7 (**C**^{12}H$_2$), 30.3 (**C**^{14}H$_3$), 23.9 (**C**^{11}H$_2$).

IR ν 1772, 1716, 1624, 1270, 1163 cm^{-1}.

HRMS (ESI$^+$) m/z calc. for $C_{14}H_{15}NO_2Na$ [M+Na]$^+$ 252.0995; found 252.0999, Δ = 1.57 ppm.

4-(7-methylisoquinolin-4-yl)butan-2-one (3l):

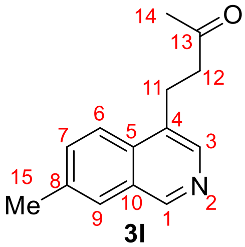

3l

Prepared according to **General Procedure C**, using 7-methylisoquinoline (**SI-1l**) (35.8 mg, 0.250 mmol) and methyl vinyl ketone (82 µL, 1.0 mmol) and was purified by flash column chromatography on SiO_2 (20:1 Et_2O/acetone) to afford **3l** as a white solid (47.0 mg, 88%).

Data for **3l**:

^{1}H NMR (600 MHz, $CDCl_3$) δ 9.03 (s, 1H, C^1**H**), 8.30 (s, 1H, C^3**H**), 7.84 (d, J = 8.6 Hz, 1H, C^6**H**), 7.73 (s, 1H, C^9**H**), 7.56 (dd, J = 8.6, 1.8 Hz, 1H, C^7**H**), 3.27 (t, J = 7.8 Hz, 2H, C^{11}**H**$_2$), 2.86 (t, J = 7.8 Hz, 2H, C^{12}**H**$_2$), 2.54 (s, 3H, C^{15}**H**$_3$), 2.16 (s, 3H, C^{14}**H**$_3$).

^{13}C NMR (151 MHz, $CDCl_3$) δ 207.4 (**C**^{13}), 151.1 (**C**^{1}H), 141.1 (**C**^{3}H), 137.0 (**C**^{8}), 132.82 (**C**^{7}H), 132.76 (**C**^{10}), 130.1 (**C**^{5}), 128.8 (**C**^{4}H), 127.3 (**C**^{9}H), 122.5 (**C**^{6}H), 44.2 (**C**^{12}H$_2$), 30.2 (**C**^{14}H$_3$), 23.8 (**C**^{11}H$_2$), 21.7 (**C**^{15}H$_3$).

IR: ν 3628, 1716, 1590, 1363, 1167 cm^{-1}.

HRMS (ESI^+) m/z calc. for $C_{14}H_{15}NONa$ $[M+Na]^+$ 236.1046; found 236.1052, Δ = 2.59 ppm.
Mp = 68-70 °C (amorphous).

4-(7-phenylisoquinolin-4-yl)butan-2-one (3m):

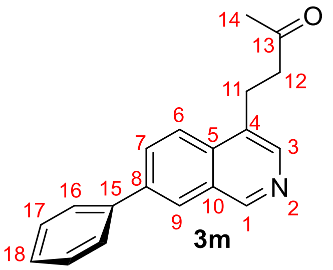

Prepared according to **General Procedure C**, using 7-phenylisoquinoline (**SI-1m**) (51.3 mg, 0.250 mmol) and methyl vinyl ketone (82 µL, 1.0 mmol) and was purified by flash column chromatography on SiO_2 (20:1 → 10:1 Et_2O/acetone) to afford **3m** as a white solid (40.2 mg, 58%).

Data for **3m**:

^{1}H NMR (600 MHz, $CDCl_3$) δ 9.19 (s, 1H, C^1**H**), 8.39 (s, 1H, C^3**H**), 8.16 (d, *J* = 1.4 Hz, 1H, C^9**H**), 8.04 (d, *J* = 8.7 Hz, 1H, C^6**H**), 8.00 (dd, *J* = 8.7, 1.9 Hz, 1H, C^7**H**), 7.72 (dd, *J* = 8.1, 1.1 Hz, 2H, 2×C^{16}**H**), 7.51 (t, *J* = 7.7 Hz, 2H, 2×C^{17}**H**), 7.42 (t, *J* = 7.4 Hz, 1H, C^{18}**H**), 3.33 (t, *J* = 7.8 Hz, 2H, C^{11}**H**$_2$), 2.92 (t, *J* = 7.8 Hz, 2H, $C^{12}H_2$), 2.20 (s, 3H, C^{14}**H**$_3$).
^{13}C NMR (151 MHz, $CDCl_3$) δ 207.3 (**C**^{13}), 152.0 (**C**^{1}H), 142.7 (**C**^{3}H), 140.1 (**C**$^{8/15}$), 140.0 (**C**$^{8/15}$), 133.6 (**C**^{10}), 130.24 (**C**^{7}), 130.19 (**C**$^{4/5}$), 129.2 (2×**C**^{17}H), 129.0 (**C**$^{4/5}$), 128.1 (**C**^{18}H), 127.5 (2×**C**^{16}H), 126.1 (**C**^{9}H), 123.4 (**C**^{6}H), 44.2 (**C**$^{12}H_2$), 30.2 (**C**$^{14}H_3$) **,** 23.8 (**C**$^{11}H_2$).
IR (neat) ν 3820, 1772, 1163 cm^{-1}.
HRMS m/z calc. for $C_{19}H_{17}NONa$ $[M+Na]^+$ 298.1202; found 298.1208, Δ = 1.88 ppm.
Mp = 96-97 °C (amorphous).

4-(7-bromoisoquinolin-4-yl)butan-2-one (3n):

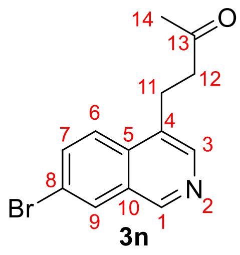

Prepared according to **General Procedure C**, using 7-bromoisoquinoline (52.0 mg, 0.250 mmol) and methyl vinyl ketone (82 µL, 1.0 mmol) and was purified by flash column chromatography on SiO_2 (20:1 Et_2O/acetone) to afford **3n** as a yellow oil which slowly solidified over time (50.5 mg, 73%).

Data for **3n**:

^{1}H NMR (600 MHz, $CDCl_3$) δ 9.03 (s, 1H, C^1**H**), 8.39 (s, 1H, C^3**H**), 8.11 (d, *J* = 2.1 Hz, 1H, C^9**H**), 7.83 (d, *J* = 8.9 Hz, 1H, C^7**H**), 7.77 (dd, *J* = 9.0, 2.0 Hz, 1H, C^6**H**), 3.27 (t, *J* = 7.7 Hz, 2H, C^{11}**H**$_2$), 2.86 (t, *J* = 7.7 Hz, 2H, C^{12}**H**$_2$), 2.16 (s, 3H, C^{14}**H**$_3$).

^{13}**C NMR** (151 MHz, $CDCl_3$) δ 207.0 (**C**^{13}), 150.6 (**C**^{1}H), 143.1 (**C**^{3}H), 134.0 (**C**^{6}H), 133.0 (**C**^{5}), 130.5 (**C**^{9}H), 130.4 (**C**^{4}), 129.5 (**C**^{8}), 124.7 (**C**^{7}H), 120.9 (**C**^{10}), 43.9 (**C**^{12}H_2), 30.2 (**C**^{14}H_3), 23.5 (**C**^{11}H_3).

IR (neat): ν 1716, 1579, 1494, 1429, 1361, 1211, 1164, 1077 cm^{-1}.

HRMS: (ESI^+) m/z calc. for $C_{13}H_{13}BrNO$ $[M+H]^+$ 278.0175; found 278.0183, Δ = 2.86 ppm.

Mp = 82-84 °C (amorphous).

4-(8-methylisoquinolin-4-yl)butan-2-one (3o):

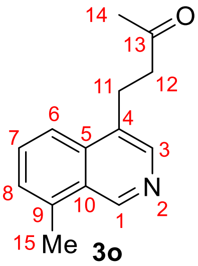

Prepared according to **General Procedure C**, using 8-methylisoquinoline (**SI-1o**) (35.8 mg, 0.250 mmol) and methyl vinyl ketone (82 μL, 1.0 mmol) and was purified by flash column chromatography on SiO_2 (20:1 Et_2O/acetone) to afford **3o** as a lightly coloured oil (43.2 mg, 81%).

Data for **3o**:

^{1}**H NMR** (600 MHz, $CDCl_3$) δ 9.34 (s, 1H, C^{1}**H**), 8.39 (s, 1H, C^{3}**H**), 7.79 (d, *J* = 8.5 Hz, 1H, C^{6}**H**), 7.59 (dd, *J* = 8.5, 7.0 Hz, 1H, C^{7}**H**), 7.38 (d, *J* = 7.0 Hz, 1H, C^{8}**H**), 3.29 (t, *J* = 7.7 Hz, 2H, C^{11}**H**$_2$), 2.87 (t, *J* = 7.9 Hz, 2H, C^{12}**H**$_2$), 2.78 (s, 3H, C^{15}**H**$_3$), 2.16 (s, 3H, C^{14}**H**$_3$).

^{13}**C NMR** (151 MHz, $CDCl_3$) δ 207.3 (**C**^{13}), 148.5 (**C**^{1}H), 142.6 (**C**^{3}H), 136.3 (**C**^{9}), 134.8 (**C**^{5}), 130.4 (**C**^{4}), 130.3 (**C**^{7}H), 128.0 (**C**^{8}H), 127.5 (**C**^{10}), 120.9 (**C**^{6}H), 44.2 (**C**^{12}H_2), 30.2 (**C**^{14}H_3), 24.0 (**C**^{11}H_2), 19.0 (**C**^{15}H_3).

IR (neat) ν 3628, 1715, 1415, 1366, 1163 cm^{-1}.

HRMS (ESI^+) m/z calc. for $C_{14}H_{15}NONa$ $[M+Na]^+$ 236.1046; found 236.1048, Δ = 0.89 ppm.

Mp = 59-60 °C (amorphous).

4-(8-phenylisoquinolin-4-yl)butan-2-one (3p):

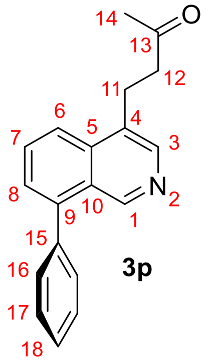

3p

Prepared according to **General Procedure C**, using 8-phenylisoquinoline (**SI-1p**) (51.3 mg, 0.250 mmol) and methyl vinyl ketone (82 µL, 1.0 mmol) and was purified by flash column chromatography on SiO_2 (10:1 Et_2O/acetone) to afford **3o** as a lightly coloured oil (54.9 mg, 80%).

Data for **3p**:

^{1}H NMR (500 MHz, $CDCl_3$) δ 9.17 (s, 1H, C^{1}**H**), 8.40 (s, 1H, C^{3}**H**), 7.97 (dt, *J* = 8.5, 1.1 Hz, 1H, C^{6}**H**), 7.77 (dd, *J* = 8.5, 7.1 Hz, 1H, C^{7}**H**), 7.57 – 7.41 (m, 6H, 6×C$^{8,16\text{-}18}$**H**), 3.35 (dd, *J* = 8.5, 7.1 Hz, 2H, C^{11}**H**$_2$), 2.93 (dd, *J* = 8.6, 7.0 Hz, 2H, C^{12}**H**), 2.20 (s, 3H, C^{14}**H**$_3$).

^{13}C NMR (126 MHz, $CDCl_3$) δ 207.3 (**C**^{13}), 150.4 (**C**^{1}H), 142.6 (**C**^{3}H), 142.0 (**C**^{9}), 139.0 (**C**^{15}), 134.9 (**C**^{5}), 130.2 (2×**C**^{16}H), 130.04 (**C**^{4}), 130.01 (**C**^{7}H), 128.6 (2×**C**^{17}H), 128.1 (**C**^{18}H), 128.0 (**C**^{8}H), 126.6 (**C**^{10}), 122.0 (**C**^{6}), 44.2 (**C**^{12}H$_2$), 30.2 (**C**^{14}H$_3$), 24.0 (**C**^{11}H$_2$).

IR (neat) ν 1715, 1623, 1488, 1362 cm^{-1}.

HRMS m/z calc. for $C_{19}H_{17}NONa$ $[M+Na]^+$ 298.1202; found 298.1216, Δ = 4.56 ppm.

Mp = 109-110 °C (amorphous).

4-(8-bromoisoquinolin-4-yl)butan-2-one (3q):

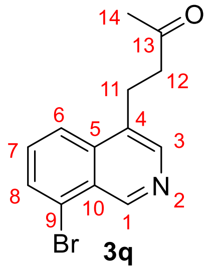

3q

Prepared according to **General Procedure C**, using 8-bromoisoquinoline (52.0 mg, 0.250 mmol) and methyl vinyl ketone (82 µL, 1.0 mmol) and was purified by flash column chromatography on SiO_2 (20:1 Et_2O/acetone) to afford **3q** as a pale yellow solid (63.7 mg, 92%).

Data for **3q**:

^{1}H NMR (600 MHz, $CDCl_3$) δ 9.52 (s, 1H, C^{1}**H**), 8.47 (s, 1H, C^{3}**H**), 7.94 (d, *J* = 8.5 Hz, 1H, C^{8}**H**), 7.85 (d, *J* = 7.4 Hz, 1H, C^{6}**H**), 7.56 (t, *J* = 8.2 Hz, 1H, C^{7}**H**), 3.31 (t, *J* = 7.7 Hz, 2H, C^{11}**H**$_2$), 2.88 (t, *J* = 7.7 Hz, 2H, C^{12}**H**$_2$), 2.18 (s, 3H, C^{14}**H**$_3$).

^{13}C NMR (150 MHz, $CDCl_3$) δ 207.0 (**C**^{13}), 151.2 (**C**^{1}H), 143.7 (**C**^{3}H), 136.2 (**C**^{5}), 131.3 (**C**^{6}H), 130.9 (**C**^{7}H), 130.0 (**C**^{4}), 126.8 (**C**^{10}), 123.7 (**C**^{9}), 122.6 (**C**^{8}), 44.1 (**C**^{14}H$_3$), 30.2 (**C**^{12}H$_2$), 23.8 (**C**^{11}H$_2$).

IR (neat) ν 2981, 1702, 1394, 1219, 1171, 965.

HRMS (ESI^+) m/z calc. for $C_{13}H_{14}BrNO$ $[M+H]^+$ 278.0175; found 278.0185, Δ = 3.58 ppm.

Mp = 80-82 °C (amorphous).

4-(8-chloroisoquinolin-4-yl)butan-2-one (3r):

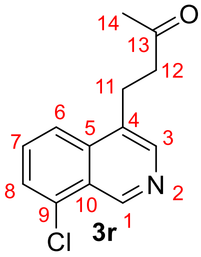

3r

Prepared according to **General Procedure C**, using 8-chloroisoquinoline (40.9 mg, 0.250 mmol) and methyl vinyl ketone (82 µL, 1.0 mmol) and was purified by flash column chromatography on SiO_2 (20:1 Et_2O/acetone) to afford **3r** as a yellow oil (46.5 mg, 80%).

Data for **3r**:

^{1}H NMR (600 MHz, $CDCl_3$) δ 9.57 (s, 1H, C^1**H**), 8.46 (s, 1H, C^3**H**), 7.91 – 7.86 (m, 1H, C^6**H**), 7.65 – 7.60 (m, 2H, 2×$C^{7/8}$**H**), 3.30 (t, J = 7.7 Hz, 2H, C^{11}**H**$_2$), 2.88 (t, J = 7.7 Hz, 2H, C^{12}**H**$_2$), 2.18 (s, 3H, C^{14}**H**$_2$).

^{13}C NMR (151 MHz, $CDCl_3$) δ 207.0, (**C**^{13}), 148.6 (**C**^{1}**H)**, 143.7 (**C**^{3}), 136.0 (**C**^{5}), 133.5 (**C**^{9}), 130.5 (**C**$^{7/8}$H), 130.1 (**C**^{4}), 127.5 (**C**$^{7/8}$H), 125.7 (**C**^{10}), 121.9 (**C**^{6}H), 44.0 (**C**^{12}H$_2$), 30.2 (**C**^{14}H$_3$), 23.9 (**C**^{11}H$_2$).

IR (neat) ν 1705, 1612, 1555, 1119, 1093 cm^{-1}.

HRMS (ESI$^+$) m/z calc. for $C_{13}H_{13}ClNO$ $[M+H]^+$ 234.0680; found 234.0683, Δ = 1.19 ppm.

Mp = 64-66 °C (amorphous).

1-(isoquinolin-4-yl)pentan-3-one[13] (5a):

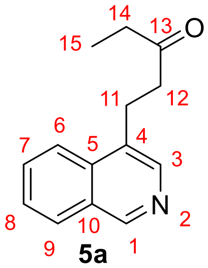

5a

Prepared according to a scaled-up version of **General Procedure C**, using isoquinoline (1.29 g, 10.0 mmol, 1.0 equiv.) and ethyl vinyl ketone (3.95 mL, 40.0 mmol, 4.0 equiv.). The crude reaction mixture was purified by flash column chromatography on SiO_2 (20:1 Et_2O/acetone) to afford **5a** as a pale yellow oil (1.20 g, 56%).

Data for **5a**:

^{1}H NMR (400 MHz, $CDCl_3$) δ 9.12 (s, 1H, C^1**H**), 8.37 (s, 1H, C^3**H**), 8.01 – 7.90 (m, 2H, 2×$C^{6,9}$**H**), 7.73 (ddd, J = 8.4, 6.9, 1.3 Hz, 1H, C^7**H**), 7.60 (ddd, J = 8.0, 6.9, 1.0 Hz, 1H, C^8**H**), 3.31 (t, J = 7.5 Hz, 3H, C^{11}**H**$_2$), 2.85 (t, J = 8.2 Hz, 2H, C^{12}**H**$_2$), 2.43 (q, J = 7.3 Hz, 2H, C^{14}**H**$_3$), 1.06 (t, J = 7.3 Hz, 3H, C^{15}**H**$_3$).

^{13}C NMR (100 MHz, $CDCl_3$) δ 210.1 (**C**^{13}), 151.7 (**C**^{1}H), 142.7 (**C**^{3}H), 134.5 (**C**^{5}), 130.6 (**C**^{7}H), 130.4 (**C**^{4}), 128.5 (**C**^{9}H+C^{10}), 127.1 (**C**^{8}H), 122.7 (**C**^{6}H), 42.8 (**C**^{12}H$_2$), 36.3 (**C**^{14}H$_2$), 23.9 (**C**^{11}H$_2$), 7.9 (**C**^{15}H$_3$).

IR (neat) ν 1713, 1623, 1584, 1504, 1376, 1229, 1149, 1113, 976 cm^{-1}.

HRMS (ESI$^+$) m/z calc. for $C_{14}H_{15}NONa$ [M+Na]$^+$ 236.1046; found 236.1051, Δ = 2.16 ppm.

1-(8-bromoisoquinolin-4-yl)pentan-3-one (5b):

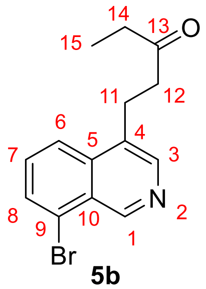

5b

Prepared according to a scaled-up version of **General Procedure C**, using 8-bromoisoquinoline (2.08 g, 10.0 mmol) and ethyl vinyl ketone (3.95 mL, 40.0 mmol) and was purified by flash column chromatography on SiO_2 (20:1 Et_2O/acetone) to afford **5b** as a yellow solid (2.41 g, 83%).

Data for **5b**:

^{1}H NMR (600 MHz, $CDCl_3$) δ 9.50 (s, 1H, C^1**H**), 8.45 (s, 1H, C^3**H**), 7.93 (d, *J* = 8.5 Hz, 1H, C^6**H**), 7.83 (dd, *J* = 7.5, 1.0 Hz, 1H, C^8**H**), 7.54 (dd, *J* = 8.5, 7.4 Hz, 1H, C^7**H**), 3.31 (t, *J* = 7.8 Hz, 2H, C^{11}**H**$_2$), 2.83 (t, *J* = 7.7 Hz, 2H, C^{12}**H**$_2$), 2.43 (q, *J* = 7.3 Hz, 2H, C^{14}**H**$_2$), 1.06 (t, *J* = 7.3 Hz, 3H, C^{15}**H**$_3$).

^{13}C NMR (151 MHz, $CDCl_3$) δ 209.8 (**C**^{13}), 151.2 (**C**^{1}H), 143.7 (**C**^{3}H), 136.1 (**C**^{5}), 131.3 (**C**^{8}H), 130.8 (**C**^{7}H), 130.1 (**C**^{4}), 126.7 (**C**^{10}), 123.6 (**C**^{9}), 122.6 (**C**^{6}H), 42.7 (**C**^{12}H$_2$), 36.3 (**C**^{14}H$_2$), 23.9 (**C**^{11}H$_2$), 7.9 (**C**^{15}H$_3$).

IR (neat) ν 1705, 1609, 1549, 1493, 1462, 1391, 1377, 1296, 1217, 1112, 1091 cm^{-1}.

HRMS (ESI$^+$) m/z calc. for $C_{14}H_{16}BrNO$ [M+H]$^+$ 292.0332; found 292.0340, Δ = 2.89 ppm.

Mp = 63-64 °C (amorphous).

1-(6-methylisoquinolin-4-yl)pentan-3-one (5c):

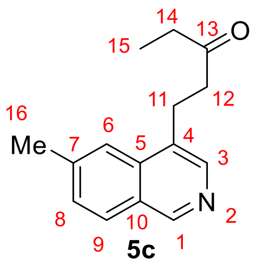

5c

Prepared according to a scaled-up version of **General Procedure C**, using 6-methylisoquinoline (1.43 g, 10.0 mmol) and ethyl vinyl ketone (3.95 mL, 40.0 mmol). The crude reaction mixture was purified by flash column chromatography on SiO_2 (20:1 Et_2O/acetone) to afford **5c** as a yellow oil (1.42 g, 63%).

Data for **5c**:

^{1}H NMR (400 MHz, $CDCl_3$) δ 9.05 (s, 1H, C^{1}**H**), 8.32 (s, 1H, C^{3}**H**), 7.87 (d, *J* = 8.3 Hz, 1H, C^{9}**H**), 7.71 (s, 1H, C^{6}**H**), 7.44 (dd, *J* = 8.4, 1.5 Hz, 1H, C^{8}**H**), 3.28 (t, *J* = 7.5 Hz, 2H, C^{11}**H**$_2$), 2.85 (t, *J* = 8.1 Hz, 2H, C^{12}**H**$_2$), 2.58 (s, 3H, C^{16}**H**$_3$), 2.45 (q, *J* = 7.3 Hz, 2H, C^{14}**H**$_2$), 1.07 (t, *J* = 7.3 Hz, 3H, C^{15}**H**$_3$).

^{13}C NMR (101 MHz, $CDCl_3$) δ 210.3 (**C**^{13}), 151.2 (**C**^{1}H), 142.5 (**C**^{3}H), 141.1 (**C**^{7}), 134.8 (**C**^{5}), 129.9 (**C**^{4}), 129.4 (**C**^{8}H), 128.4 (**C**^{9}H), 126.9 (**C**^{10}), 121.7 (**C**^{6}H), 42.8 (**C**^{12}H_2), 36.3 (**C**^{14}H_2), 23.9 (**C**^{11}H_2), 22.6 (**C**^{16}H_3), 7.9 (**C**^{15}H_3).

IR (neat) ν 2980, 1710, 1632, 1585, 1498, 1456, 1407, 1373, 1286, 1156 cm^{-1}.

HRMS (ESI^{+}) m/z calc. for $C_{15}H_{17}NONa$ $[M+Na]^{+}$ 250.1202; found 250.1205, Δ = 1.04 ppm.

1-(isoquinolin-4-yl)-4-methylpentan-3-one (6):

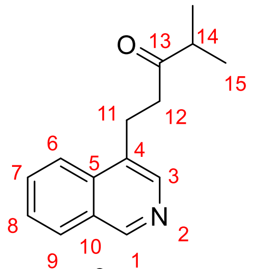

6

Prepared according to **General Procedure C**, using isoquinoline (32.3 mg, 0.250 mmol, 1.0 equiv.) and *iso*-propyl vinyl ketone (**SI-2**) (98.2 mg, 1.00 mmol, 4.0 equiv.) and was purified by flash column chromatography on SiO_2 (20:1 Et_2O/acetone) to afford **6** as a pale yellow oil (35.5 mg, 62%).

Data for **6**:

^{1}H NMR (400 MHz, $CDCl_3$) δ 9.11 (s, 1H, C^{1}**H**), 8.36 (s, 1H, C^{3}**H**), 8.00 – 7.92 (m, 2H, 2×$C^{6,9}$**H**), 7.73 (ddd, *J* = 8.5, 6.9, 1.3 Hz, 1H, C^{7}**H**), 7.60 (ddd, *J* = 8.1, 6.9, 1.1 Hz, 1H, C^{8}**H**), 3.29 (t, *J* = 7.4 Hz, 2H, C^{11}**H**$_2$), 2.88 (t, *J* = 8.1 Hz, 2H, C^{12}**H**$_2$), 2.57 (hept, *J* = 6.9 Hz, 1H, C^{14}**H**), 1.07 (d, *J* = 6.9 Hz, 6H, 2×C^{15}**H**$_3$).

^{13}C NMR (101 MHz, $CDCl_3$) δ 213.4 (**C**^{13}), 151.7 (**C**^{1}H), 142.7 (**C**^{3}H), 134.6 (**C**^{5}), 130.6 (**C**^{7}H), 130.5 (**C**^{4}), 128.6 (**C**^{9}H), 127.8 (**C**^{10}), 127.1 (**C**^{8}H), 122.7 (**C**^{6}H), 41.2 (**C**^{14}H), 40.9 (**C**^{12}H_2), 24.0 (**C**^{11}H_2), 18.3 (2×**C**^{15}H_3).

IR (neat) ν 3628, 1709, 1624, 1541, 1506, 1478, 1387, 1282 cm^{-1}.

HRMS (ESI^{+}) m/z calc. for $C_{15}H_{17}NONa$ $[M+Na]^{+}$ 250.1202; found 250.1204, Δ = 0.64 ppm.

4-(isoquinolin-4-yl)-1-phenylbutan-2-one (7):

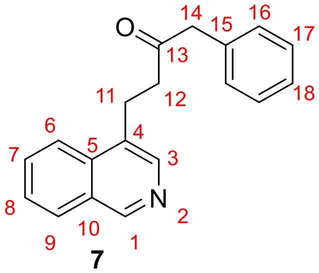

7

Prepared according to **General Procedure C**, using isoquinoline (32.3 mg, 0.250 mmol, 1.0 equiv.) and benzyl vinyl ketone (**SI-3**) (146 mg, 1.00 mmol, 4.0 equiv.) and was purified by flash column chromatography on SiO_2 (15:5:2 pentane/EtOAc/acetone) to afford **7** as an orange oil (50.3 mg, 73%).

Similarly, a scaled-up version of this reaction was performed using isoquinoline (258 mg, 2.00 mmol) and reduced amount of **SI-3** (584 mg, 4.00 mmol, 2.0 equiv.) and was flash column chromatography on SiO_2 (15:5:2 pentane/EtOAc/acetone) to afford **7** as an orange oil (311 mg, 56%).

Data for **7**:

^{1}H NMR (600 MHz, $CDCl_3$) δ 9.11 (s, 1H, C^{1}**H**), 8.32 (s, 1H, C^{3}**H**), 7.96 (dd, J = 8.1, 1.0 Hz, 1H, C^{9}**H**), 7.86 – 7.81 (m, 1H, C^{6}**H**), 7.68 (ddd, J = 8.3, 6.8, 1.3 Hz, 1H, C^{7}**H**), 7.62 – 7.54 (m, 1H, C^{8}**H**), 7.29 (tt, J = 7.0, 1.7 Hz, 2H, 2×C^{17}**H**), 7.24 (tt, J = 7.3, 2.0 Hz, 1H, C^{18}H), 7.16 (d, J = 7.4 Hz, 2H, 2×C^{16}**H**), 3.69 (s, 2H, C^{14}**H**), 3.26 (t, J = 7.6 Hz, 2H, C^{11}**H$_2$**), 2.88 (t, J = 8.1 Hz, 2H, C^{12}**H$_2$**).

^{13}C NMR (151 MHz, $CDCl_3$) δ 207.0 (**C**^{13}), 151.7 (**C**^{1}H), 142.7 (**C**^{3}H), 134.4 (**C**^{5}), 133.9 (**C**^{15}), 130.5 (**C**^{7}H), 130.1 (**C**^{4}), 129.5 (2×**C**^{16}H), 128.9 (2×**C**^{17}H), 128.5 (**C**^{9}H + **C**^{10}), 127.2 (**C**^{18}H), 127.0 (**C**^{8}H), 122.6 (**C**^{6}H), 50.6 (**C**^{14}H$_2$), 42.3 (**C**^{12}H$_2$), 24.0 (**C**^{11}H$_2$).

IR (neat) ν 3628, 1716, 1497, 786, 751, 699 cm^{-1}.

HRMS (ESI$^+$) m/z calc. for $C_{19}H_{17}NONa$ [M+Na]$^+$ 298.1202; found 298.1197, Δ = –1.81 ppm.

3-(isoquinolin-4-yl)-1-phenylpropan-1-one (8):

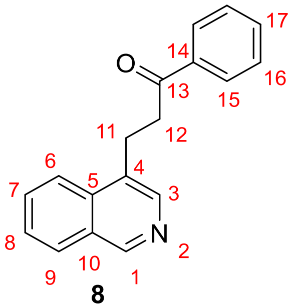

8

The title compound was prepared using a modified version of **General Procedure C** with isoquinoline (32.3 mg, 0.250 mmol. 1.0 equiv.) and phenyl vinyl ketone (**SI-4**) (132 mg, 1.00 mmol, 4.0 equiv.) and heating to 100 °C for 18 h. Purification by flash column chromatography on SiO_2 (40:1 → 20:1 Et_2O/acetone) afforded **8** as an off-white solid (16.2 mg, 25%).

N.B. Purification of this compound was made difficult due to large amounts of remaining isoquinoline (65% by crude qNMR, 47% isolated). The yield reported is after two sequential flash columns.

Data for **8**:

^{1}H NMR (600 MHz, $CDCl_3$) δ 9.15 (s, 1H, C^1**H**), 8.46 (s, 1H, C^3**H**), 8.03 (d, *J* = 8.5 Hz, 1H, C^6**H**), 8.00 (d, *J* = 8.2 Hz, 1H, C^9**H**), 7.98 – 7.93 (m, 2H, 2×C^{15}**H**), 7.75 (ddd, *J* = 8.3, 6.8, 1.3 Hz, 1H, C^7**H**), 7.62 (ddd, *J* = 8.1, 6.8, 1.1 Hz, 1H, C^8**H**), 7.59 – 7.53 (m, C^{17}**H**), 7.48 – 7.42 (m, 2H, 2×C^{16}**H**), 3.54 – 3.46 (m, 2H, C^{11}**H**$_2$), 3.46 – 3.40 (m, 2H, C^{12}**H**$_2$).
^{13}C NMR (151 MHz, $CDCl_3$) δ 198.8 (**C**^{13}), 151.8 (**C**^{1}H), 142.9 (**C**^{3}H), 136.8 (**C**^{14}), 134.6 (**C**^{5}), 133.4 (**C**^{17}H), 130.7 (**C**^{7}H), 130.5 (**C**^{4}), 128.8 (2×**C**^{16}H), 128.6 (**C**^{9}H and **C**^{10}), 128.2 (2×**C**^{15}H), 127.1 (**C**^{8}H), 122.8 (**C**^{6}H), 39.4 (**C**^{12}H$_2$), 24.3 (**C**^{11}H$_2$).
IR (neat) ν 2917, 1684, 1623, 1597, 1503, 1449, 1364, 1296, 1226, 1205, 976, 887, 786, 743, 690, 664, 650, 647, 638, 626, 606, 601 cm^{-1}.
HRMS (ESI$^+$) m/z calc. for $C_{18}H_{16}NO$ $[M+H]^+$ 262.1226; found 262.1222, Δ = –1.53 ppm.
M.p. = 101-102 °C (recrystallised from acetone).

3-(isoquinolin-4-yl)-1-(4-(trifluoromethyl)phenyl)propan-1-one (9):

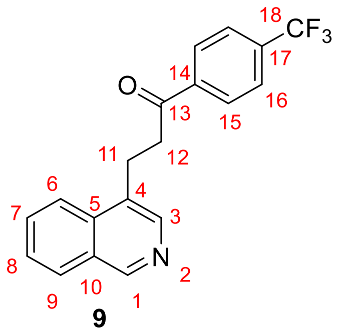

9

The title compound was prepared using a modified version of **General Procedure C** with isoquinoline (32.3 mg, 0.250 mmol, 1.0 equiv.) and 1-(4-(trifluoromethyl)phenyl)prop-2-en-1-one (**SI-5**) (200 mg, 1.0 mmol, 4.0 equiv.) and heating to 100 °C. Purification by flash column chromatography on SiO_2 (1% acetone in Et_2O) afforded **9** as a pale yellow solid (40 mg, 48%).

Data for **9**:

^{1}H NMR (600 MHz, $CDCl_3$) δ 9.14 (s, 1H, C^1**H**), 8.45 (s, 1H, C^3**H**), 8.03 (d, *J* = 8.0 Hz, 1H, 2×C^{15}**H**), 8.01 – 7.97 (m, 2H, 2×C^{6,9}**H**), 7.74 (ddd, *J* = 8.4, 6.9, 1.4 Hz, 1H, C^7**H**), 7.71 – 7.66 (m, 2H, 2×C^{16}**H**), 7.61 (ddd, *J* = 7.9, 6.8, 1.1 Hz, 1H, C^8**H**), 3.49 (ddd, *J* = 8.5, 6.5, 1.7 Hz, 2H, C^{11}**H**$_2$), 3.43 (ddd, *J* = 8.4, 6.7, 1.7 Hz, 2H, C^{12}**H**$_2$).
^{13}C NMR (151 MHz, $CDCl_3$) δ 197.8 (**C**^{13}), 151.9 (**C**^{1}H), 142.9 (**C**^{3}H), 139.3 (**C**^{14}), 134.7 (q, *J* = 33.4 Hz, C^{17}), 134.5 (C^5), 130.8 (**C**^{7}H), 130.1 (**C**^{4}), 128.6 (**C**^{9}H), 128.6 (**C**^{10}), 128.5

(2×**C**[15]H), 127.2 (**C**[8]H), 125.8 (q, J = 3.7 Hz, 2×**C**[16]H), 123.5 (q, J = 272.5 Hz, **C**[18]F_3), 122.6 (**C**[6]H), 39.6 (**C**[12]H_2), 24.0 (**C**[11]H_2).

^{19}F NMR (377 MHz, $CDCl_3$) δ –63.1 C[18]**F_3**).

IR (neat) ν 1694, 1412, 1331, 1202, 1119, 1107, 1070, 988, 844, 772, 750, 731 cm^{-1}.

HRMS (ESI^+) m/z calc. for $C_{19}H_{15}F_3NO$ $[M+H]^+$ 330.1100; found 330.1099, Δ = –0.40 ppm.

M.p. = 136-138 °C (recrystallised from acetone).

1-(furan-2-yl)-3-(isoquinolin-4-yl)propan-1-one (10):

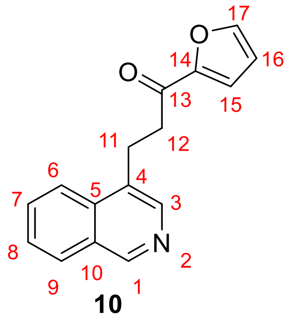

10

The title compound was prepared using a modified version of **General Procedure C** with isoquinoline (32.3 mg, 0.250 mmol, 1.0 equiv.) and 1-(furan-2-yl)prop-2-en-1-one (**SI-6**) (120 mg, 1.00 mmol, 4.0 equiv.) and hearting to 100 °C. Purification by flash column chromatography on SiO_2 (1% acetone in Et_2O) afforded **10** as an orange solid (42 mg, 68%).

Data for **10**:

^{1}H NMR (500 MHz, $CDCl_3$) δ 9.11 (s, 1H, C[1]**H**), 8.42 (s, 1H, C[3]**H**), 8.01 (d, J = 8.5 Hz, 1H, C[6]**H**), 7.96 (d, J = 8.2 Hz, 1H, C[9]**H**), 7.72 (ddd, J = 8.4, 6.9, 1.3 Hz, 1H, C[7]**H**), 7.59 (ddd, J = 8.0, 6.8, 1.1 Hz, 1H, C[8]**H**), 7.54 (d, J = 1.7 Hz, 1H, C[17]**H**), 7.15 (d, J = 3.6 Hz, 1H, C[15]H), 6.49 (dd, J = 3.5, 1.7 Hz, 1H, C[16]H), 3.43 (dd, J = 8.9, 6.8 Hz, 2H, C[11]**H_2**), 3.25 (dd, J = 8.9, 6.8 Hz, 2H, C[12]**H_2**).

^{13}C NMR (126 MHz, $CDCl_3$) δ 188.0 (**C**[13]), 152.5 (**C**[14]), 151.7 (**C**^{1}H), 146.5 (**C**[17]H), 142.7 (**C**[3]H), 134.5 (**C**[5]), 130.6 (**C**[7]), 130.1 (**C**[4]), 128.5 (**C**[9]H), 128.5 (**C**[10]), 127.1 (**C**[8]H), 122.7 (**C**[6]H), 117.2 (**C**[15]H), 112.4 (**C**[16]H), 39.0 (**C**[12]H_2), 24.1 (**C**[11]H_2).

IR (neat) ν 1672, 1623, 1585, 1504, 1468, 1394, 1364, 1306, 1268, 1085, 1047, 1021 cm^{-1}.

HRMS (ESI^+) m/z calc. for $C_{16}H_{14}NO_2$ $[M+H]^+$ 252.1019; found 252.1019, Δ = 0.00 ppm.

M.p. = 71-72 °C (recrystallised from acetone).

2.5 *Derivatisation Reactions*

4-(isoquinolin-4-yl)-2-methylbutan-2-ol (11):

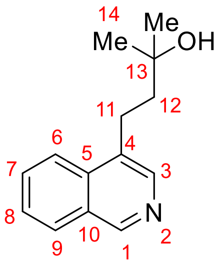

11

To a solution of **3a** (199 mg, 1.00 mmol) in dry THF (5 mL) was added MeMgBr (3.0 M in Et_2O, 0.40 mL, 1.2 mmol) dropwise at 0 °C. The mixture was stirred for 10 min, then warmed to room temperature and stirred for an additional 2.5 h. The reaction was quenched with saturated NH_4Cl (5 mL) and water (5 mL) was added. The mixture was then extracted with EtOAc (10 mL × 4), and the combined organic extracts were washed with water (20 mL) and brine (20 mL), dried over $MgSO_4$, filtered, and concentrated *in vacuo*. The residue was purified by flash column chromatography on SiO_2 (20:1 CH_2Cl_2/MeOH) to afford **11** as a colourless oil (175 mg, 81%).

Data for **11**:

^{1}H NMR (600 MHz, $CDCl_3$) δ 9.12 (s, 1H, C^1**H**), 8.40 (s, 1H, C^3**H**), 8.03 (d, *J* = 7.9 Hz, 1H, C^6**H**), 7.97 (d, *J* = 8.1 Hz, 1H, C^9**H**), 7.73 (ddd, *J* = 8.4, 6.9, 1.3 Hz, 1H, C^7**H**), 7.60 (ddd, *J* = 8.1, 6.8, 1.1 Hz, 1H, C^8**H**), 3.17 – 3.11 (m, 2H, C^{11}**H**$_2$), 1.92 – 1.86 (m, 2H, C^{12}**H**$_2$), 1.74 (br s, 1H, C^{13}**OH**), 1.38 (s, 6H, 2 × C^{14}**H**$_3$).

^{13}C NMR (151 MHz, $CDCl_3$) δ 151.4 (**C**^{1}H), 142.6 (**C**^{3}H), 134.8 (**C**^{5}), 131.9 (**C**^{4}), 130.4 (**C**^{7}H), 128.6 (**C**^{10}), 128.5 (**C**^{9}H), 127.0 (**C**^{8}H), 123.0 (**C**^{6}H), 71.0 (**C**^{13}OH), 44.8 (**C**^{12}H$_2$), 29.6 (2 × **C**^{14}H$_3$), 25.0 (**C**^{11}H$_2$).

IR ν 3365(broad), 2360, 2341, 1739 cm^{-1}.

HRMS (ESI$^+$) m/z calc. for $C_{14}H_{18}NO$ [M+H]$^+$ 216.1383; found 216.1391, Δ = 3.73 ppm.

4-(3-(benzyloxy)-3-oxopropyl)isoquinoline 2-oxide (SI-7):

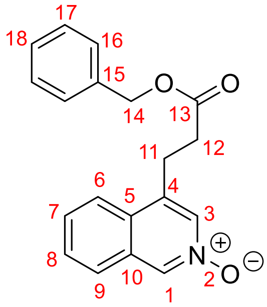

SI-7

In a GC vial, to a solution of **7** (34.4 mg, 0.250 mmol) in AcOH (0.2 mL) was added NaOAc (51 mg, 1.25 mmol) followed by peracetic acid (39% in AcOH, 0.30 mL, 2.5 mmol). The reaction mixture was heated to 80 °C for 3 h. The reaction was then cooled and dissolved in EtOAc (10 mL) and quenched with sat. Na_2CO_3 solution (15 mL). The layers were separated and the aqueous phase was extracted with EtOAc (10 mL × 4). The combined organic extracts were washed sequentially with

sat. $Na_2S_2O_3$ solution (10 mL), sat. Na_2CO_3 solution (10 mL × 2), water (10 mL) and brine (10 mL). The organic phase was then dried over $MgSO_4$, filtered, and concentrated *in vacuo*. The residue was purified by flash column chromatography on SiO_2 (10:1 CH_2Cl_2/MeOH) to afford **SI-7** as a clear colourless oil (23.0 mg, 63%).

Data for **SI-7**:

^{1}**H NMR** (600 MHz, $CDCl_3$) δ 8.67 (d, *J* = 1.7 Hz, 1H, C^{1}**H**), 8.05 (d, *J* = 1.7 Hz, 1H, C^{3}**H**), 7.92 – 7.88 (m, 1H, C^{6}**H**), 7.74 – 7.70 (m, 1H, C^{9}**H**), 7.63 – 7.59 (m, 2H, 2×C$^{7/8}$**H**), 7.36 – 7.30 (m, 5H, 5×C$^{16\text{-}18}$**H**), 5.13 (s, 2H, C^{14}**H**$_2$), 3.32 (t, *J* = 7.8 Hz, 2H, C^{11}**H**$_2$), 2.80 (t, *J* = 7.8 Hz, 2H, C^{12}**H**$_2$).
^{13}**C NMR** (151 MHz, $CDCl_3$) δ 171.7 (**C**^{13}), 135.8 (**C**^{3}H), 135.6 (**C**^{15}), 135.2 (**C**^{1}H), 134.8 (**C**^{4}), 129.8 (**C**^{10}), 129.5 (**C**$^{7/8}$H), 129.4 (**C**$^{7/8}$H), 128.7 (2×**C**$^{16/17}$H), 128.5 (**C**^{18}H), 128.5 (2×**C**$^{16/17}$H), 128.2 (**C**^{5}), 126.1 (**C**^{9}H), 123.0 (**C**^{6}H), 66.9 (**C**^{14}H$_2$), 33.9 (**C**^{12}H$_2$), 24.9 (**C**^{11}H$_2$).
HRMS (ESI^{+}) m/z calc. for $C_{19}H_{17}NO_3Na$ $[M+Na]^+$ 330.1101; found 330.1113, Δ = 3.73 ppm.
IR ν 1733, 1626, 1224, 1167, 752 cm^{-1}.

benzyl 3-(isoquinolin-4-yl)propanoate (12):

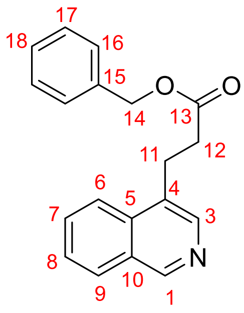

12

To a solution of *N*-oxide **12** (20 mg, 0.065 mmol) in MeCN (0.5 mL) was added B_2pin_2 (16.5 mg, 0.065 mmol). The reaction was stirred and heated to 70 °C for 20 h. Then, the mixture was taken up in CH_2Cl_2 (2 mL) and concentrated *in vacuo*. The residue was purified by flash column chromatography on SiO_2 (15:5:2 pentane/EtOAc/acetone) to afford **12** as a clear colourless oil (12.1 mg, 64%)

Data for **12**:

^{1}**H NMR** (400 MHz, $CDCl_3$) δ 9.15 (s, 1H, C^{1}**H**), 8.40 (s, 1H, C^{3}**H**), 8.02 – 7.95 (m, 2H, 2×C$^{6/9}$**H**), 7.74 (ddd, *J* = 8.5, 6.9, 1.3 Hz, 1H, C$^{7/8}$H), 7.62 (ddd, *J* = 7.9, 6.9, 1.1 Hz, 1H, C$^{7/8}$**H**), 7.39 – 7.29 (m, 5H, 5×C$^{16\text{-}18}$**H**), 5.14 (s, 2H, C^{14}**H**$_2$), 3.39 (dd, *J* = 8.7, 7.1 Hz, 2H, C^{11}**H**$_2$), 2.82 (t, *J* = 8.2 Hz, 2H, C^{12}**H**$_2$).
^{13}**C NMR** (101 MHz, $CDCl_3$) δ 172.5 (**C**^{13}), 152.0 (**C**^{1}H), 142.7 (**C**^{3}H), 135.8 (**C**$_q$), 134.5 (**C**$_q$), 130.7 (**C**$^{7/8}$H), 129.6 (**C**$_q$), 128.7 (2×**C**$^{16/17}$H), 128.6 (**C**$^{6/9}$H), 128.5 (**C**$_q$), 128.46 (**C**^{18}H), 128.43 (2×**C**$^{16/17}$H), 127.1 (**C**$^{7/8}$H), 122.6 (**C**$^{6/9}$H), 66.7 (**C**^{14}H$_2$), 35.0 (**C**^{12}H$_2$), 25.3 (**C**^{11}H$_2$).

IR (neat) ν 1685, 1623, 1503, 1449, 1364, 1296, 1225, 1205 cm^{-1}.

HRMS (ESI^{+}) m/z calc. for $C_{19}H_{17}NO_2$ [M+Na]$^{+}$ 314.1152; found 314.1156, Δ = 1.42 ppm.

4-(2-(2-methyloxiran-2-yl)ethyl)isoquinoline (13):

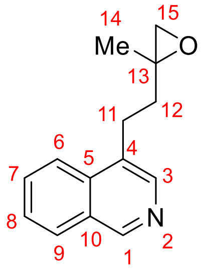

13

Trimethylsulfonium iodide (224 mg, 1.10 mmol, 1.1 equiv.) and potassium *tert*-butoxide (112 mg, 1.00 mmol, 1.0 equiv.) were stirred in DMSO (1.5 mL) at rt for 15 min. A solution of **3** (200 mg, 1.00 mmol, 1.0 equiv.) in DMSO (0.50 mL) was added to the reaction mixture, which was then stirred at 40 °C for 18 h. The reaction was quenched with sat. aq. NH_4Cl, diluted with CH_2Cl_2, shaken and the layers partitioned. The aqueous layer was extracted twice more with CH_2Cl_2, the combined organic layers were dried over $MgSO_4$, filtered under gravity and concentrated *in vacuo*. Purification by flash column chromatography (5% acetone in Et_2O) afforded **13** as a pale orange oil (150 mg, 70%).

Data for **13**:

^{1}H NMR (500 MHz, $CDCl_3$) δ 9.12 (1H, s, C^{1}**H**), 8.37 (1H, s, C^{3}**H**), 8.04 – 7.92 (2H, m, 2×C$^{6/9}$**H**), 7.72 (1H, ddt, *J* = 8.2, 6.8, 1.2 Hz, C^{7}**H**), 7.67 – 7.54 (1H, m, C^{8}**H)**, 3.16 – 3.05 (2H, m, C^{11}**H**$_2$), 2.66 (1H, d, *J* = 4.7 Hz, 1×C^{15}**H**$_2$), 2.64 (1H, d, *J* = 4.7 Hz, 1×C^{15}**H**$_2$), 2.04 – 1.93 (2H, m, C^{12}**H**$_2$), 1.45 (3H, s, C^{14}**H**$_2$).

^{13}C NMR (126 MHz, $CDCl_3$) δ 151.6 (**C**^{1}H), 142.5 (**C**^{3}H), 134.6 (**C**^{5}), 130.8 (**C**^{4}), 130.5 (**C**^{7}H), 128.6 (**C**^{10}), 128.5 (**C**^{9}H), 127.1 (**C**^{8}H), 122.8 (**C**^{6}H), 56.8 (**C**^{13}), 54.0 (**C**^{15}H$_2$), 37.6 (**C**^{12}H$_2$), 25.6 (**C**^{11}H$_2$), 21.2 (**C**^{14}H$_3$).

IR (neat) ν 1623, 1585, 1504, 1455, 1390, 1300, 1254, 1230, 1147, 1107, 1078, 1021 cm^{-1}.

HRMS (ESI^{+}) m/z calc. for $C_{14}H_{16}NO$ [M+H]$^{+}$ 214.1226; found 214.1225, Δ = –0.47 ppm.

4-butylisoquinoline (14):

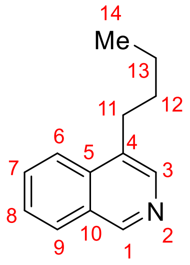

14

3a (50 mg, 0.25 mmol, 1.0 equiv.) was dissolved in ethylene glycol (0.5 mL) and added to a solution of potassium hydroxide (56 mg, 1.0 mmol, 4.0 equiv.) and hydrazine monohydrate (50 mg, 1.00 mmol, 4.0 equiv.) in ethylene glycol (0.5 mL). The solution was heated to 180 °C for 7 h then diluted with EtOAc and quenched with sat. aq. NH_4Cl. The suspension was shaken, the layers partitioned and the aqueous layer extracted twice more with EtOAc. The combined organic layers were dried over $MgSO_4$, filtered under gravity and concentrated *in vacuo*. Purification by flash column chromatography (90% Et_2O in pentane) afforded **14** as a dark orange oil (28 mg, 61%). Spectroscopic data were consistent with the literature data for this compound.[15]

Data for **14**:

^{1}H NMR (600 MHz, $CDCl_3$) δ 9.11 (s, 1H, C^1**H**), 8.37 (s, C^3**H**), 7.99 (d, *J* = 8.5 Hz, 1H, C^6**H**), 7.96 (dd, *J* = 8.3, 1.4 Hz, 1H, C^9**H**), 7.74 – 7.68 (m, 1H, C^7**H**), 7.61 – 7.55 (m, 1H, C^8**H**), 3.01 (t, *J* = 7.8 Hz, 2H, C^{11}**H**$_2$), 1.76 – 1.68 (m, 2H, C^{12}**H**$_2$), 1.50 – 1.41 (2H, m, C^{13}**H**$_2$), 0.97 (3H, t, *J* = 7.4 Hz, 3H, C^{14}**H**$_2$).

^{13}C NMR (151 MHz, $CDCl_3$) δ 151.2 (**C**^{1}H), 142.7 (**C**^{3}H), 134.8 (**C**^{5}), 131.9 (**C**^{4}), 130.2 (**C**^{7}H), 128.6 (**C**^{10}), 128.4 (**C**^{9}H), 126.8 (**C**^{8}H), 123.1 (**C**^{6}H), 32.9 (**C**^{12}H$_2$), 29.9 (**C**^{11}H$_2$), 22.9 (**C**^{13}H$_2$), 14.0 (**C**^{14}H$_3$).

IR (neat) ν 2957, 2931, 1701, 1685, 1653, 1623, 1583, 1569, 1540, 1503, 1465, 1457, 1389, 1379, 1266, 1249, 1230, 1166, 1148, 1106, 1020 cm^{-1}.

HRMS (ESI^+) m/z calc. for $C_{13}H_{16}N$ $[M+H]^+$ 186.1277; found 186.1277, Δ = 0.00 ppm.

_N_-benzyl-4-(isoquinolin-4-yl)butan-2-amine (15):

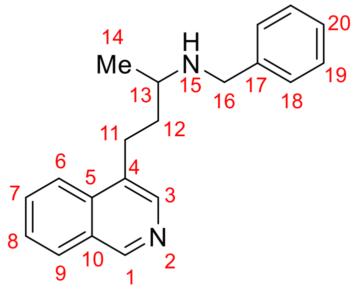

15

To a solution of **3a** (50 mg, 0.25 mmol, 1.0 equiv.) in MeOH (0.20 mL) was added benzylamine (30 µL, 0.28 mmol, 1.1 equiv.), $NaCNBH_3$ (20 mg, 0.33 mmol, 1.3 equiv.) and powdered 3Å molecular sieves. Acetic acid (29 µL, 0.50 mmol, 2.0 equiv.) was added dropwise to the stirred mixture at 0 °C, after which the mixture was stirred at rt for 3 h. The reaction mixture was quenched with sat. aq. Na_2CO_3 solution and extracted with EtOAc (×3), then the combined organic phases were dried over Na_2SO_4, filtered, and concentrated *in vacuo*. Purification by flash column chromatography (2 → 10% acetone in EtOAc) afford **15** as a pale orange oil (48 mg, 66%).

Data for **15**:

^{1}H NMR (500 MHz, $CDCl_3$) δ 9.04 (s, 1H, C^{1}**H**), 8.29 (s, 1H, C^{3}**H**), 7.93 (dt, *J* = 8.5, 1.0 Hz, 1H, C^{6}**H**), 7.89 (dp, *J* = 8.1, 1.0 Hz, 1H, C^{9}**H**), 7.63 (ddt, *J* = 8.4, 6.8, 1.4 Hz, 1H, C^{7}**H**), 7.51 (ddt, *J* = 8.2, 6.9, 1.3 Hz, 1H, C^{8}**H**), 7.28 – 7.22 (m, 5H, C^{8}**H**, C^{18}**H**, C^{19}**H**), 7.20 – 7.15 (m, 1H, C^{20}**H**), 3.79 (d, *J* = 13.0 Hz, 1H, 1×C^{16}**H**$_2$), 3.69 (d, *J* = 13.0 Hz, 1H, 1×C^{16}**H**$_2$), 3.00 (dd, *J* = 9.0, 7.2 Hz, 2H, C^{11}**H**$_2$), 2.76 (sext, *J* = 6.2 Hz, 1H, C^{13}**H**), 1.87 – 1.76 (m, 1H, 1×C^{12}**H**$_2$), 1.76 – 1.67 (m, 1H, 1×C^{12}**H**$_2$), 1.14 (dd, *J* = 6.3, 0.9 Hz, 3H, C^{14}**H**$_3$).
^{13}C NMR (126 MHz, $CDCl_3$) δ 151.3 (**C**^{1}H), 142.7 (**C**^{3}H), 140.8 (**C**^{17}), 134.7 (**C**^{5}), 131.8 (**C**^{4}), 130.3 (**C**^{7}H), 128.6 (**C**^{10}), 128.5 (2×**C**^{19}H), 128.4 (**C**^{9}H), 128.2 (2×**C**^{18}H), 127.0 (**C**^{20}H), 126.9 (**C**^{8}H), 123.0 (**C**^{6}H), 52.4 (**C**^{13}H), 51.5 (**C**^{16}H$_2$), 38.0 (**C**^{12}H$_2$), 26.5 (**C**^{11}H$_2$), 20.5 (**C**^{14}H$_3$).
IR (neat) ν 1653, 1623, 1505, 1455, 1376, 1148, 1027, 886, 787, 749 cm^{-1}.
HRMS (ESI^+) m/z calc. for $C_{20}H_{23}N_2$ $[M+H]^+$ 291.1856; found 291.1862, Δ = 2.18 ppm.

3. *Screening and Optimisation*

Figure SI-1: List of Electrophiles Screened for the C-4 alkylation of Isoquinoline

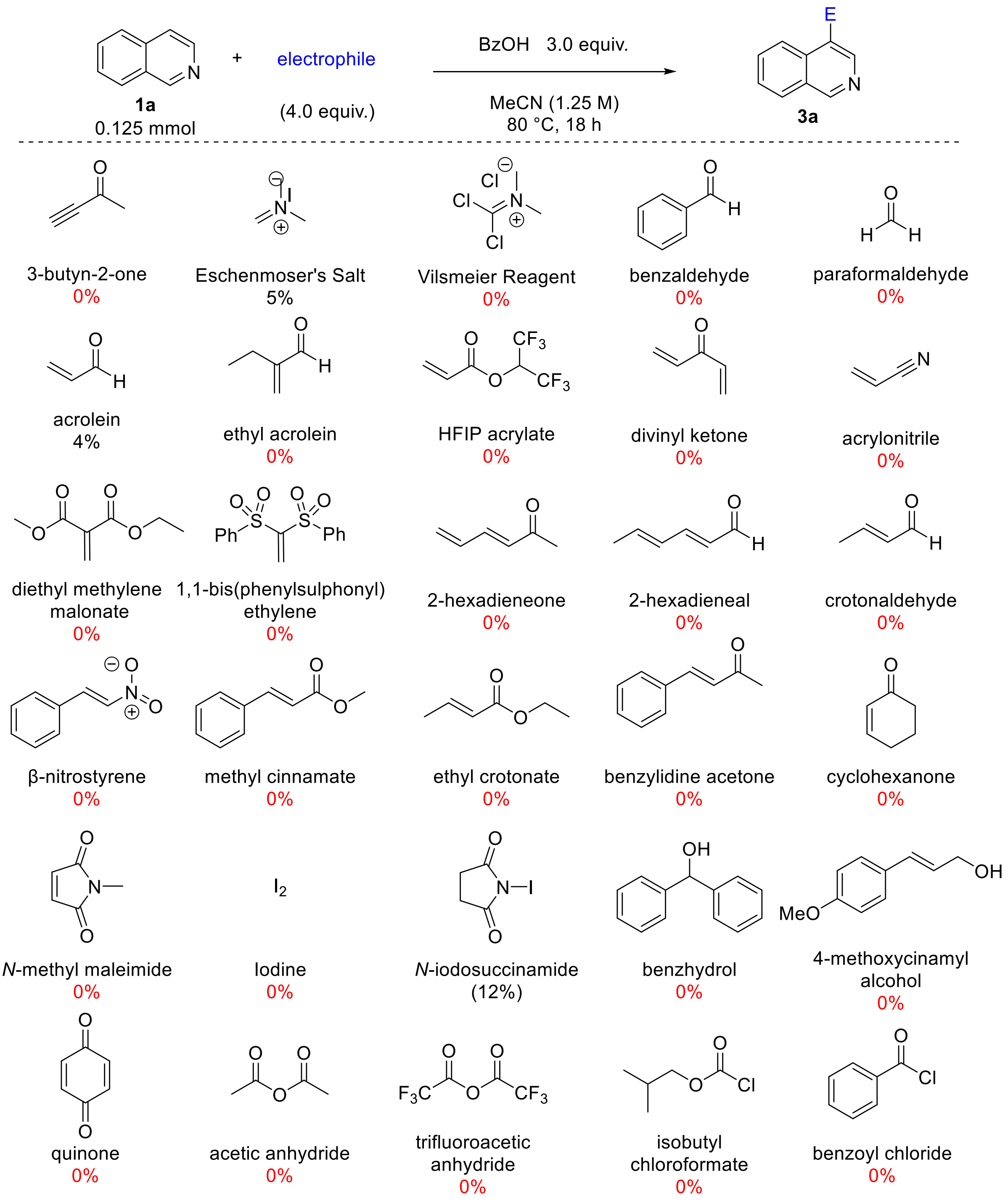

Reactions were performed under the conditions for **General Procedure C** on either a 0.125 or 0.250 mmol scale. qNMR yields were determined using trimethoxybenzene as an internal standard. Yields in parentheses were isolated yields. *Reaction was performed at 100 °C.

Figure SI-2: List of Electrophiles Screened for the C-4 alkylation of Isoquinoline, Continued

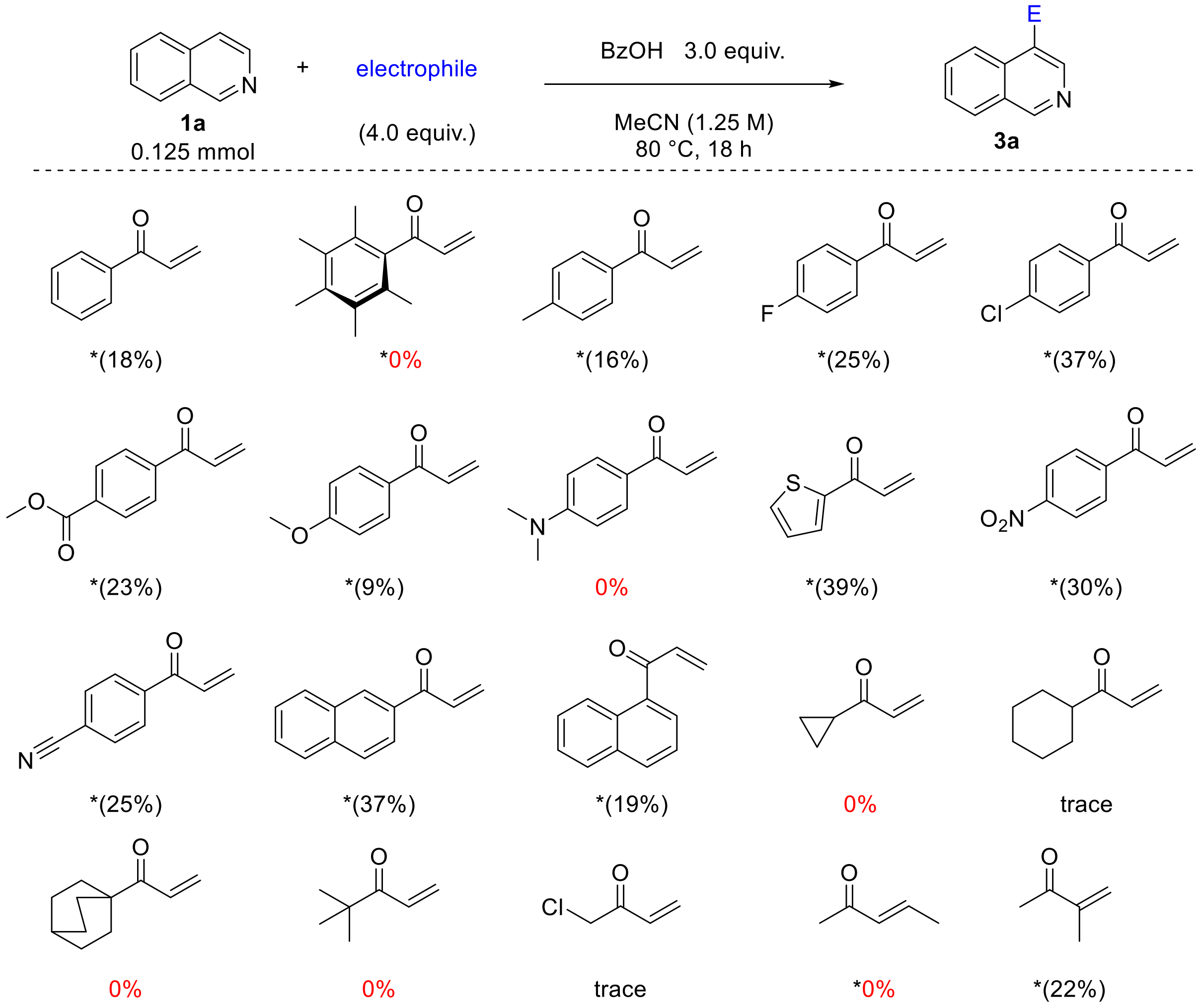

Reactions were performed under the conditions for **General Procedure C** on either a 0.125 or 0.250 mmol scale. qNMR yields were determined using trimethoxybenzene as an internal standard. Yields in parentheses were isolated yields. *Reaction was performed at 100 °C.

Figure SI-3: List of Carboxylic Acids/Nucleophiles Screened for the C-4 alkylation of Isoquinoline with Methyl Vinyl Ketone

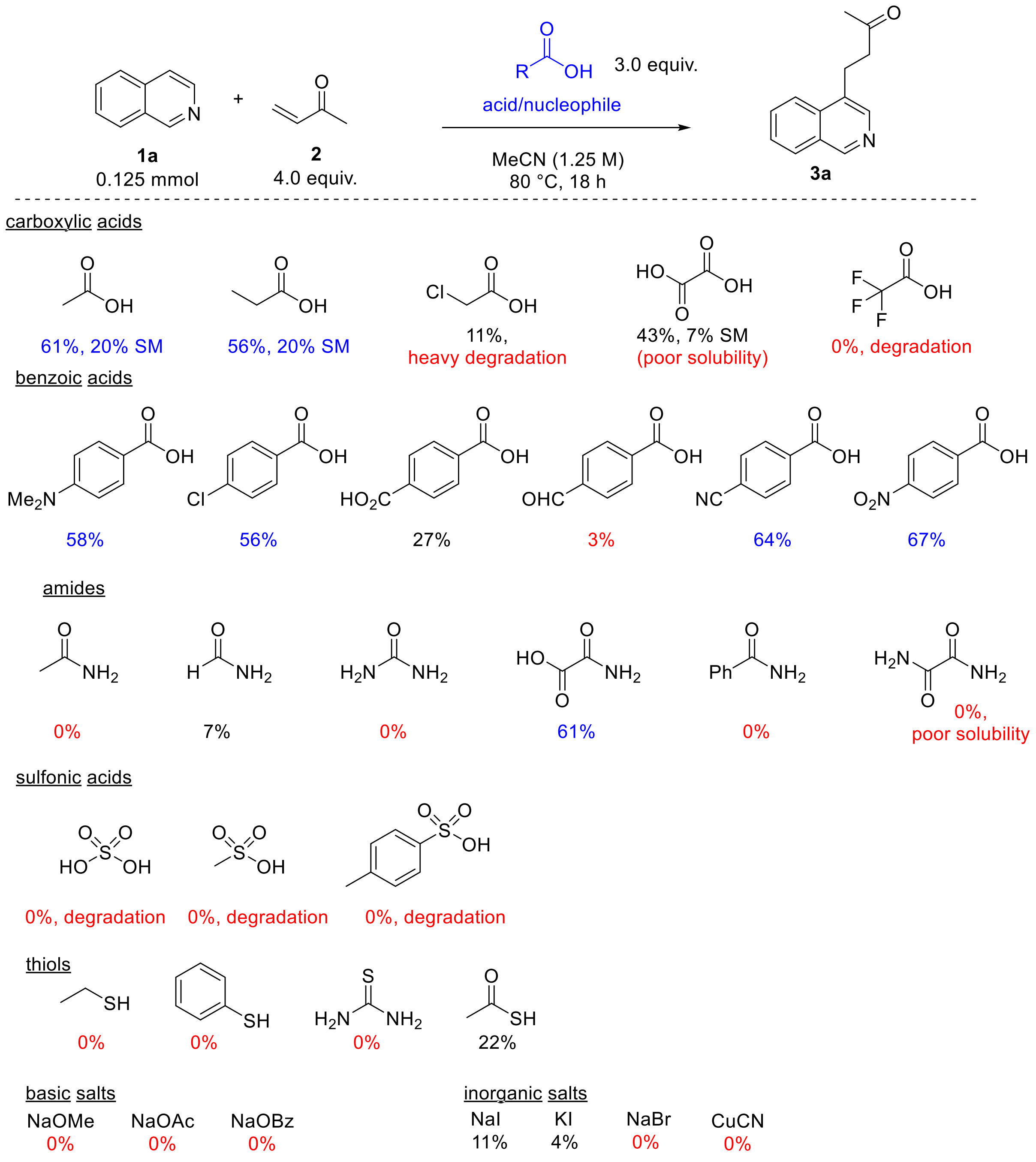

Reactions were performed under the conditions for **General Procedure C** on a 0.125 mmol scale. qNMR yields were determined using trimethoxybenzene as an internal standard.

Figure SI-4: List of Isoquinolines, Quinolines, and Pyridines Screened for the Benzoic Acid Promoted Alkylation Reaction with Methyl Vinyl Ketone

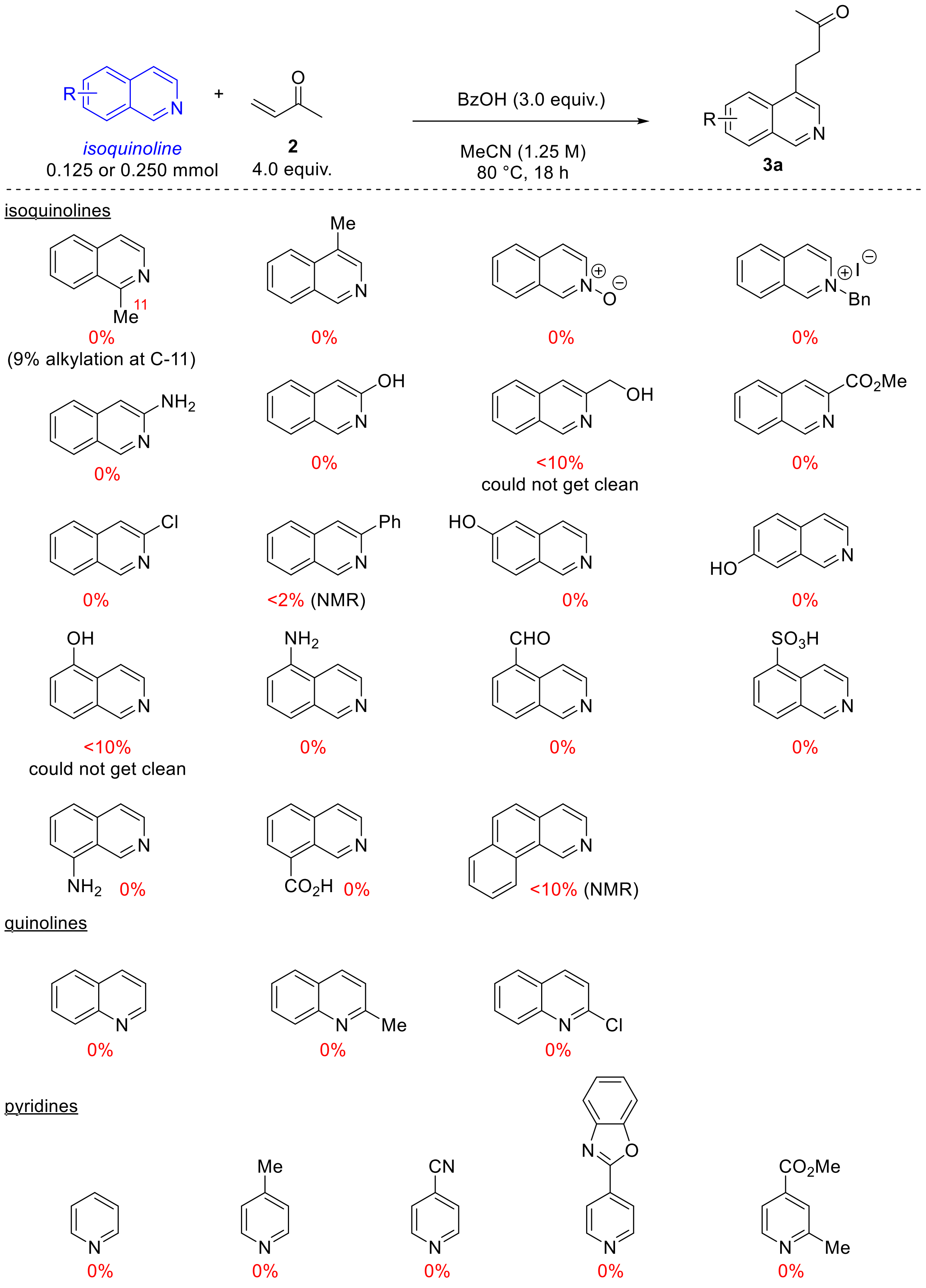

Reactions were performed under the conditions for **General Procedure C** on a 0.125 or 0.250 mmol scale.

Table SI-1: Extended Optimisation Table for the C-4 Alkylation of Isoquinoline with Methyl Vinyl Ketone

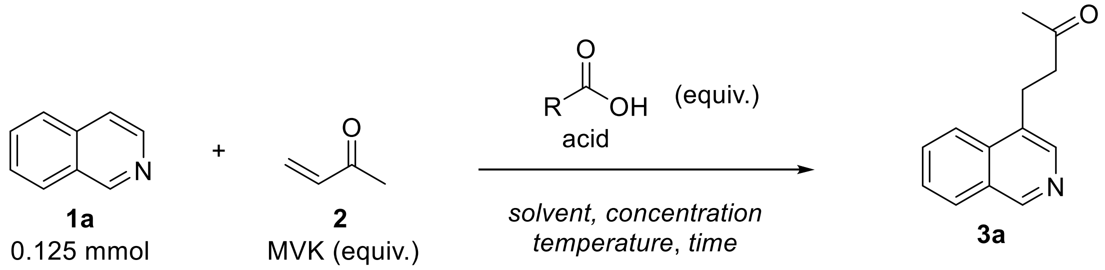

Entry	(°C)	Time (h)	Acid	MVK equiv.	Solvent	Conc. (M)	3a
**1**	45	18	AcOH (3.0)	2.0	MeCN	1.25	43
**2**	45	18	AcOH (3.0)	4.0	MeCN	1.25	54
**3**	45	18	AcOH (1.5)	2.0	MeCN	1.25	40
**4**	45	18	AcOH (2.0)	2.0	MeCN	1.25	17
**5**	65	18	AcOH (3.0)	2.0	MeCN	1.25	53
**6**	80	18	AcOH (3.0)	2.0	MeCN	1.25	45
**7**	65	18	AcOH (3.0)	4.0	MeCN	1.25	61
**8**	80	8	AcOH (3.0)	4.0	MeCN	1.25	59
**9**	80	4	AcOH (3.0)	4.0	MeCN	1.25	58
**10**	80	2	AcOH (3.0)	4.0	MeCN	1.25	60
**11**	80	18	AcOH (3.0)	4.0	Acetone	1.25	62
**12**	80	18	AcOH (3.0)	4.0	DCE	1.25	62
**13**	80	18	AcOH (3.0)	4.0	MeOH	1.25	60
**14**	80	18	AcOH (3.0)	4.0	none	-	70
**15**	80	18	BzOH (3.0)	4.0	MeCN	1.25	73
**16**	80	8	BzOH (3.0)	4.0	MeCN	1.25	67
**17**	80	4	BzOH (3.0)	4.0	MeCN	1.25	60
**18**	80	2	BzOH (3.0)	4.0	MeCN	1.25	56
**19**	80	6	BzOH (3.0)	4.0	MeCN	0.625	49
**20**	80	6	BzOH (3.0)	4.0	MeCN	0.313	27
**21**	80	6	BzOH (3.0)	4.0	MeCN	0.156	14
**22**	80	18	BzOH (1.0)	4.0	MeCN	1.25	48
**23**	80	18	BzOH (2.0)	4.0	MeCN	1.25	47
**24**	80	18	BzOH (6.0)	4.0	MeCN	1.25	54

Reactions were performed under the conditions for **General Procedure C** on a 0.125 mmol scale. qNMR yields were determined using trimethoxybenzene as an internal standard.

4. *NMR Experiment*

To investigate the mechanism of the reaction, an experiment was performed inside an NMR tube with trimethoxybenzene present in the reaction as an internal standard. The reaction was heated in a preheated oil-bath set to 80 °C and removed at regular intervals to collect qNMR data. qNMR was measured at 0 (before heating), 1, 2, 3, 4, and 6 h total heating time as well as after aqueous work up using the conditions of **General Procedure B**.

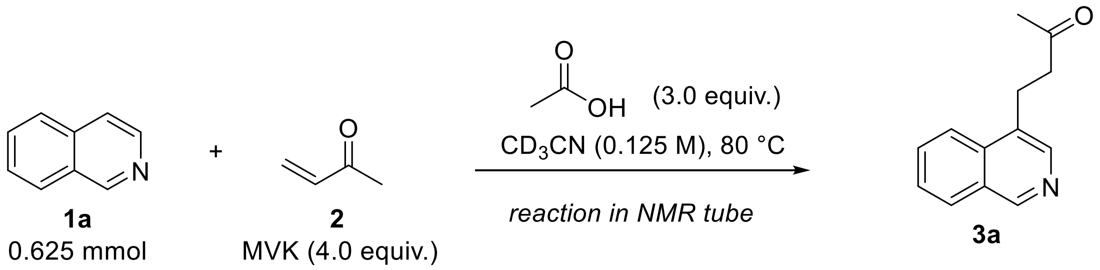

In the reaction, four isoquinoline compounds were observed, these were identified as the starting material **1a**, the product **3a**, as well as an MVK adduct of **1a** and **3a** here labelled **SI-8** and **SI-9** respectively were formed.

Figure SI-5: C-4 Alkylation of Isoquinoline with Methyl Vinyl Ketone Measured Over Time

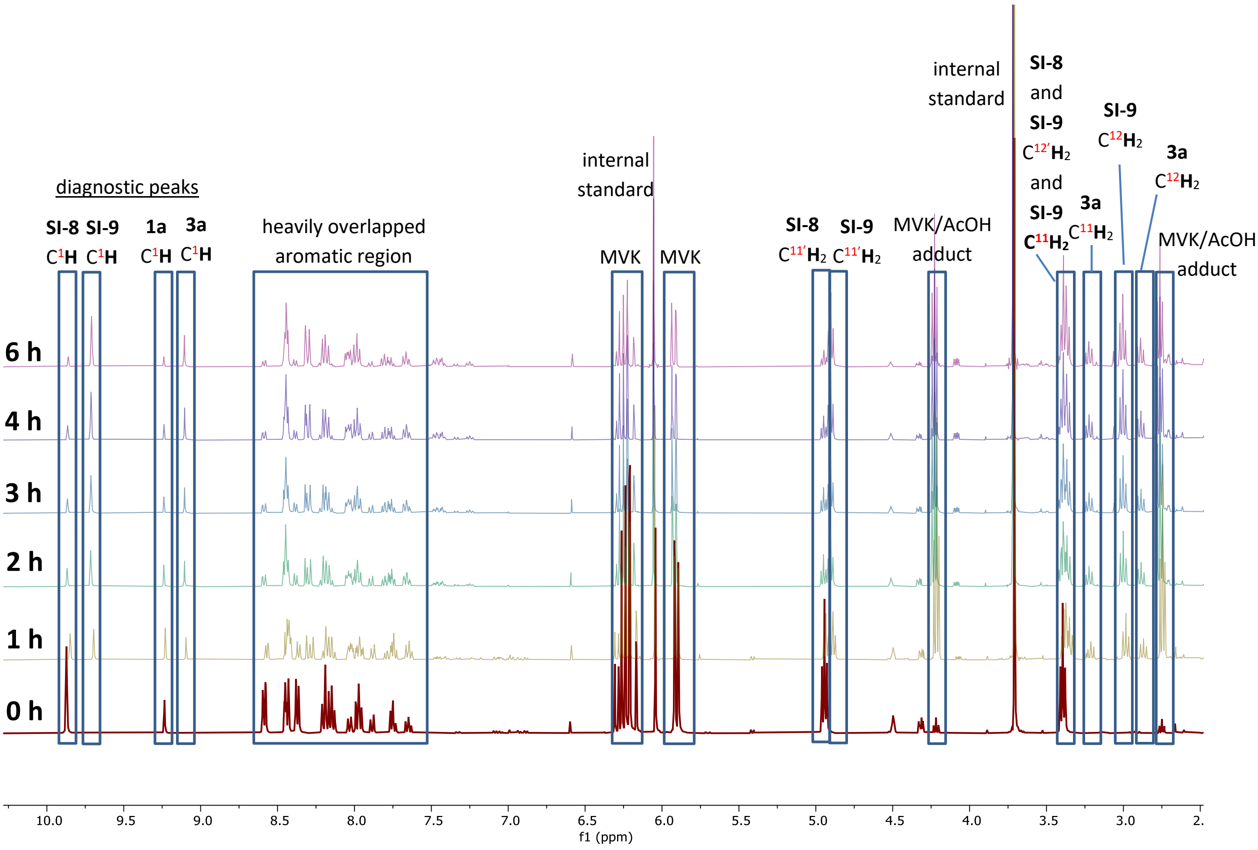

Table SI-2: Conversion of Reactive Species in NMR Reaction

	**1a** (%)	**SI-8** (%)	**3a** (%)	**SI-9** (%)
0 h	21	79	0	0
1 h	18	22	13	29
2 h	14	18	17	39
3 h	12	17	19	45
4 h	10	13	20	47
6 h	7	9	20	49
after work up	6	0	71 (58)	0

The abundance % was determined by qNMR using trimethoxybenzene as an internal standard. The isolated yield of **3a** is shown in parentheses.

Monitoring the reaction by NMR did not allow us to observe the proposed intermediates **A** or **B**.

Figure SI-6: Proposed Formation of **SI-8** and **SI-9**.

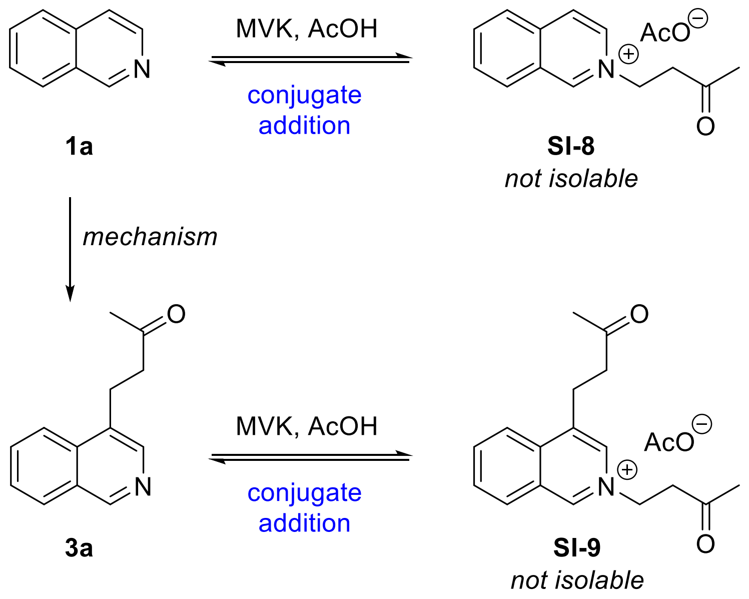

5. *NMR Spectra*

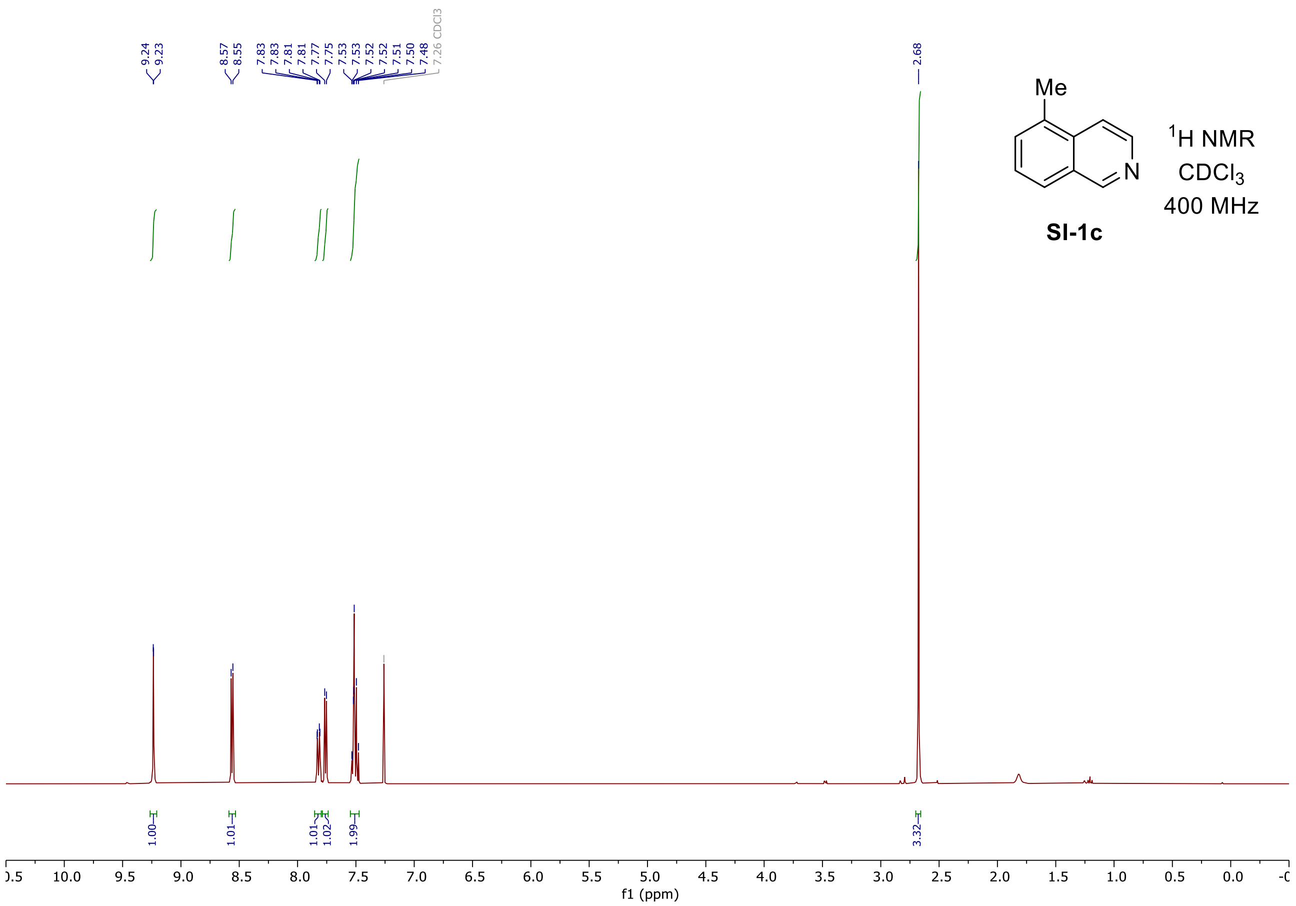

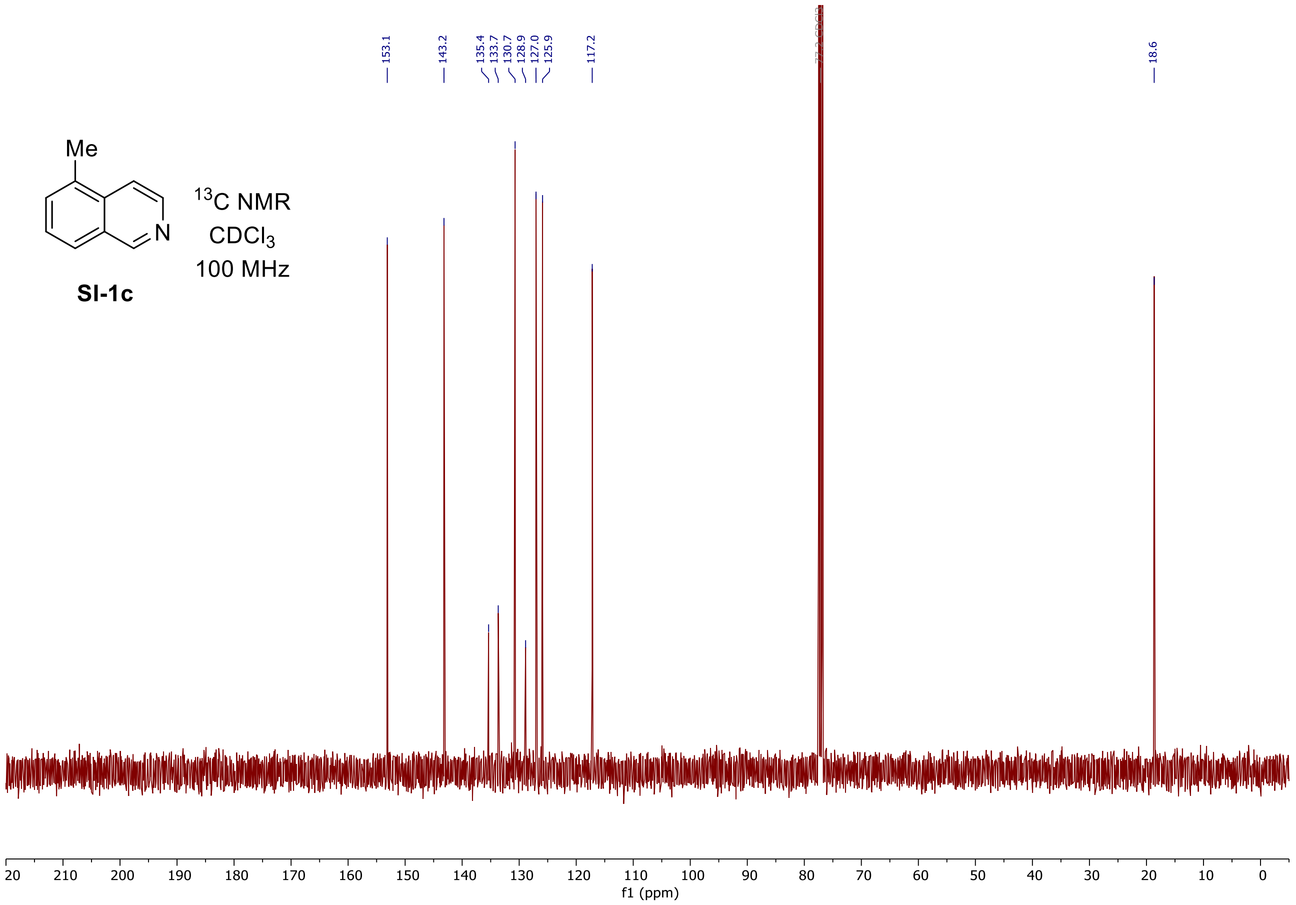

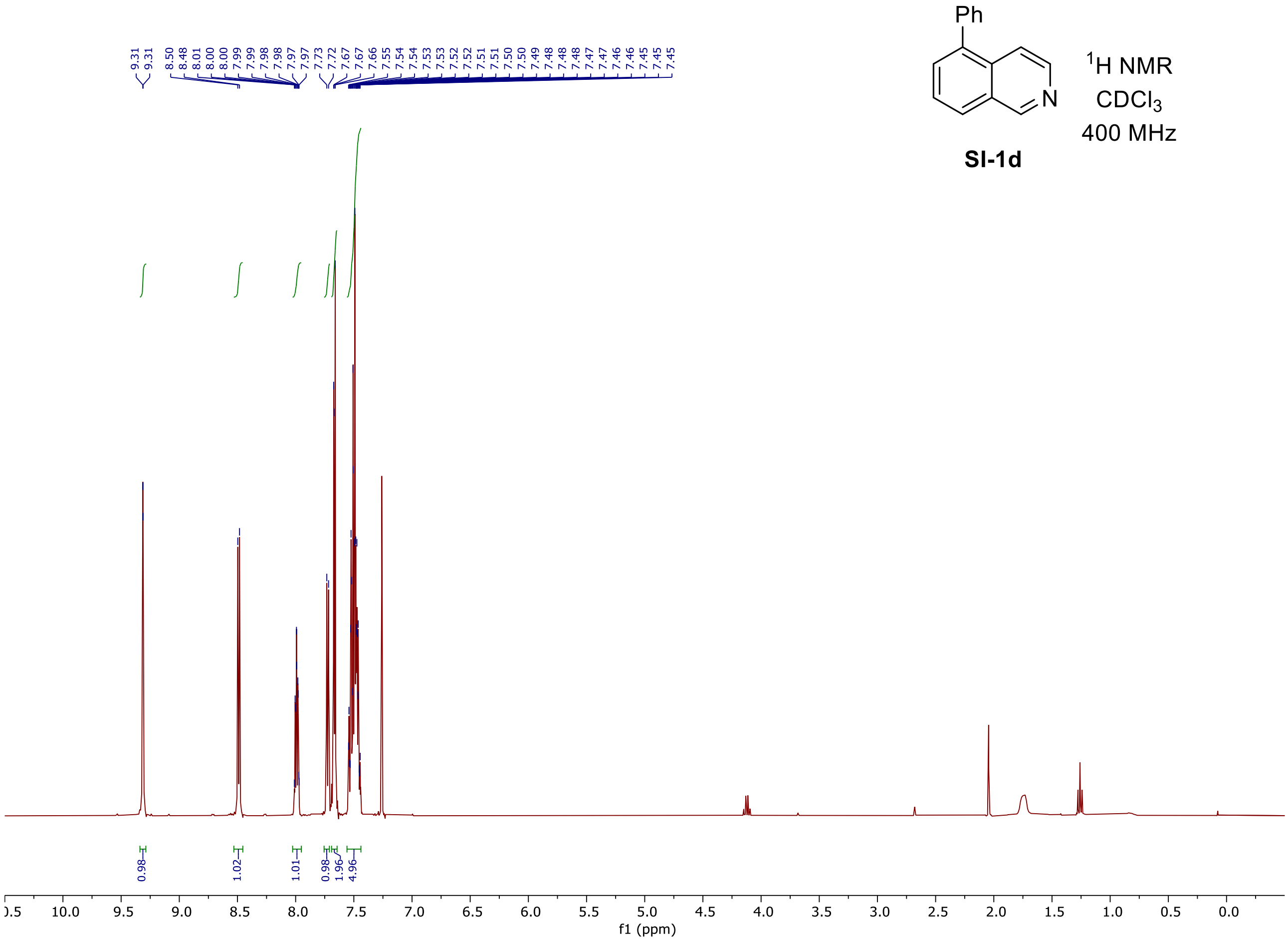

9.31
9.31
8.50
8.48
8.01
8.00
7.99
7.99
7.98
7.98
7.97
7.97
7.73
7.72
7.67
7.67
7.66
7.55
7.54
7.54
7.53
7.53
7.52
7.52
7.51
7.51
7.50
7.49
7.48
7.48
7.48
7.47
7.46
7.46
7.45
7.45
7.45
Ph
N
SI-1d
^{1}H NMR
$CDCl_3$
400 MHz
0.98
1.02
1.01
0.98
1.96
4.96
10.0 9.5 9.0 8.5 8.0 7.5 7.0 6.5 6.0 5.5 5.0 4.5 4.0 3.5 3.0 2.5 2.0 1.5 1.0 0.5 0.0
f1 (ppm)

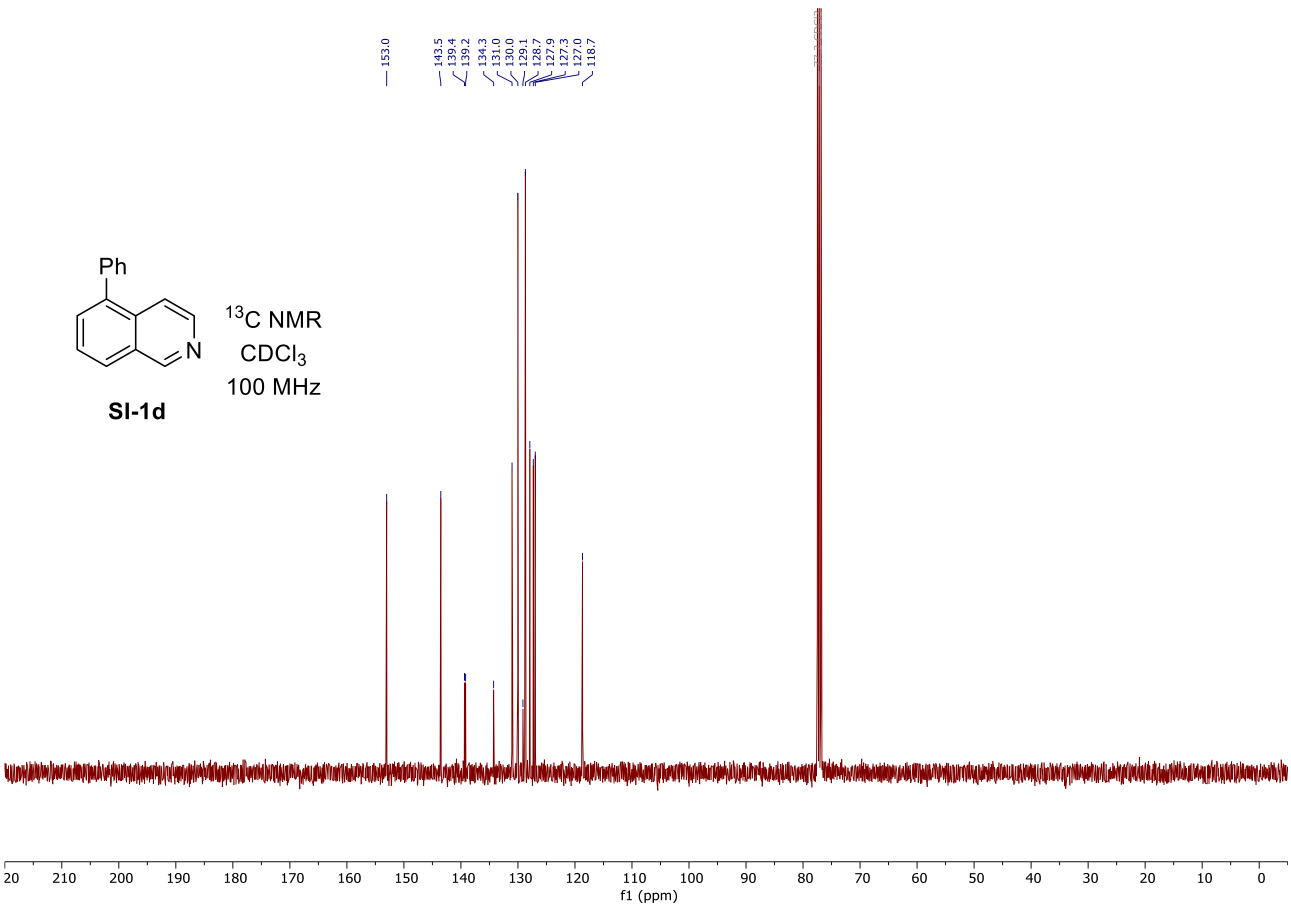

153.0
143.5
139.4
139.2
134.3
131.0
130.0
129.1
128.7
127.9
127.3
127.0
118.7
Ph
N
SI-1d
^{13}C NMR
$CDCl_3$
100 MHz
210 200 190 180 170 160 150 140 130 120 110 100 90 80 70 60 50 40 30 20 10 0
f1 (ppm)

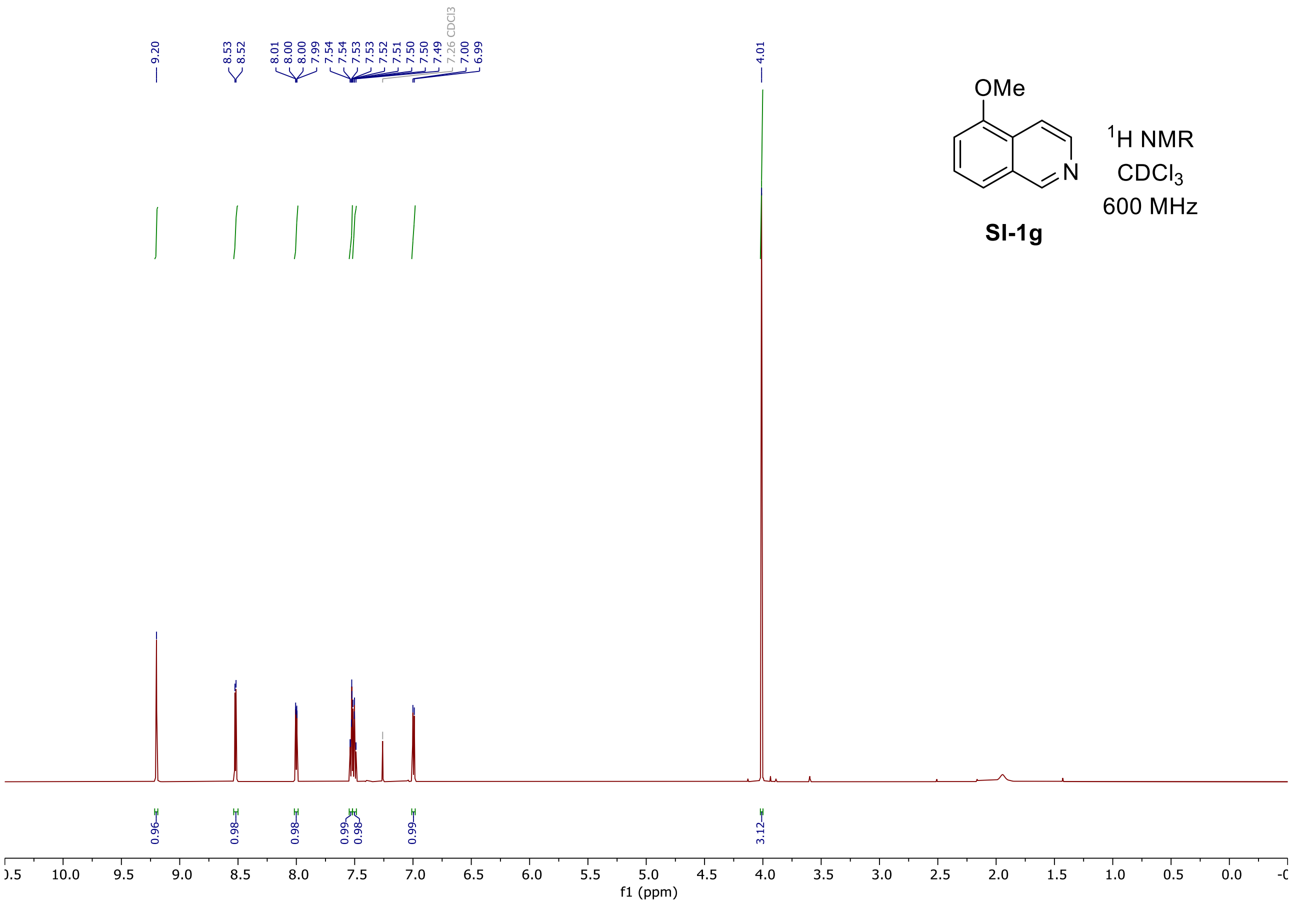

OMe
N
SI-1g
^{1}H NMR
$CDCl_3$
600 MHz
f1 (ppm)

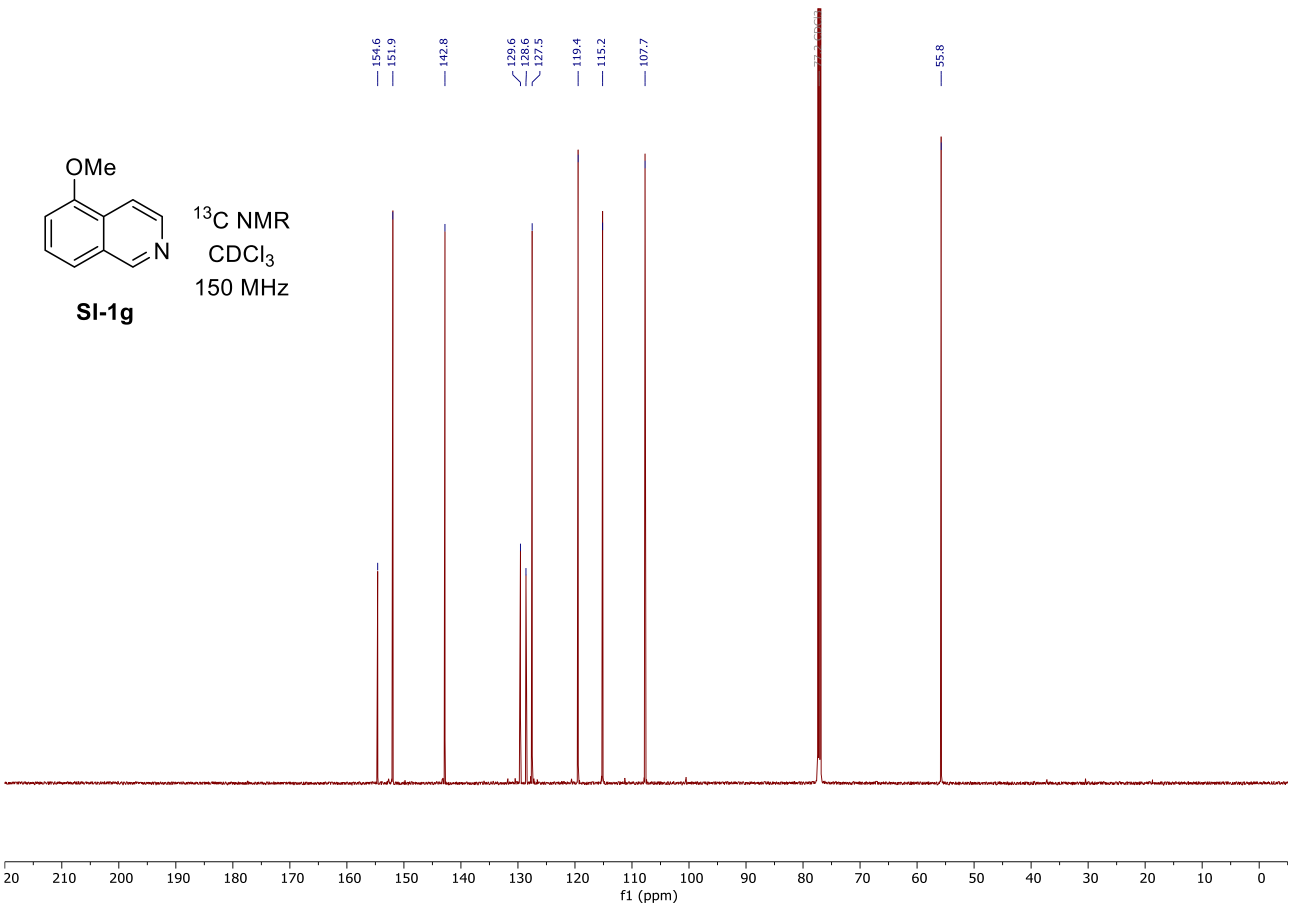

OMe
N
SI-1g
^{13}C NMR
$CDCl_3$
150 MHz
f1 (ppm)

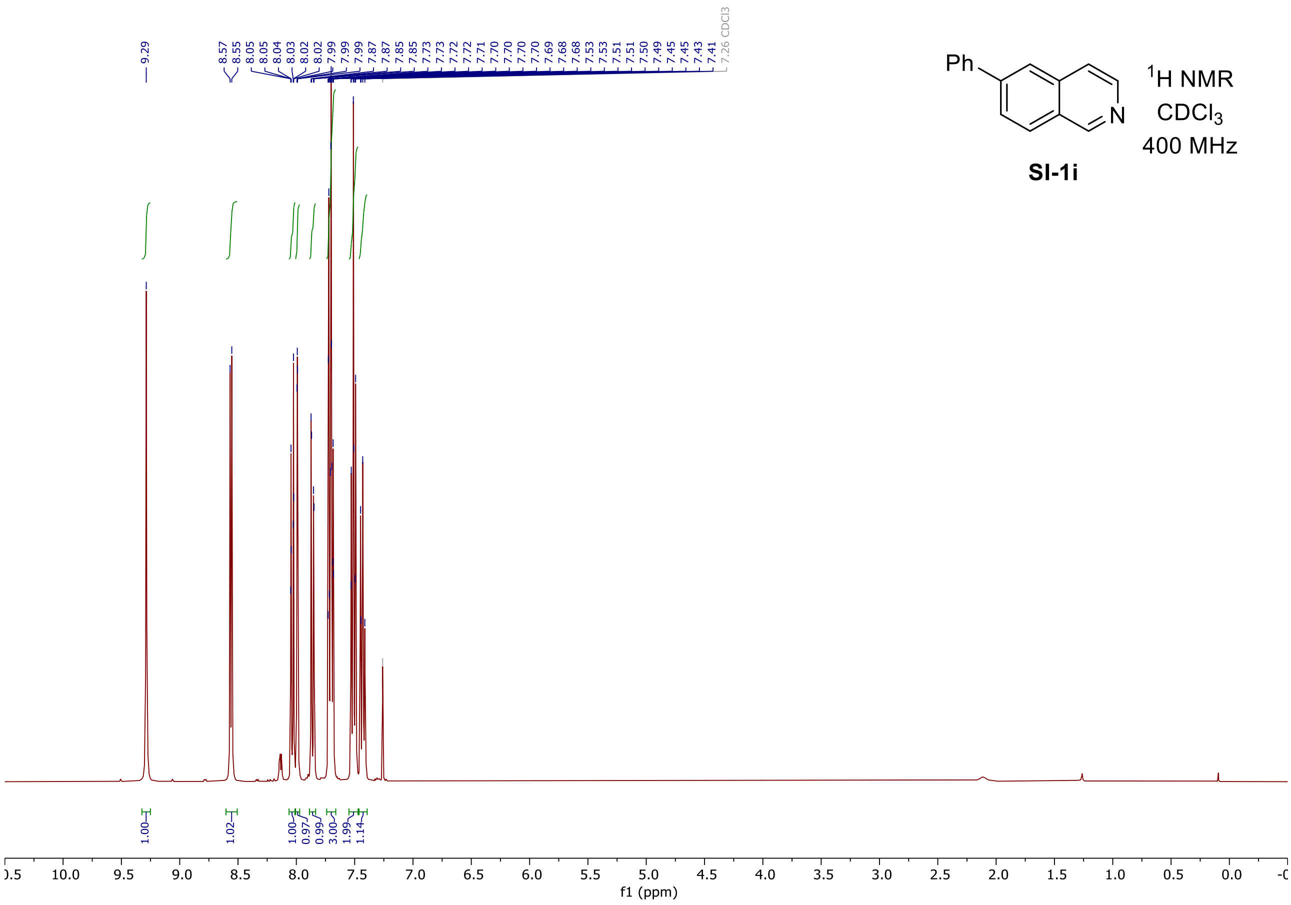
Ph
N
SI-1i
^{1}H NMR
$CDCl_3$
400 MHz
9.29
8.57
8.55
8.05
8.05
8.04
8.03
8.02
8.02
7.99
7.99
7.99
7.87
7.85
7.85
7.73
7.73
7.72
7.71
7.70
7.70
7.70
7.69
7.68
7.53
7.53
7.51
7.50
7.49
7.45
7.45
7.43
7.41
7.26 CDCl3
1.00
1.02
1.00
0.97
0.99
3.00
1.99
1.14
f1 (ppm)

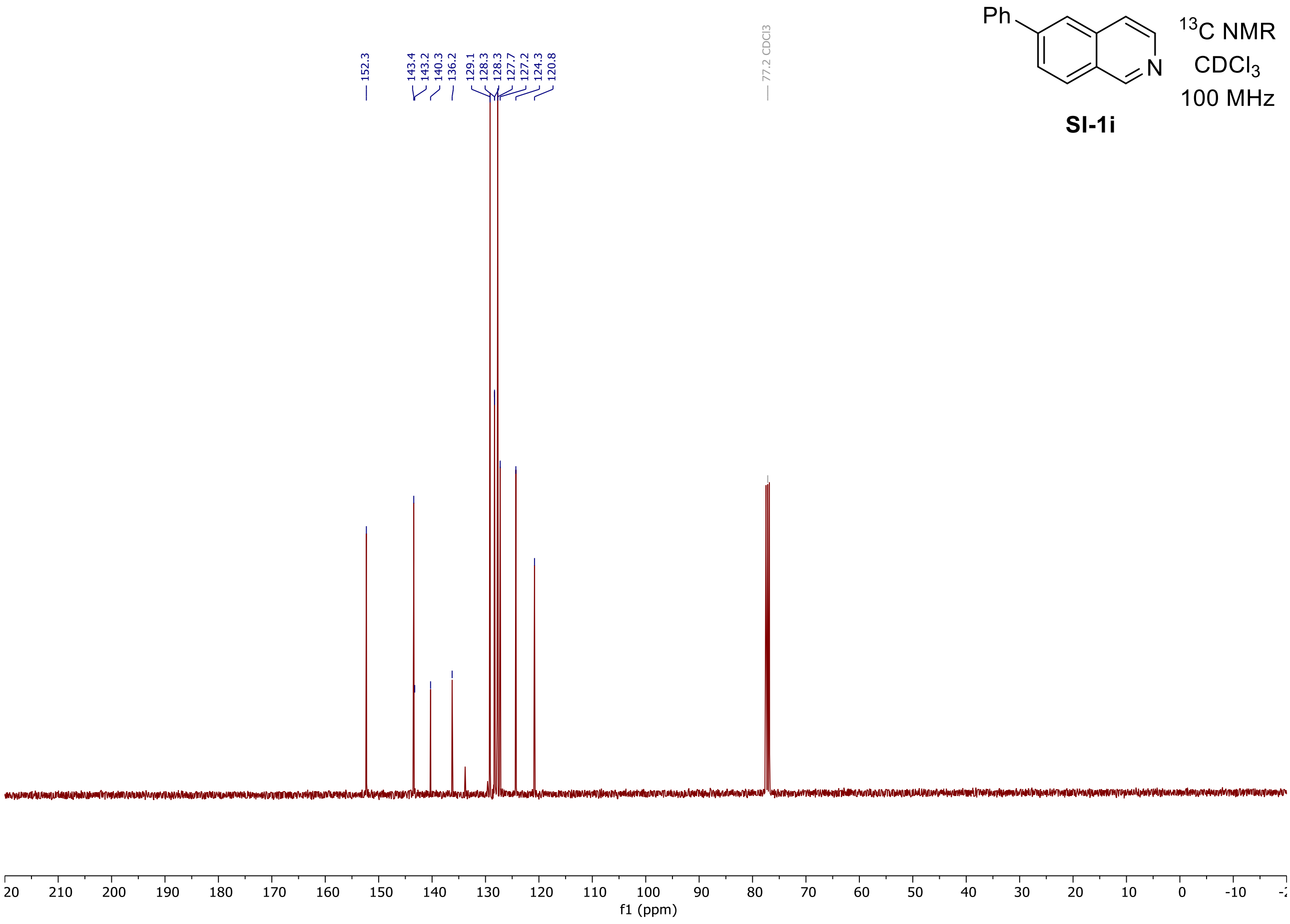
Ph
N
SI-1i
^{13}C NMR
$CDCl_3$
100 MHz
152.3
143.4
143.2
140.3
136.2
129.1
128.3
128.3
127.7
127.2
124.3
120.8
77.2 CDCl3
f1 (ppm)

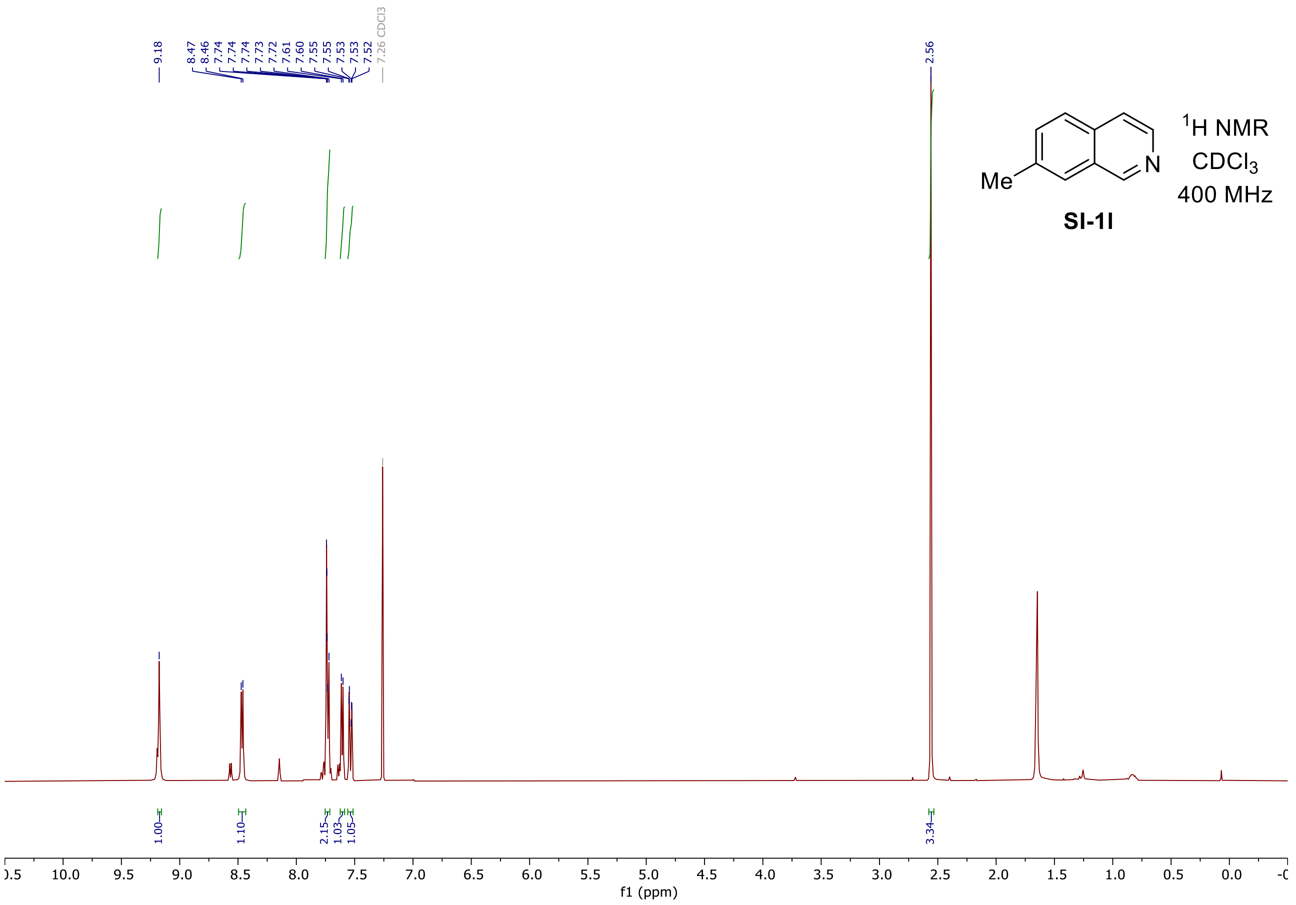
9.18
8.47
8.46
7.74
7.74
7.74
7.73
7.72
7.61
7.60
7.55
7.55
7.53
7.53
7.52
7.26 CDCl3
2.56
Me
N
SI-1l
1H NMR
CDCl3
400 MHz
1.00
1.10
2.15
1.03
1.05
3.34
f1 (ppm)

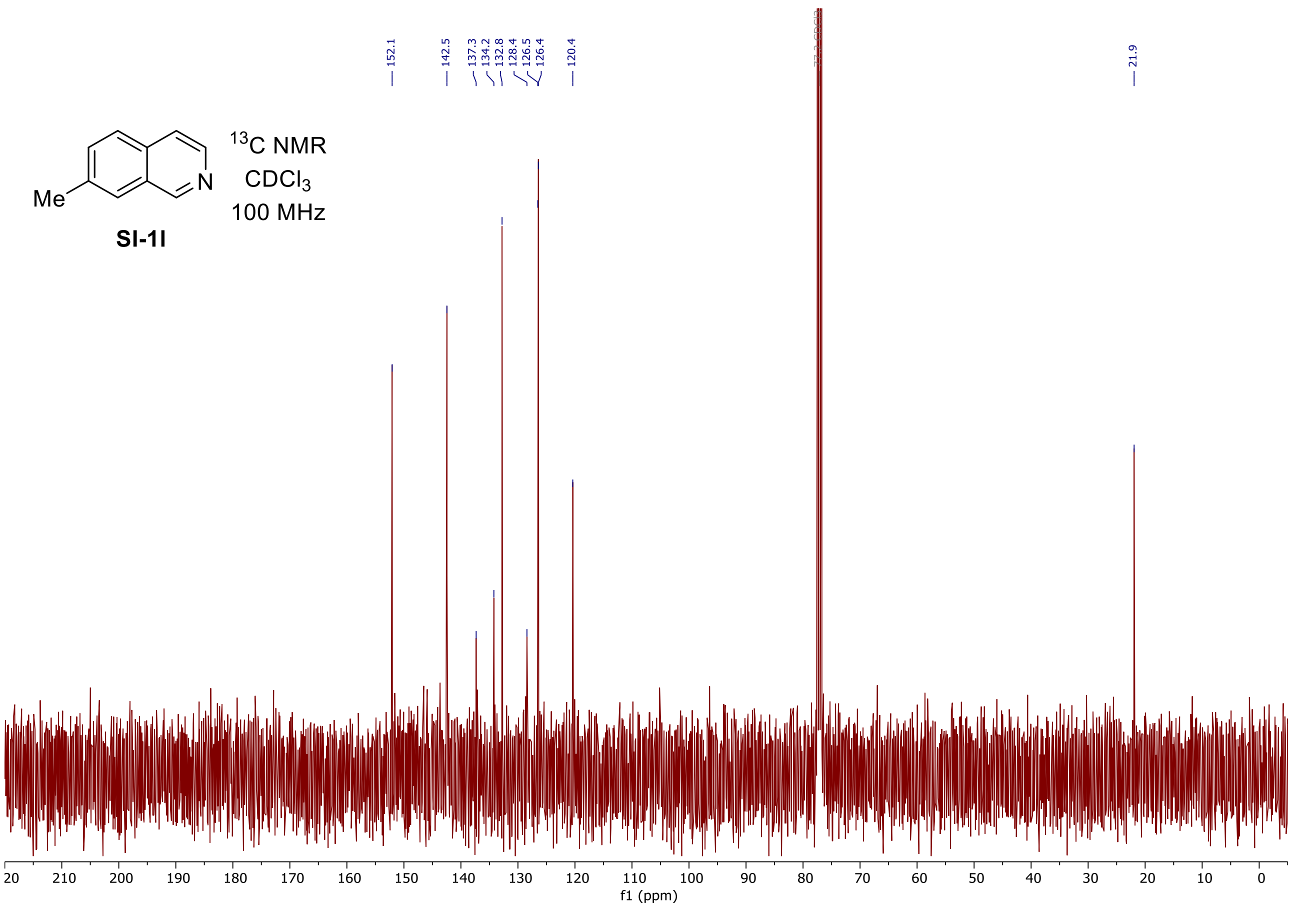
152.1
142.5
137.3
134.2
132.8
128.4
126.5
126.4
120.4
21.9
Me
N
SI-1l
13C NMR
CDCl3
100 MHz
f1 (ppm)

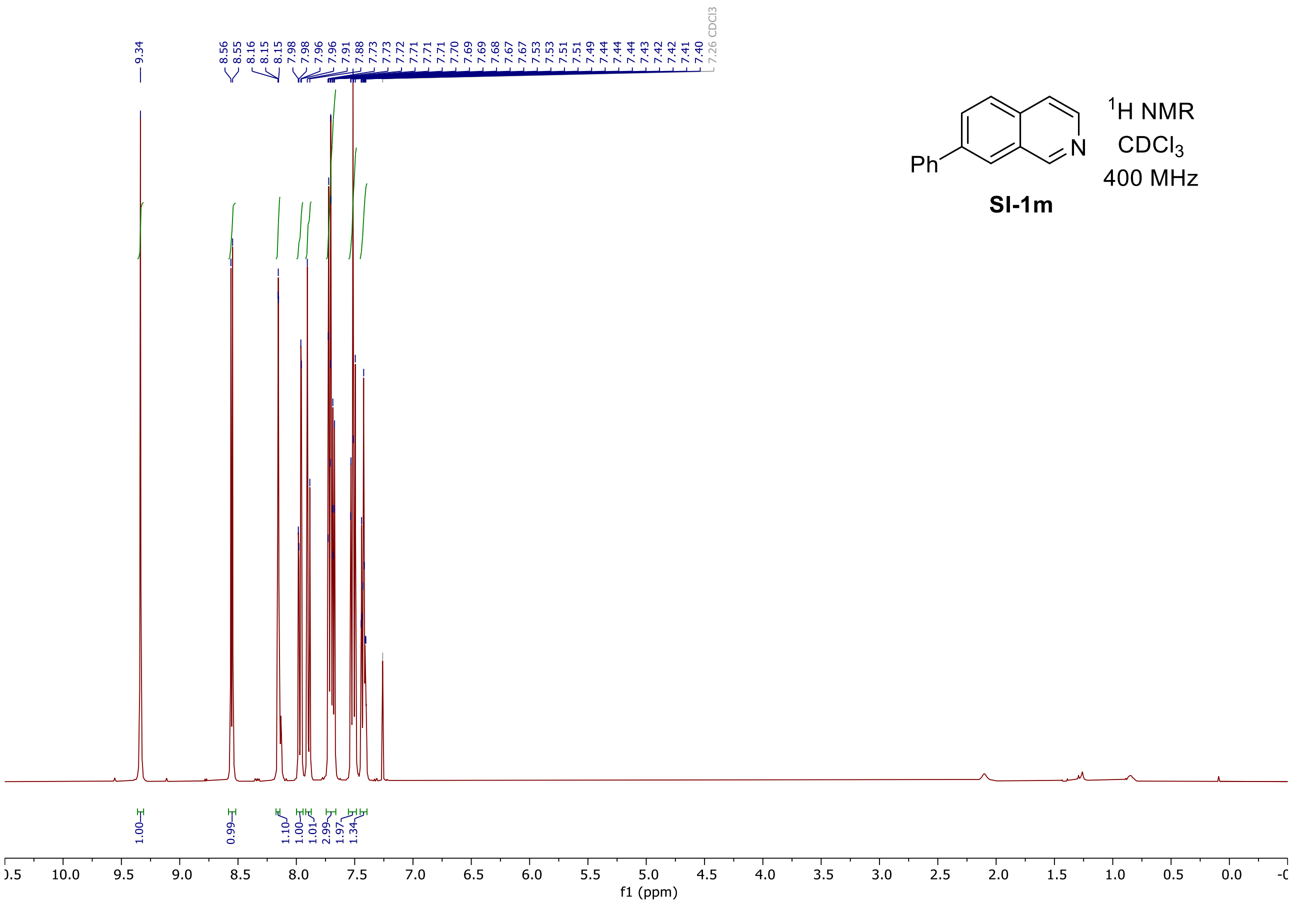

9.34
8.56
8.55
8.16
8.15
8.15
7.98
7.98
7.96
7.96
7.91
7.88
7.73
7.73
7.72
7.71
7.71
7.71
7.70
7.69
7.69
7.68
7.67
7.67
7.53
7.53
7.51
7.51
7.49
7.44
7.44
7.43
7.42
7.42
7.41
7.40
7.26 CDCl3
Ph
N
SI-1m
1H NMR
CDCl3
400 MHz
1.00
0.99
1.10
1.00
1.01
2.99
1.97
1.34
f1 (ppm)

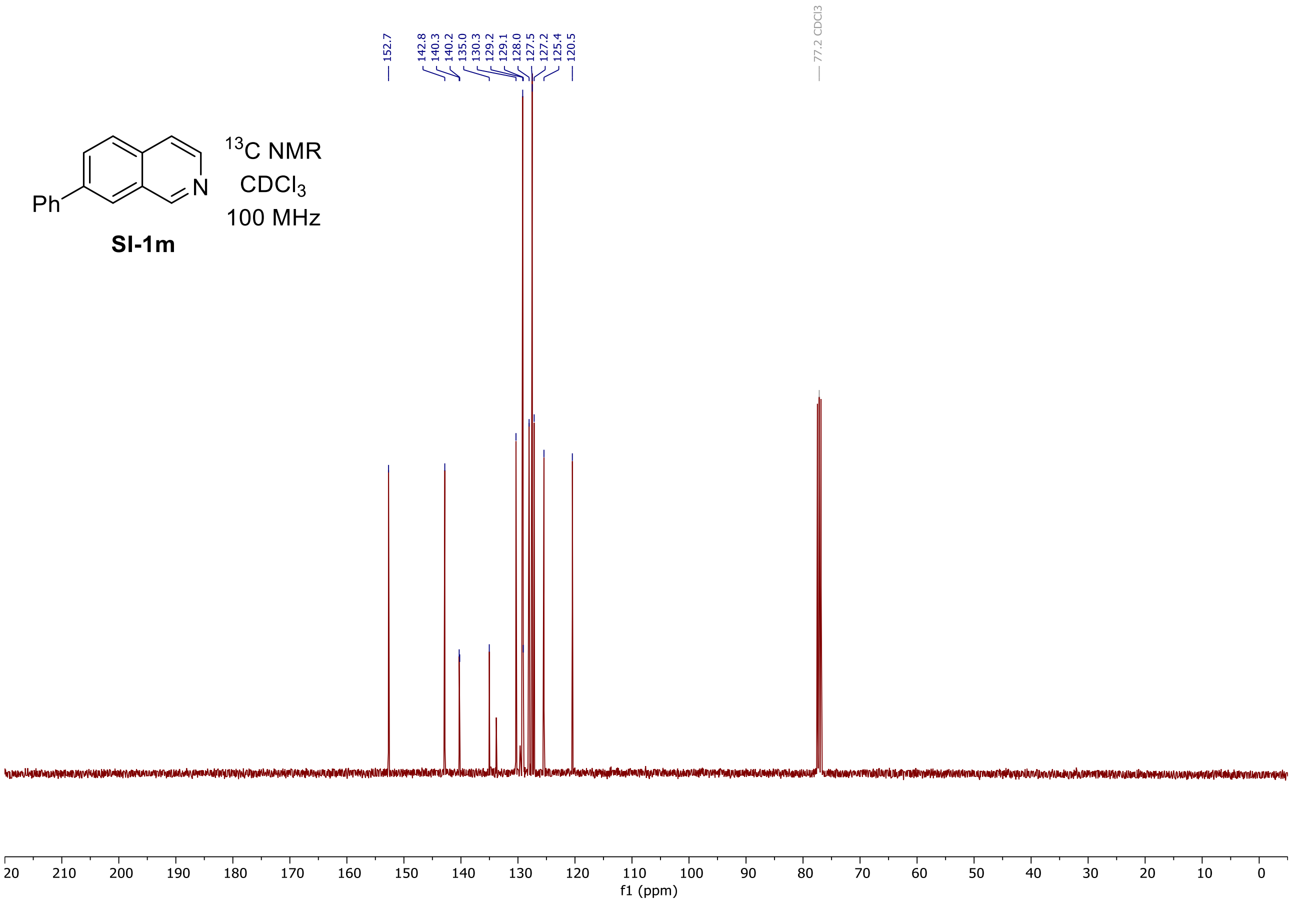

152.7
142.8
140.3
140.2
135.0
130.3
129.2
129.1
128.0
127.5
127.2
125.4
120.5
77.2 CDCl3
Ph
N
SI-1m
13C NMR
CDCl3
100 MHz
f1 (ppm)

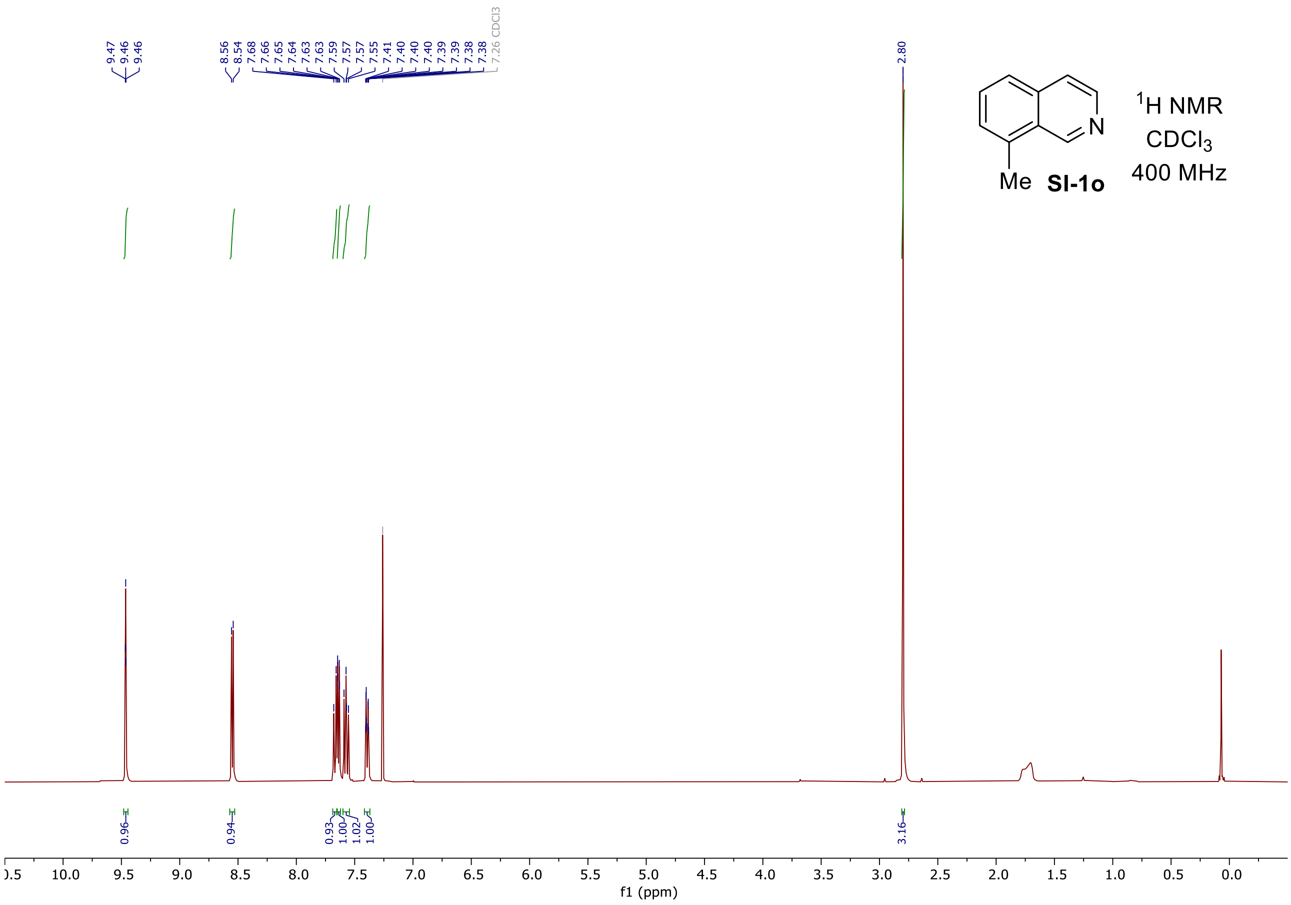
9.47
9.46
9.46
8.56
8.54
7.68
7.66
7.65
7.64
7.63
7.63
7.59
7.57
7.57
7.55
7.41
7.40
7.40
7.40
7.39
7.39
7.38
7.38
7.26 CDCl3
2.80
Me
SI-1o
^{1}H NMR
$CDCl_3$
400 MHz
0.96
0.94
0.93
1.00
1.02
1.00
3.16
f1 (ppm)

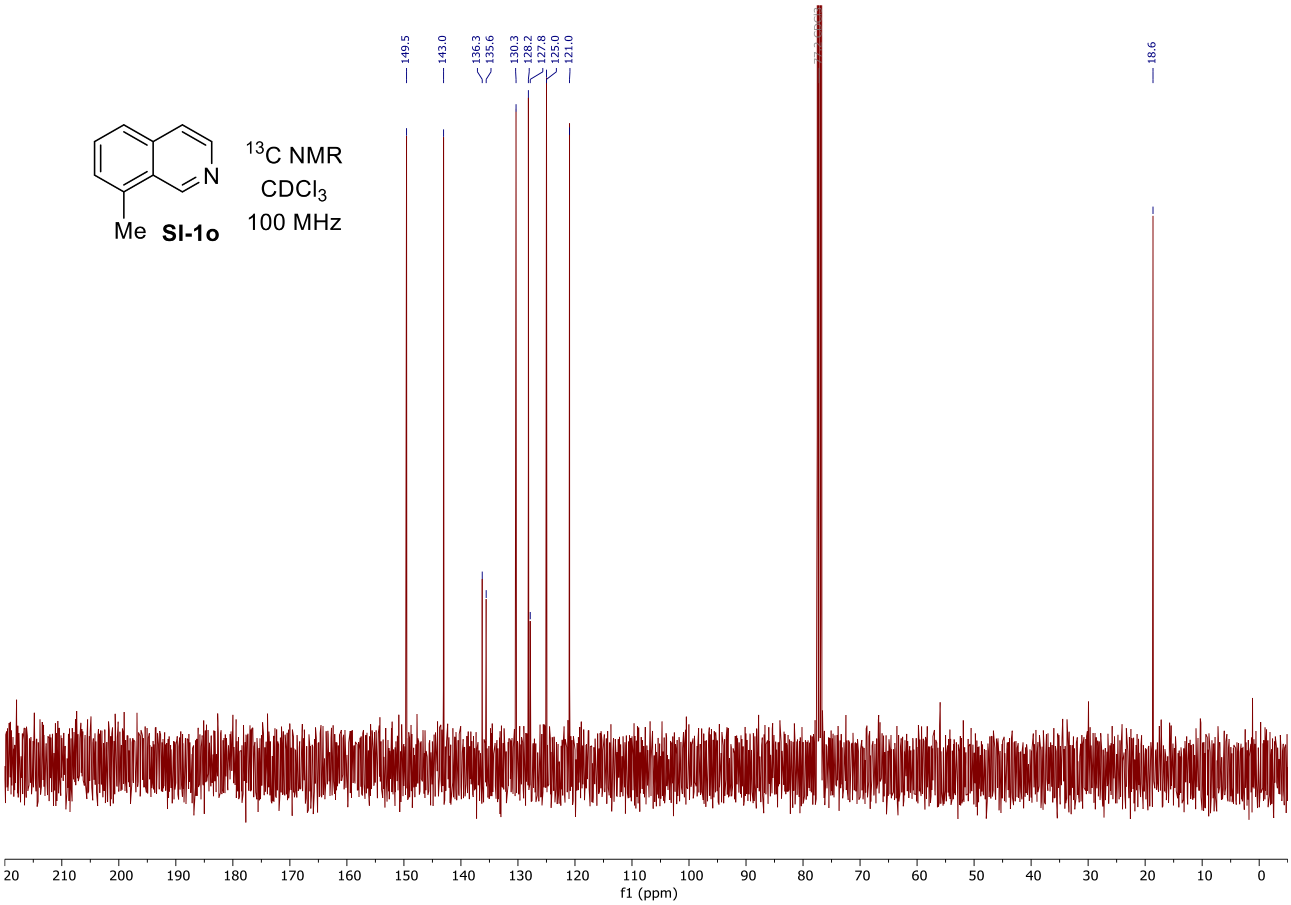
149.5
143.0
136.3
135.6
130.3
128.2
127.8
125.0
121.0
18.6
Me
SI-1o
^{13}C NMR
$CDCl_3$
100 MHz
f1 (ppm)

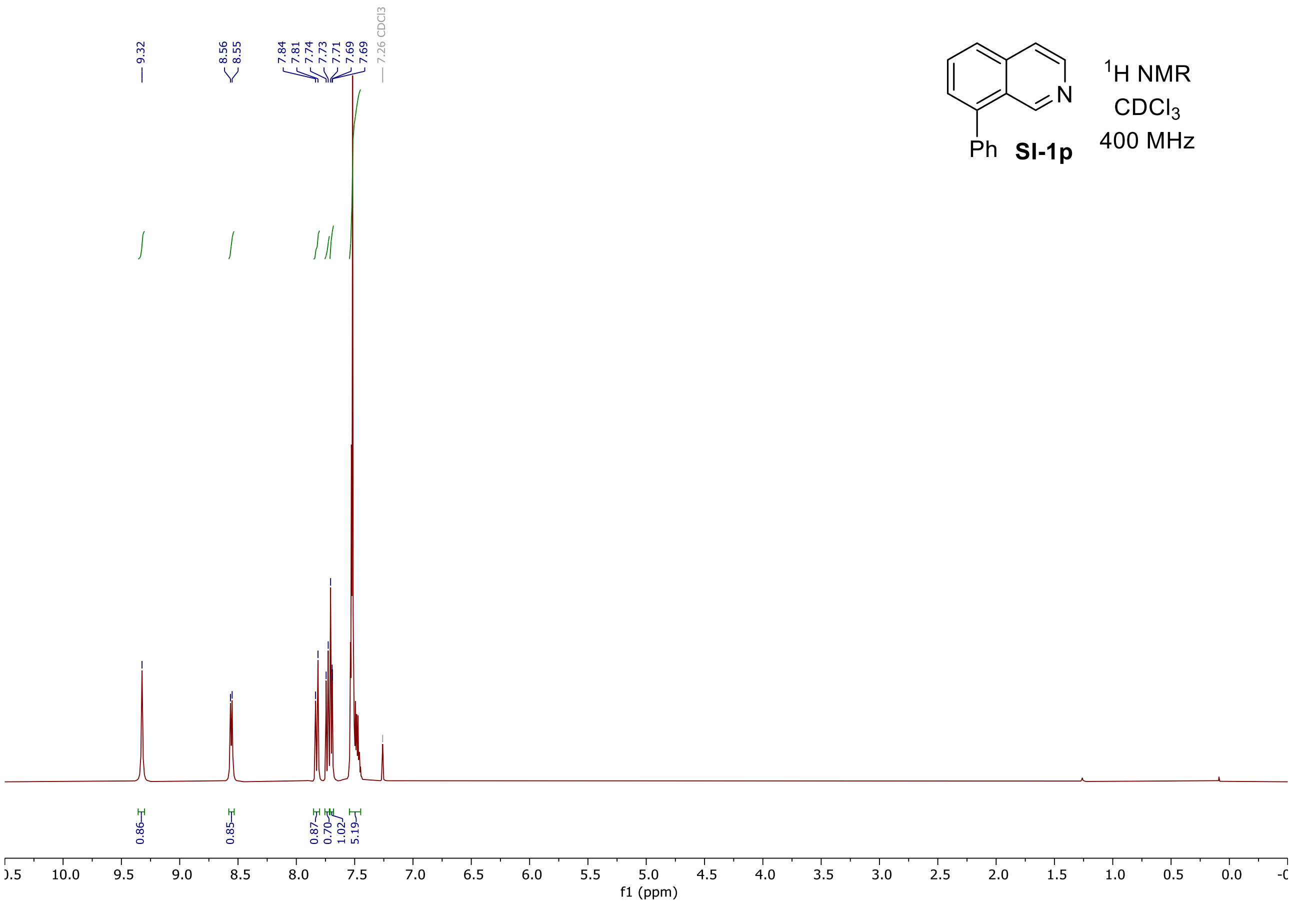
9.32
8.56
8.55
7.84
7.81
7.74
7.73
7.71
7.69
7.69
7.26 CDCl3
N
Ph
SI-1p
1H NMR
CDCl3
400 MHz
0.86
0.85
0.87
0.70
1.02
5.19
f1 (ppm)

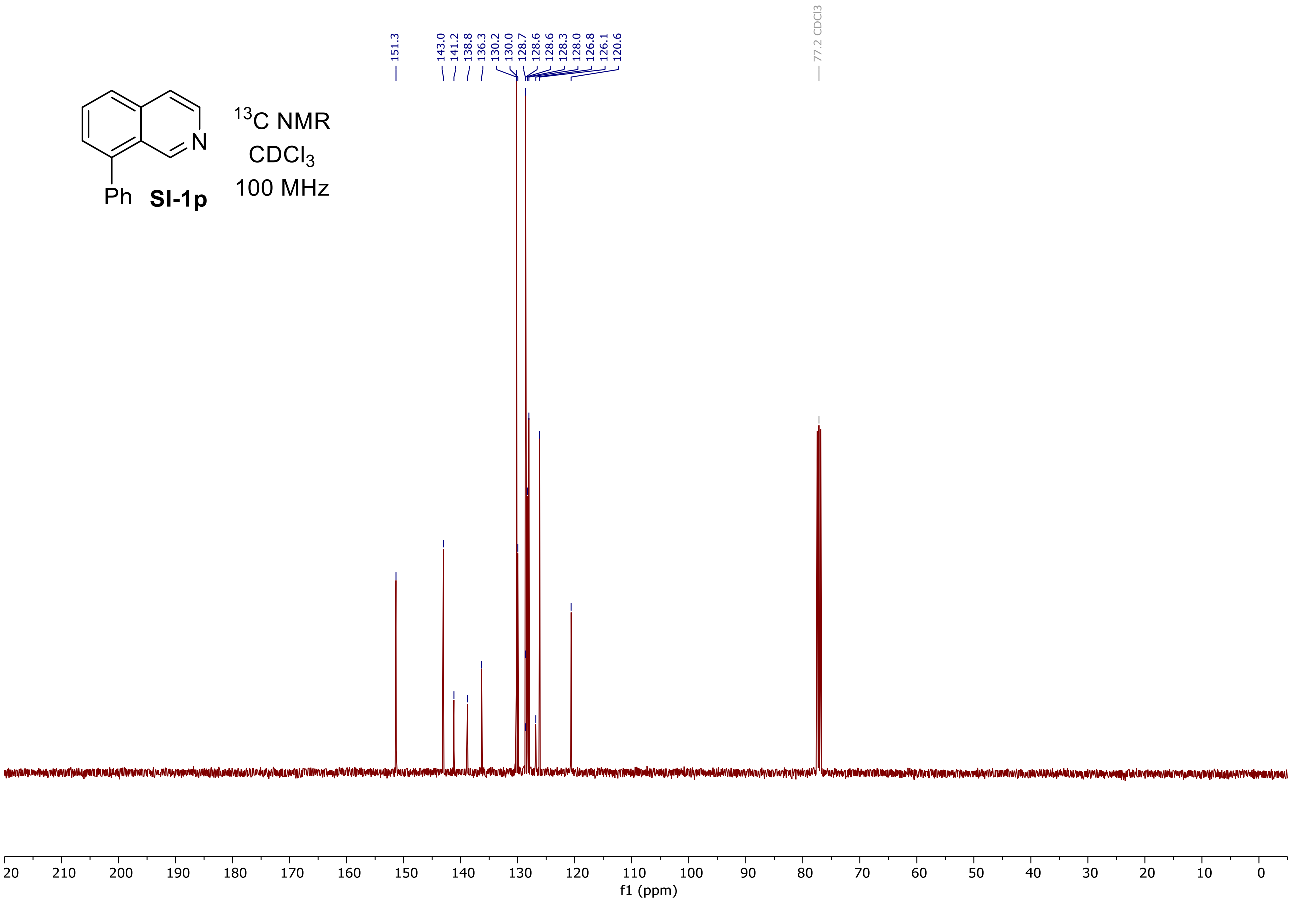
151.3
143.0
141.2
138.8
136.3
130.2
130.0
128.7
128.6
128.6
128.3
128.0
126.8
126.1
120.6
77.2 CDCl3
N
Ph
SI-1p
13C NMR
CDCl3
100 MHz
f1 (ppm)

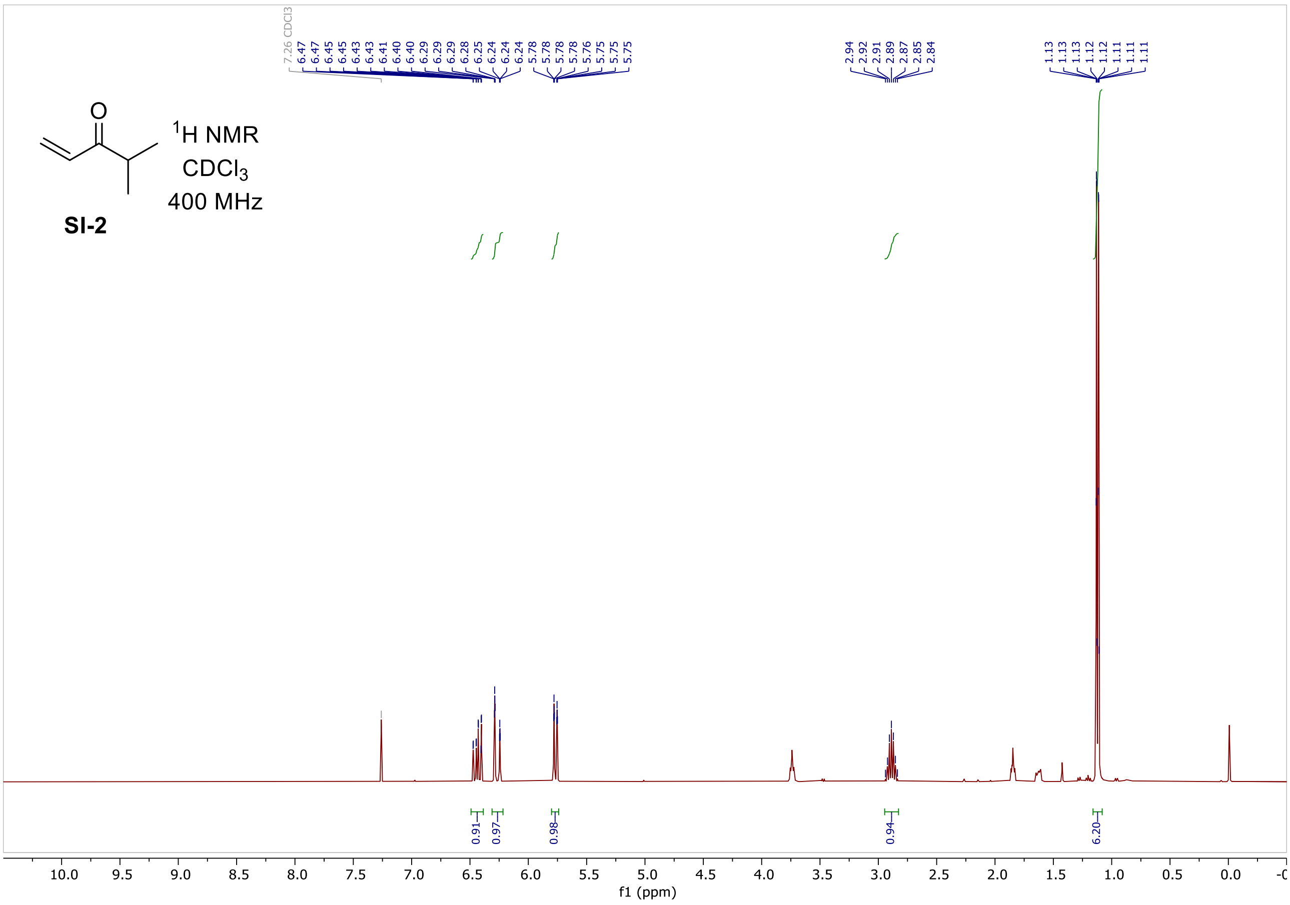

1H NMR
CDCl3
400 MHz
SI-2
7.26 CDCl3
6.47 6.47 6.45 6.45 6.43 6.43 6.41 6.40 6.40 6.29 6.29 6.29 6.28 6.25 6.24 6.24 6.24
5.78 5.78 5.78 5.76 5.75 5.75 5.75
2.94 2.92 2.91 2.89 2.87 2.85 2.84
1.13 1.13 1.13 1.12 1.12 1.11 1.11 1.11
0.91 0.97 0.98 0.94 6.20
f1 (ppm)

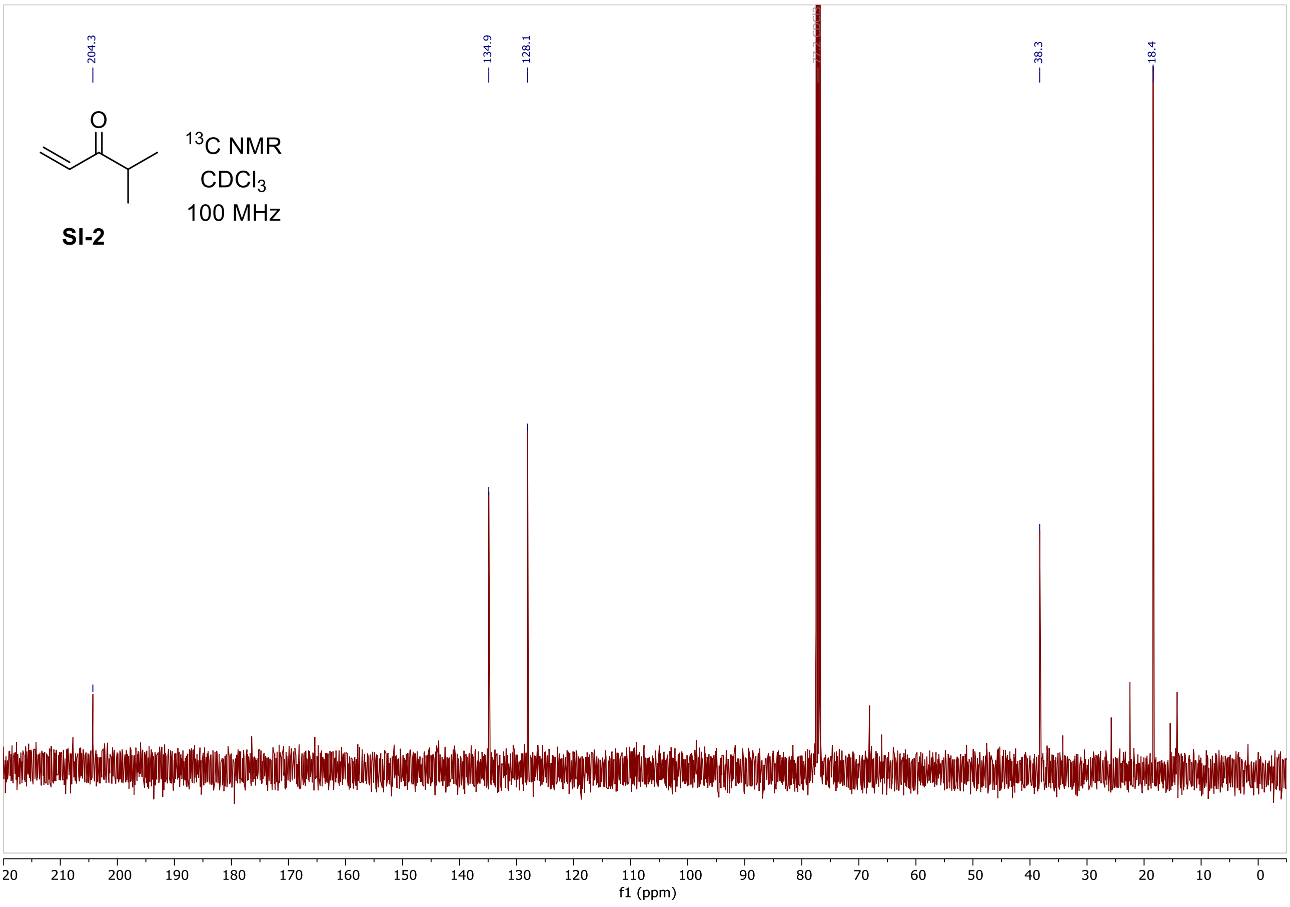

13C NMR
CDCl3
100 MHz
SI-2
204.3
134.9
128.1
38.3
18.4
f1 (ppm)

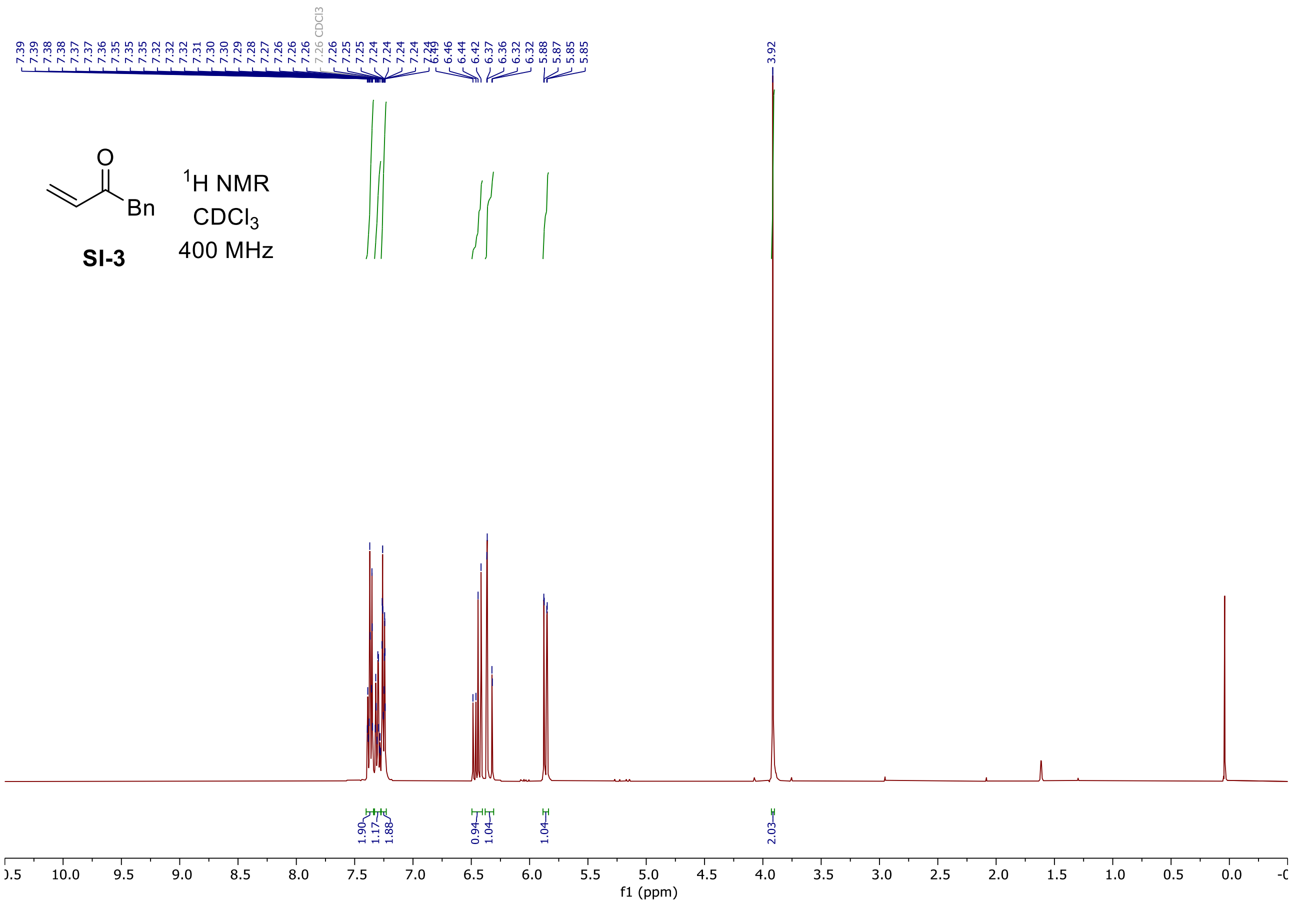
1H NMR
CDCl3
400 MHz
SI-3
f1 (ppm)

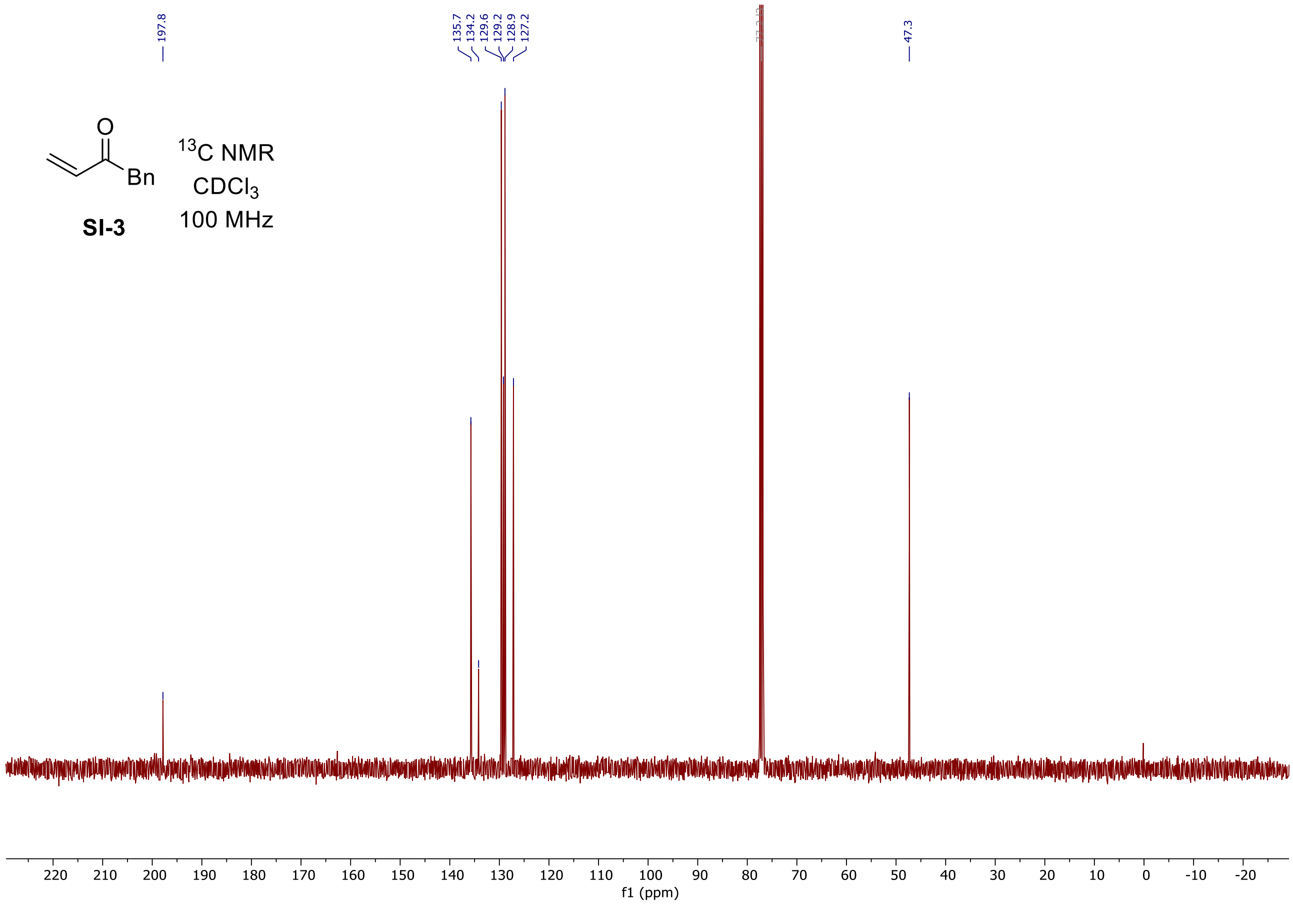
13C NMR
CDCl3
100 MHz
SI-3
197.8
135.7
134.2
129.6
129.2
128.9
127.2
47.3
f1 (ppm)

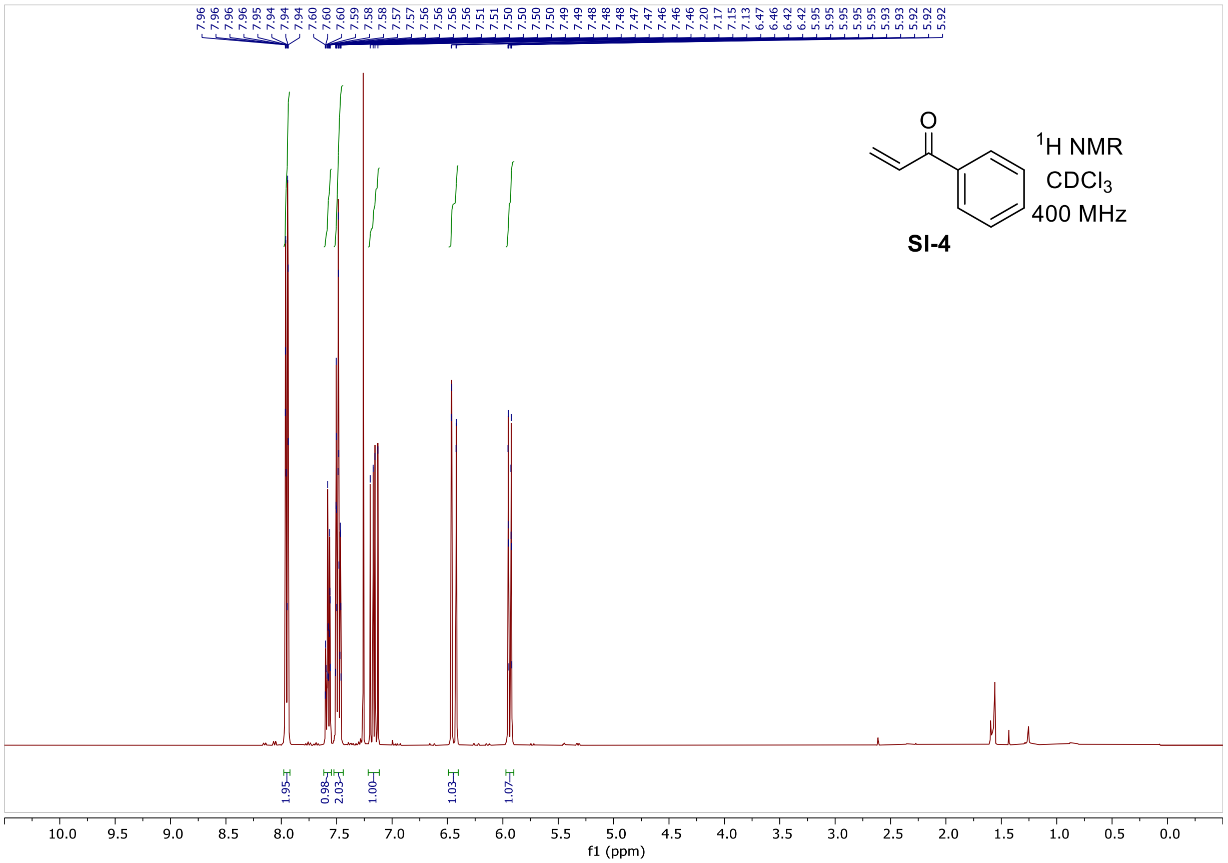

SI-4
1H NMR
CDCl3
400 MHz
f1 (ppm)

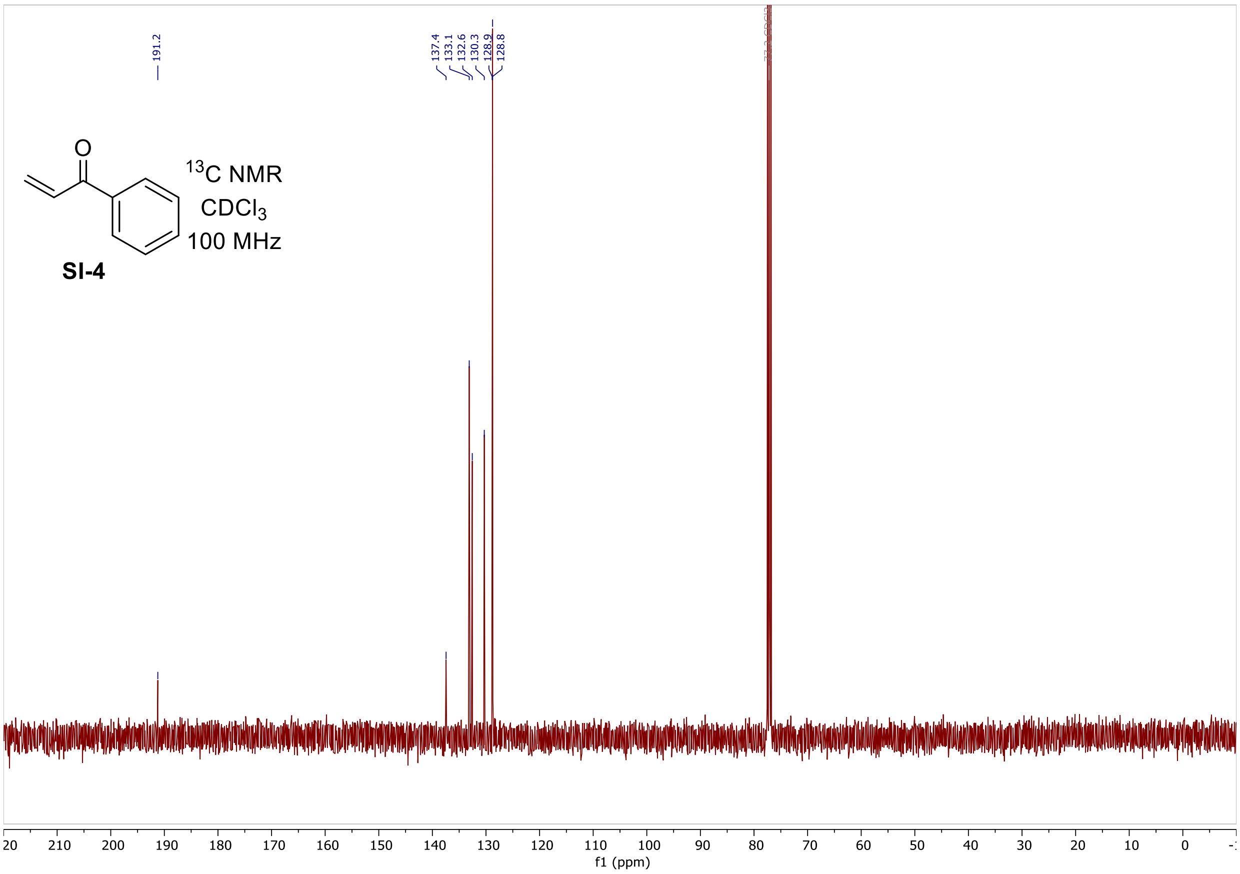

191.2
137.4
133.1
132.6
130.3
128.9
128.8
SI-4
13C NMR
CDCl3
100 MHz
f1 (ppm)

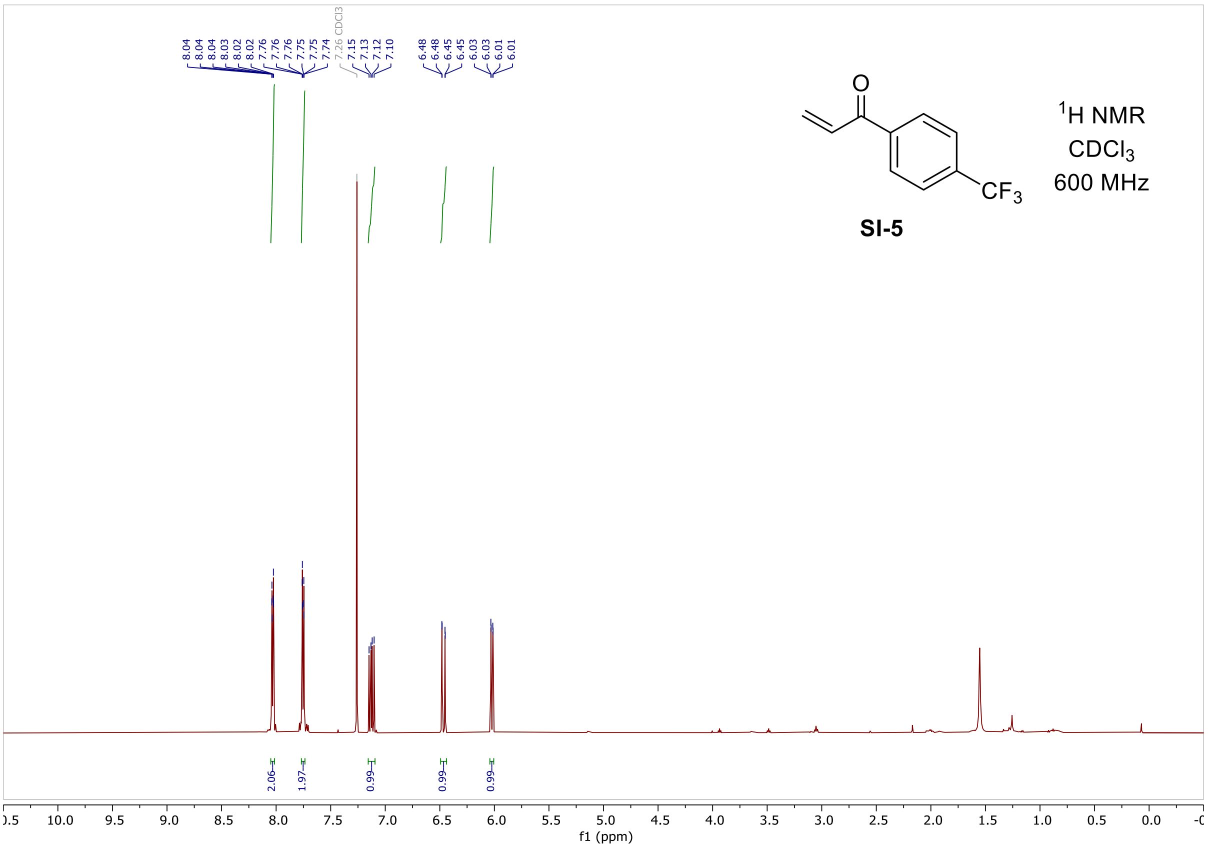
8.04 8.04 8.04 8.03 8.02 8.02 7.76 7.76 7.75 7.75 7.74
7.26 CDCl3
7.15 7.13 7.12 7.10
6.48 6.48 6.45 6.45
6.03 6.03 6.01 6.01
O
CF3
SI-5
1H NMR
CDCl3
600 MHz
2.06
1.97
0.99
0.99
0.99
f1 (ppm)

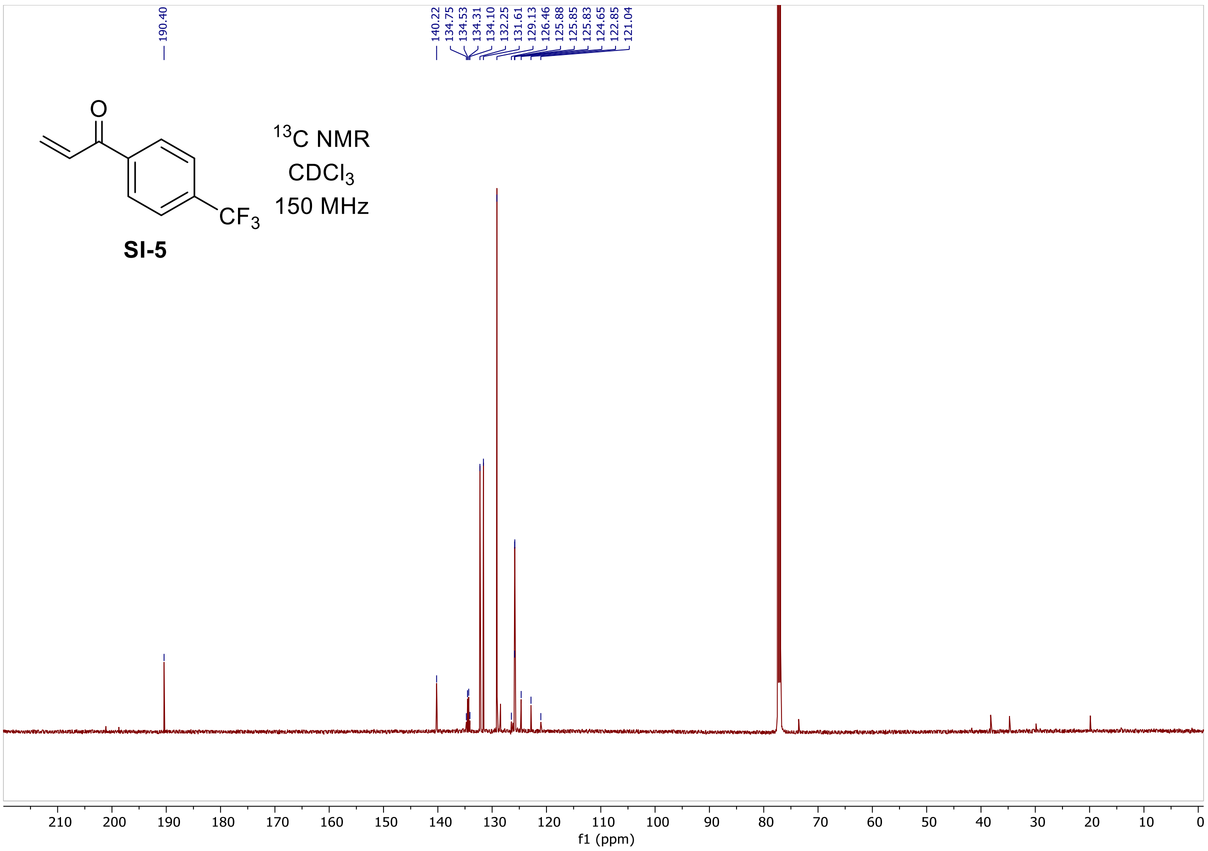
190.40
140.22 134.75 134.53 134.31 134.10 132.25 131.61 129.13 126.46 125.88 125.85 125.83 124.65 122.85 121.04
O
CF3
SI-5
13C NMR
CDCl3
150 MHz
f1 (ppm)

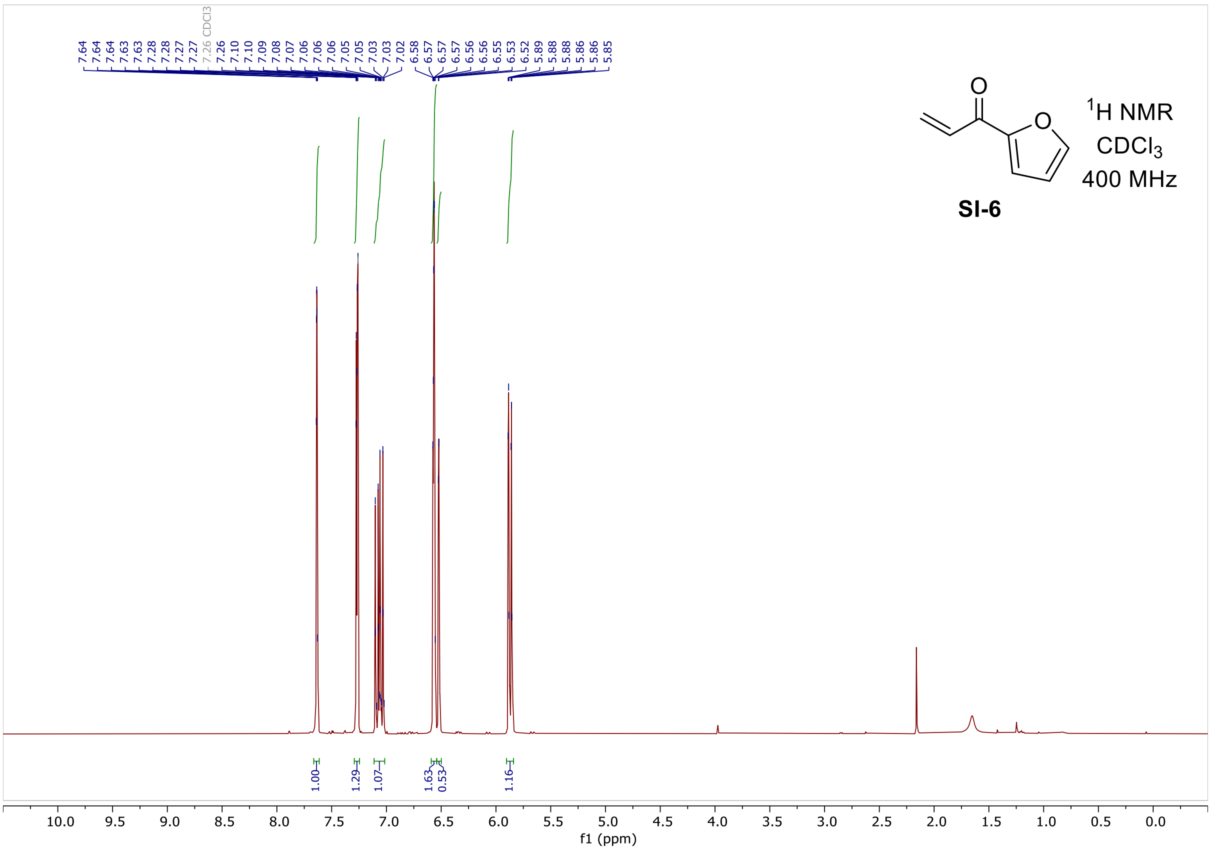

7.64
7.64
7.64
7.63
7.63
7.28
7.28
7.27
7.27
7.26 CDCl3
7.26
7.10
7.10
7.09
7.08
7.07
7.06
7.06
7.06
7.05
7.05
7.03
7.03
7.02
6.58
6.57
6.57
6.57
6.56
6.56
6.55
6.53
6.52
5.89
5.88
5.88
5.86
5.86
5.85
O
O
SI-6
^{1}H NMR
$CDCl_3$
400 MHz
1.00
1.29
1.07
1.63
0.53
1.16
10.0
9.5
9.0
8.5
8.0
7.5
7.0
6.5
6.0
5.5
5.0
4.5
4.0
3.5
3.0
2.5
2.0
1.5
1.0
0.5
0.0
f1 (ppm)

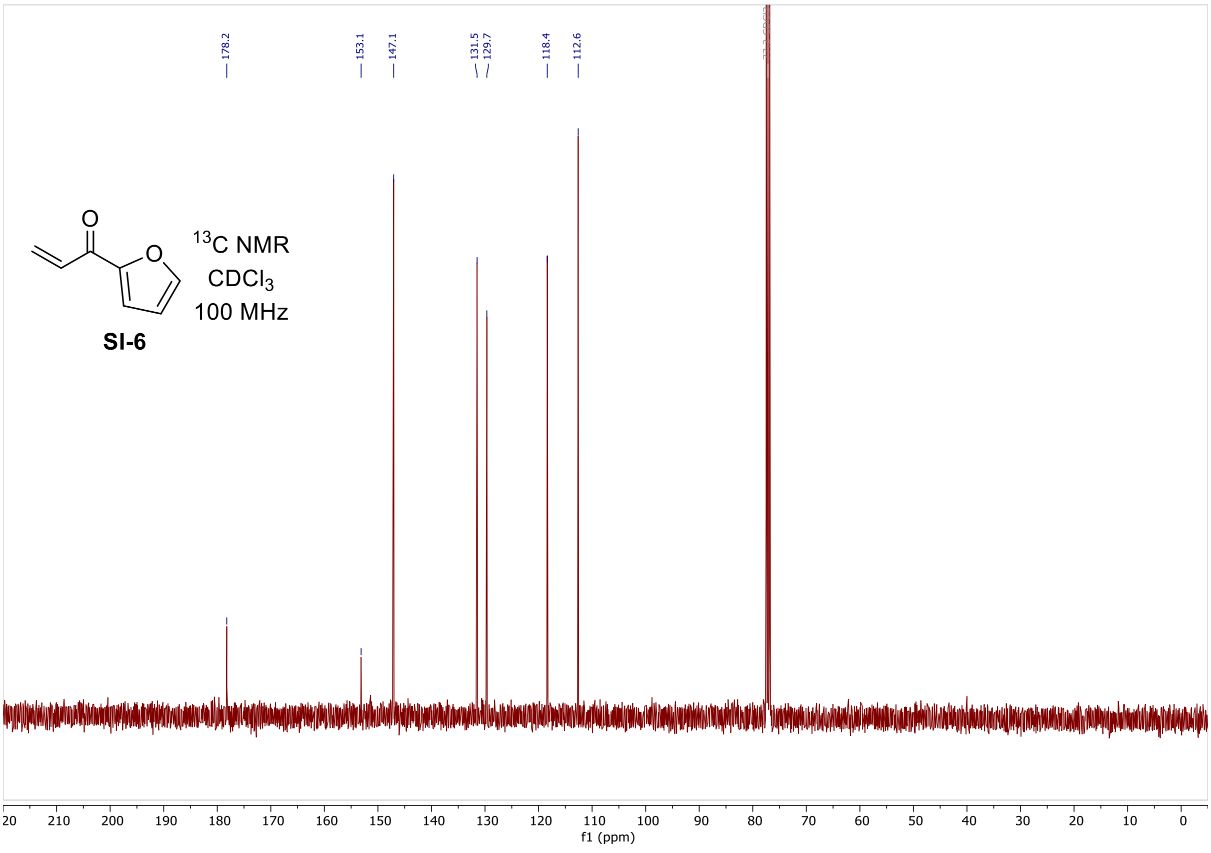

178.2
153.1
147.1
131.5
129.7
118.4
112.6
O
O
SI-6
^{13}C NMR
$CDCl_3$
100 MHz
20
210
200
190
180
170
160
150
140
130
120
110
100
90
80
70
60
50
40
30
20
10
0
f1 (ppm)

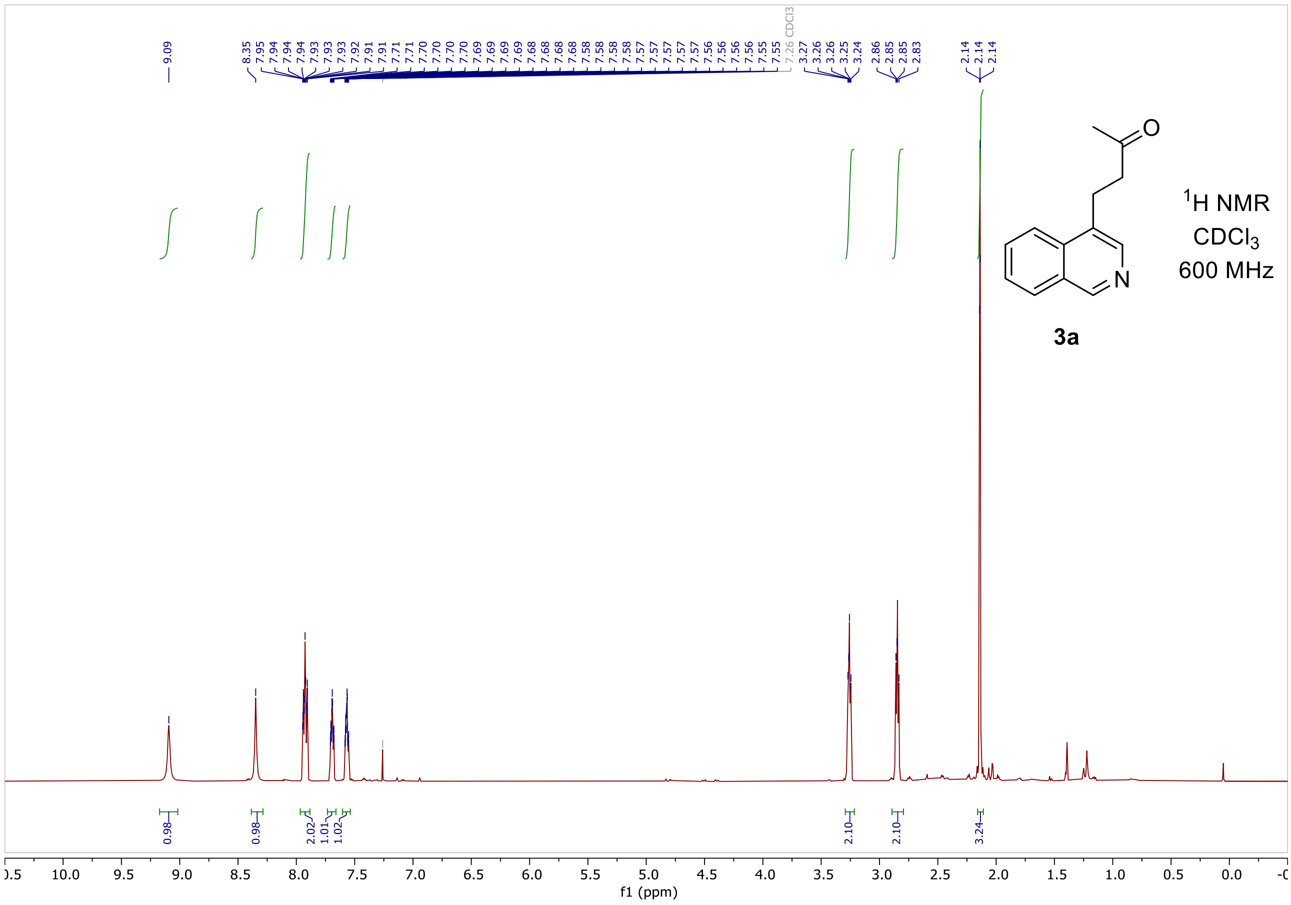
9.09
8.35 7.95 7.94 7.94 7.94 7.93 7.93 7.93 7.92 7.91 7.91 7.71 7.70 7.70 7.70 7.70 7.69 7.69 7.69 7.69 7.68 7.68 7.68 7.68 7.58 7.58 7.58 7.58 7.57 7.57 7.57 7.57 7.57 7.56 7.56 7.56 7.56 7.55 7.55
7.26 CDCl3
3.27 3.26 3.26 3.25 3.24
2.86 2.85 2.85 2.83
2.14 2.14 2.14
O
N
3a
1H NMR
CDCl3
600 MHz
0.98
0.98
2.02
1.01
1.02
2.10
2.10
3.24
f1 (ppm)

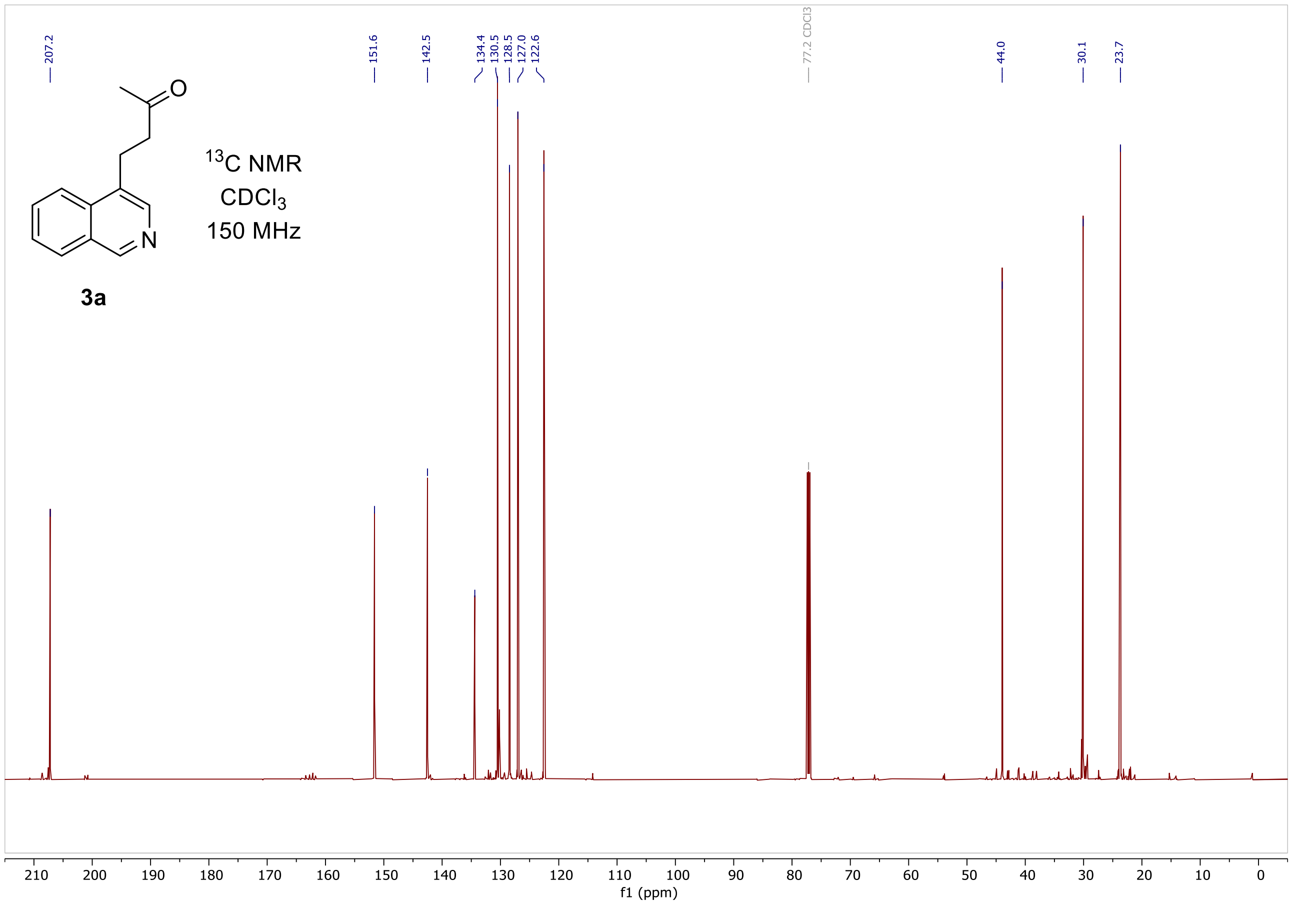
207.2
151.6
142.5
134.4 130.5 128.5 127.0 122.6
77.2 CDCl3
44.0
30.1
23.7
O
N
3a
13C NMR
CDCl3
150 MHz
f1 (ppm)

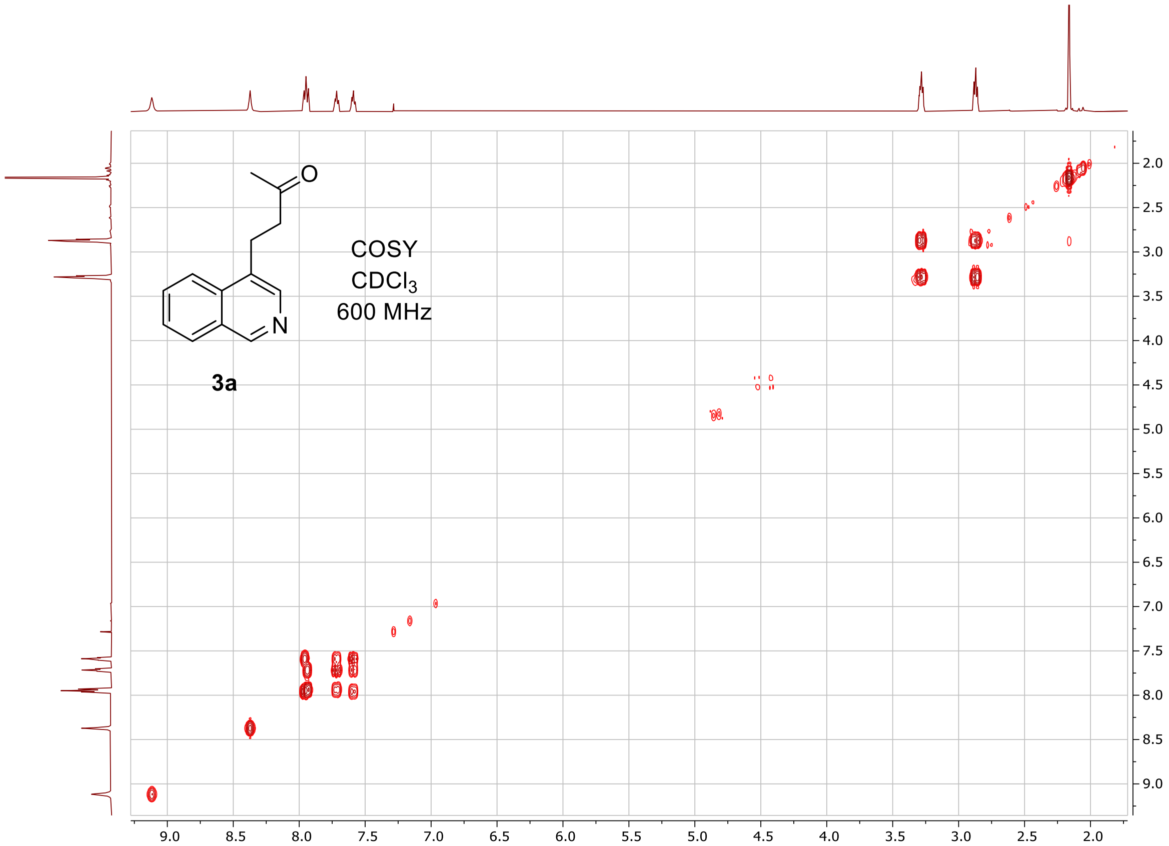

O
N
3a
COSY
CDCl3
600 MHz

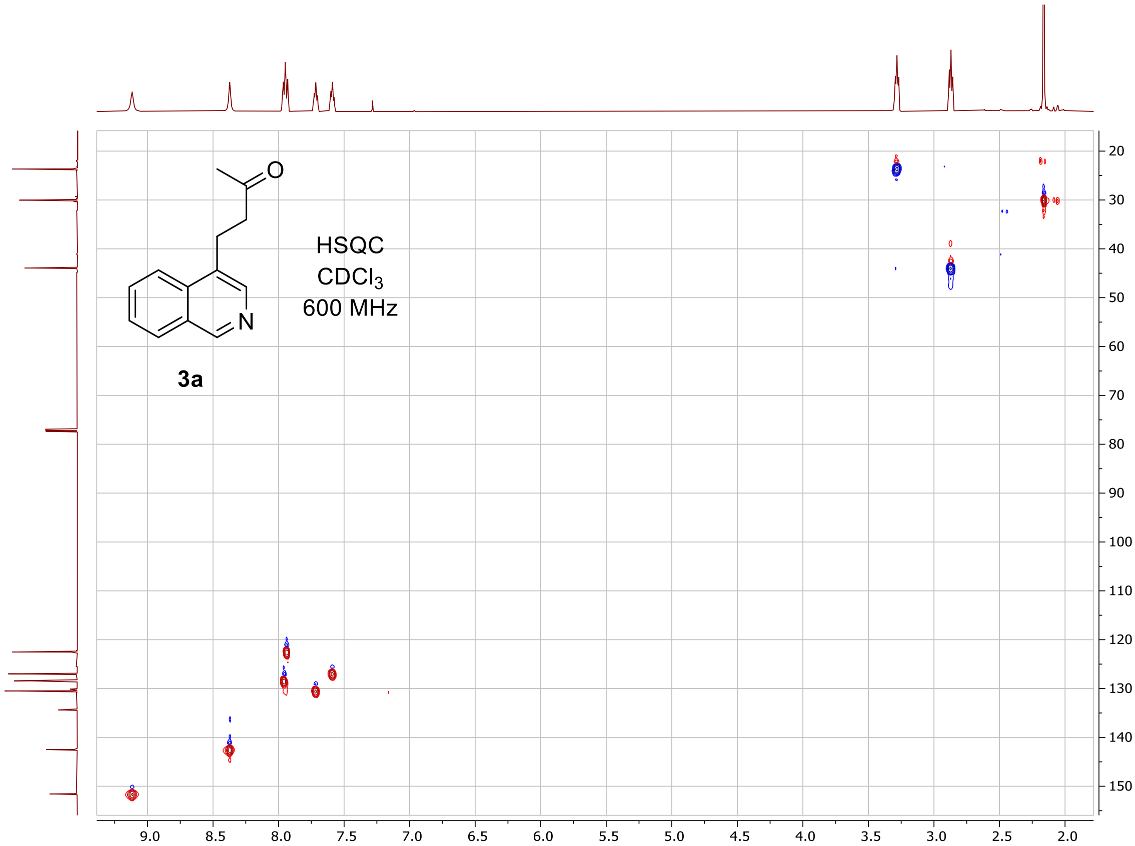

O
N
3a
HSQC
CDCl3
600 MHz

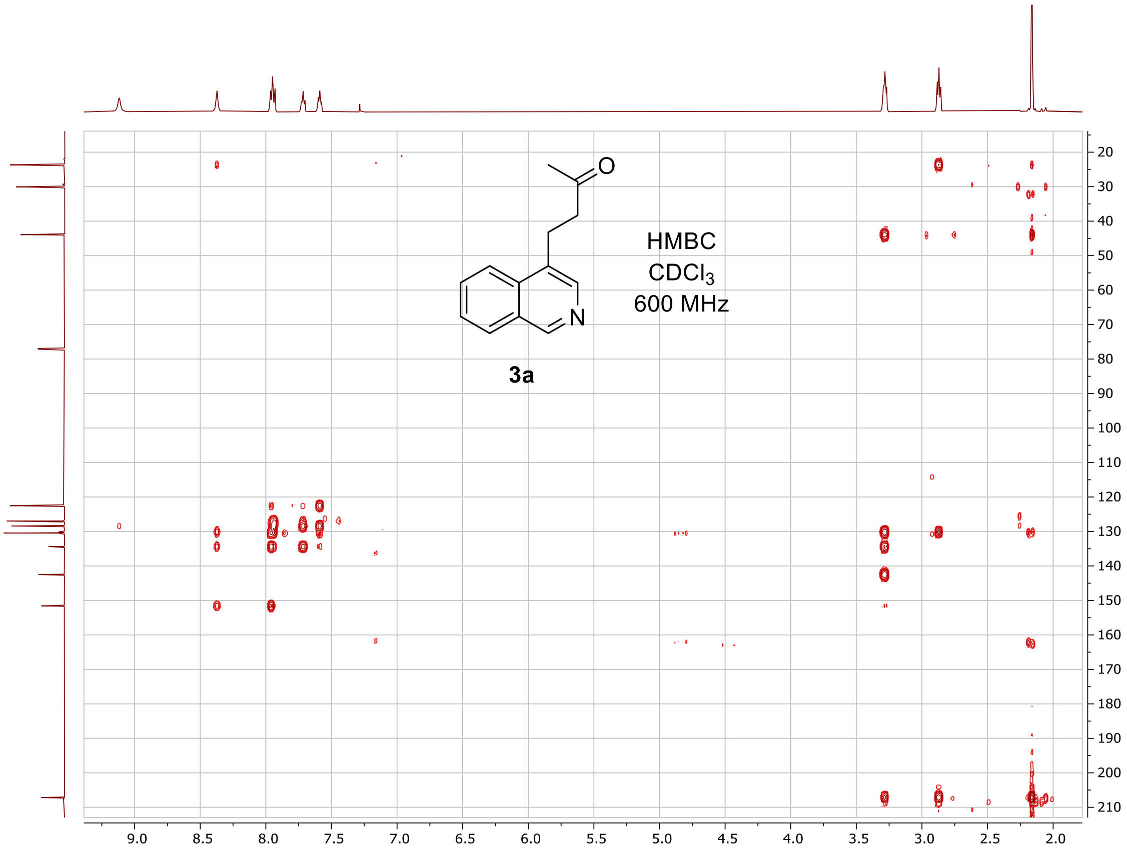
HMBC
CDCl3
600 MHz
3a

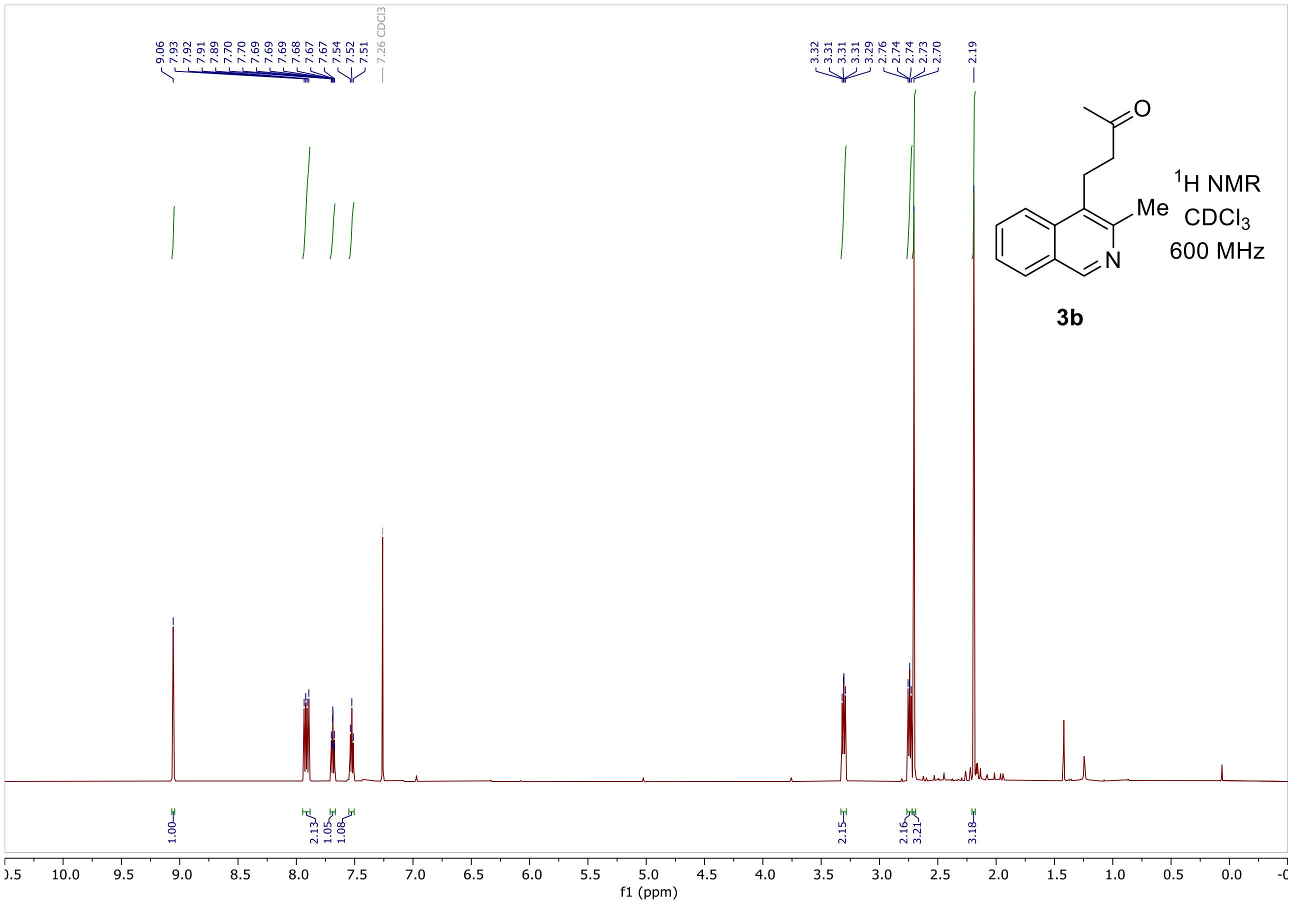

1H NMR
CDCl3
600 MHz
3b
f1 (ppm)

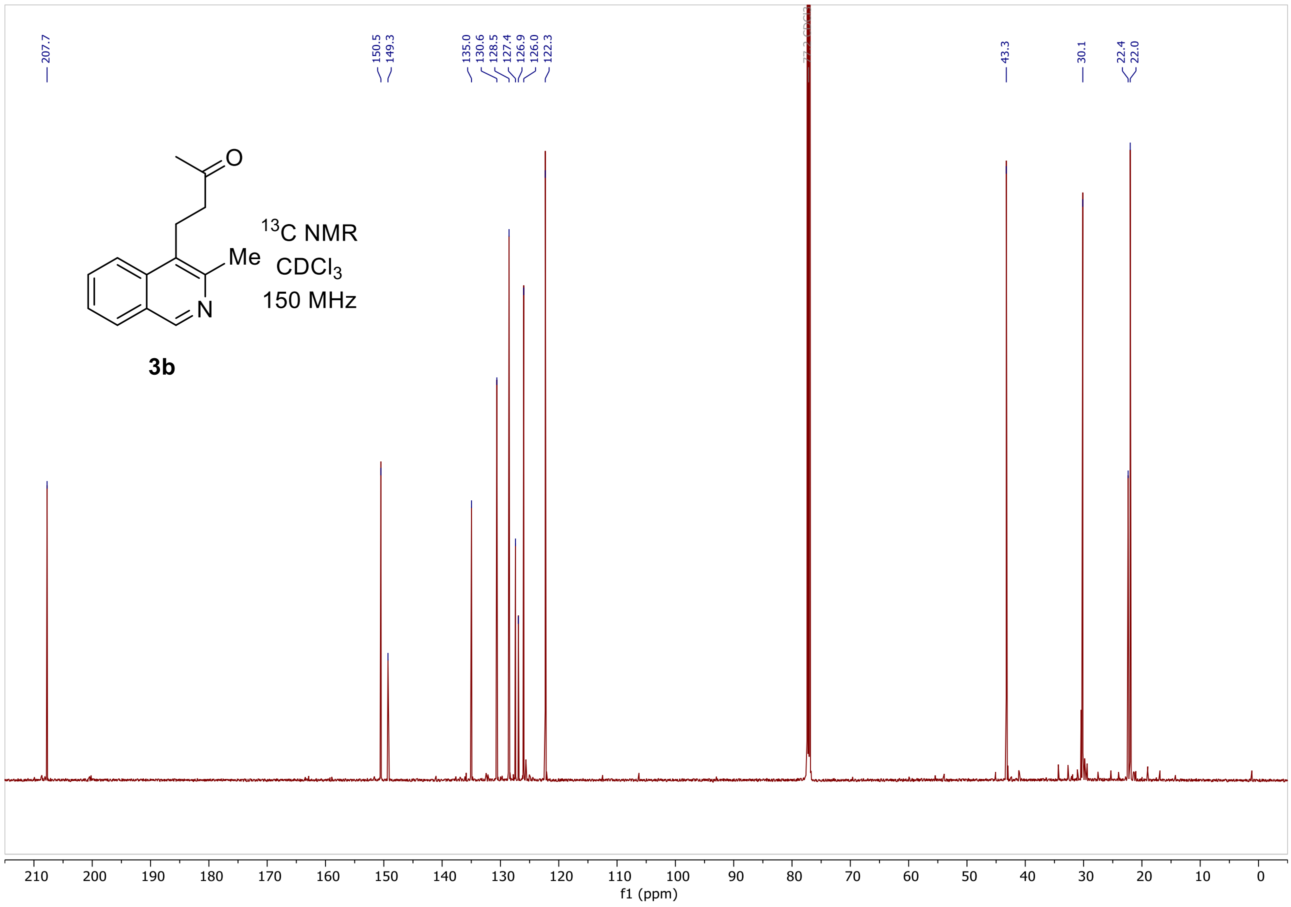

13C NMR
CDCl3
150 MHz
3b
f1 (ppm)

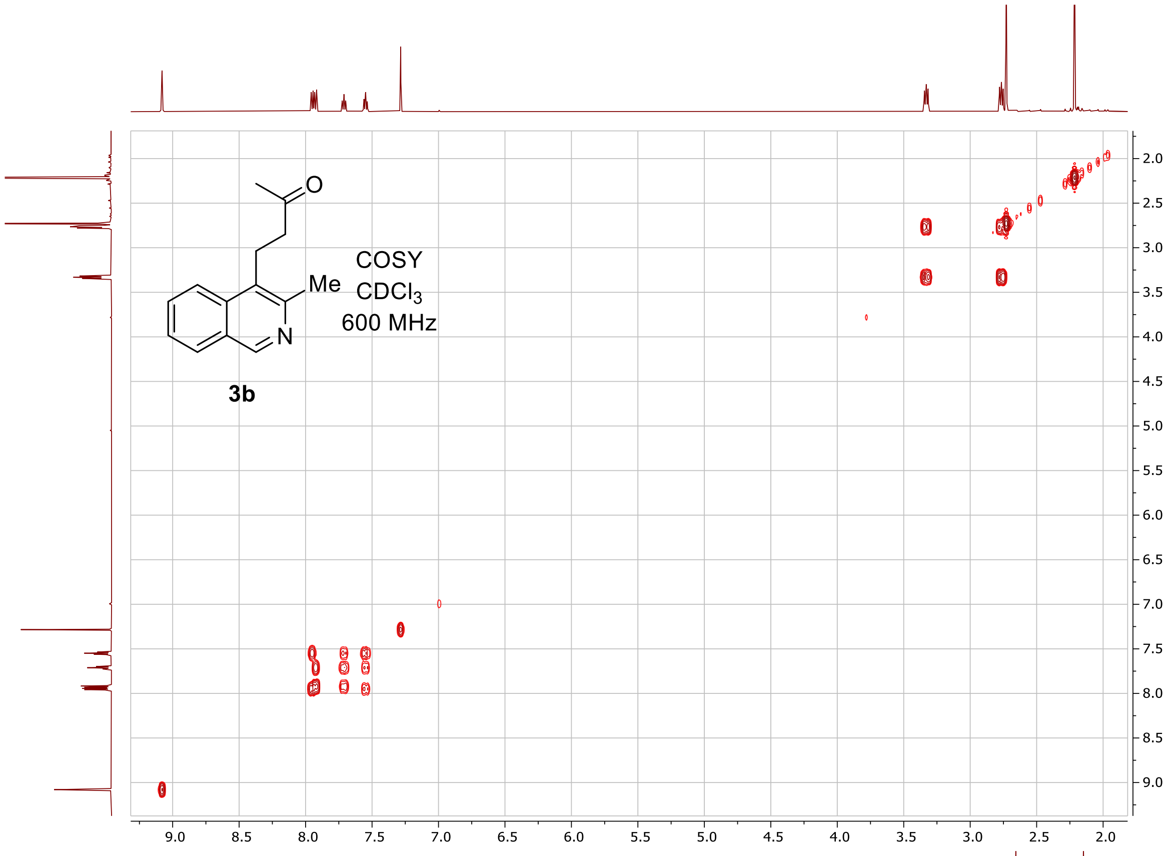
O
Me
N
3b
COSY
CDCl3
600 MHz

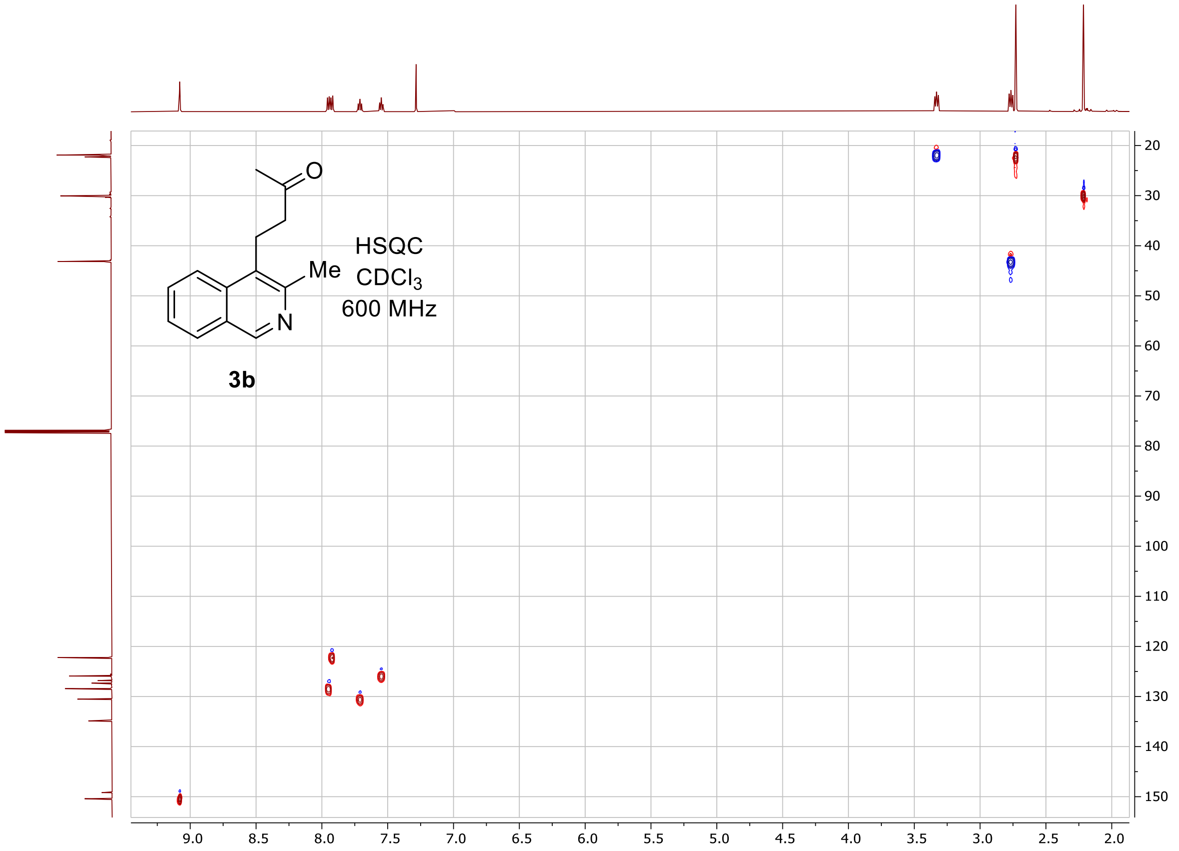
O
Me
N
3b
HSQC
CDCl3
600 MHz

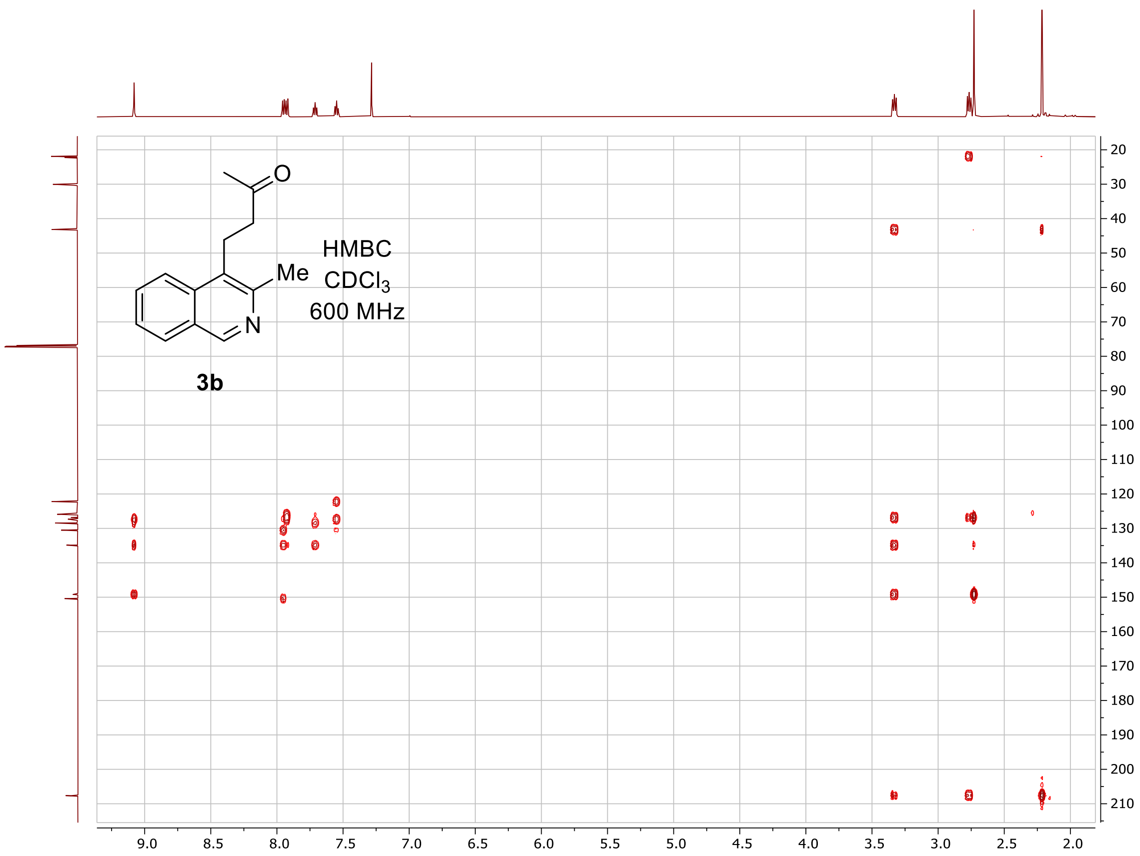
O
Me
N
3b
HMBC
$CDCl_3$
600 MHz

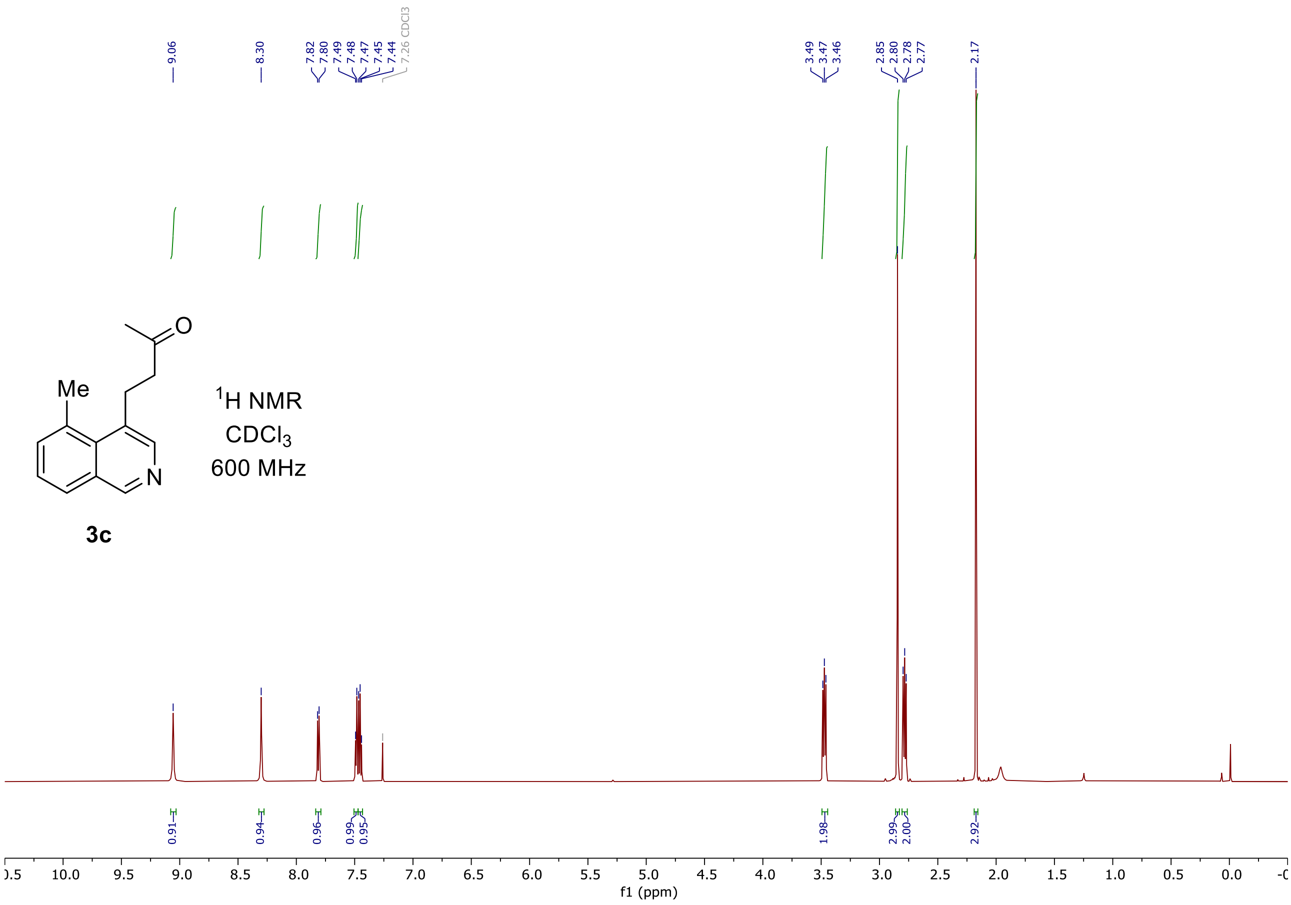
9.06
8.30
7.82 7.80 7.49 7.48 7.47 7.45 7.44
7.26 CDCl3
3.49 3.47 3.46
2.85 2.80 2.78 2.77
2.17
Me
O
N
3c
1H NMR
CDCl3
600 MHz
0.91 0.94 0.96 0.99 0.95 1.98 2.99 2.00 2.92
f1 (ppm)

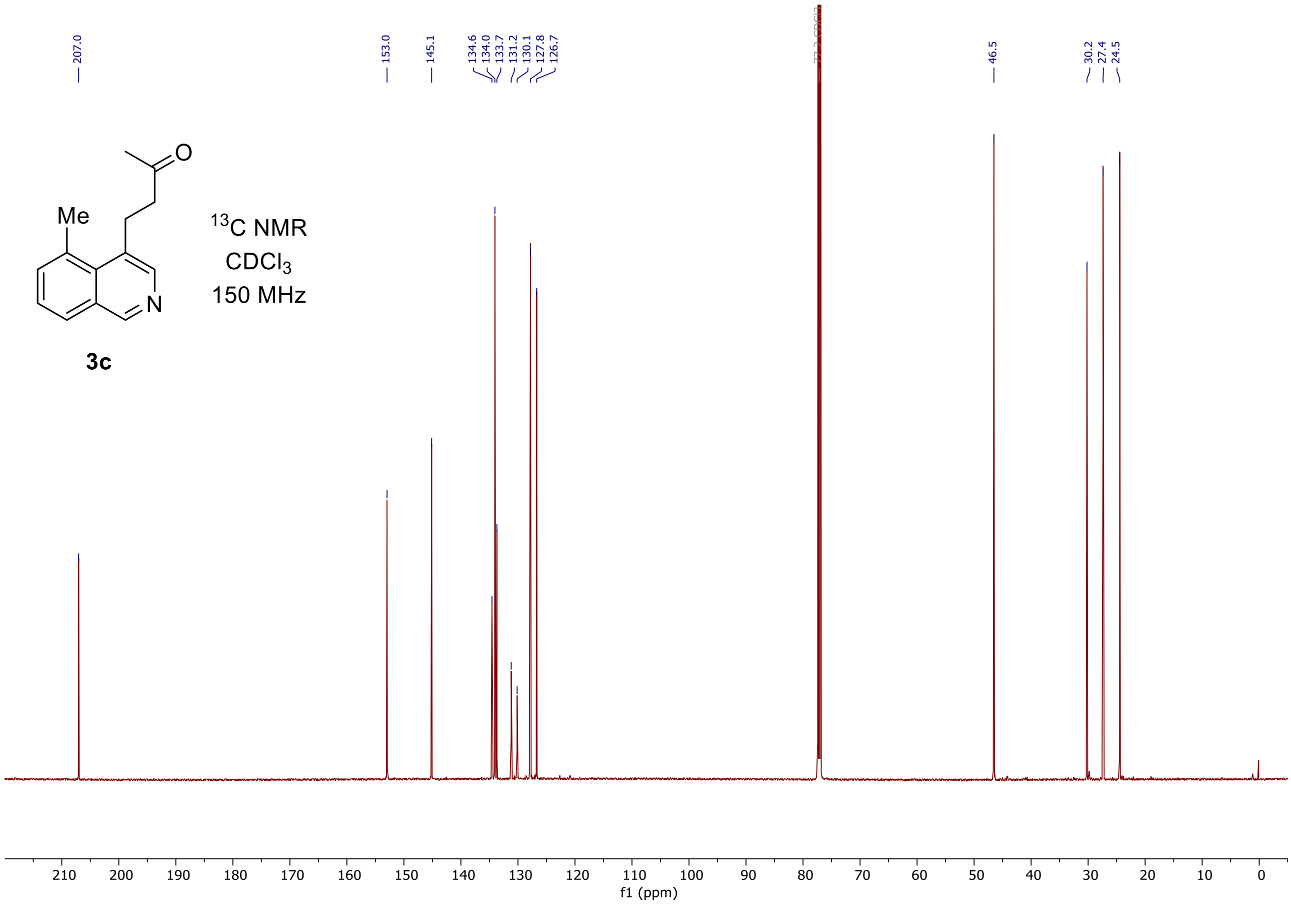
207.0
153.0
145.1
134.6 134.0 133.7 131.2 130.1 127.8 126.7
46.5
30.2 27.4 24.5
Me
O
N
3c
13C NMR
CDCl3
150 MHz
f1 (ppm)

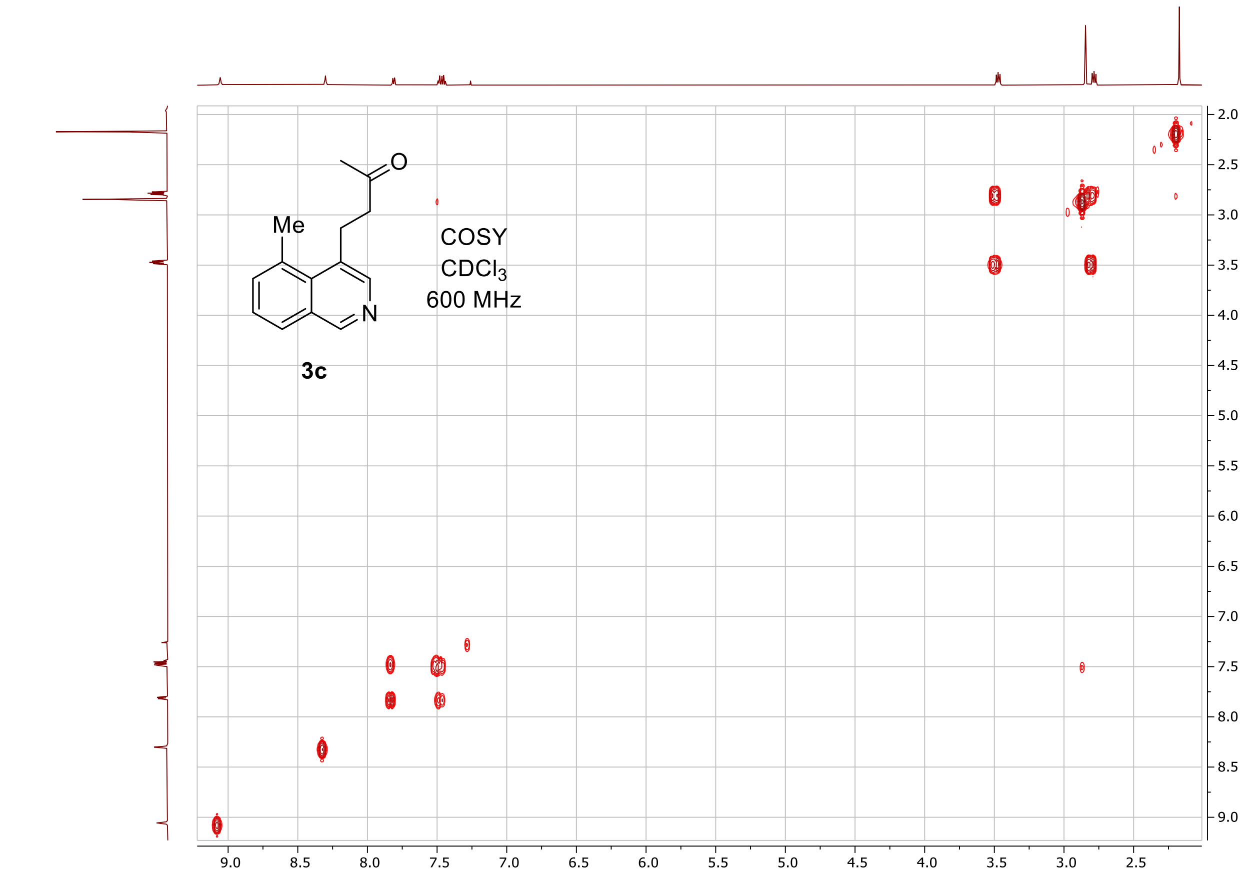
Me
O
N
3c
COSY
$CDCl_3$
600 MHz

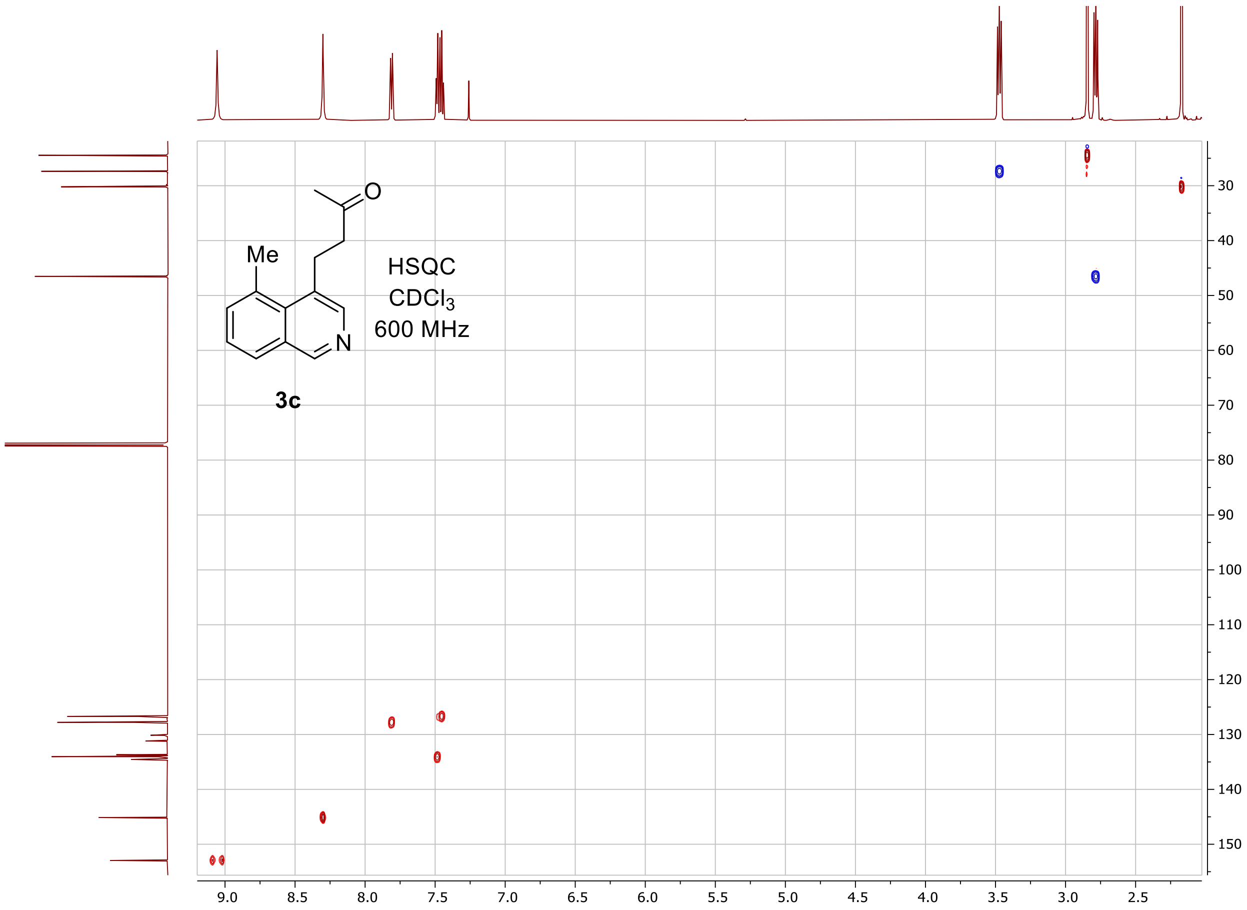
Me
O
N
3c
HSQC
$CDCl_3$
600 MHz

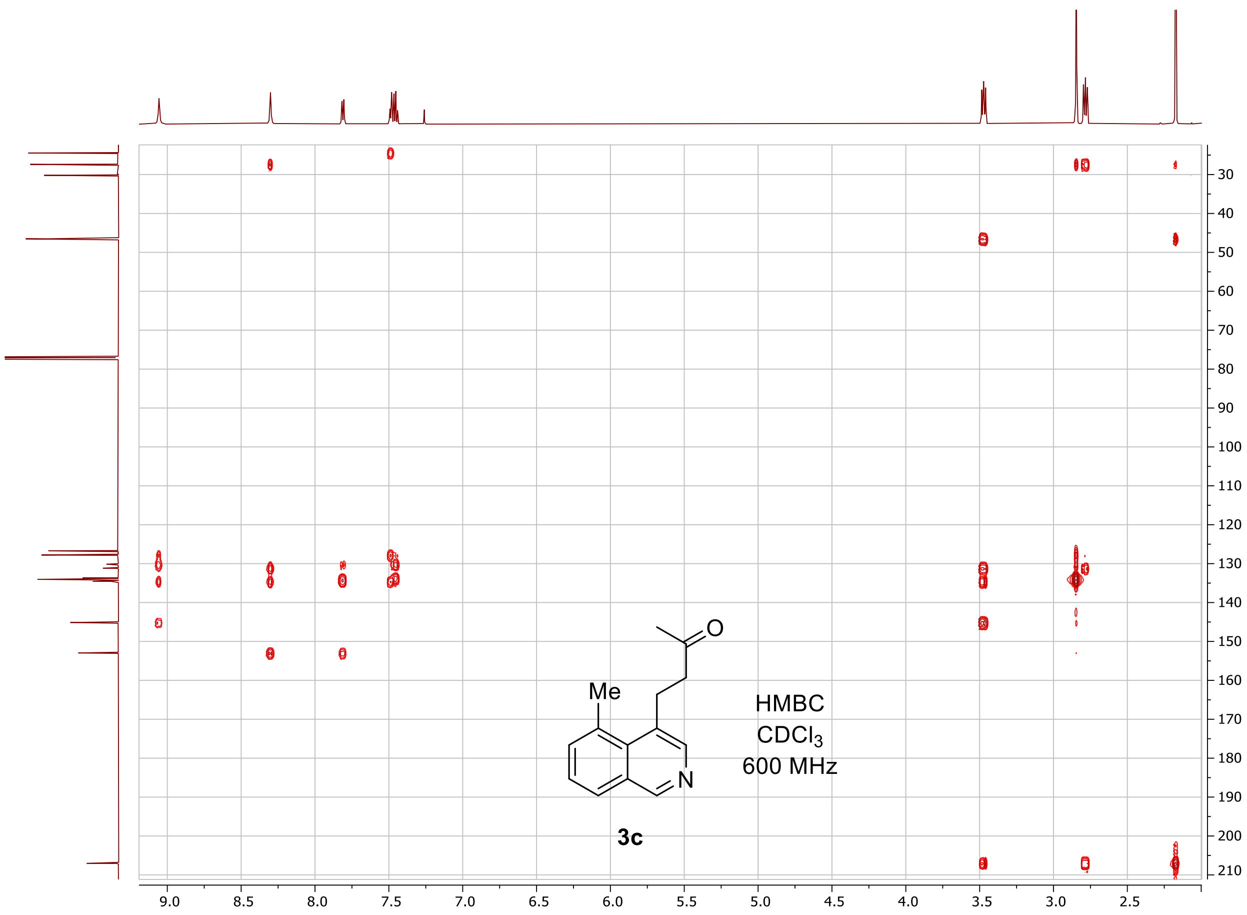

HMBC
CDCl3
600 MHz
Me
O
N
3c

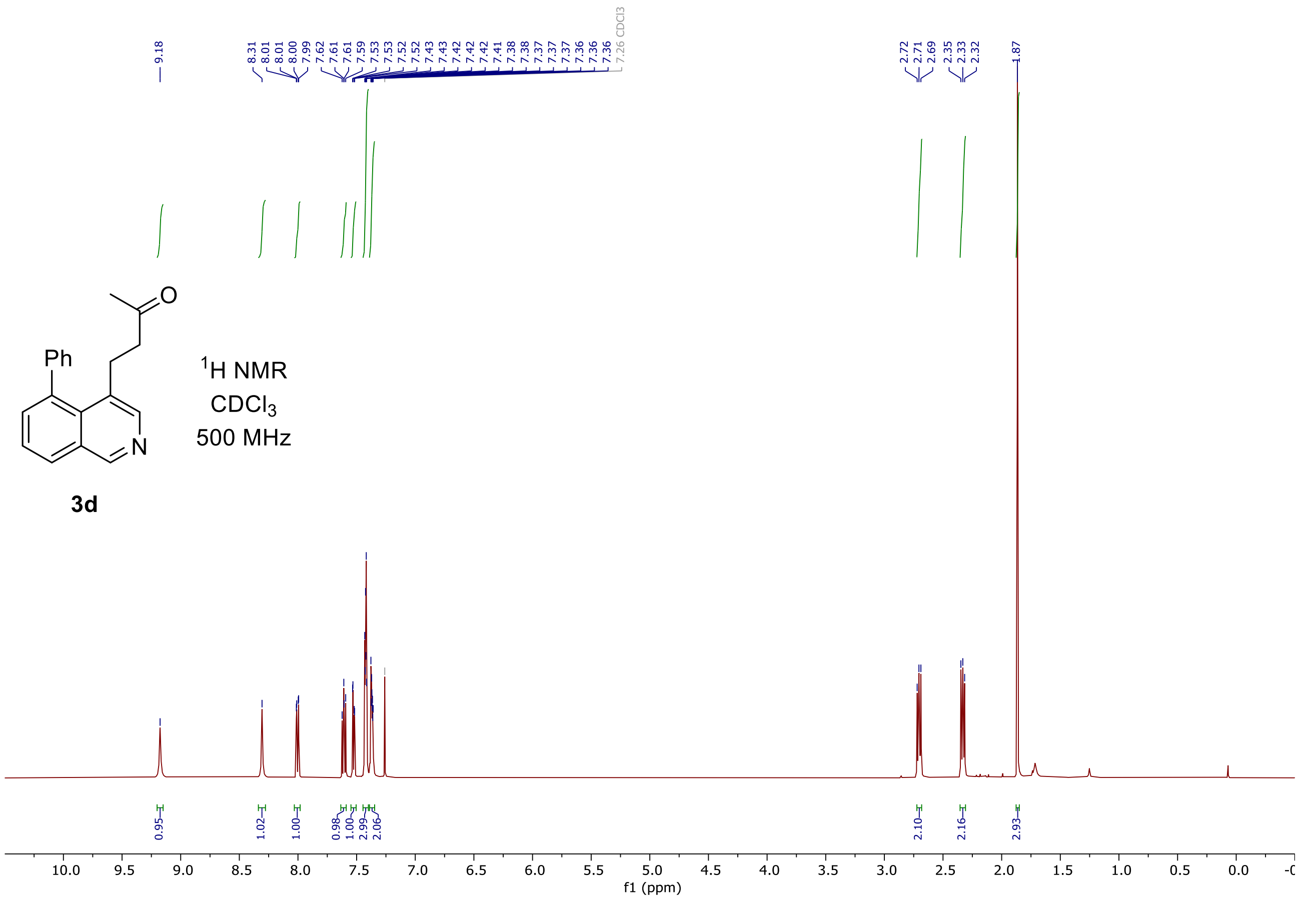
Ph
O
N
3d
1H NMR
CDCl3
500 MHz
9.18
8.31
8.01
8.01
8.00
7.99
7.62
7.61
7.61
7.59
7.53
7.53
7.52
7.52
7.43
7.43
7.42
7.42
7.42
7.41
7.38
7.38
7.37
7.37
7.37
7.36
7.36
7.26 CDCl3
2.72
2.71
2.69
2.35
2.33
2.32
1.87
0.95
1.02
1.00
0.98
1.00
2.99
2.06
2.10
2.16
2.93
f1 (ppm)

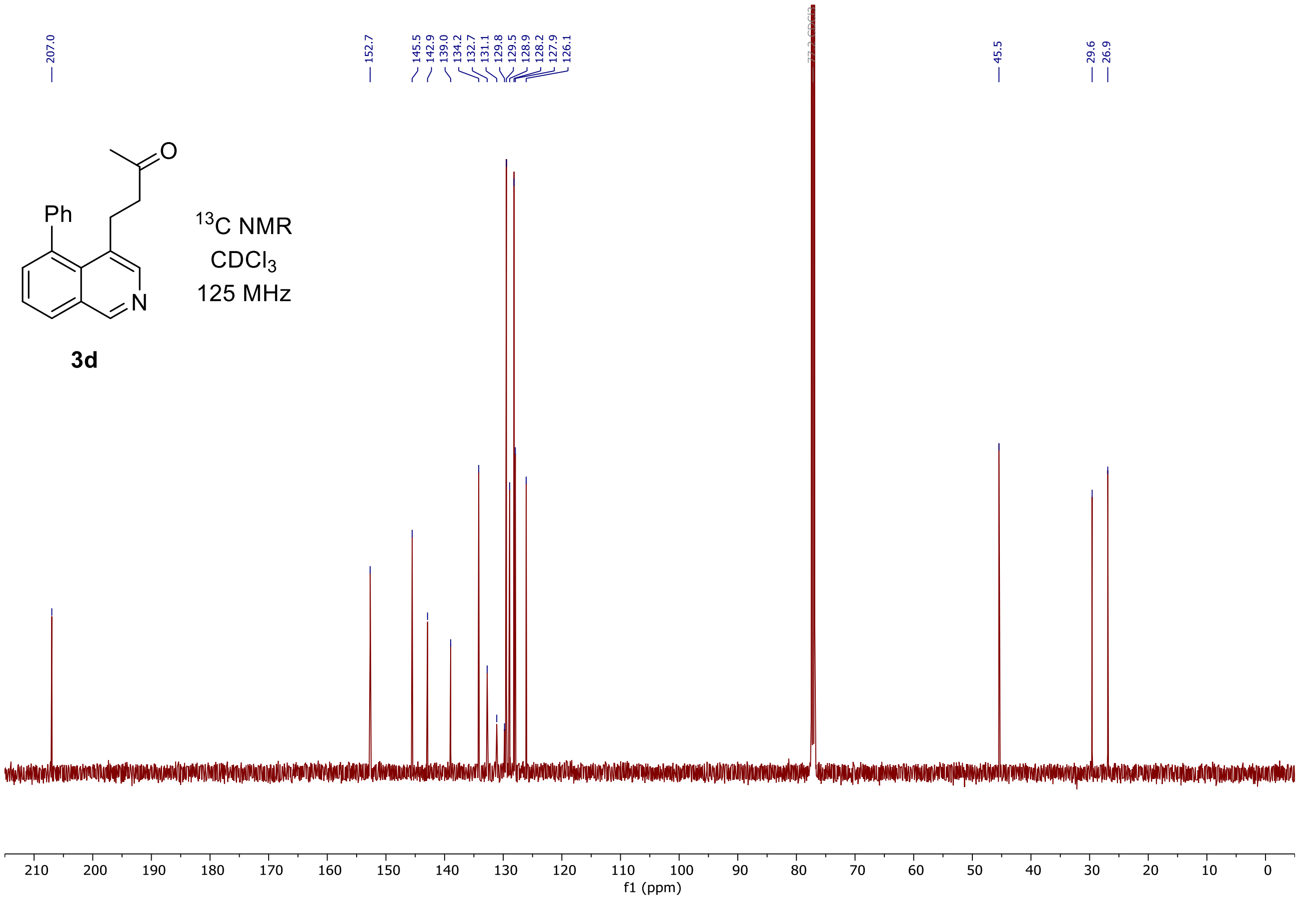
Ph
O
N
3d
13C NMR
CDCl3
125 MHz
207.0
152.7
145.5
142.9
139.0
134.2
132.7
131.1
129.8
129.5
128.9
128.2
127.9
126.1
45.5
29.6
26.9
f1 (ppm)

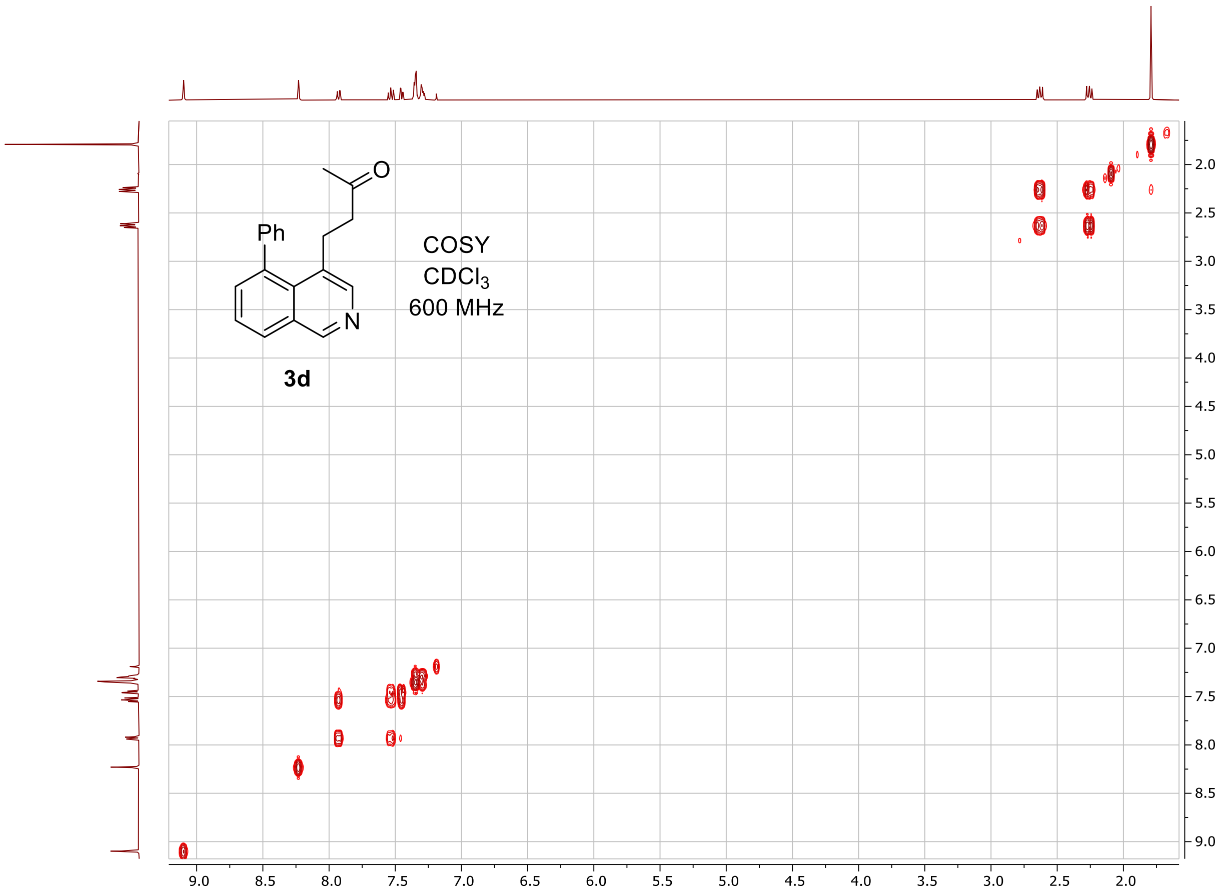
Ph
O
N
3d
COSY
$CDCl_3$
600 MHz

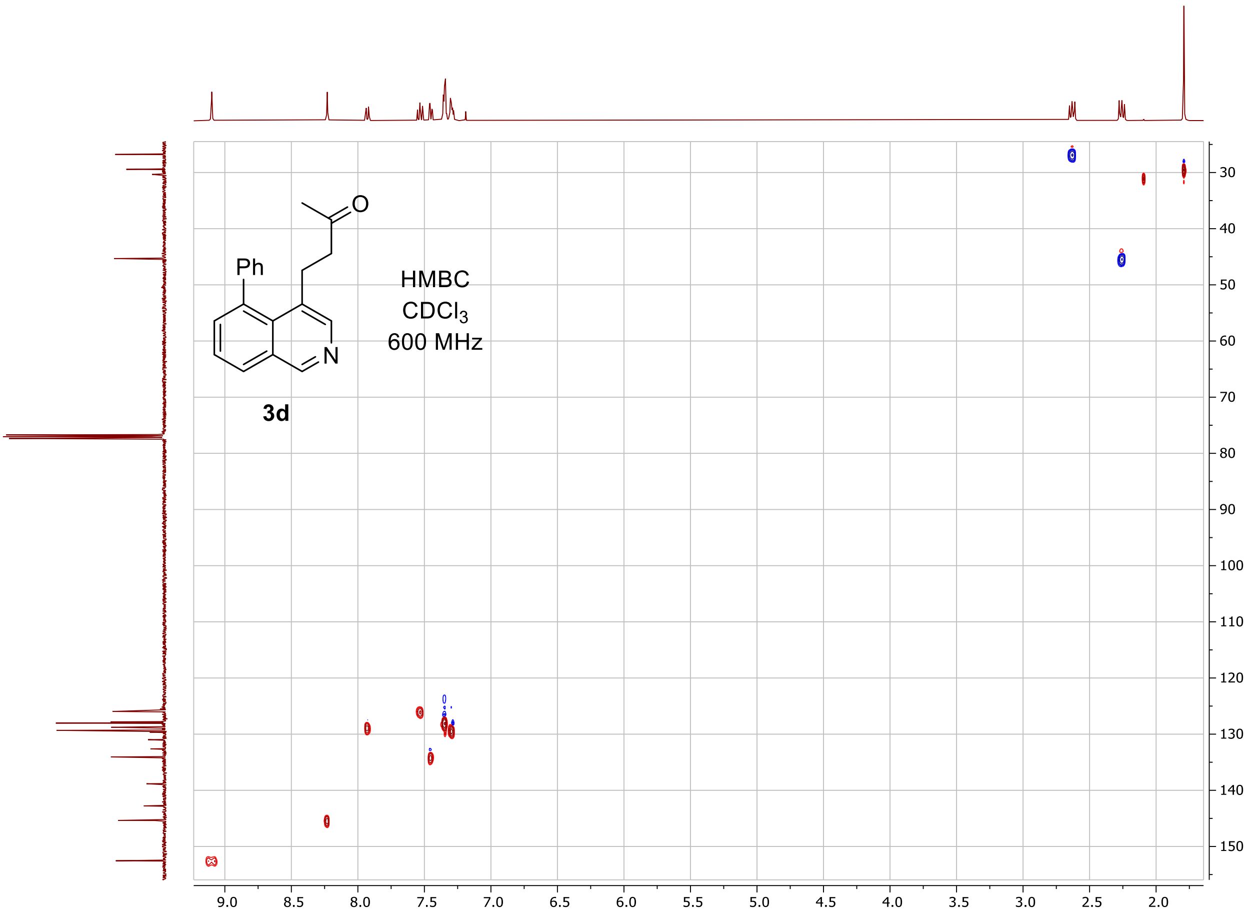
Ph
O
N
3d
HMBC
$CDCl_3$
600 MHz

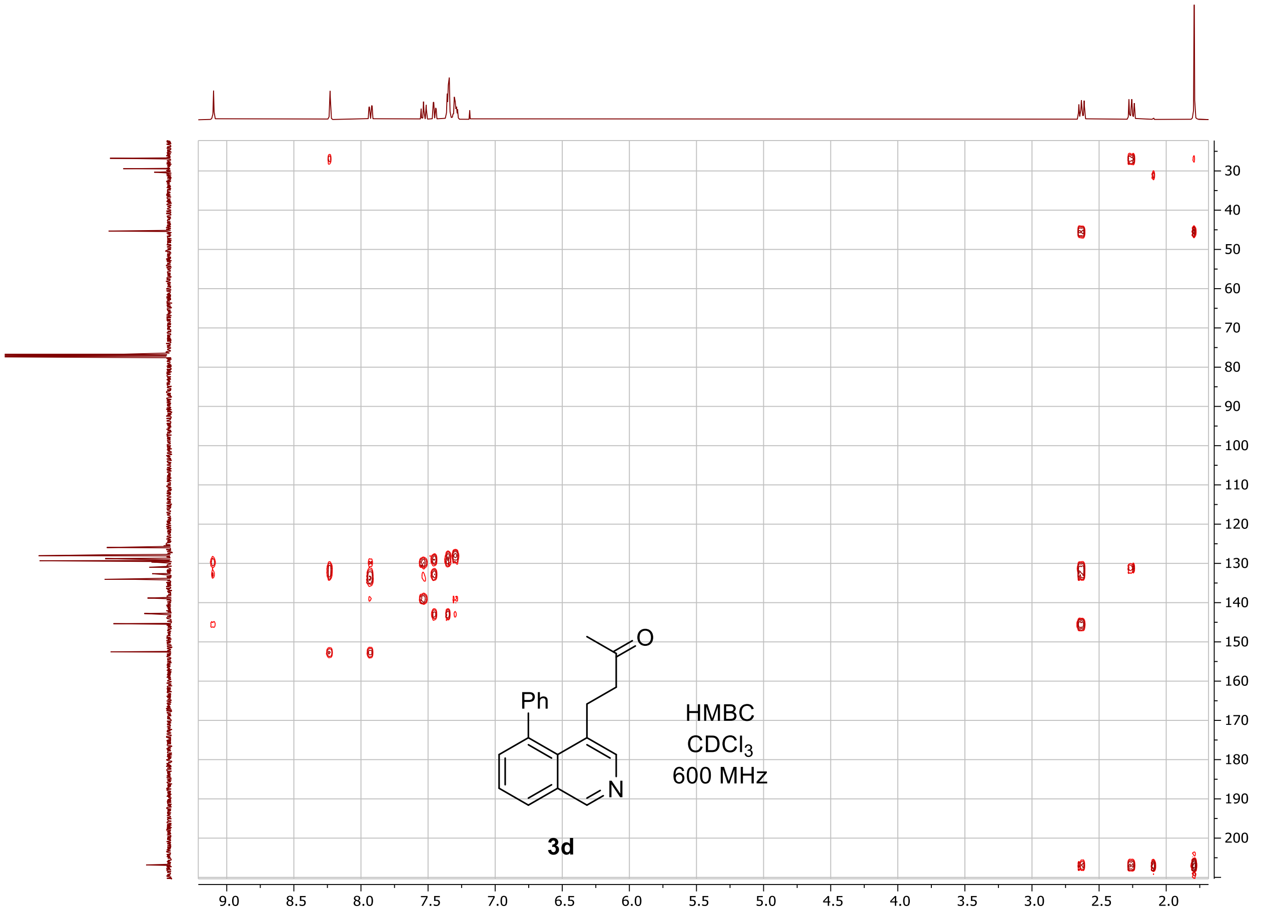

Ph
O
N
3d
HMBC
$CDCl_3$
600 MHz

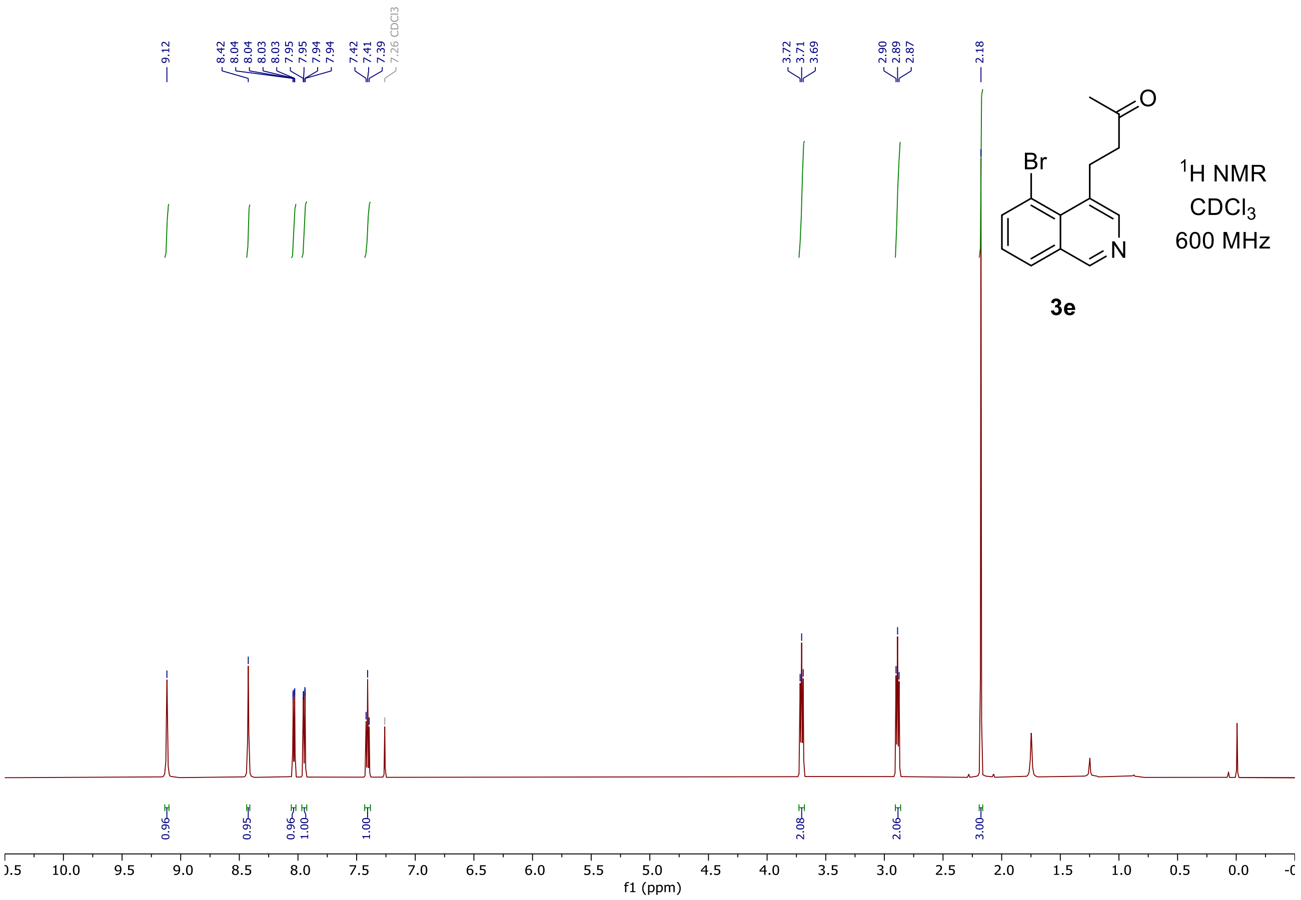

9.12
8.42 8.04 8.04 8.03 8.03 7.95 7.95 7.94 7.94
7.42 7.41 7.39 7.26 CDCl3
3.72 3.71 3.69
2.90 2.89 2.87
2.18
Br
O
N
3e
^{1}H NMR
$CDCl_3$
600 MHz
0.96 0.95 0.96 1.00 1.00 2.08 2.06 3.00
f1 (ppm)

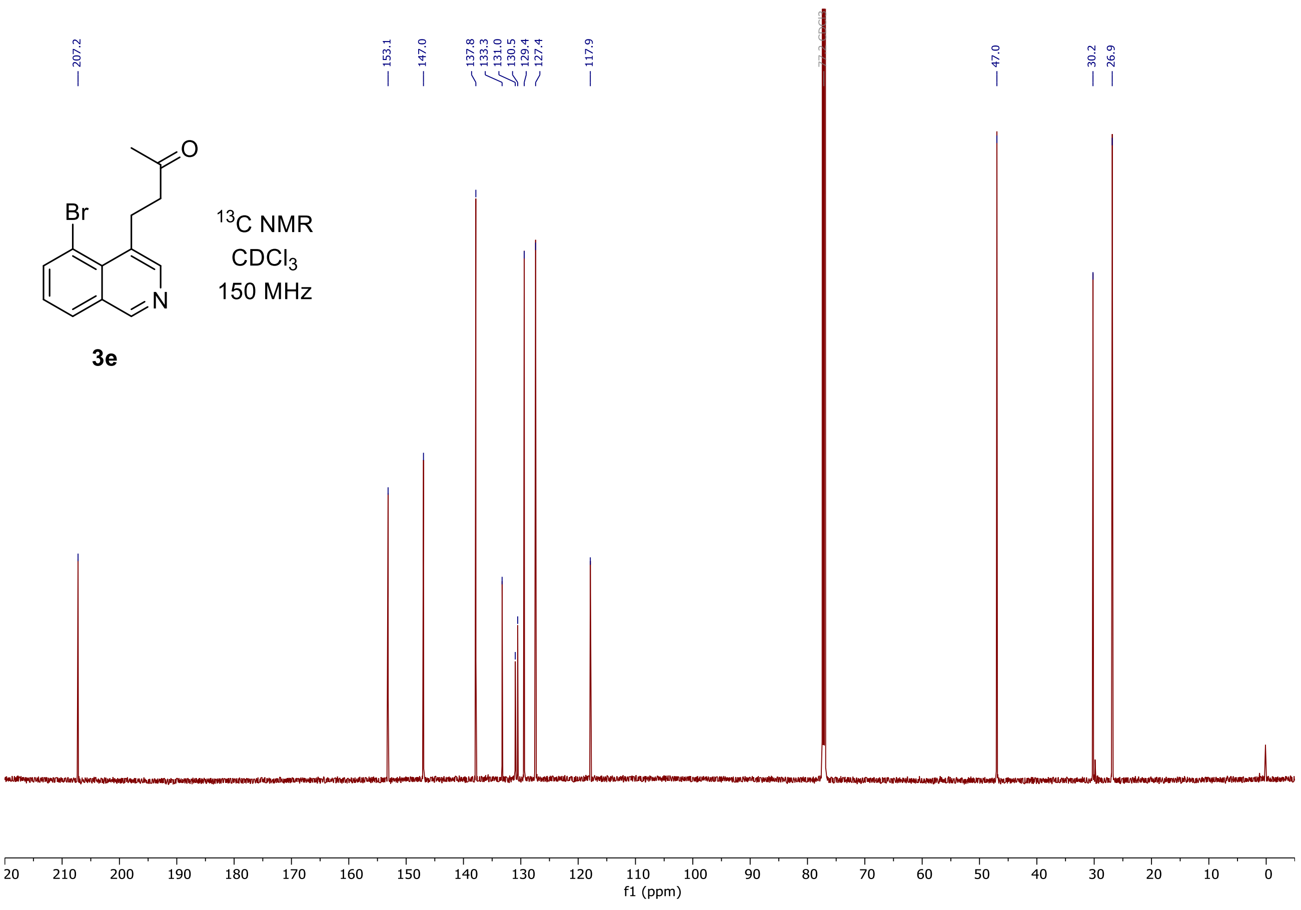

207.2
153.1
147.0
137.8 133.3 131.0 130.5 129.4 127.4
117.9
47.0
30.2
26.9
Br
O
N
3e
^{13}C NMR
$CDCl_3$
150 MHz
f1 (ppm)

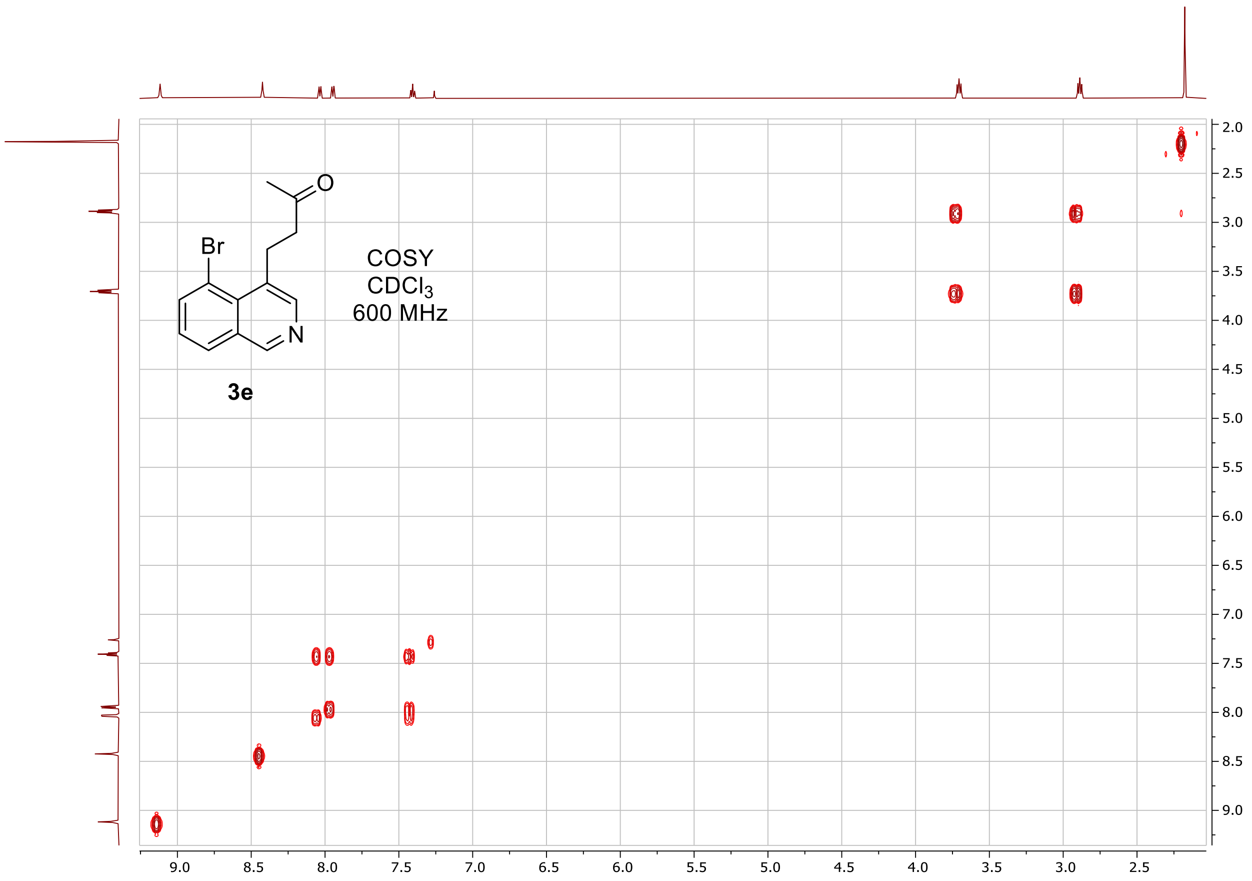
Br
O
N
3e
COSY
$CDCl_3$
600 MHz
9.0 8.5 8.0 7.5 7.0 6.5 6.0 5.5 5.0 4.5 4.0 3.5 3.0 2.5
2.0 2.5 3.0 3.5 4.0 4.5 5.0 5.5 6.0 6.5 7.0 7.5 8.0 8.5 9.0

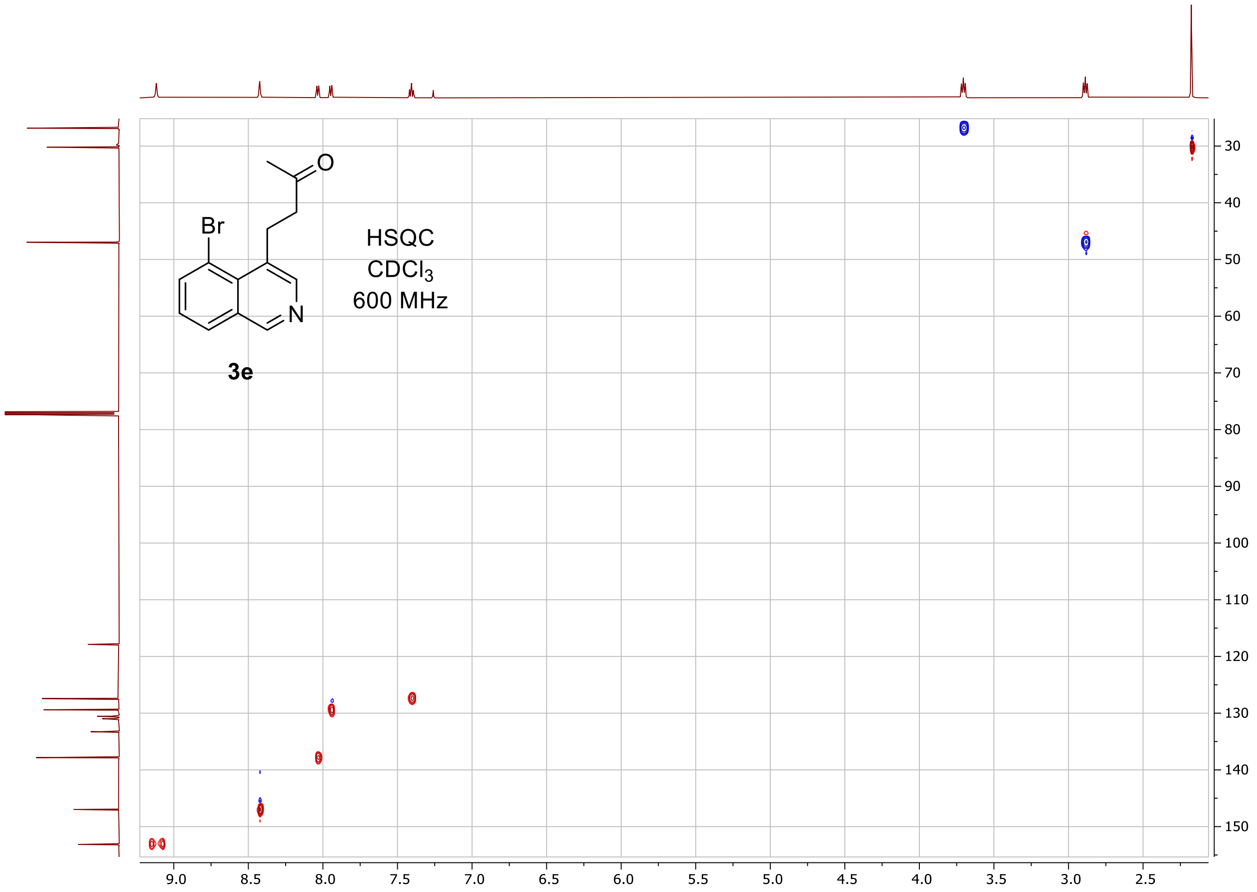
Br
O
N
3e
HSQC
$CDCl_3$
600 MHz
9.0 8.5 8.0 7.5 7.0 6.5 6.0 5.5 5.0 4.5 4.0 3.5 3.0 2.5
30 40 50 60 70 80 90 100 110 120 130 140 150

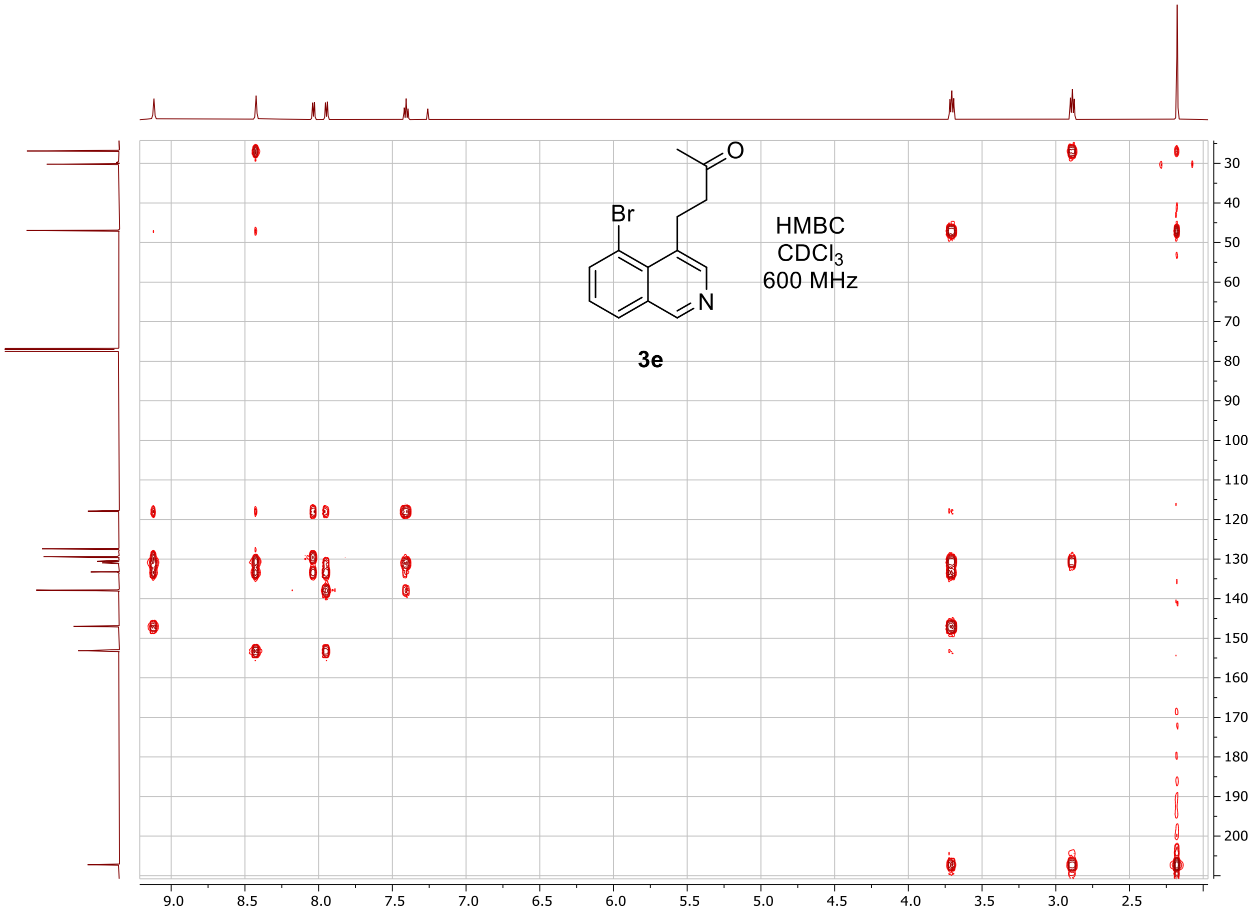
Br
O
N
3e
HMBC
CDCl3
600 MHz

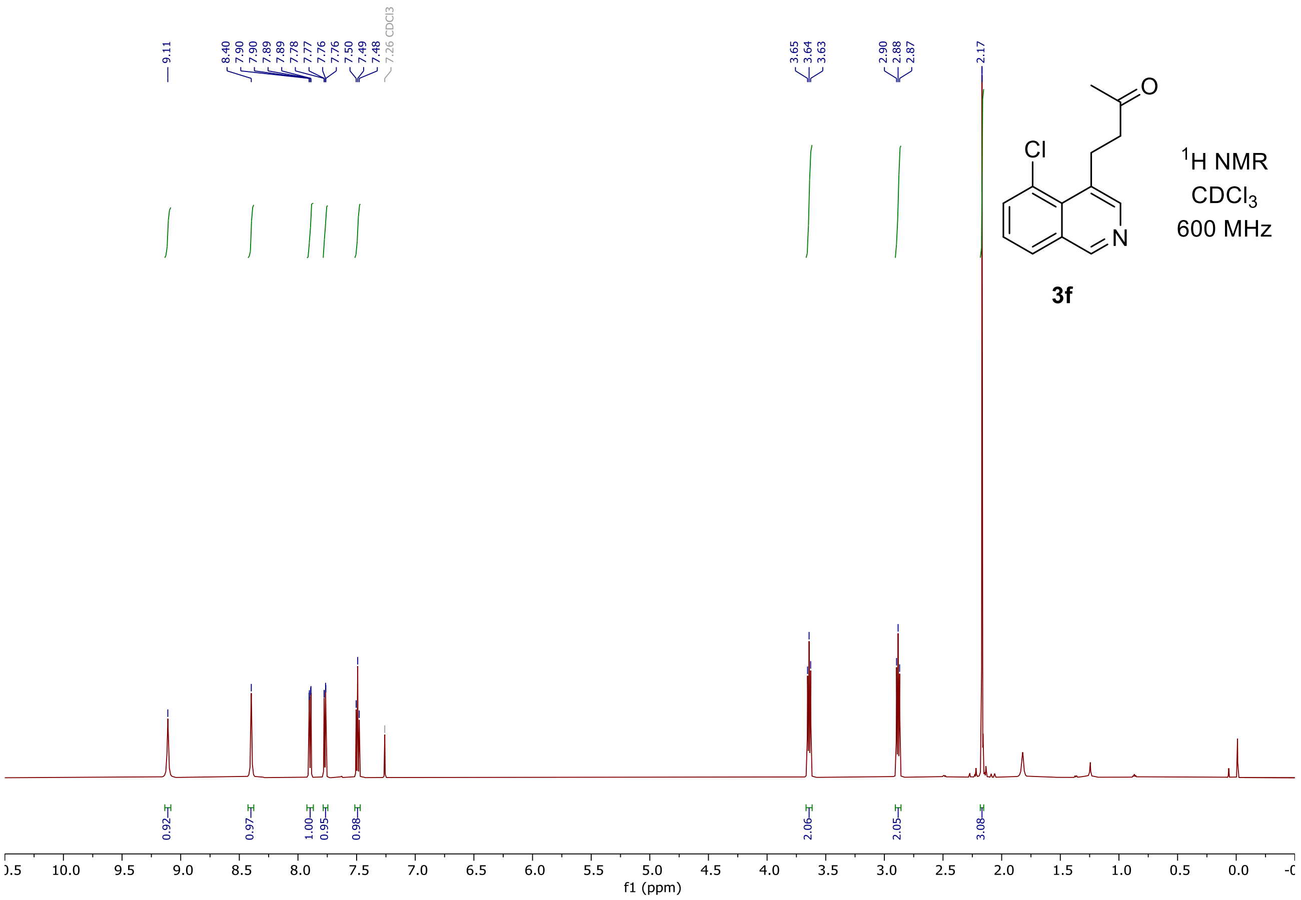

9.11
8.40
7.90
7.90
7.89
7.89
7.78
7.77
7.76
7.76
7.50
7.49
7.48
7.26 CDCl3
3.65
3.64
3.63
2.90
2.88
2.87
2.17
Cl
O
N
3f
1H NMR
CDCl3
600 MHz
0.92
0.97
1.00
0.95
0.98
2.06
2.05
3.08
f1 (ppm)

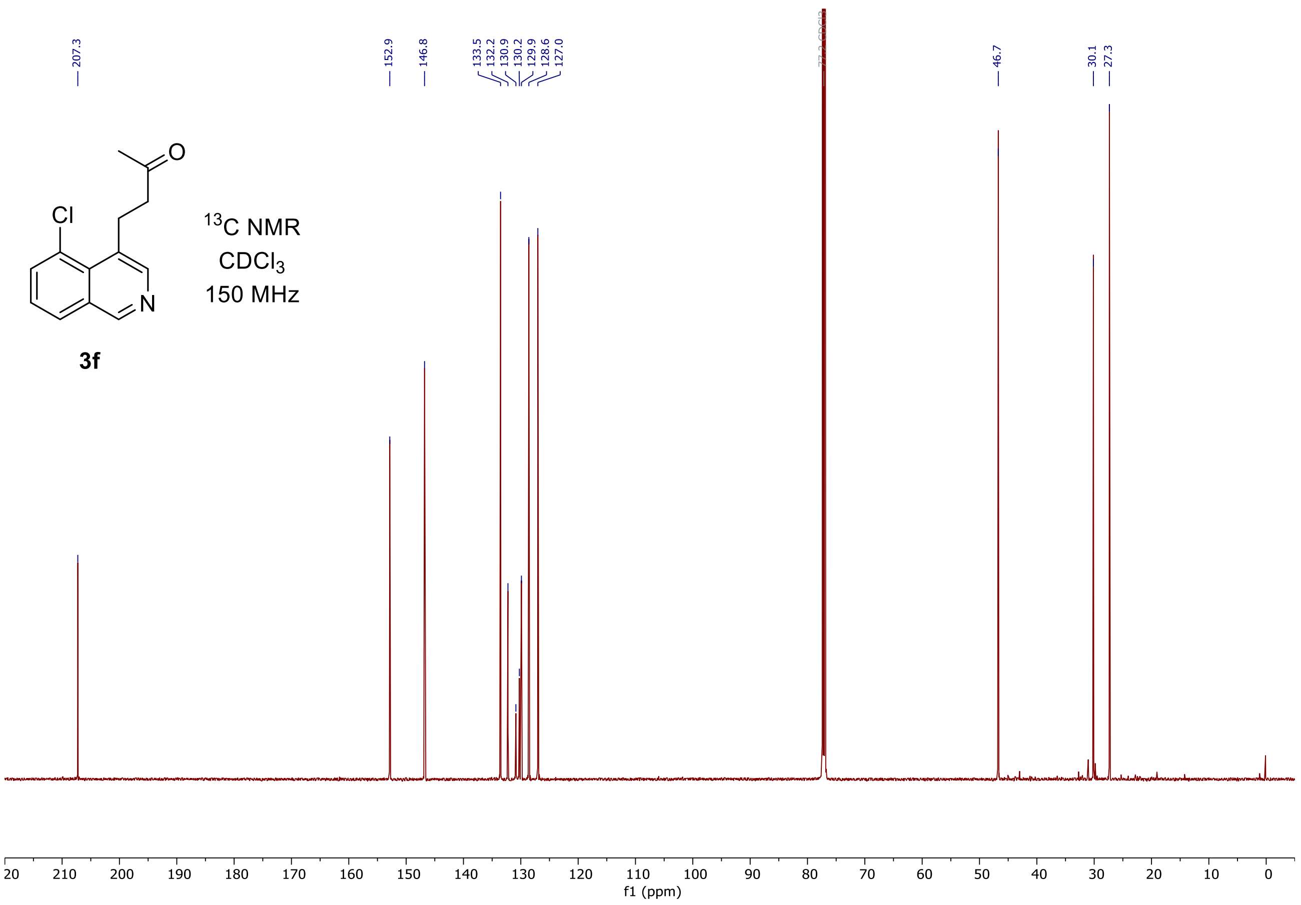

207.3
152.9
146.8
133.5
132.2
130.9
130.2
129.9
128.6
127.0
46.7
30.1
27.3
Cl
O
N
3f
13C NMR
CDCl3
150 MHz
f1 (ppm)

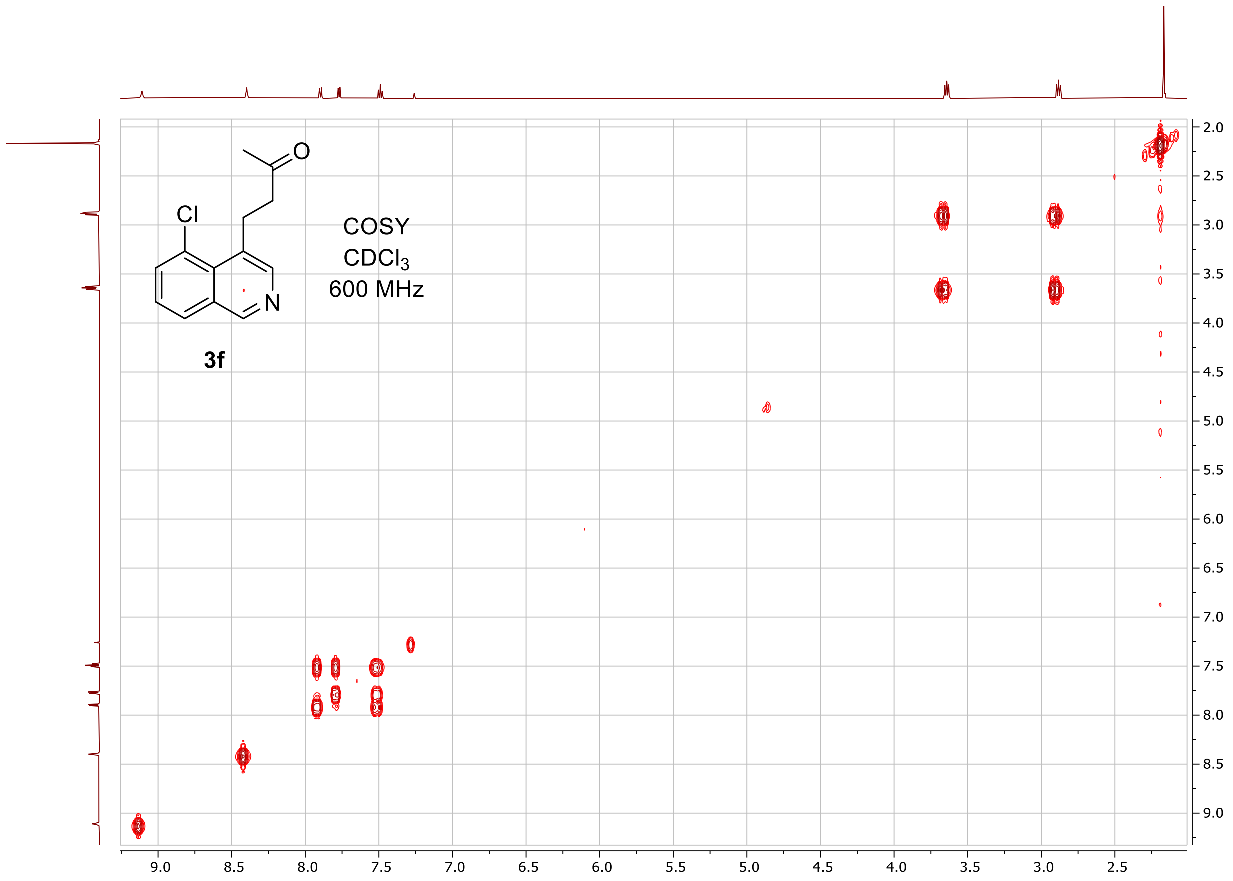
Cl
O
N
3f
COSY
$CDCl_3$
600 MHz
9.0 8.5 8.0 7.5 7.0 6.5 6.0 5.5 5.0 4.5 4.0 3.5 3.0 2.5
2.0 2.5 3.0 3.5 4.0 4.5 5.0 5.5 6.0 6.5 7.0 7.5 8.0 8.5 9.0

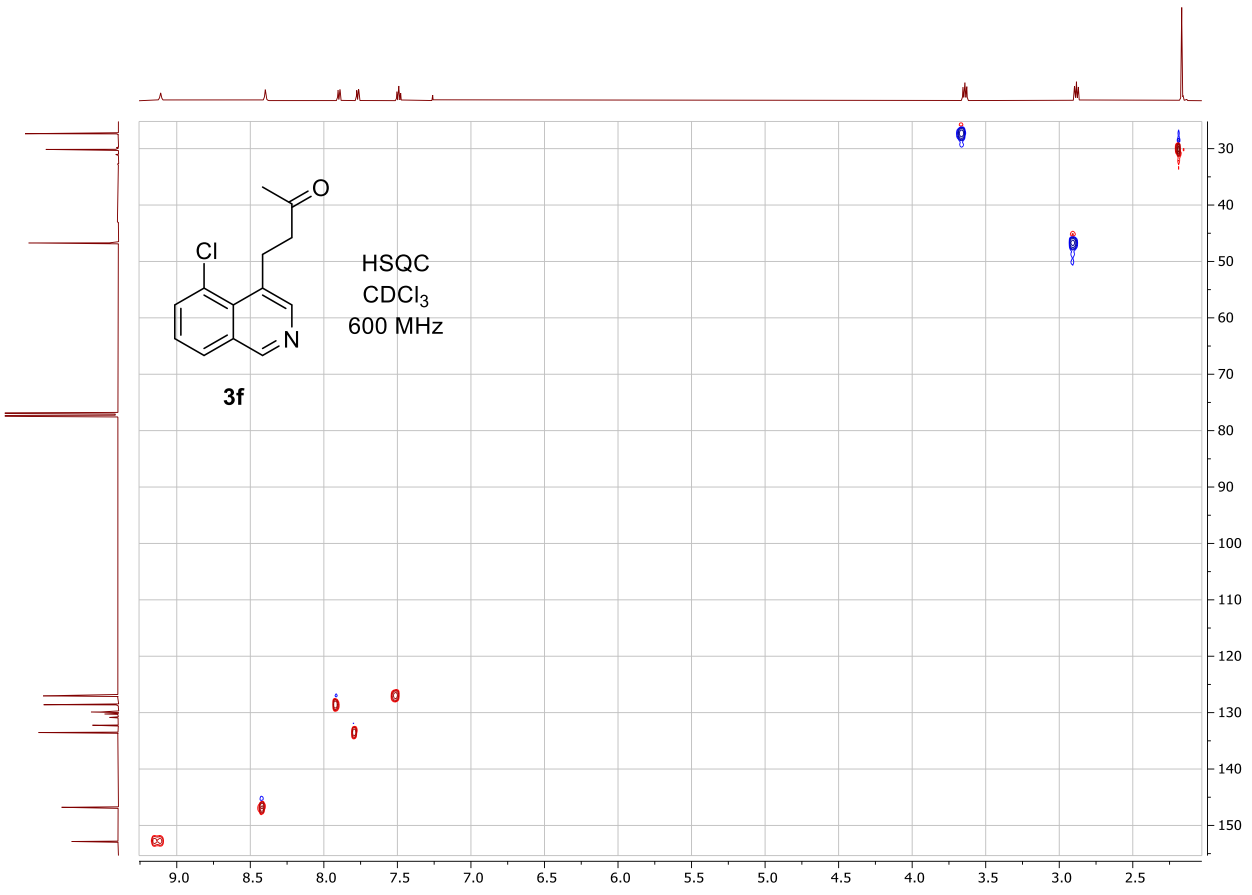
Cl
O
N
3f
HSQC
$CDCl_3$
600 MHz
9.0 8.5 8.0 7.5 7.0 6.5 6.0 5.5 5.0 4.5 4.0 3.5 3.0 2.5
30 40 50 60 70 80 90 100 110 120 130 140 150

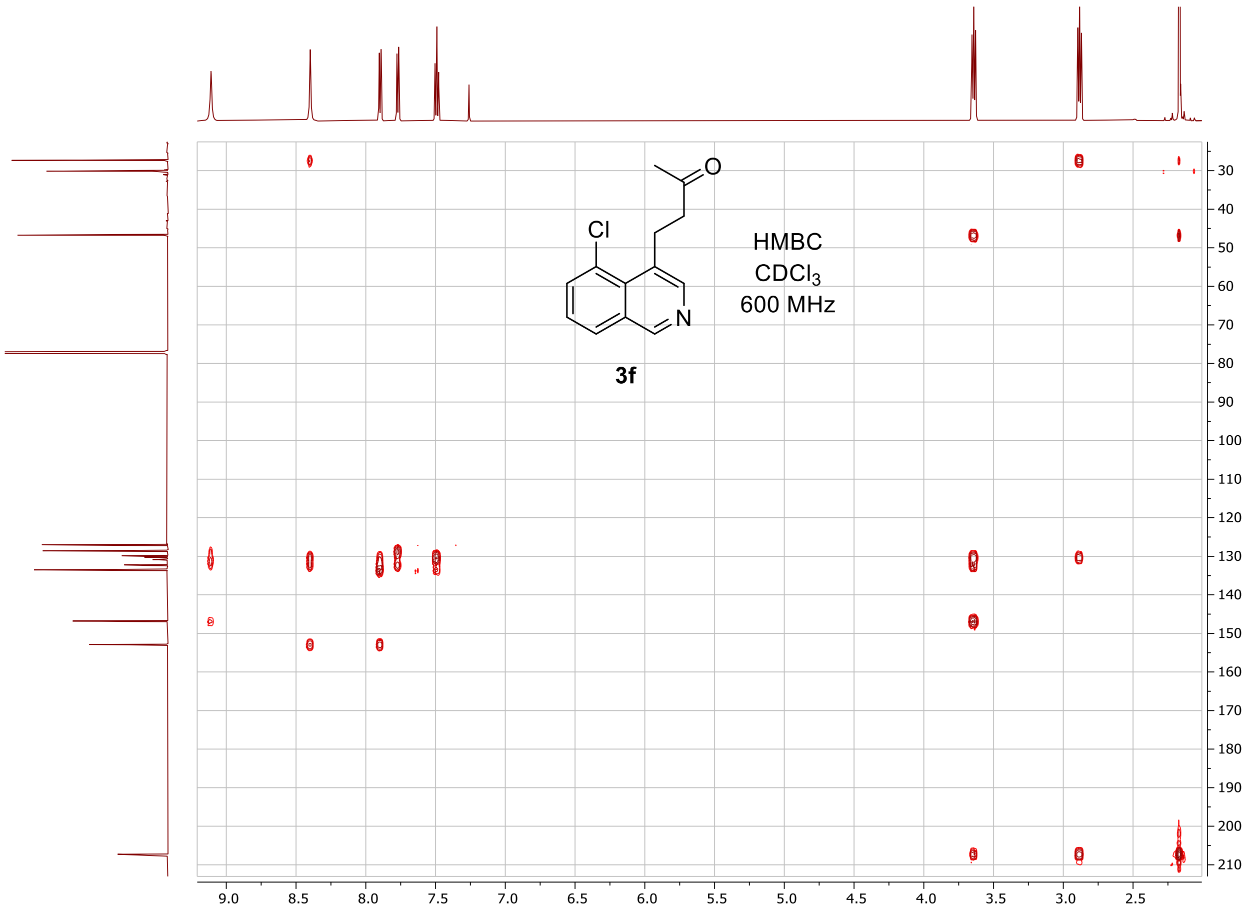
Cl
O
N
3f
HMBC
CDCl3
600 MHz

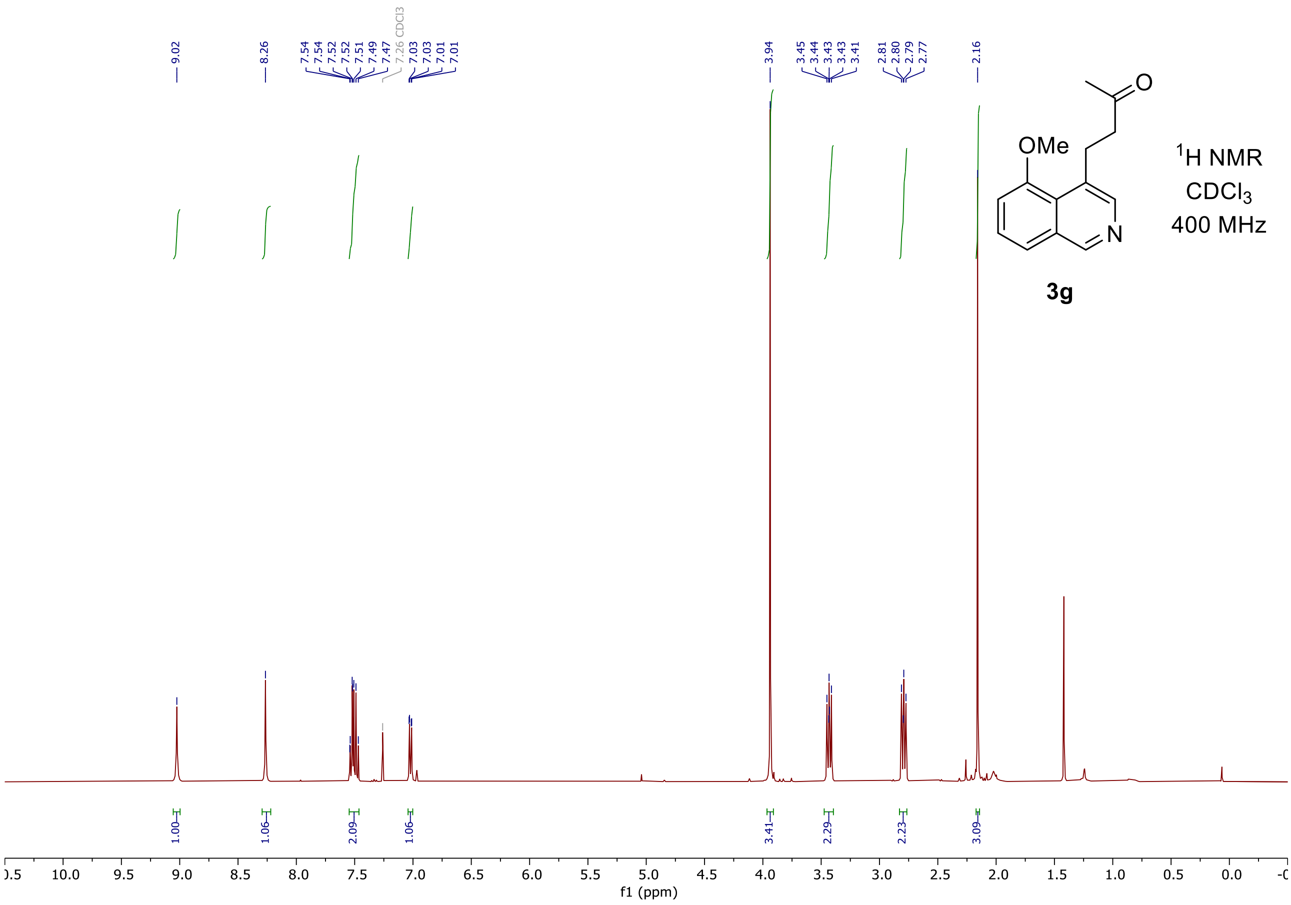
9.02
8.26
7.54
7.54
7.52
7.52
7.51
7.49
7.47
7.26 CDCl3
7.03
7.03
7.01
7.01
3.94
3.45
3.44
3.43
3.43
3.41
2.81
2.80
2.79
2.77
2.16
OMe
O
N
3g
1H NMR
CDCl3
400 MHz
1.00
1.06
2.09
1.06
3.41
2.29
2.23
3.09
f1 (ppm)

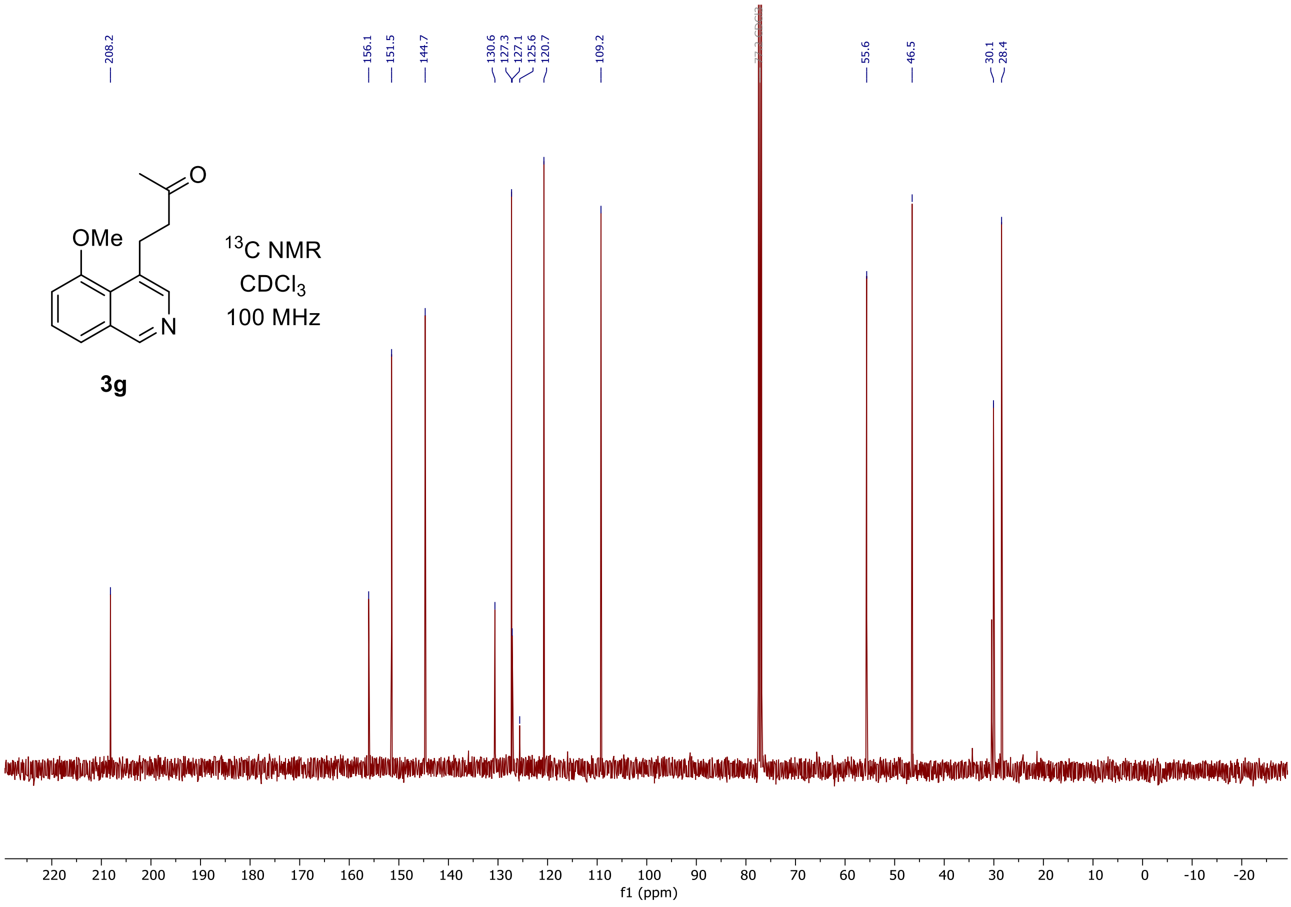
208.2
156.1
151.5
144.7
130.6
127.3
127.1
125.6
120.7
109.2
55.6
46.5
30.1
28.4
OMe
O
N
3g
13C NMR
CDCl3
100 MHz
f1 (ppm)

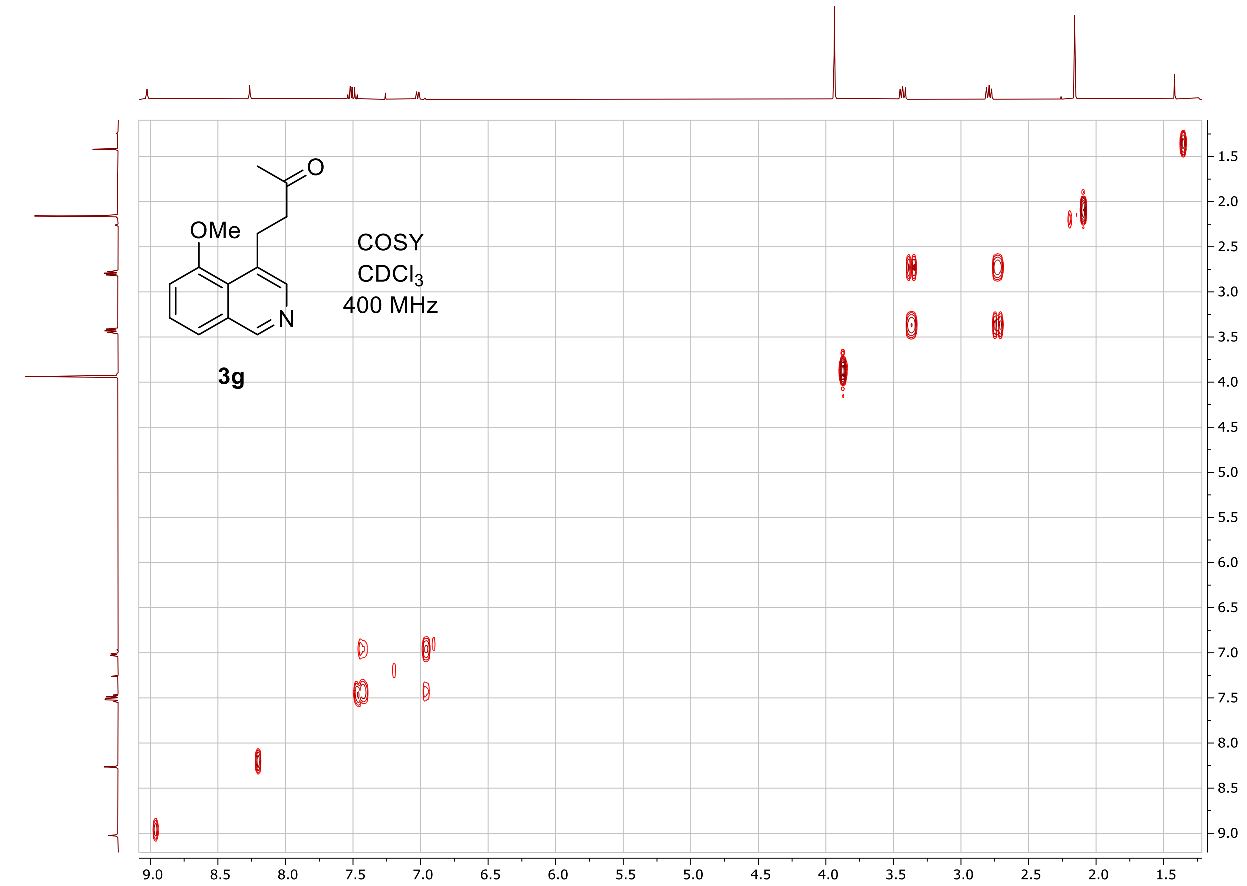

OMe
O
N
3g
COSY
CDCl3
400 MHz

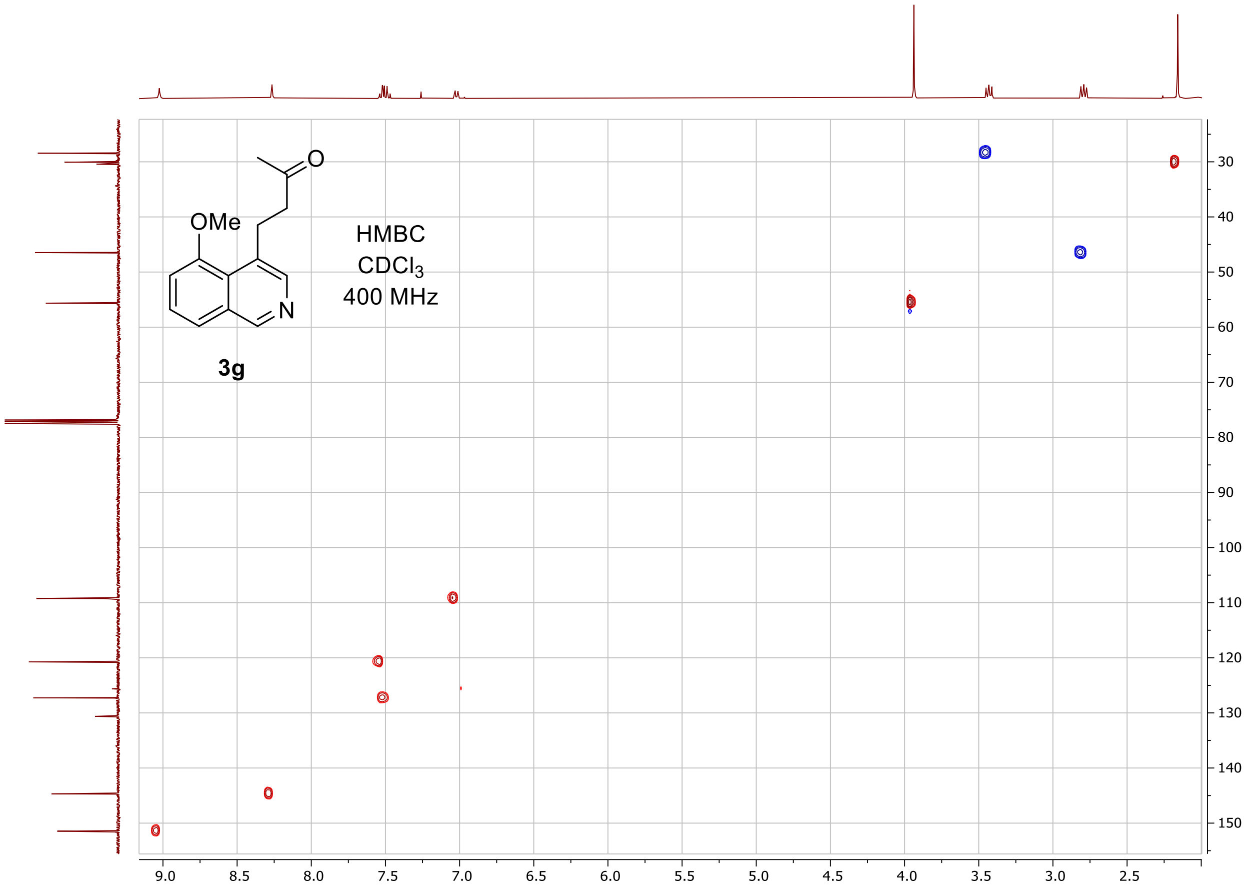

OMe
O
N
3g
HMBC
CDCl3
400 MHz

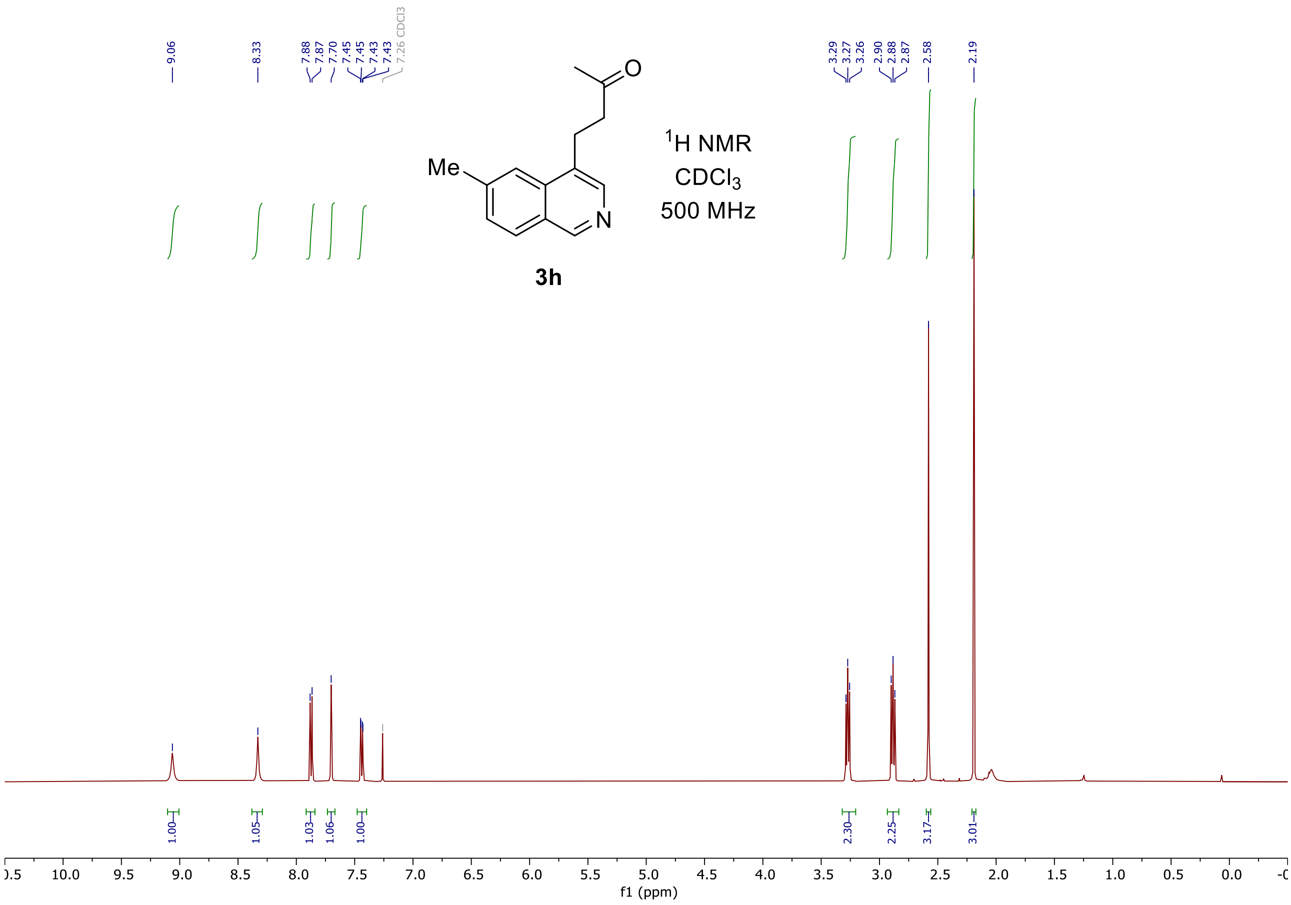
9.06
8.33
7.88
7.87
7.70
7.45
7.45
7.43
7.43
7.26 CDCl3
3.29
3.27
3.26
2.90
2.88
2.87
2.58
2.19
Me
N
O
3h
1H NMR
CDCl3
500 MHz
1.00
1.05
1.03
1.06
1.00
2.30
2.25
3.17
3.01
f1 (ppm)

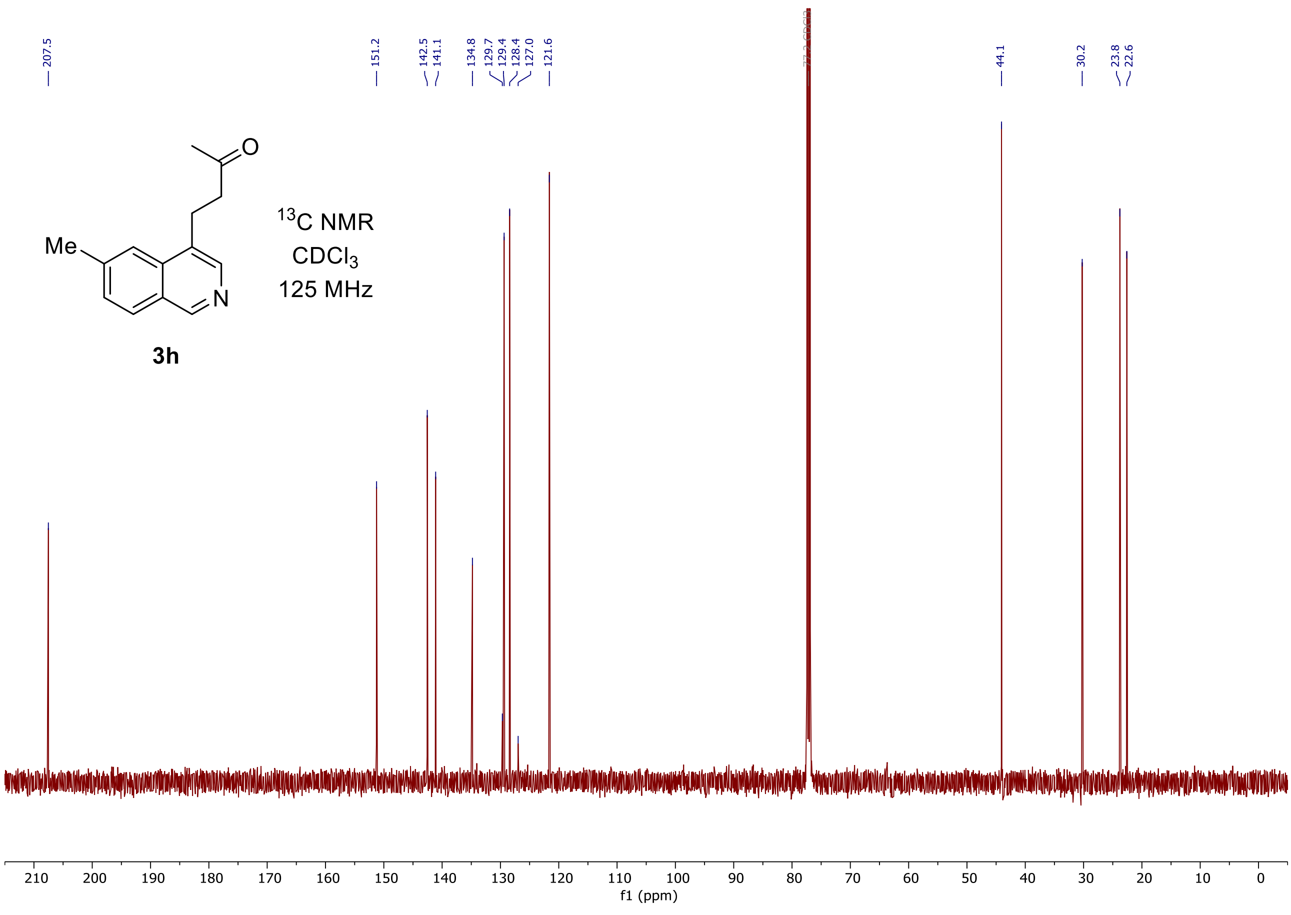
207.5
151.2
142.5
141.1
134.8
129.7
129.4
128.4
127.0
121.6
44.1
30.2
23.8
22.6
Me
N
O
3h
13C NMR
CDCl3
125 MHz
f1 (ppm)

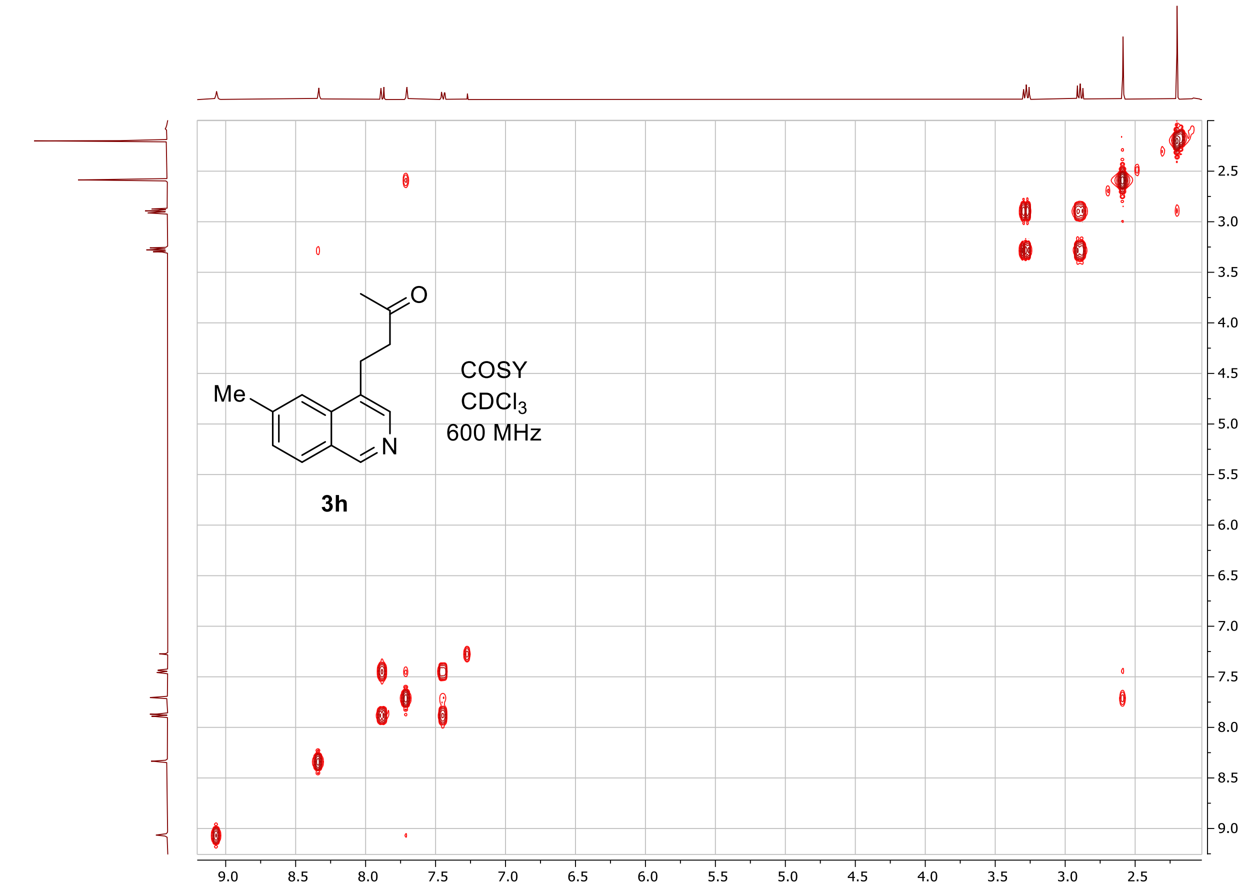

Me
N
O
3h
COSY
CDCl3
600 MHz

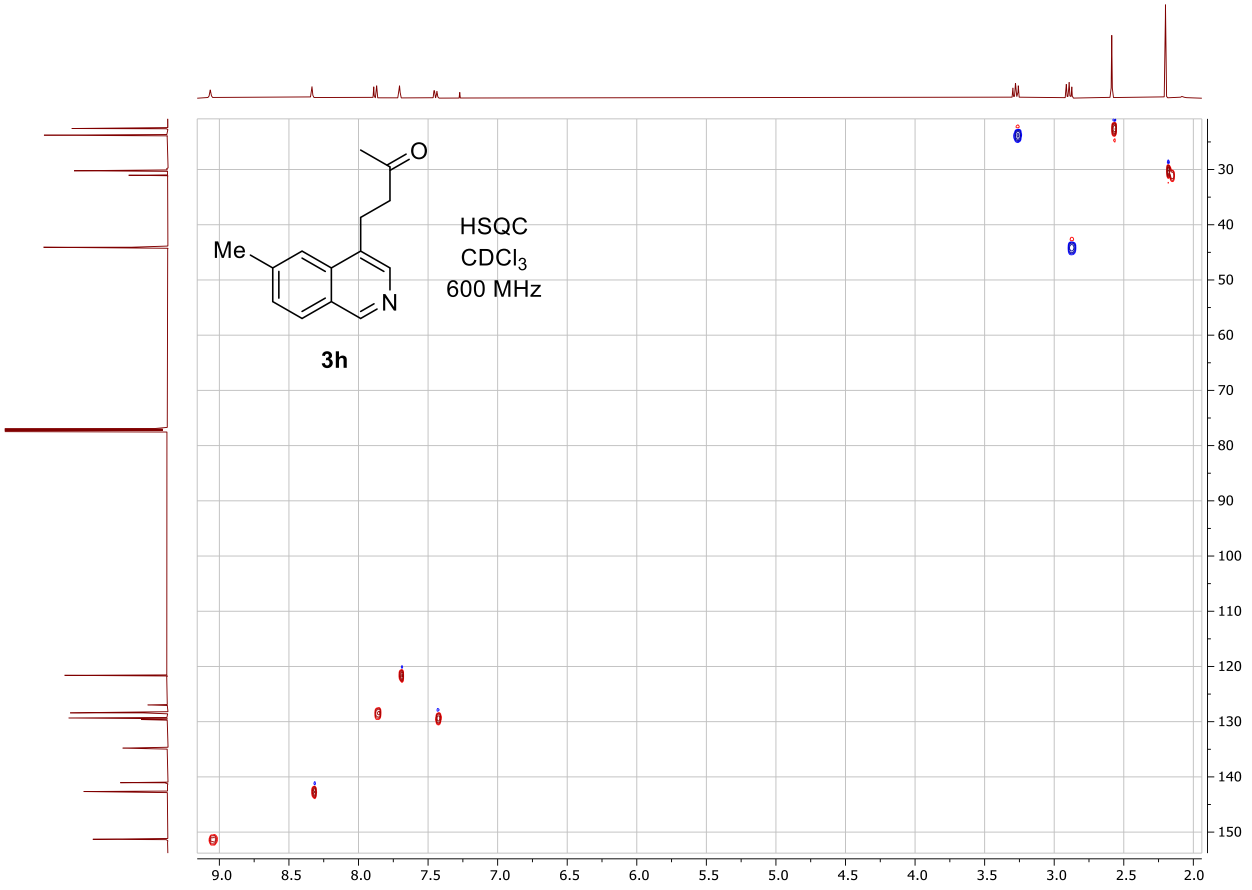

Me
N
O
3h
HSQC
CDCl3
600 MHz

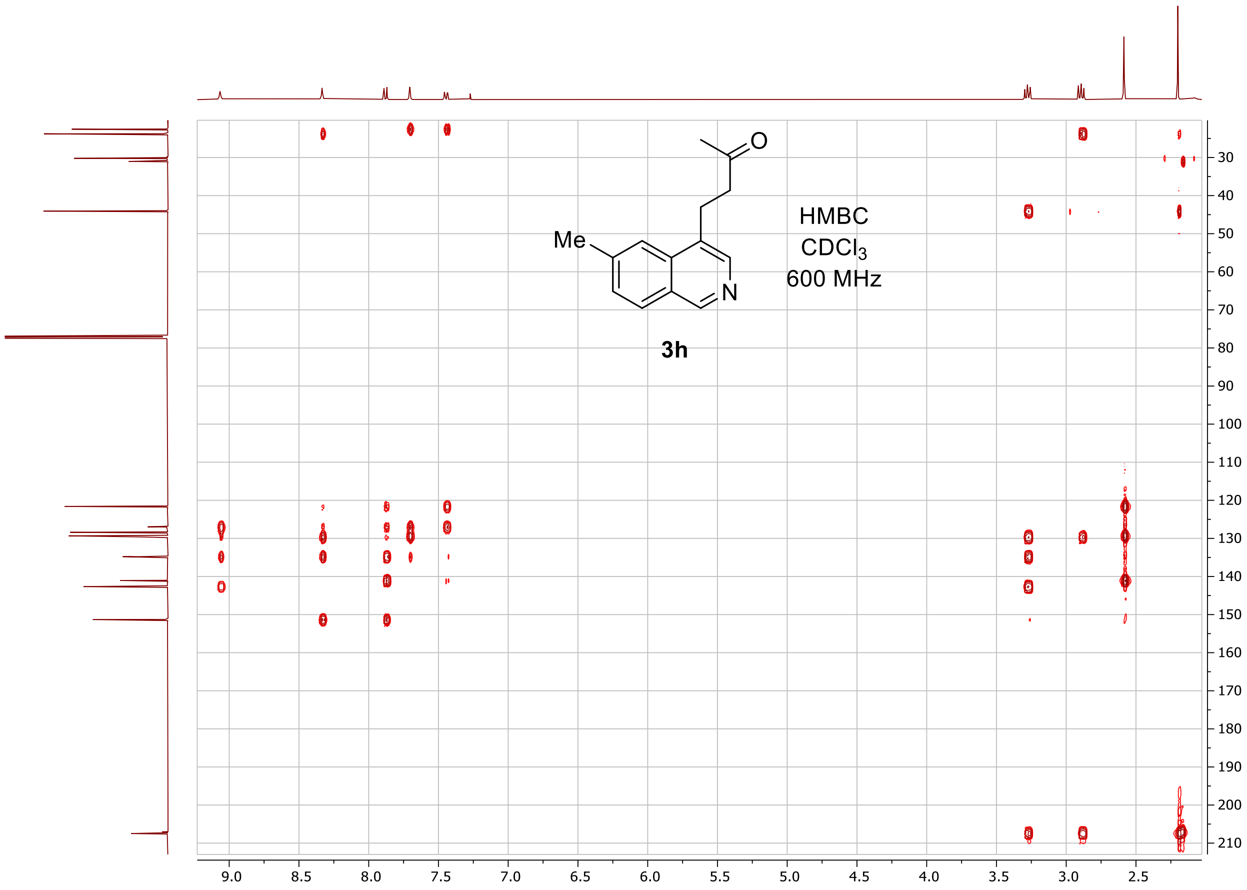
O
Me
N
3h
HMBC
CDCl3
600 MHz

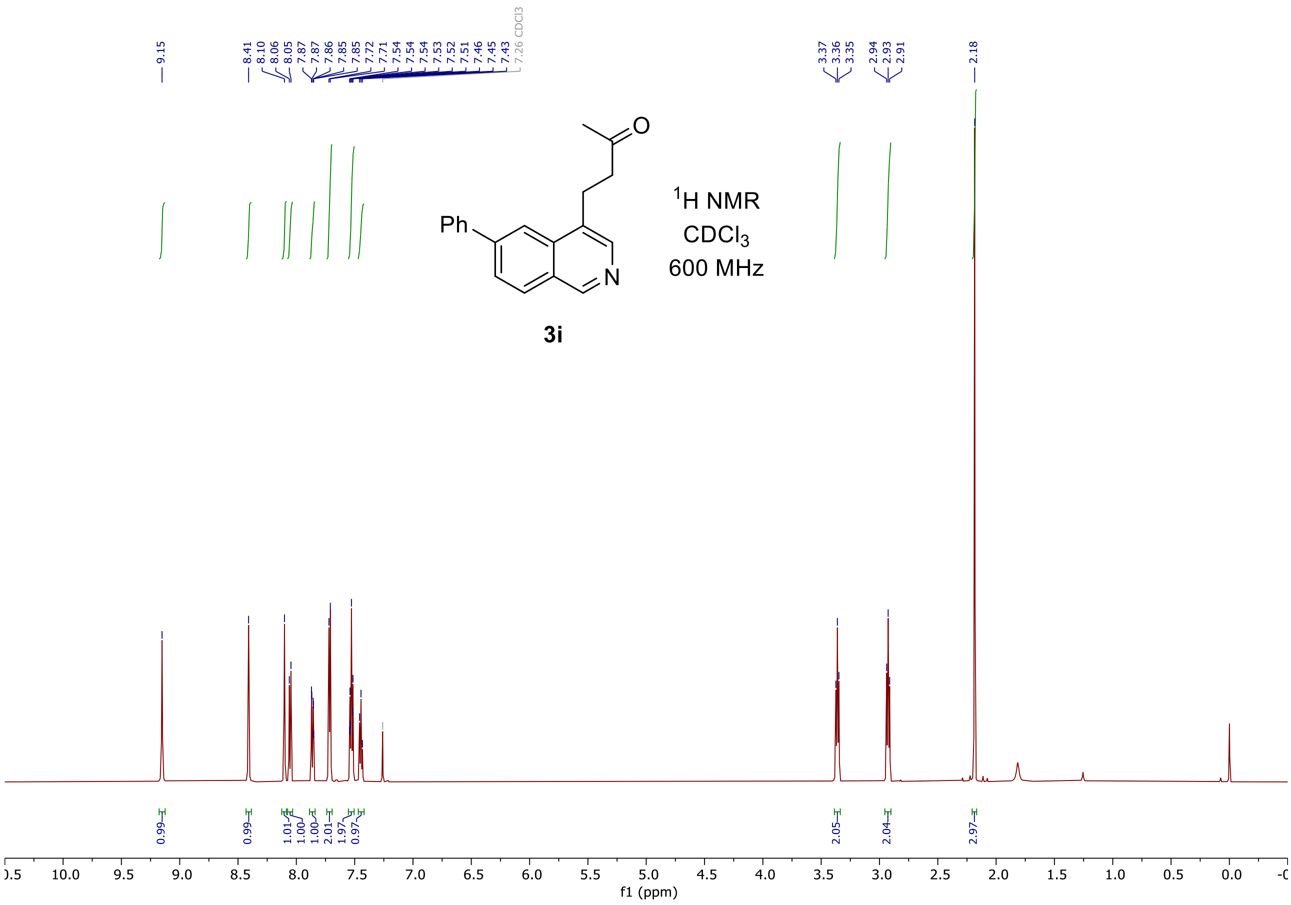

9.15
8.41
8.10
8.06
8.05
7.87
7.86
7.85
7.85
7.72
7.71
7.54
7.54
7.54
7.53
7.52
7.51
7.46
7.45
7.43
7.26 CDCl3
3.37
3.36
3.35
2.94
2.93
2.91
2.18
Ph
O
N
3i
^{1}H NMR
$CDCl_3$
600 MHz
0.99
0.99
1.01
1.00
1.00
2.01
1.97
0.97
2.05
2.04
2.97
f1 (ppm)

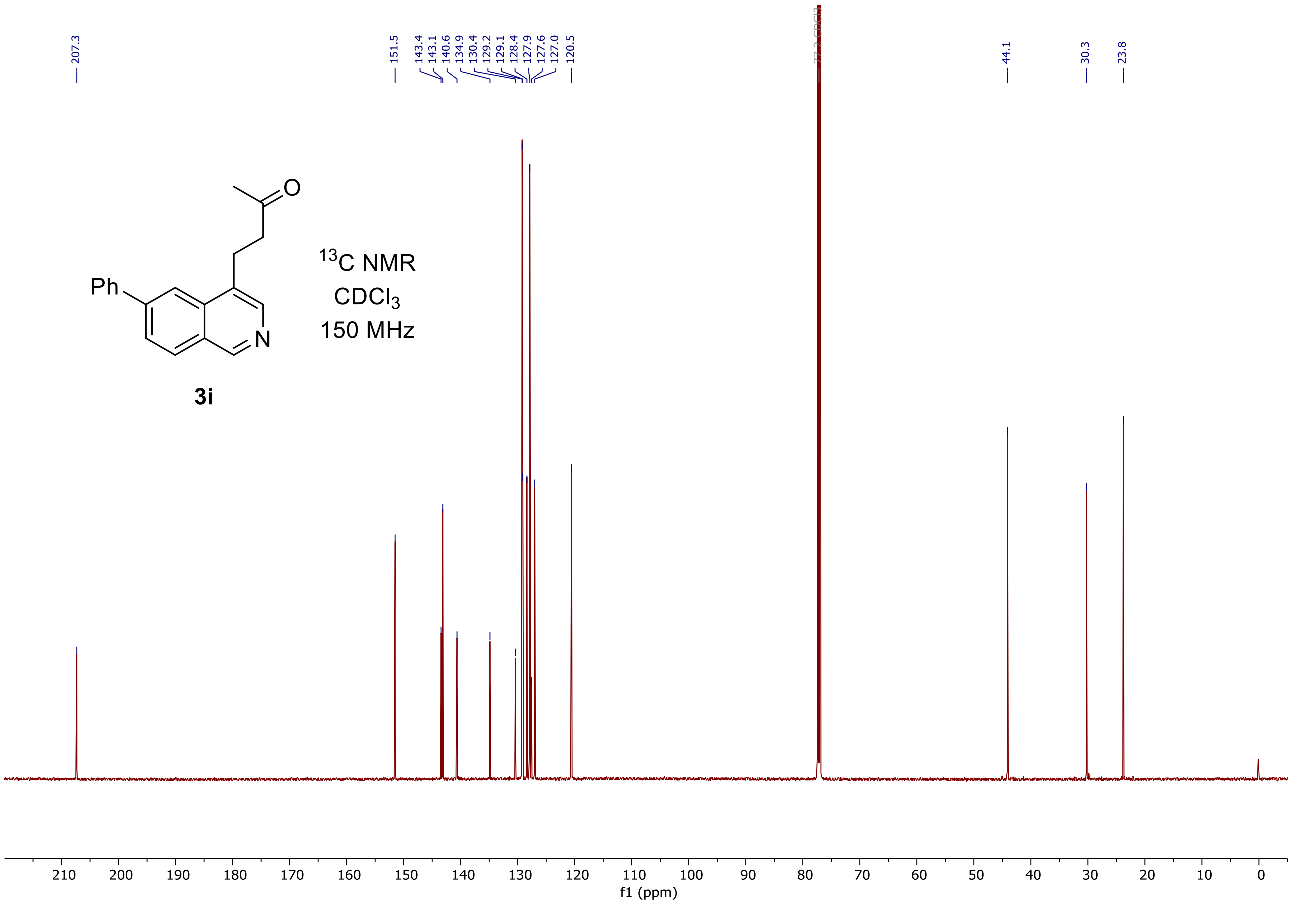

207.3
151.5
143.4
143.1
140.6
134.9
130.4
129.2
129.1
128.4
127.9
127.6
127.0
120.5
44.1
30.3
23.8
Ph
O
N
3i
^{13}C NMR
$CDCl_3$
150 MHz
f1 (ppm)

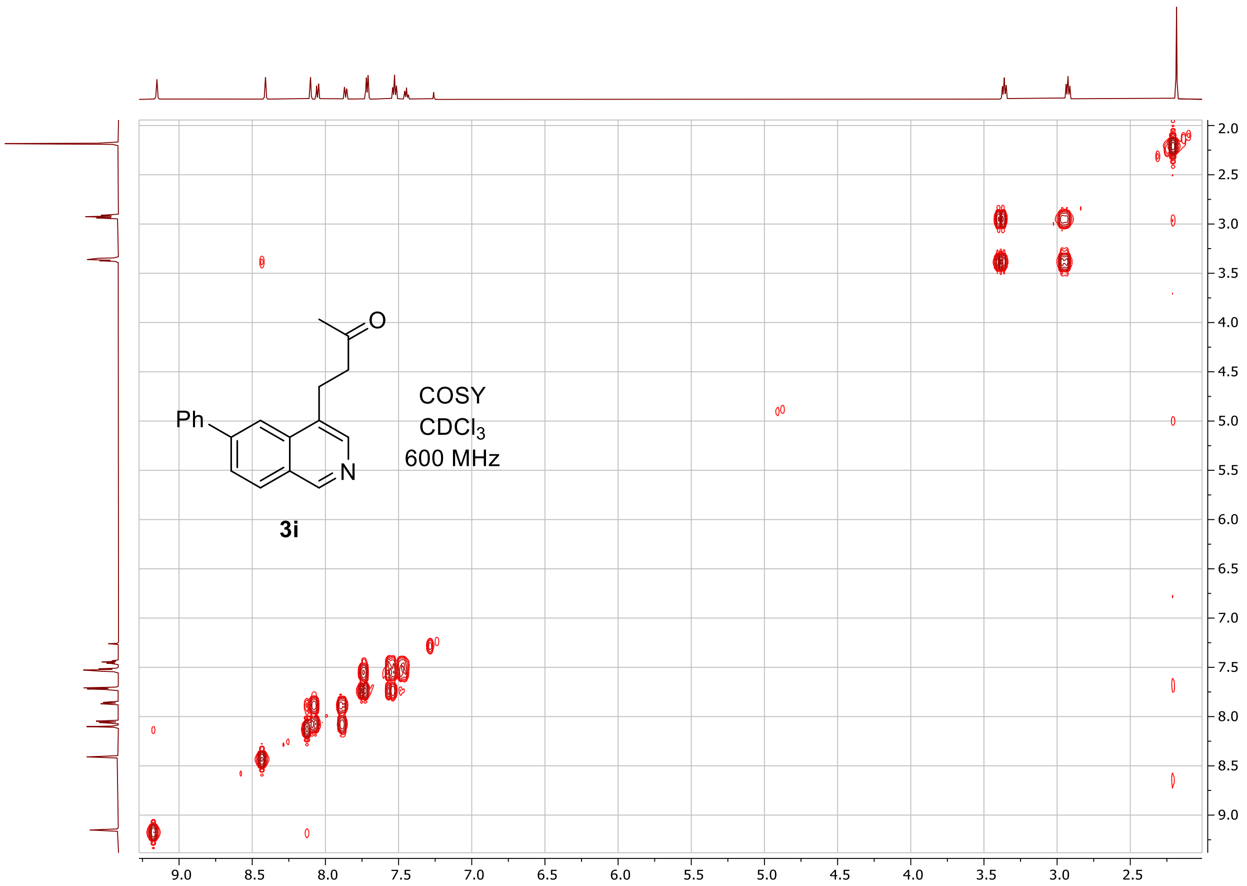
COSY
CDCl3
600 MHz
3i

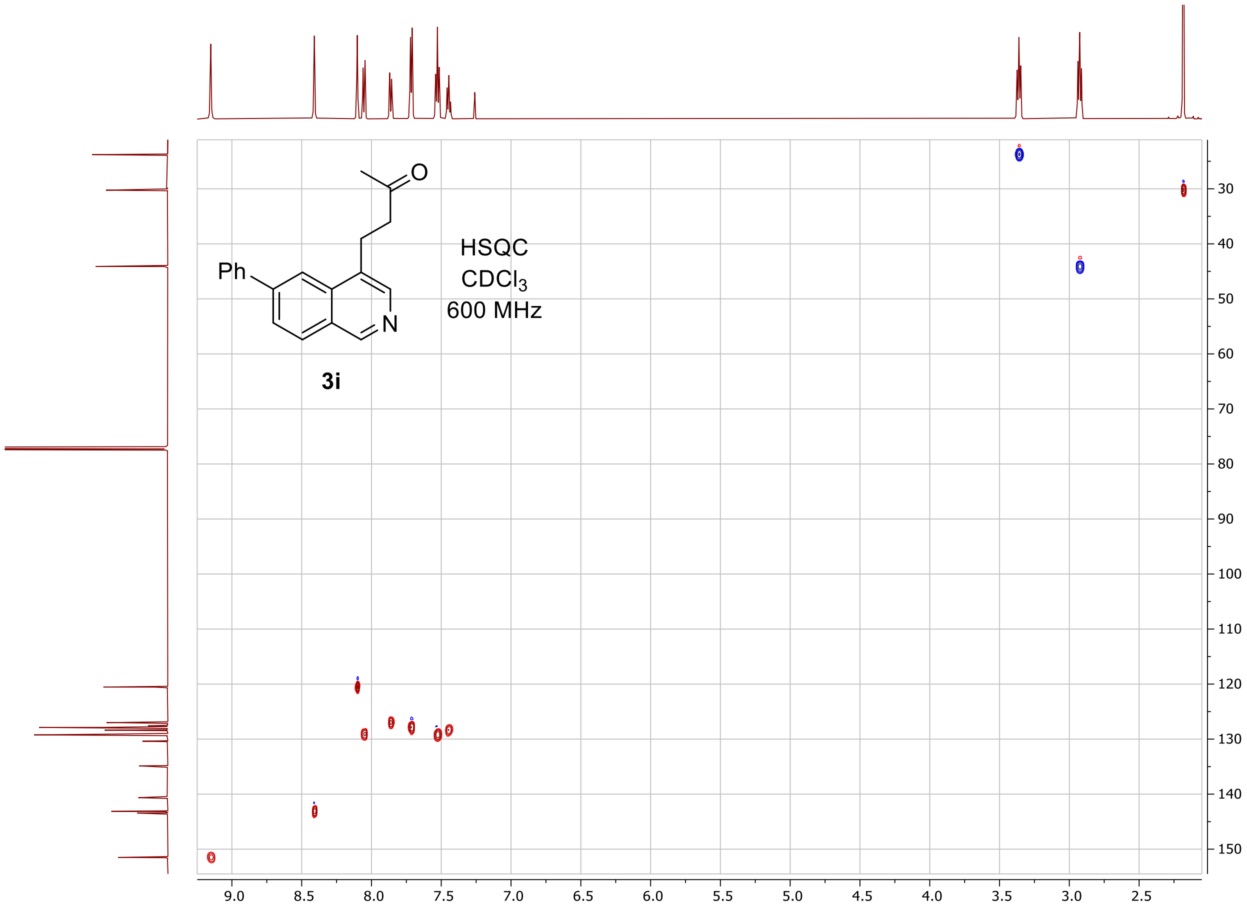
HSQC
CDCl3
600 MHz
3i

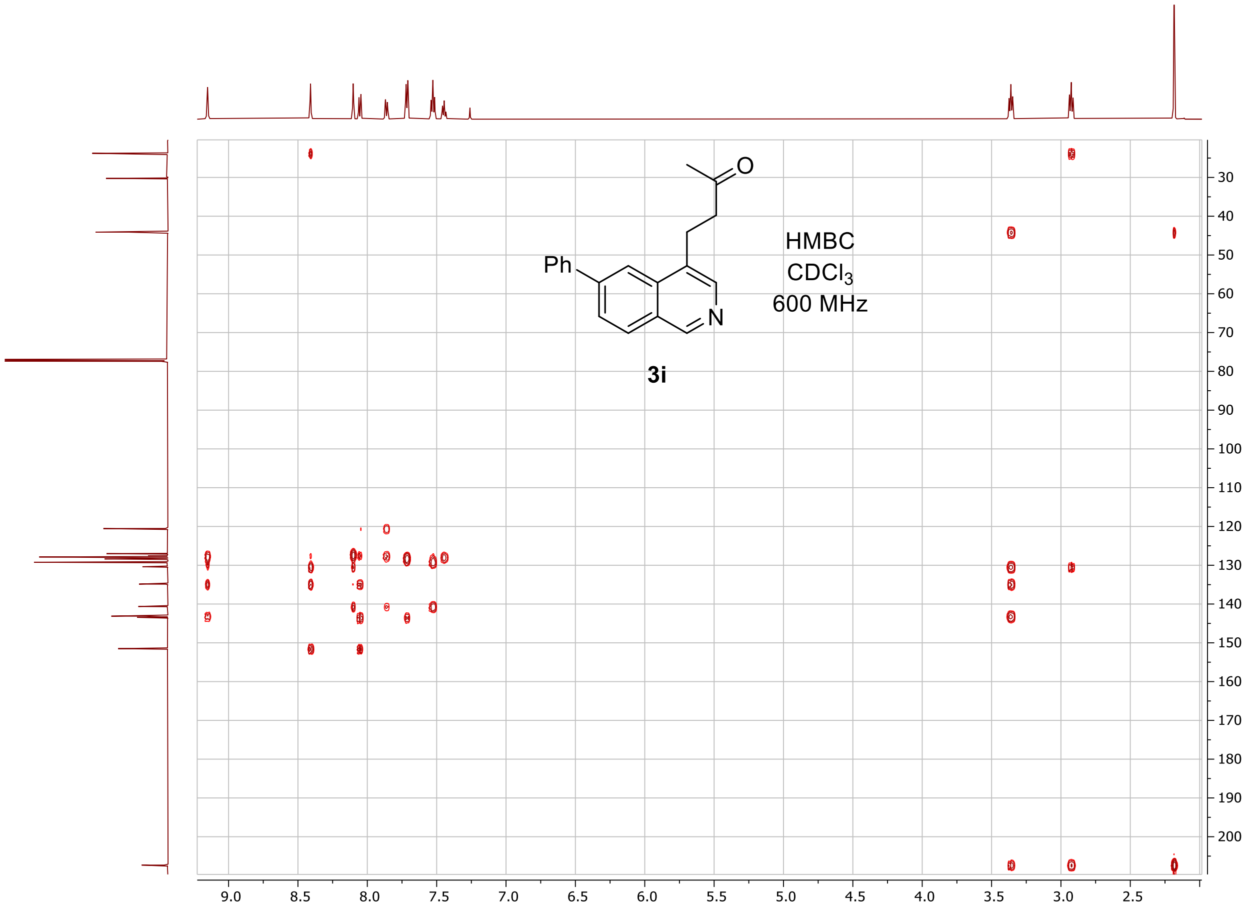

HMBC
CDCl3
600 MHz
Ph
N
O
3i

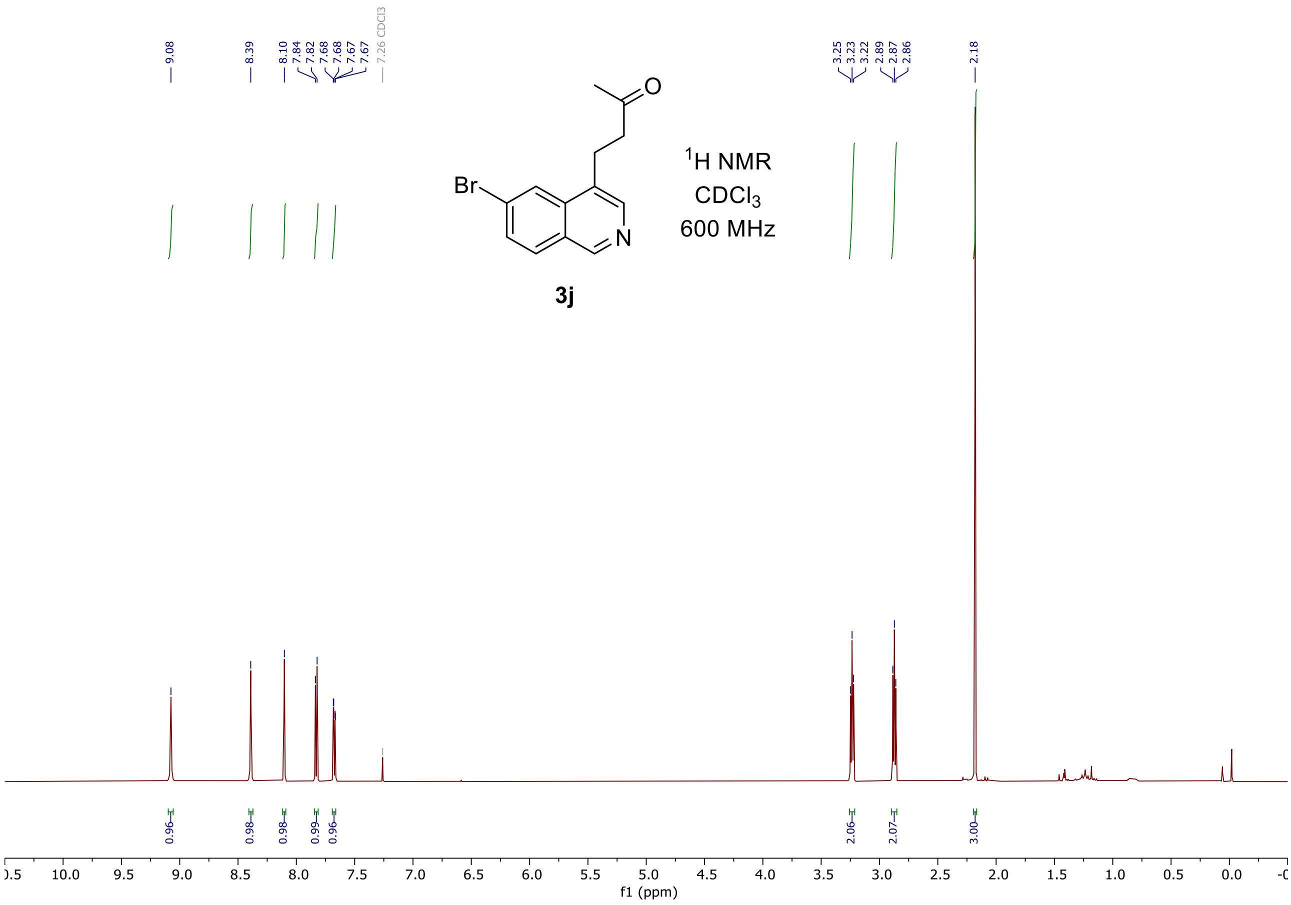
9.08
8.39
8.10
7.84
7.82
7.68
7.68
7.67
7.67
7.26 CDCl3
3.25
3.23
3.22
2.89
2.87
2.86
2.18
Br
O
N
3j
^{1}H NMR
$CDCl_3$
600 MHz
0.96
0.98
0.98
0.99
0.96
2.06
2.07
3.00
f1 (ppm)

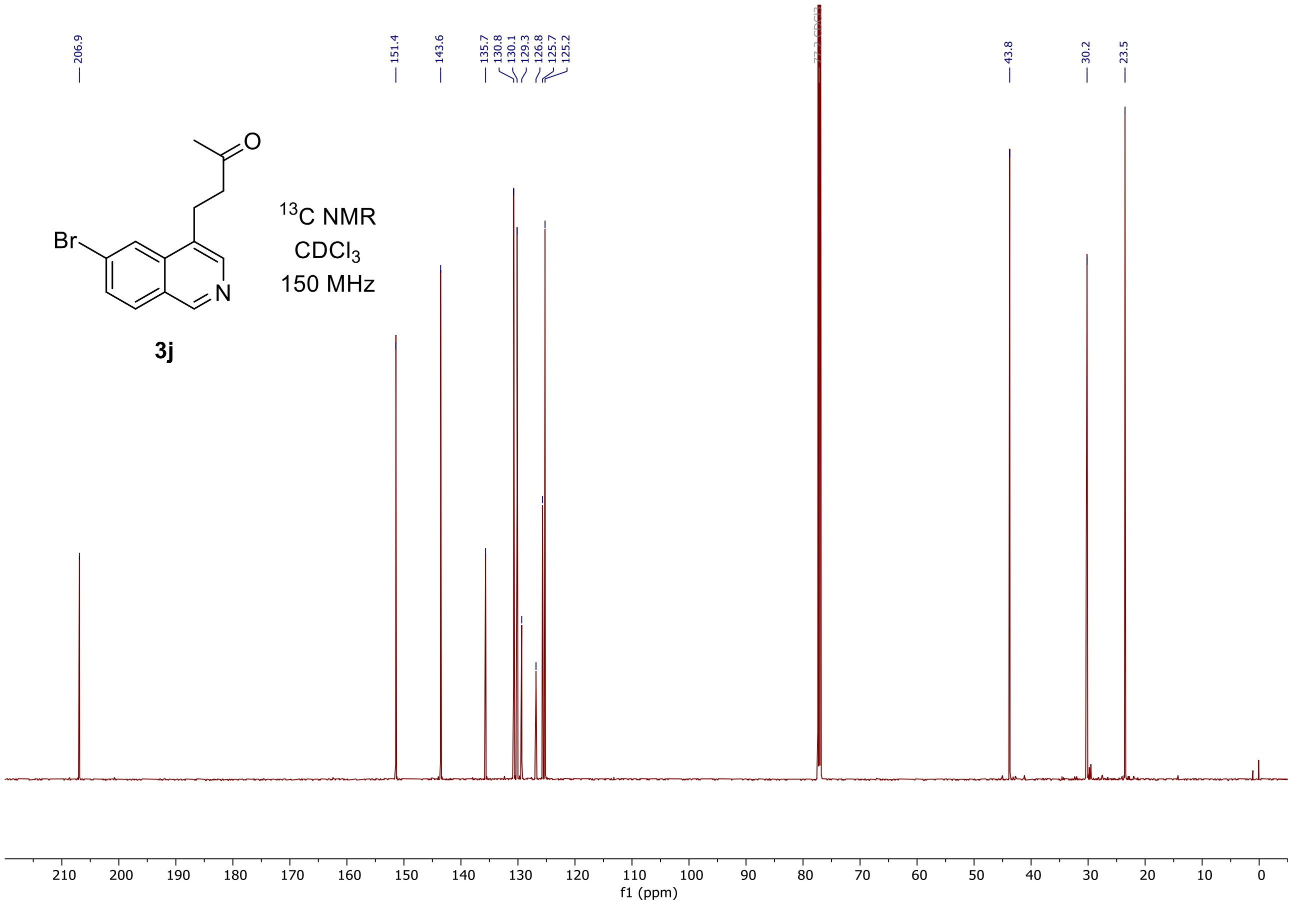
206.9
151.4
143.6
135.7
130.8
130.1
129.3
126.8
125.7
125.2
43.8
30.2
23.5
Br
O
N
3j
^{13}C NMR
$CDCl_3$
150 MHz
f1 (ppm)

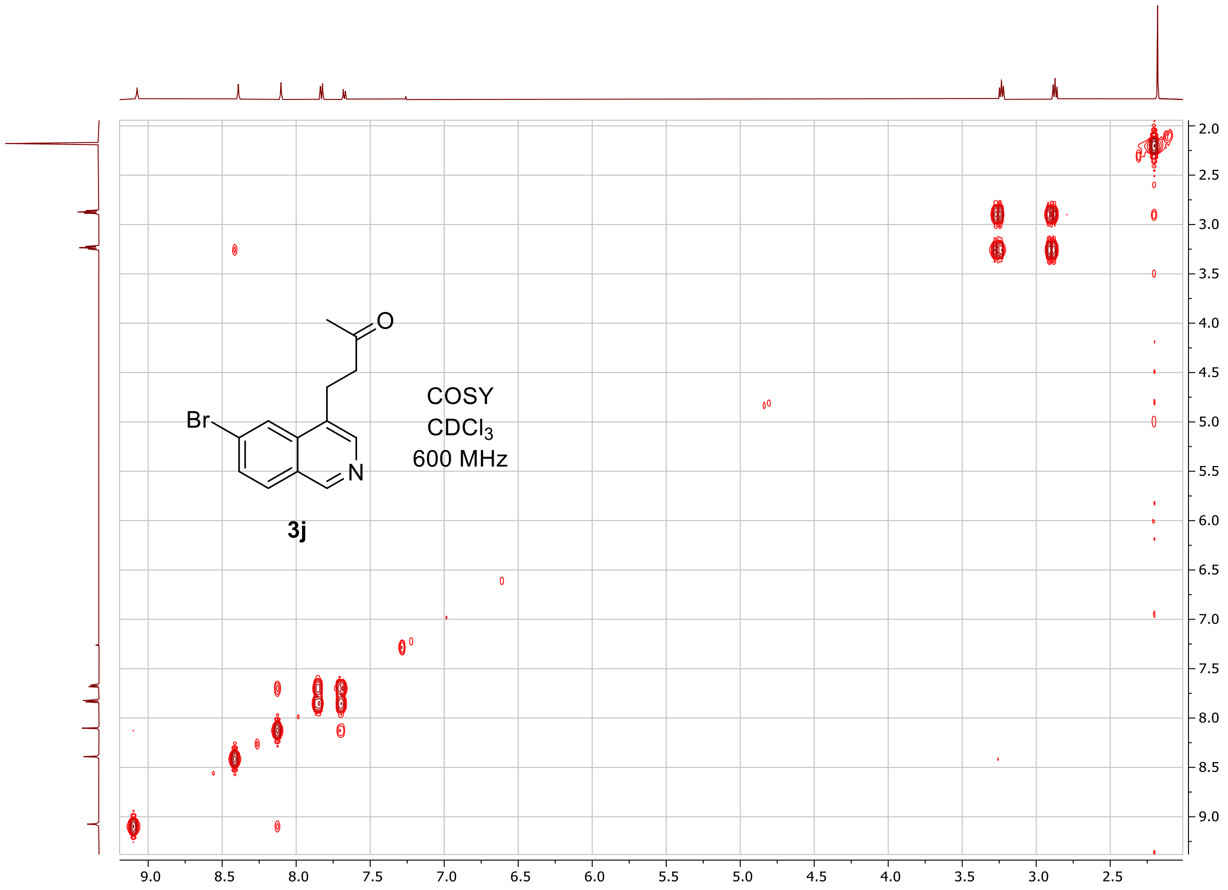

Br
O
N
3j
COSY
CDCl3
600 MHz

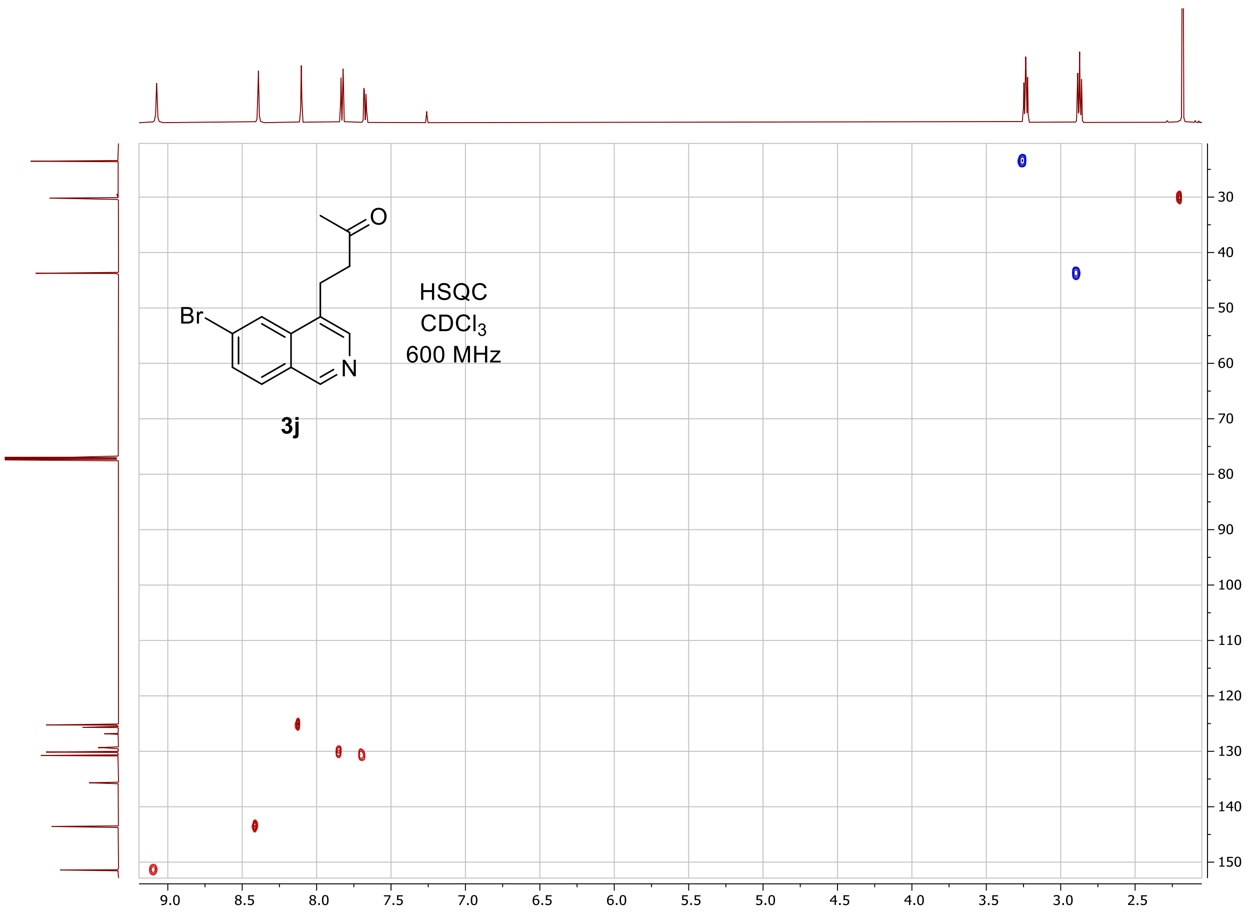

Br
O
N
3j
HSQC
CDCl3
600 MHz

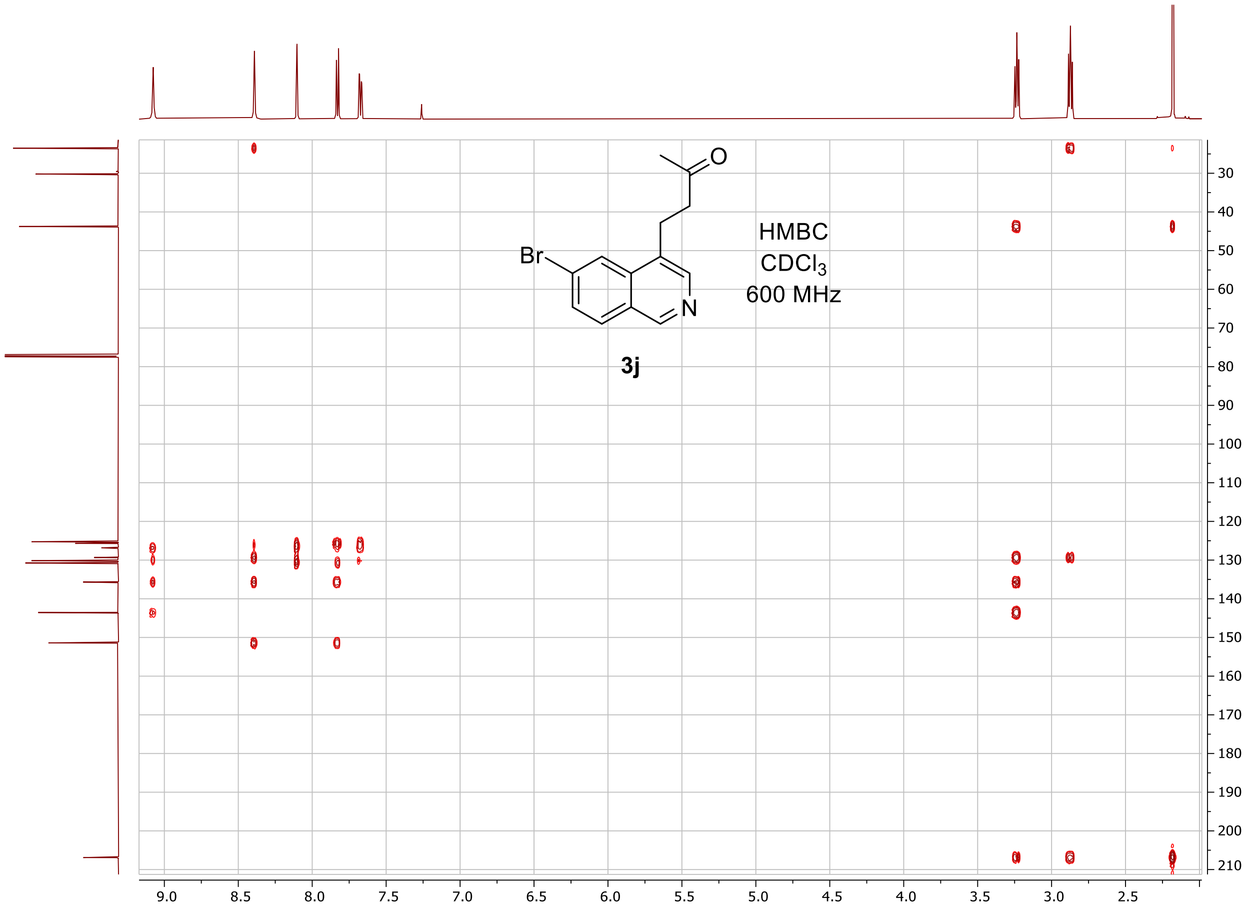
HMBC
CDCl3
600 MHz
3j
Br
N
O

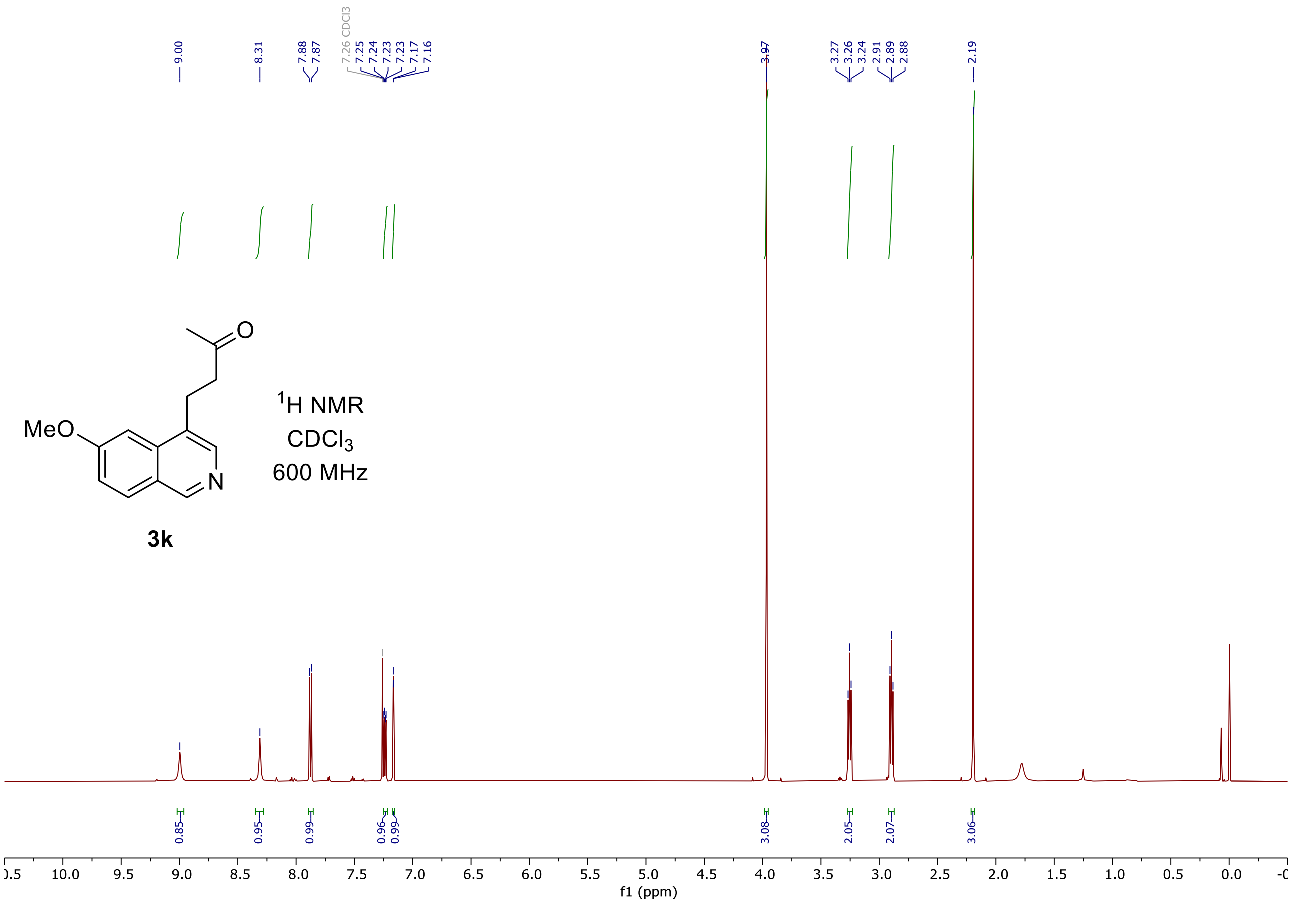
9.00
8.31
7.88
7.87
7.26 CDCl3
7.25
7.24
7.23
7.23
7.17
7.16
3.97
3.27
3.26
3.24
2.91
2.89
2.88
2.19
MeO
O
N
3k
^{1}H NMR
CDCl$_3$
600 MHz
0.85
0.95
0.99
0.96
0.99
3.08
2.05
2.07
3.06
f1 (ppm)

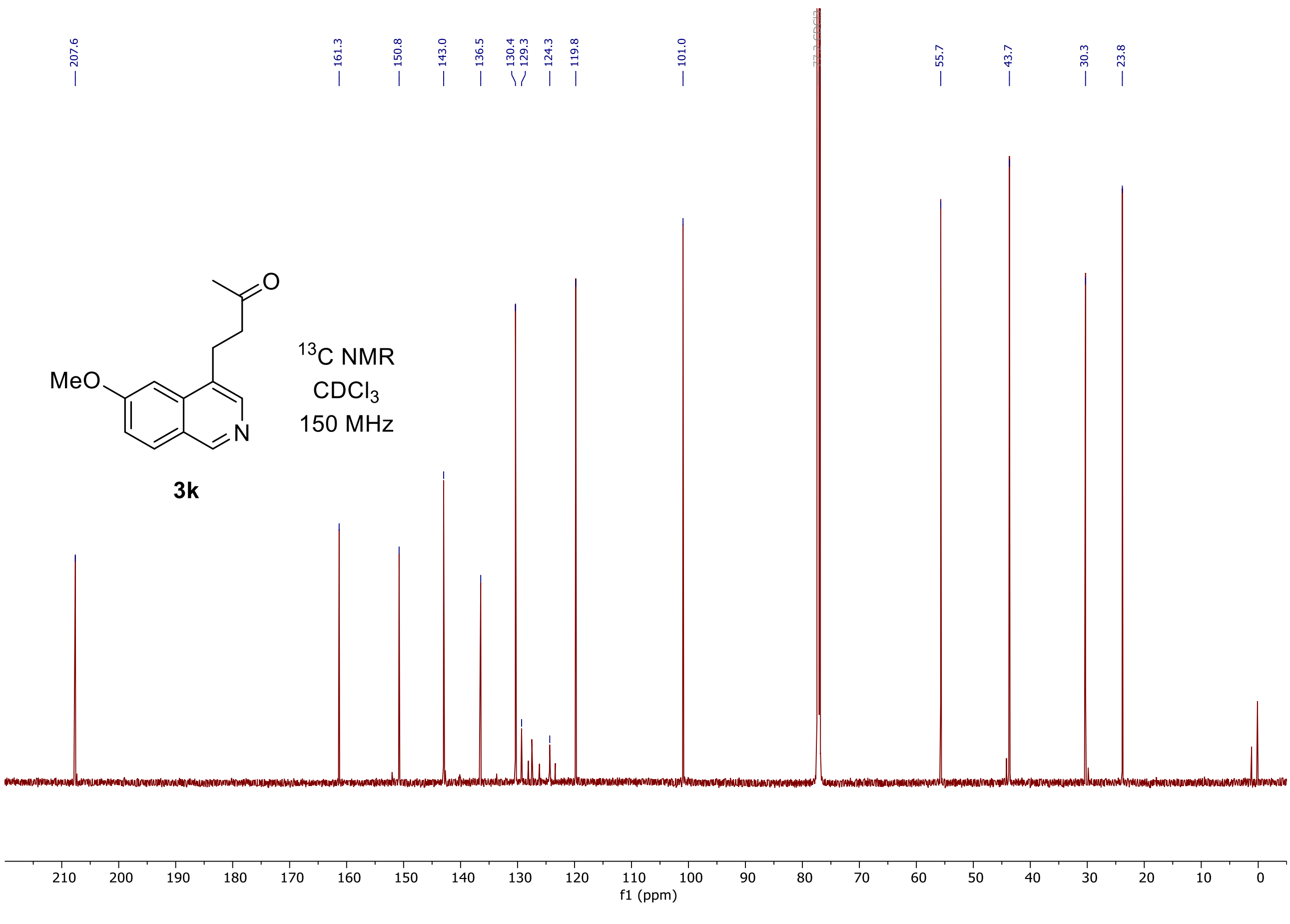
207.6
161.3
150.8
143.0
136.5
130.4
129.3
124.3
119.8
101.0
55.7
43.7
30.3
23.8
MeO
O
N
3k
^{13}C NMR
CDCl$_3$
150 MHz
f1 (ppm)

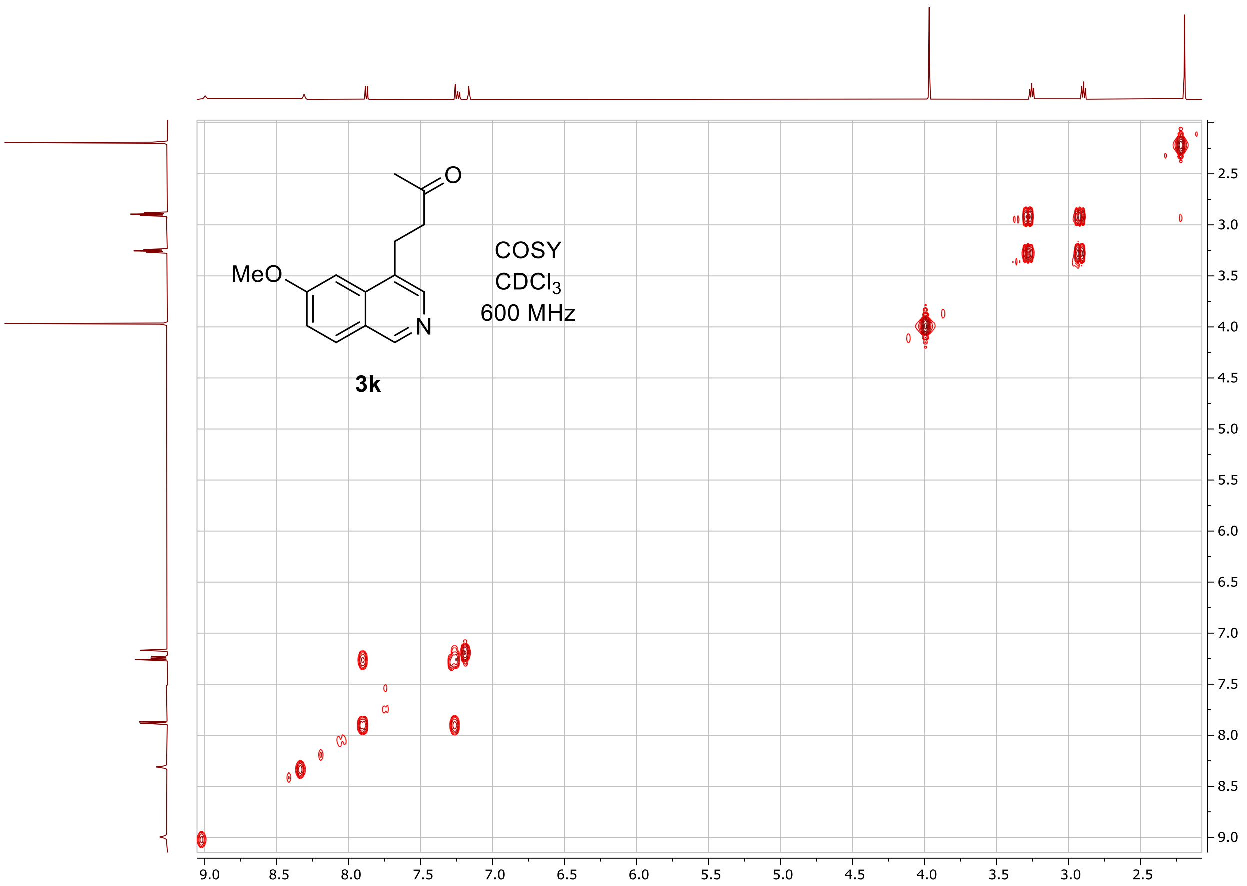
MeO
O
N
3k
COSY
$CDCl_3$
600 MHz

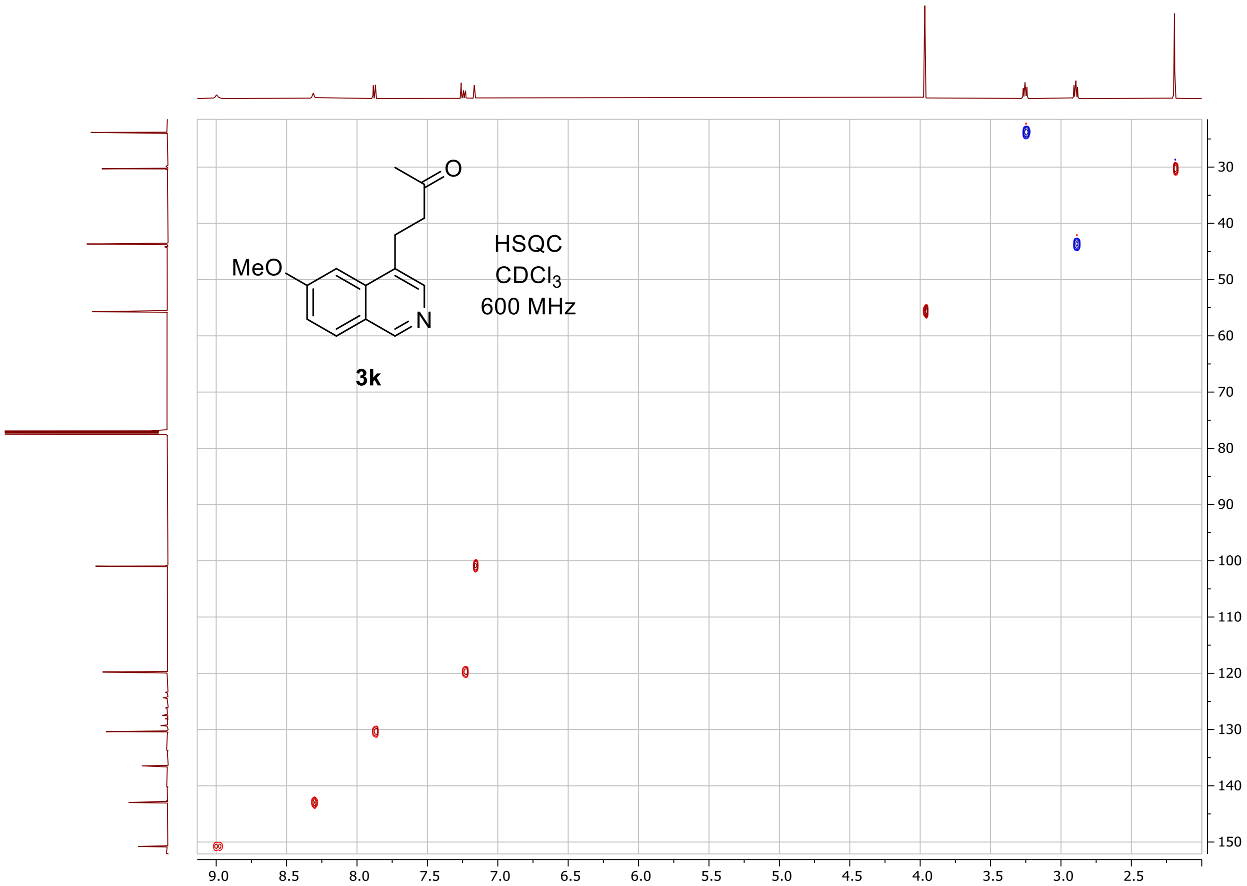
MeO
O
N
3k
HSQC
$CDCl_3$
600 MHz

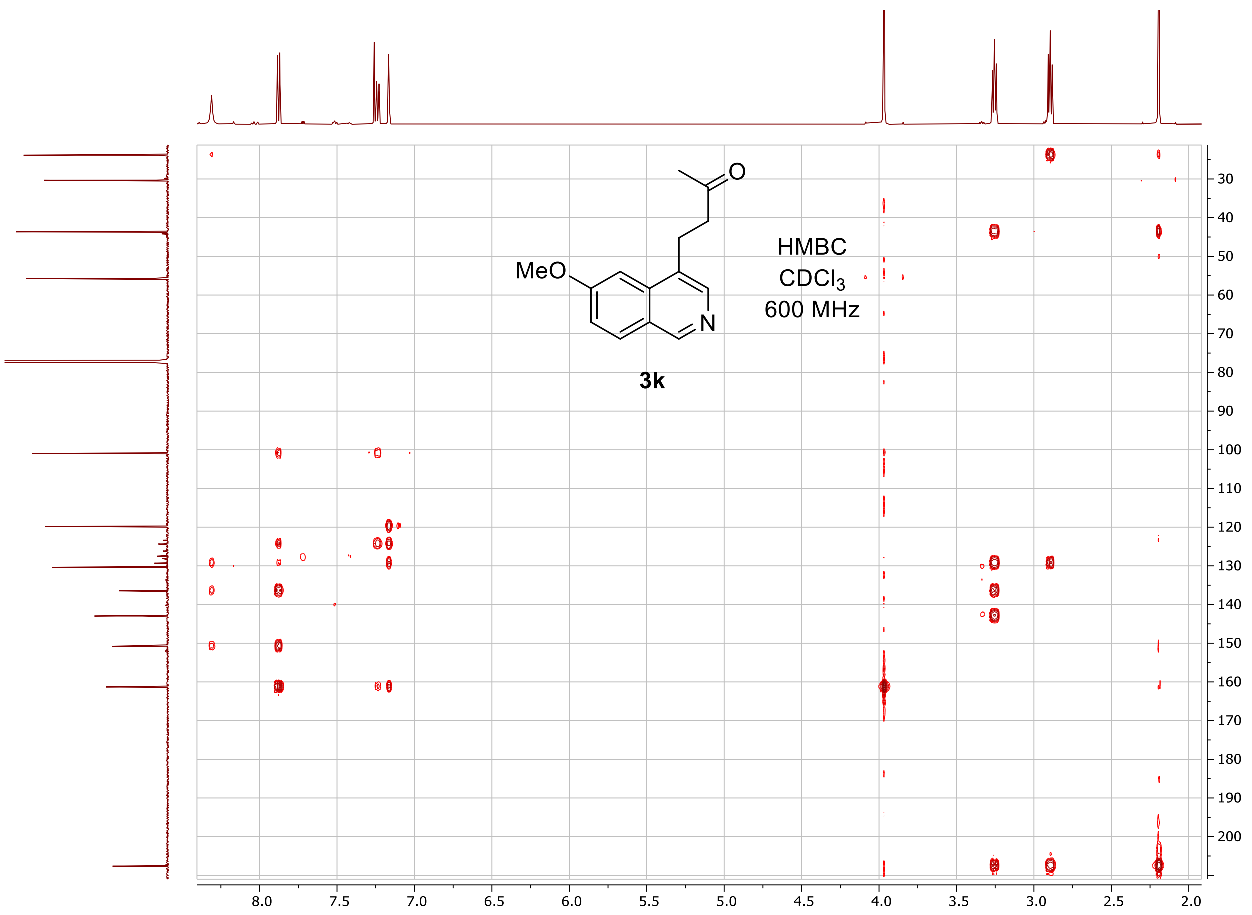

MeO
O
N
3k
HMBC
CDCl$_3$
600 MHz

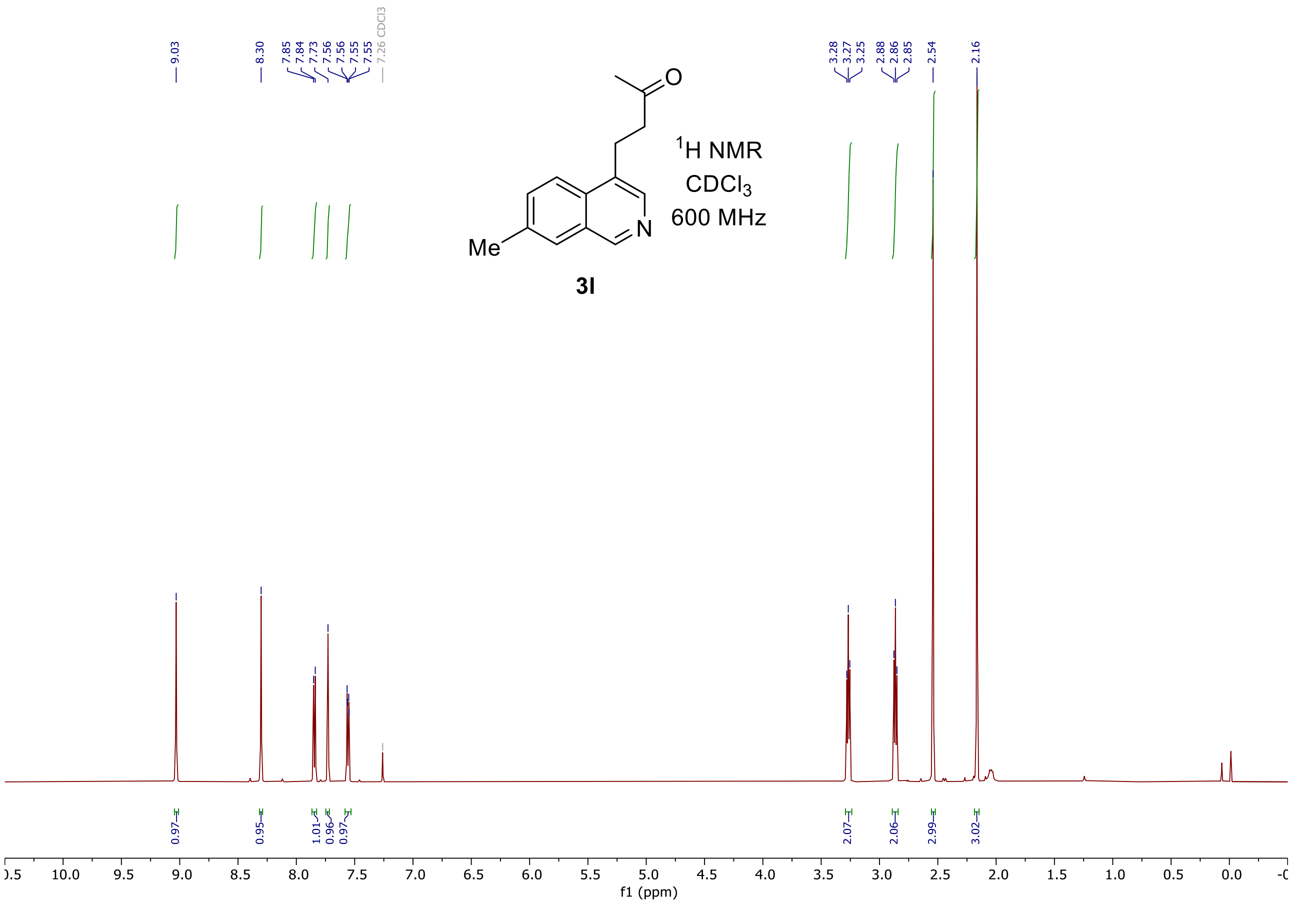
1H NMR
CDCl3
600 MHz
3l
f1 (ppm)

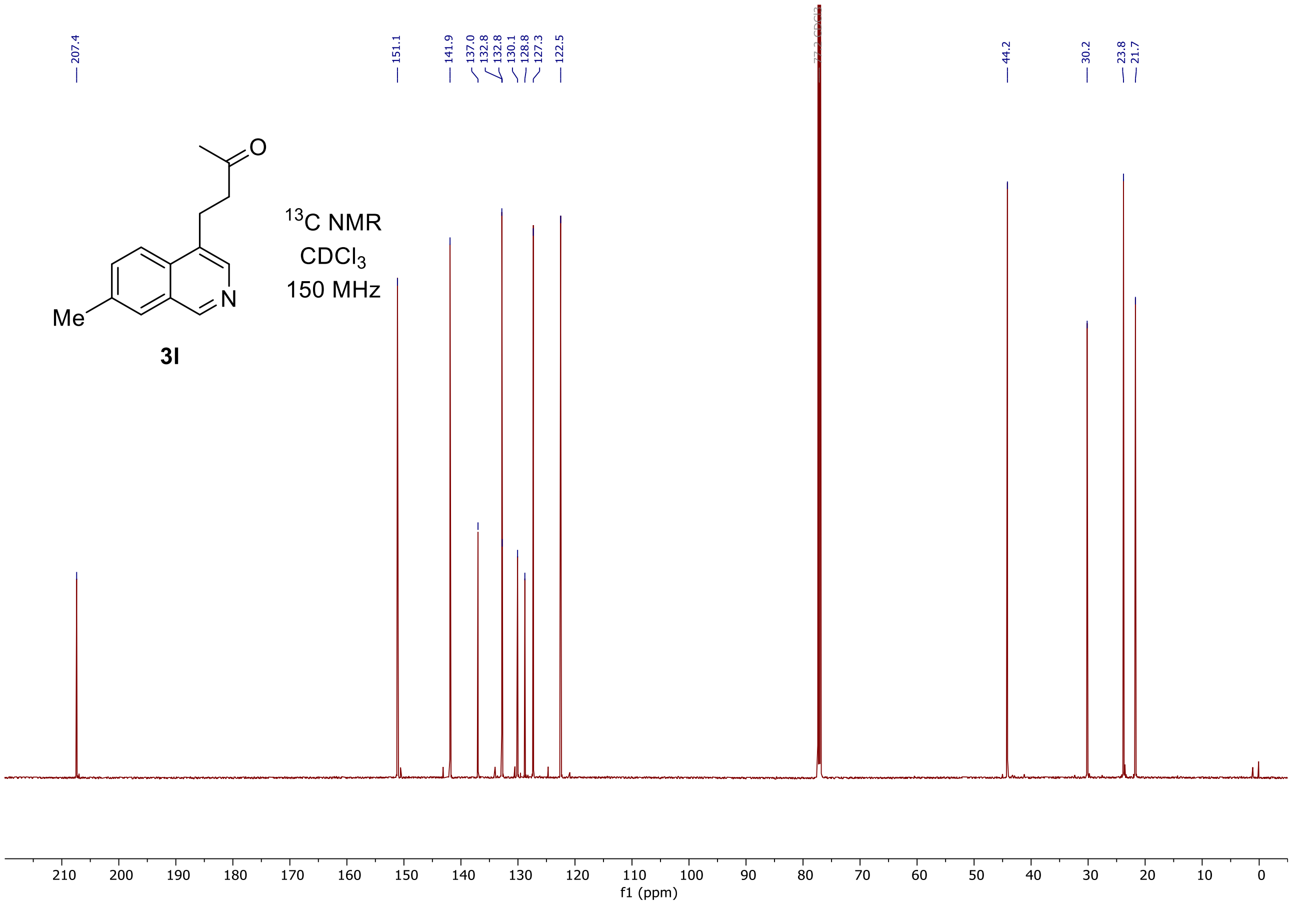
13C NMR
CDCl3
150 MHz
3l
f1 (ppm)

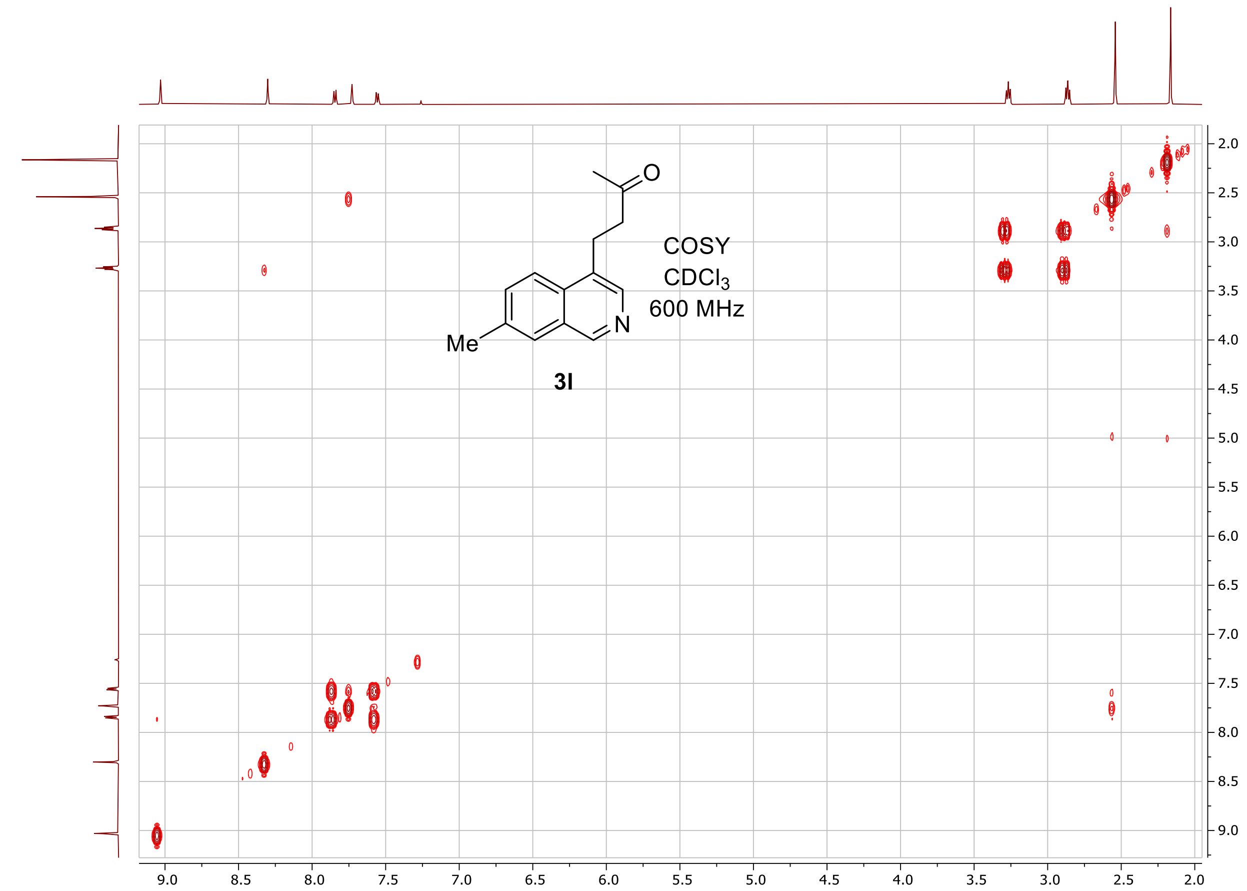
COSY
CDCl3
600 MHz
3l
Me
N
O

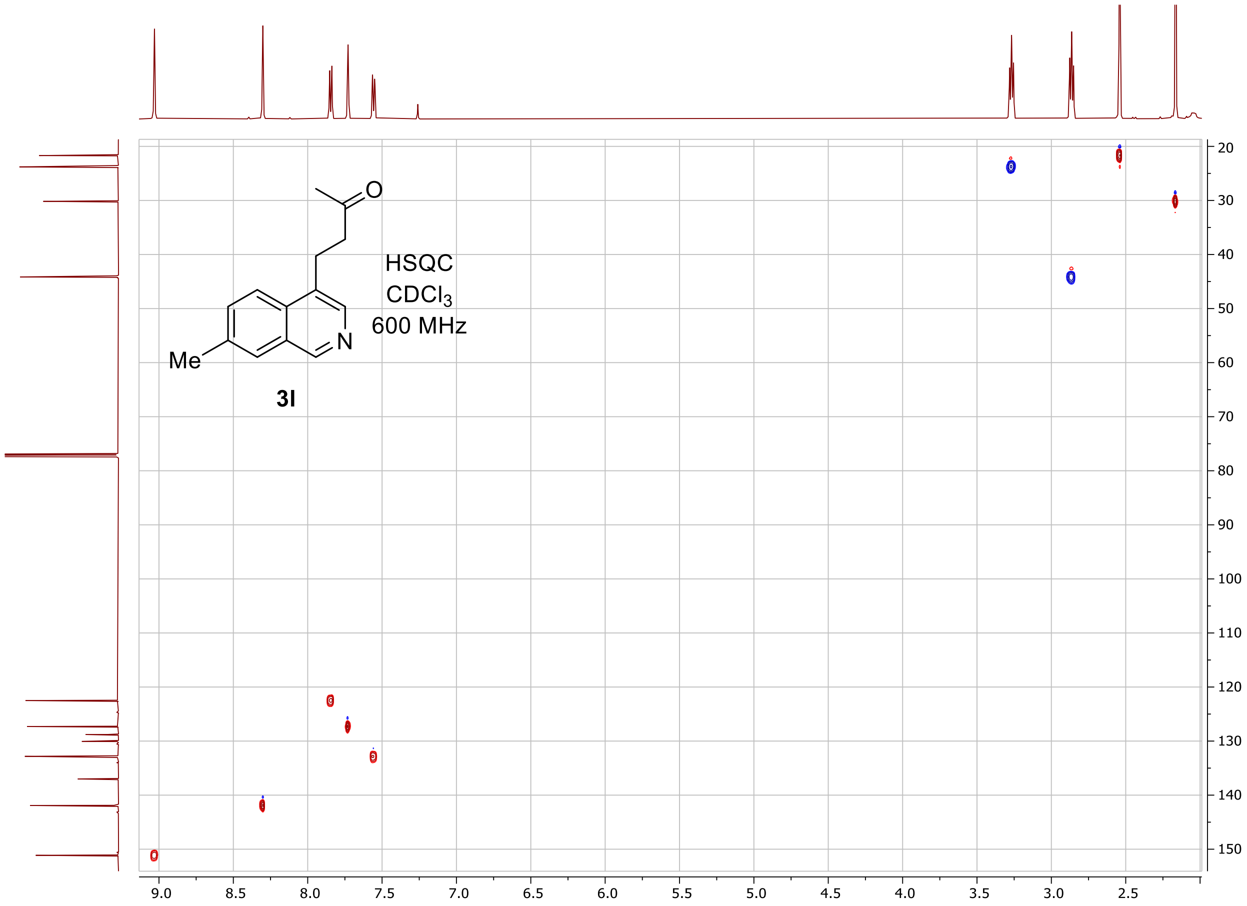
HSQC
CDCl3
600 MHz
3l
Me
N
O

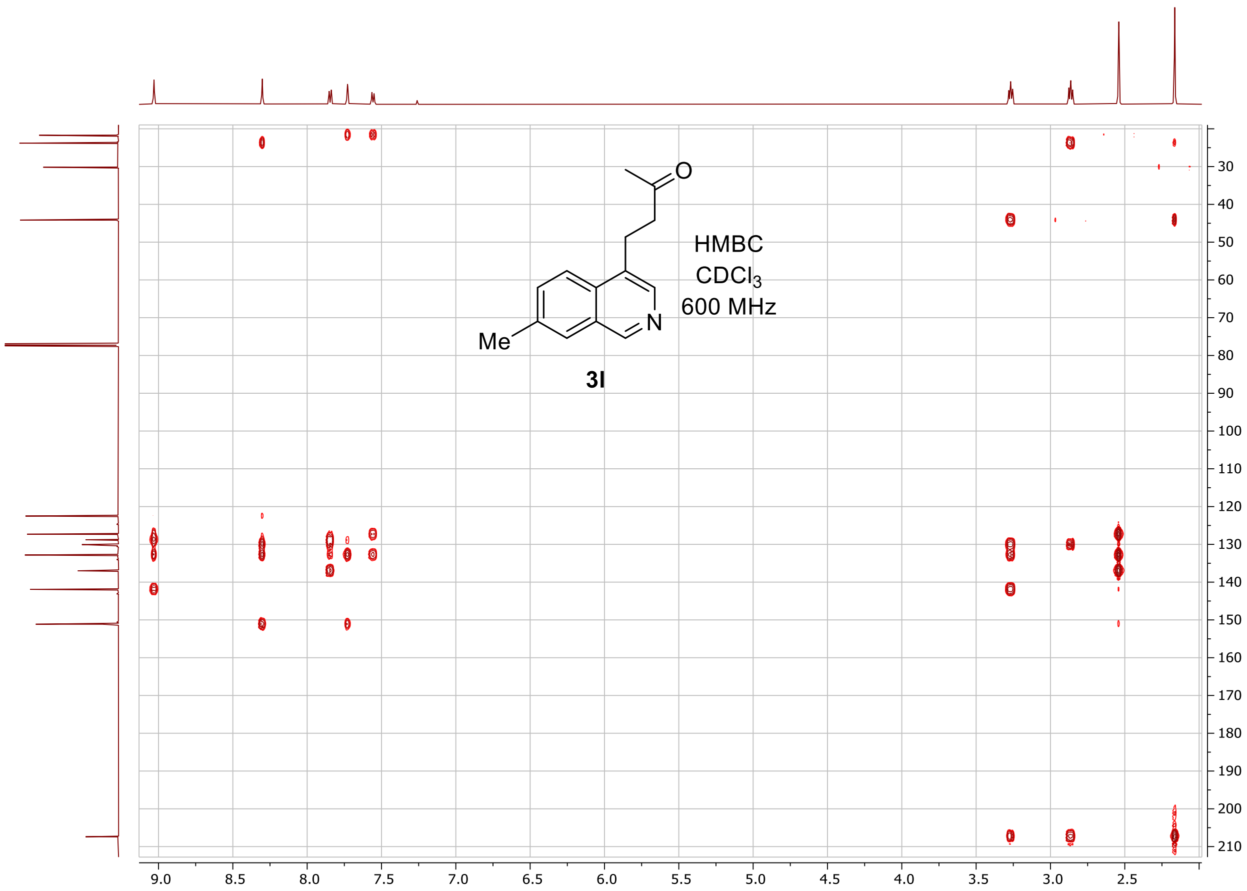

O
Me
N
HMBC
CDCl3
600 MHz
3l
9.0
8.5
8.0
7.5
7.0
6.5
6.0
5.5
5.0
4.5
4.0
3.5
3.0
2.5
30
40
50
60
70
80
90
100
110
120
130
140
150
160
170
180
190
200
210

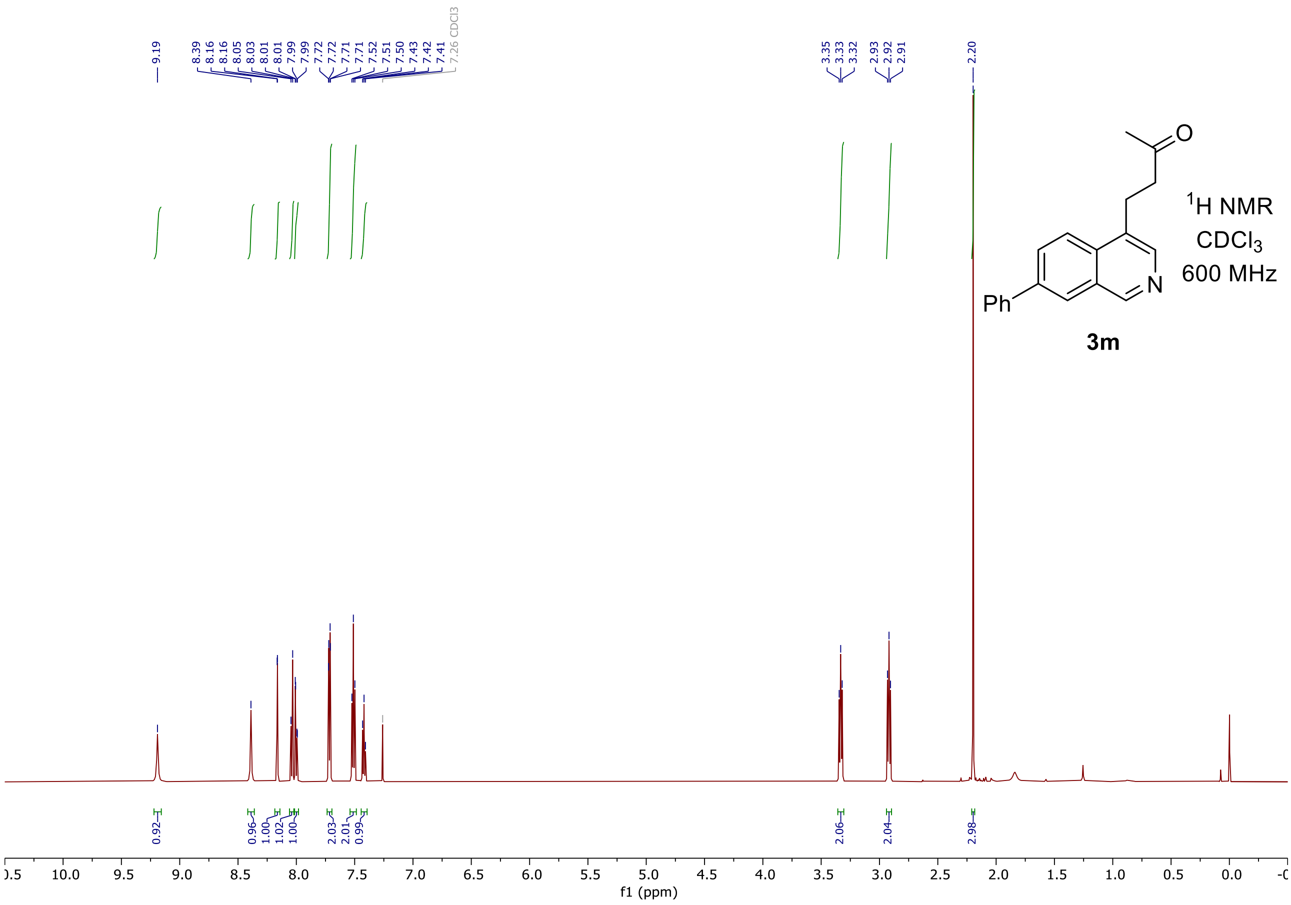
1H NMR
CDCl3
600 MHz
3m
f1 (ppm)

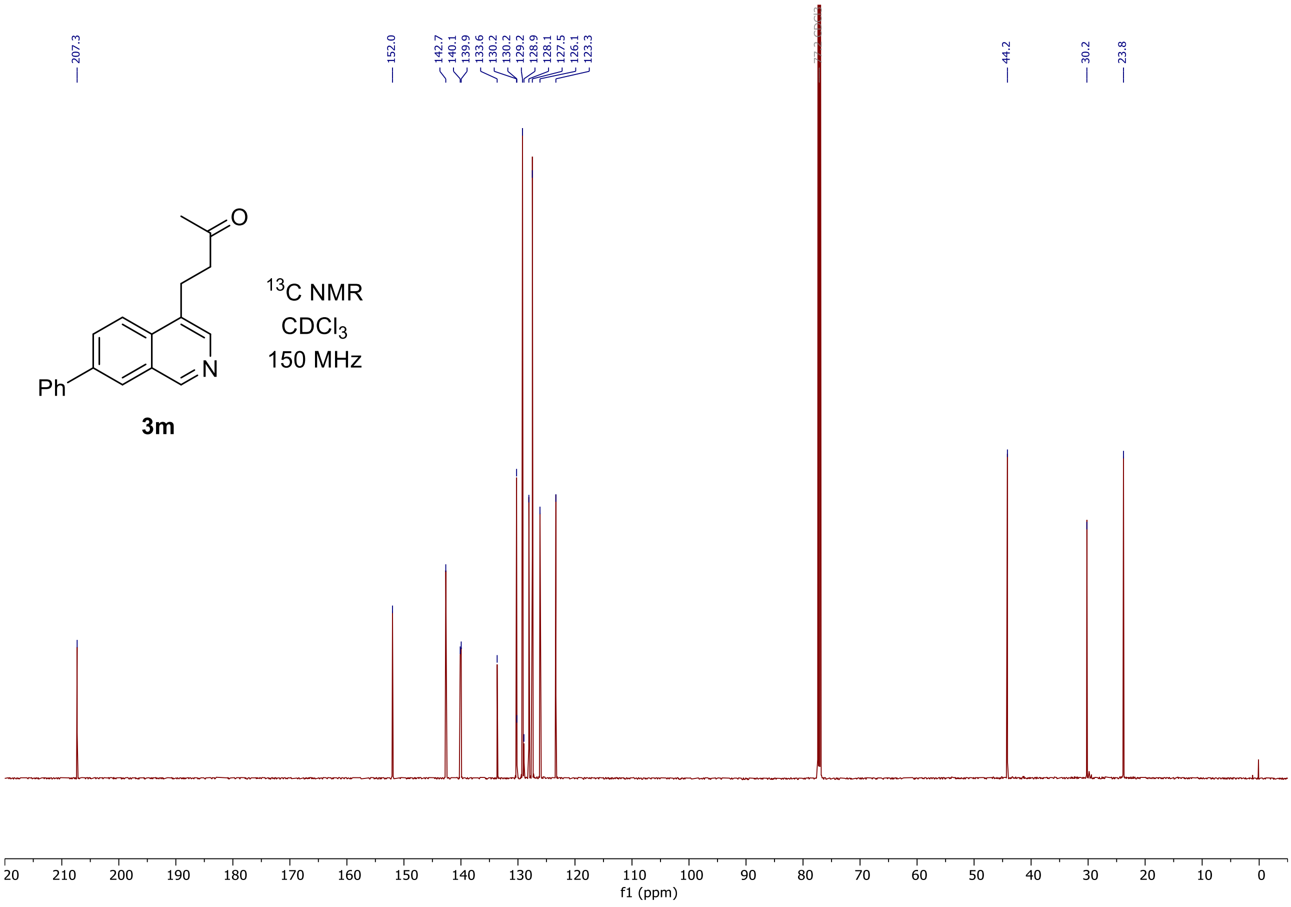
13C NMR
CDCl3
150 MHz
3m
f1 (ppm)

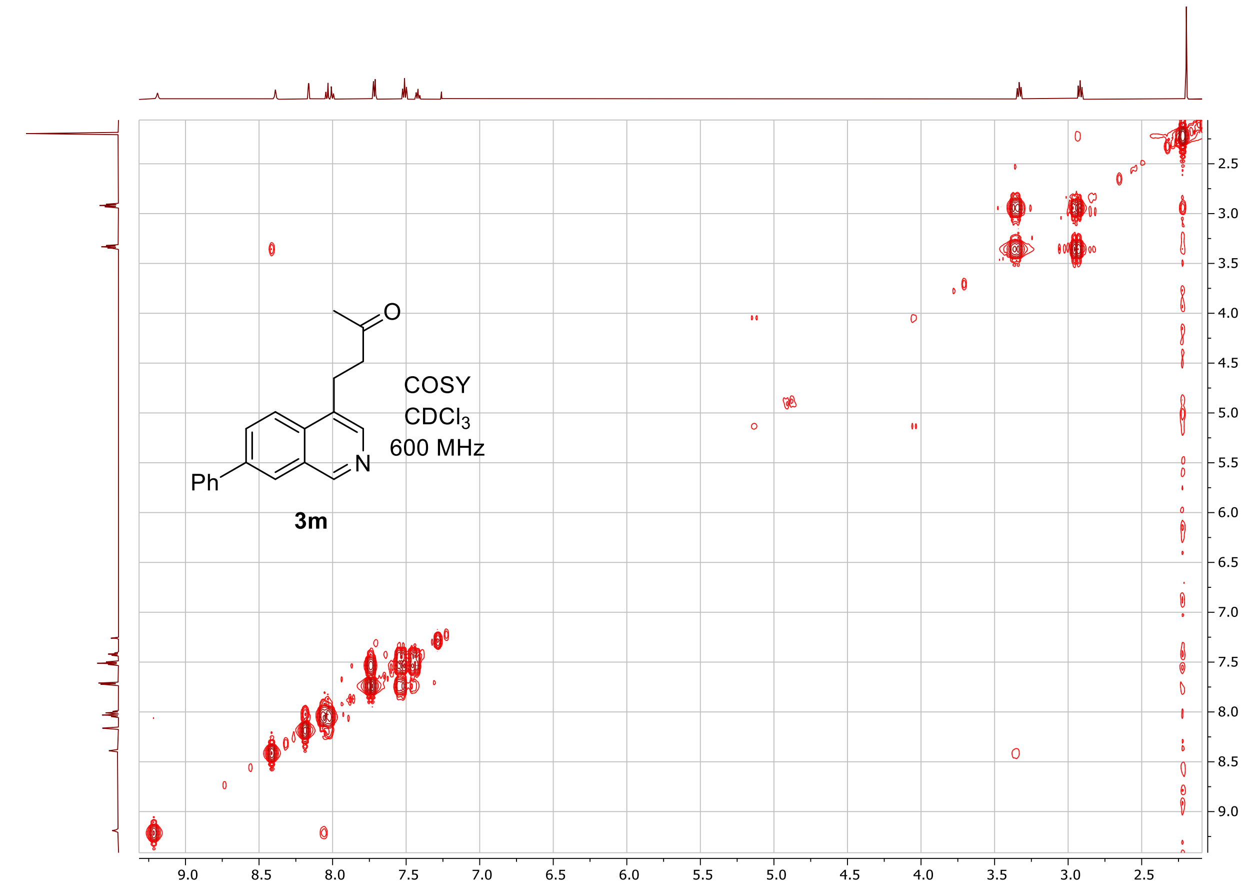
COSY
CDCl3
600 MHz
Ph
N
O
3m

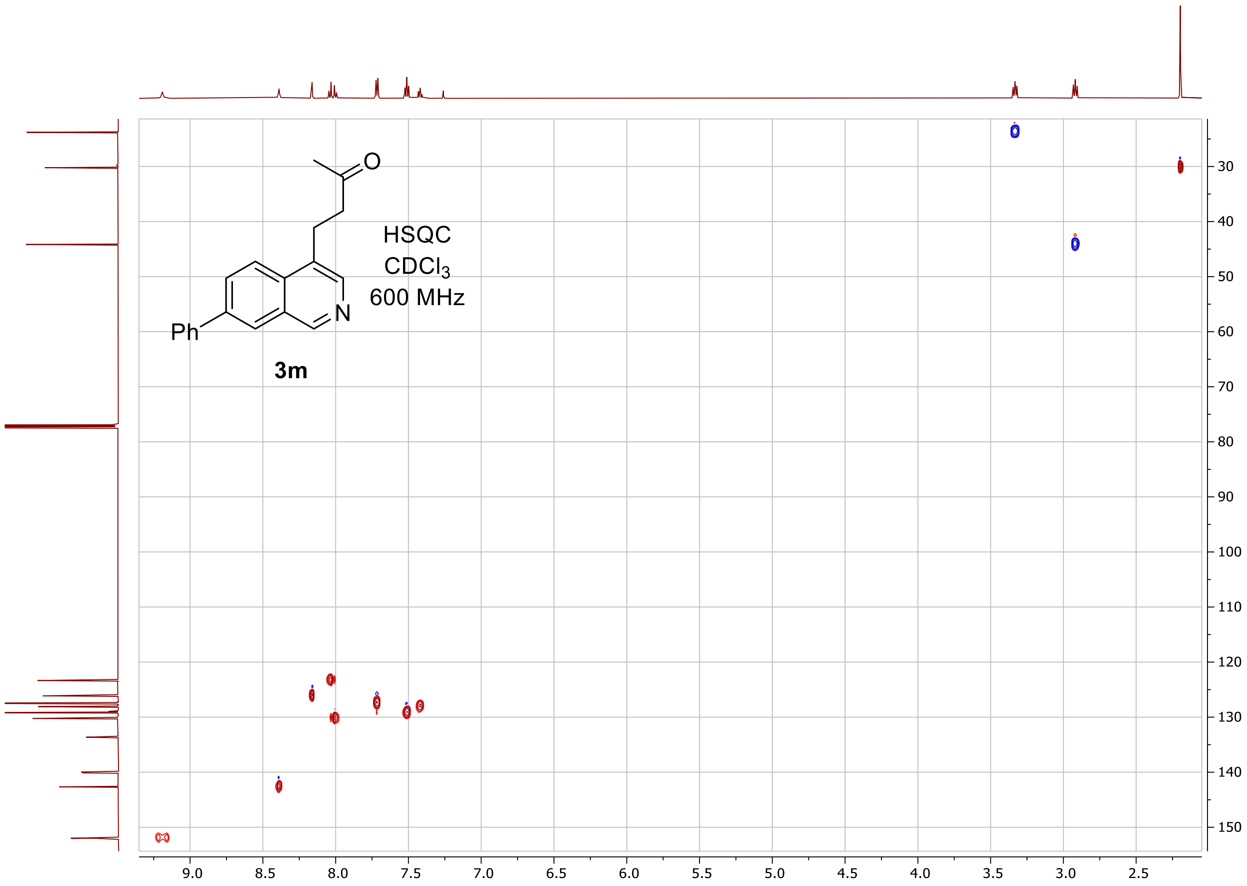
HSQC
CDCl3
600 MHz
Ph
N
O
3m

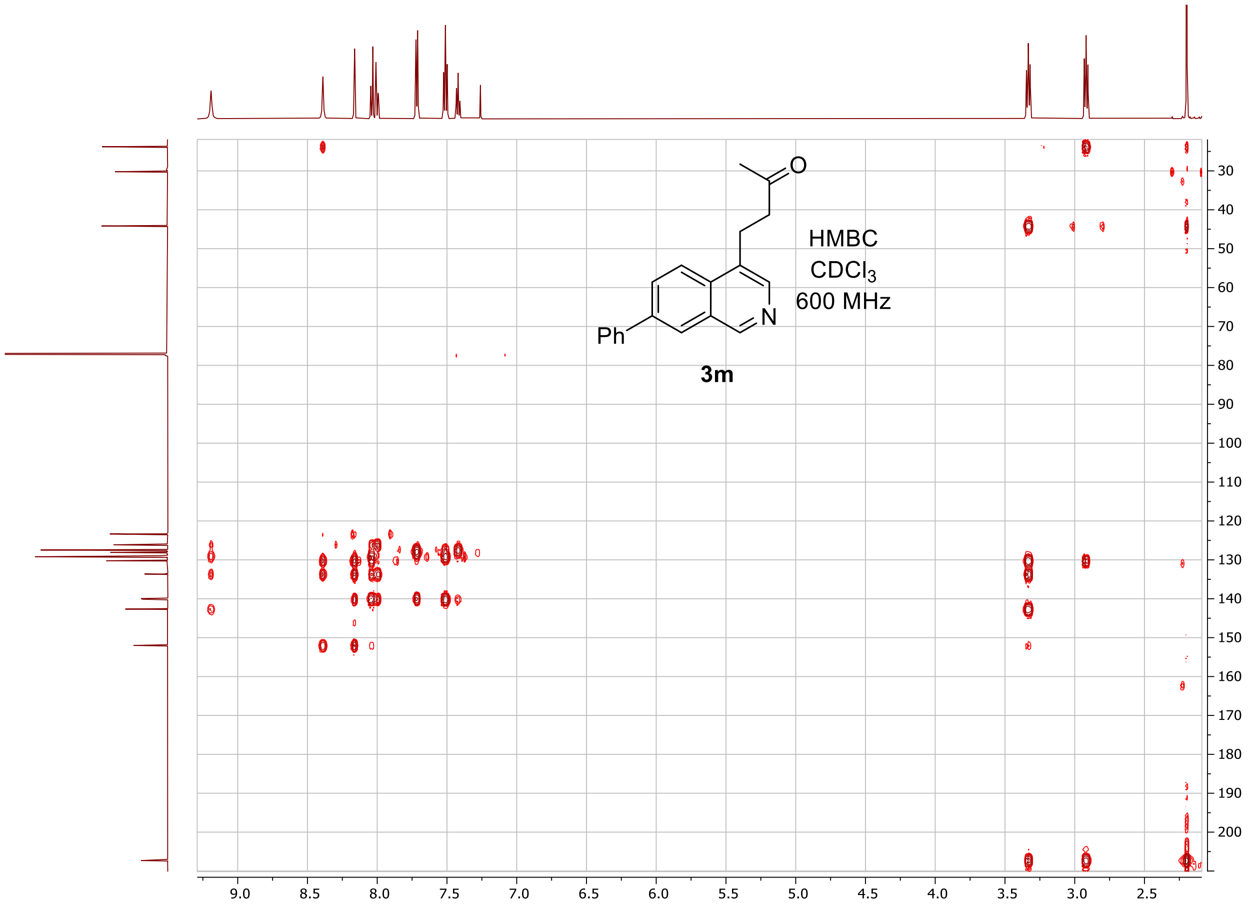

O
HMBC
CDCl$_3$
600 MHz
Ph
N
3m

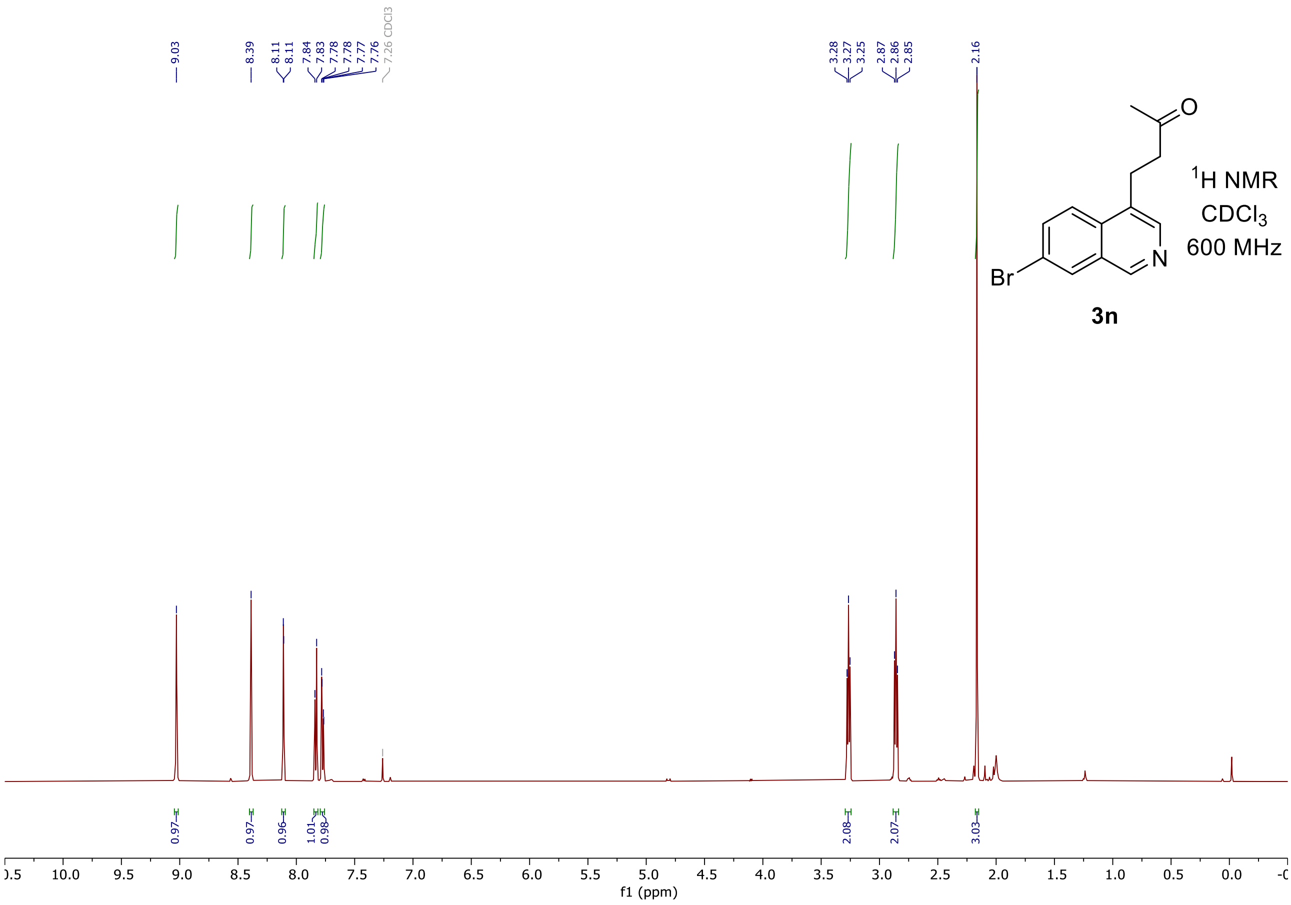

^{1}H NMR
$CDCl_3$
600 MHz
3n
9.03
8.39
8.11
8.11
7.84
7.83
7.78
7.78
7.77
7.76
7.26 CDCl3
3.28
3.27
3.25
2.87
2.86
2.85
2.16
0.97
0.97
0.96
1.01
0.98
2.08
2.07
3.03
f1 (ppm)

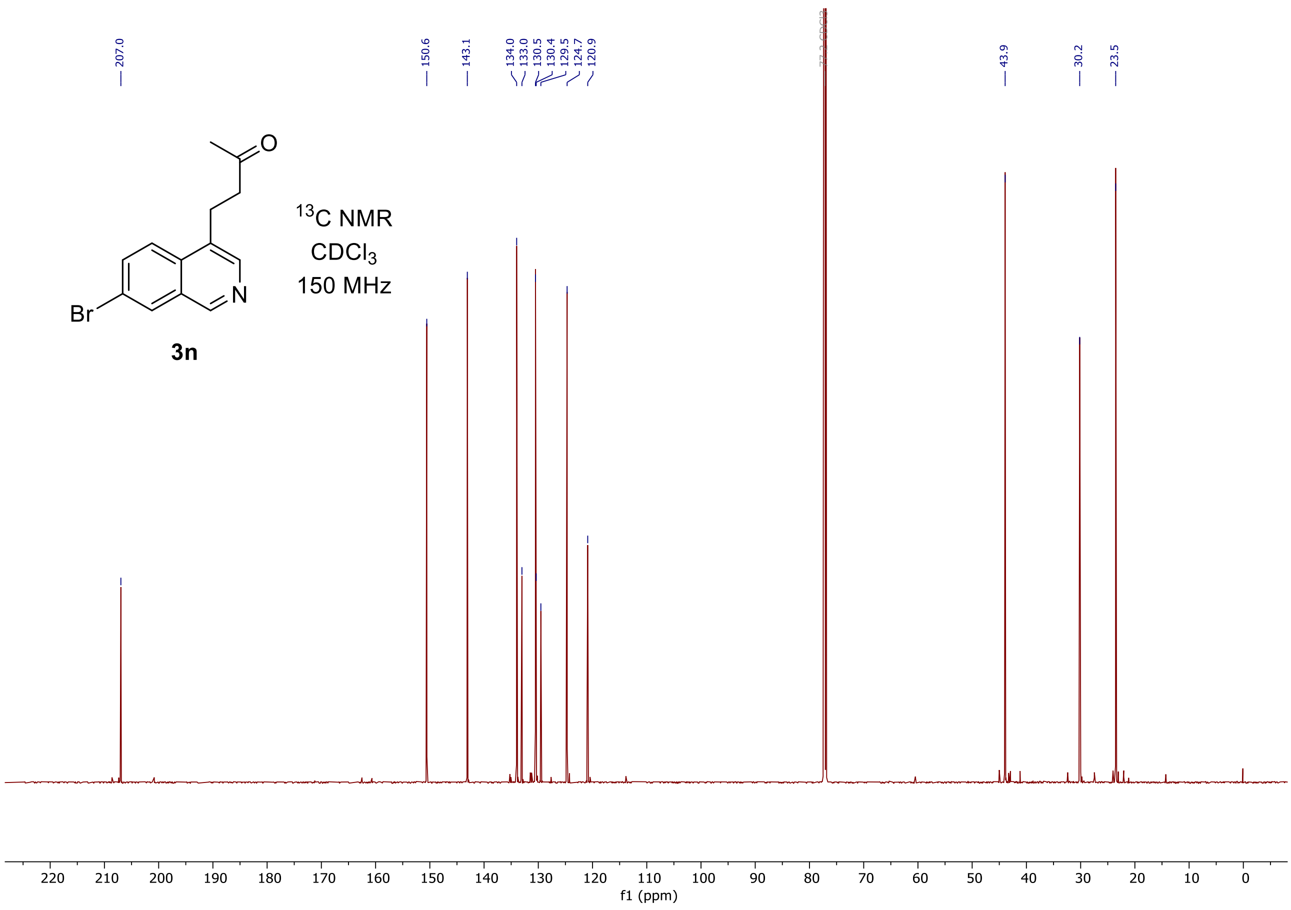

^{13}C NMR
$CDCl_3$
150 MHz
3n
207.0
150.6
143.1
134.0
133.0
130.5
130.4
129.5
124.7
120.9
43.9
30.2
23.5
f1 (ppm)

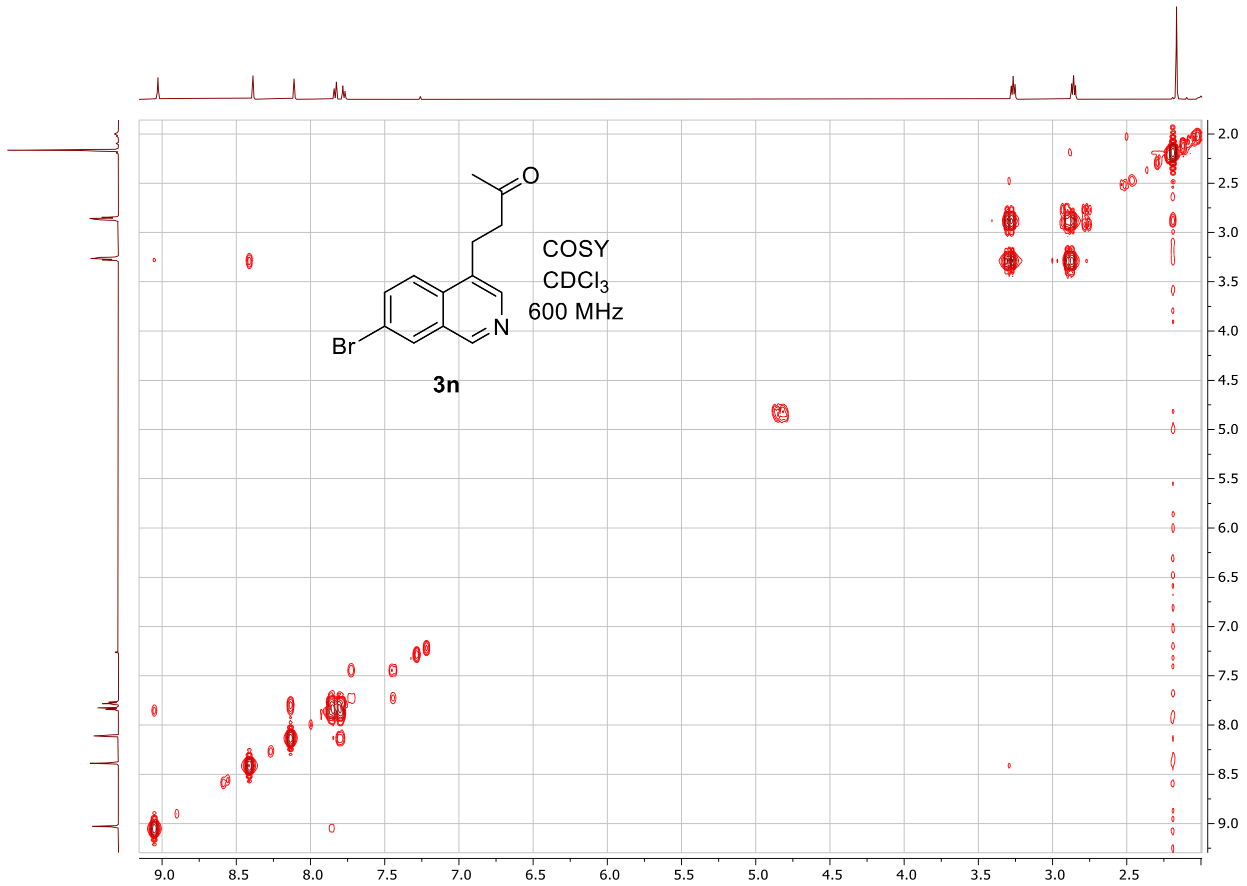
COSY
CDCl3
600 MHz
Br
N
O
3n

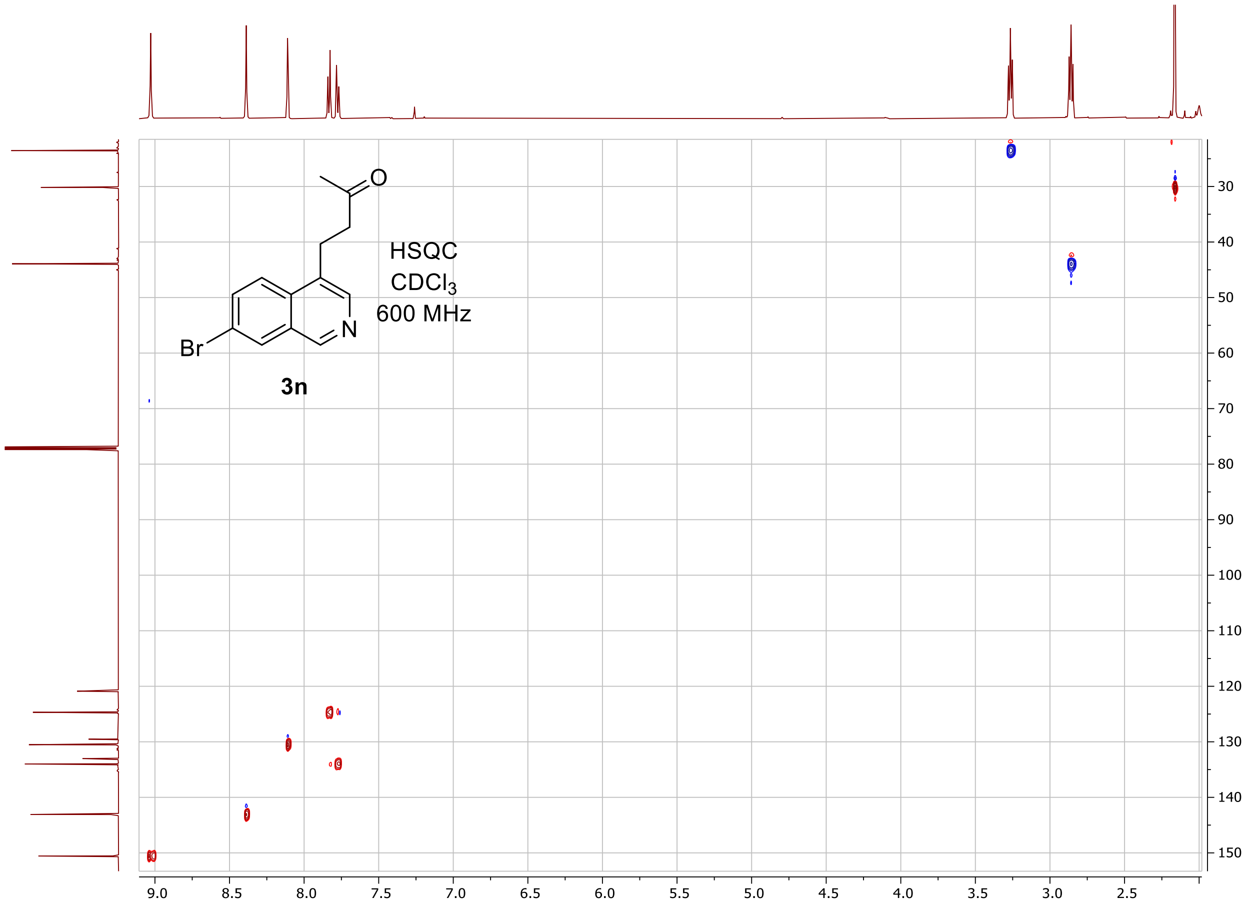
HSQC
CDCl3
600 MHz
Br
N
O
3n

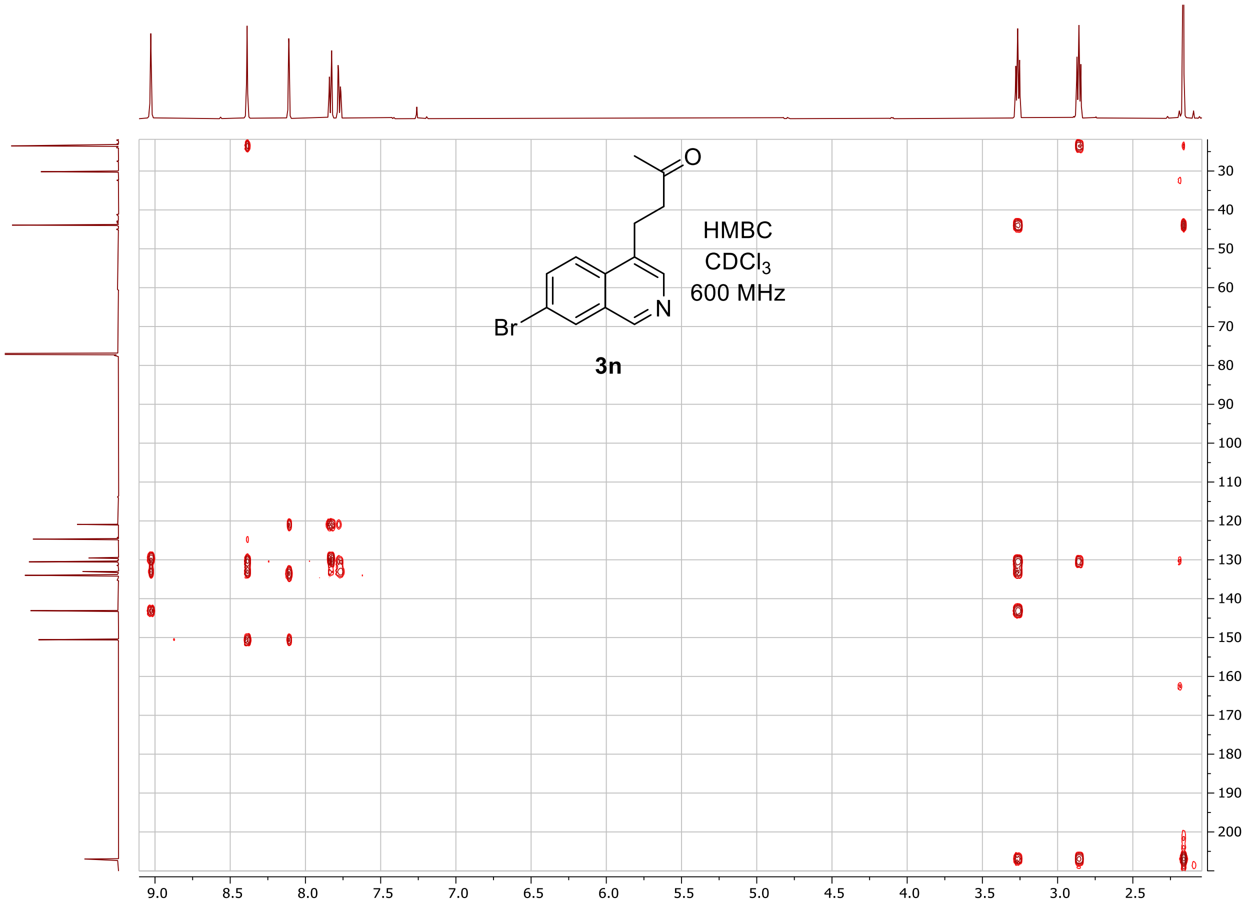

HMBC
$CDCl_3$
600 MHz
Br
N
O
3n

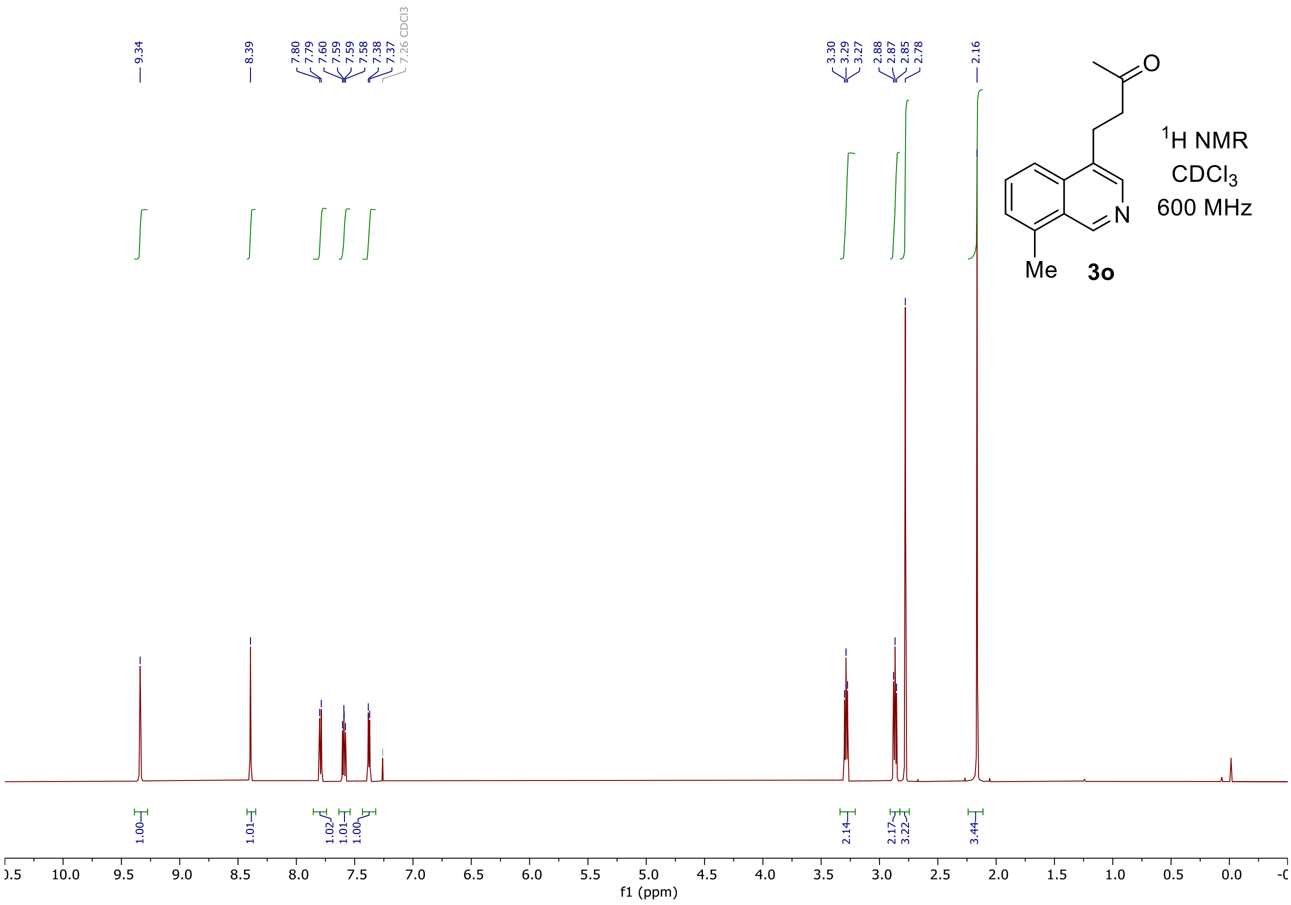
9.34
8.39
7.80
7.79
7.60
7.59
7.59
7.58
7.38
7.37
7.26 CDCl3
3.30
3.29
3.27
2.88
2.87
2.85
2.78
2.16
1H NMR
CDCl3
600 MHz
N
O
Me
3o
1.00
1.01
1.02
1.01
1.00
2.14
2.17
3.22
3.44
f1 (ppm)

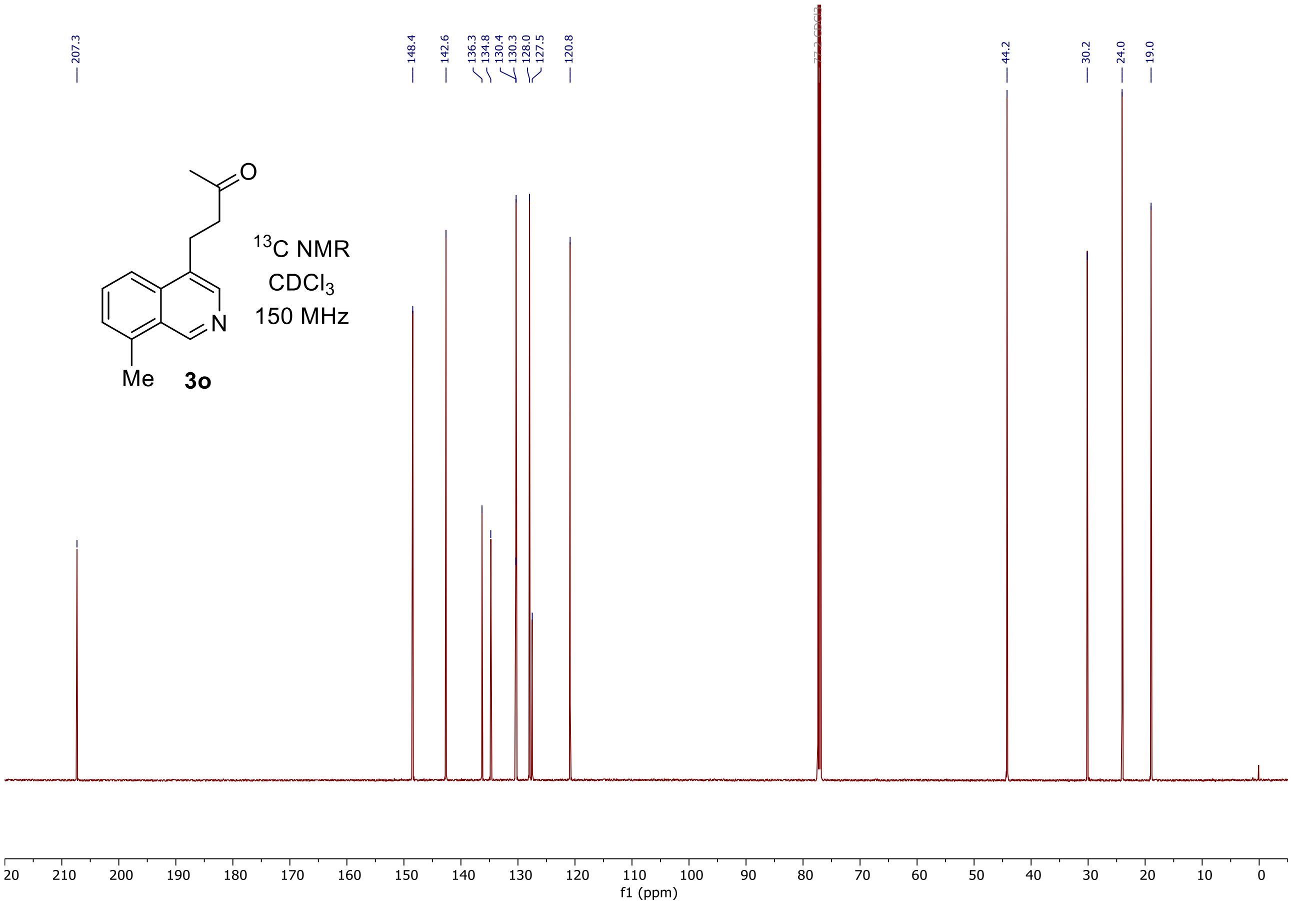
207.3
148.4
142.6
136.3
134.8
130.4
130.3
128.0
127.5
120.8
44.2
30.2
24.0
19.0
13C NMR
CDCl3
150 MHz
N
O
Me
3o
f1 (ppm)

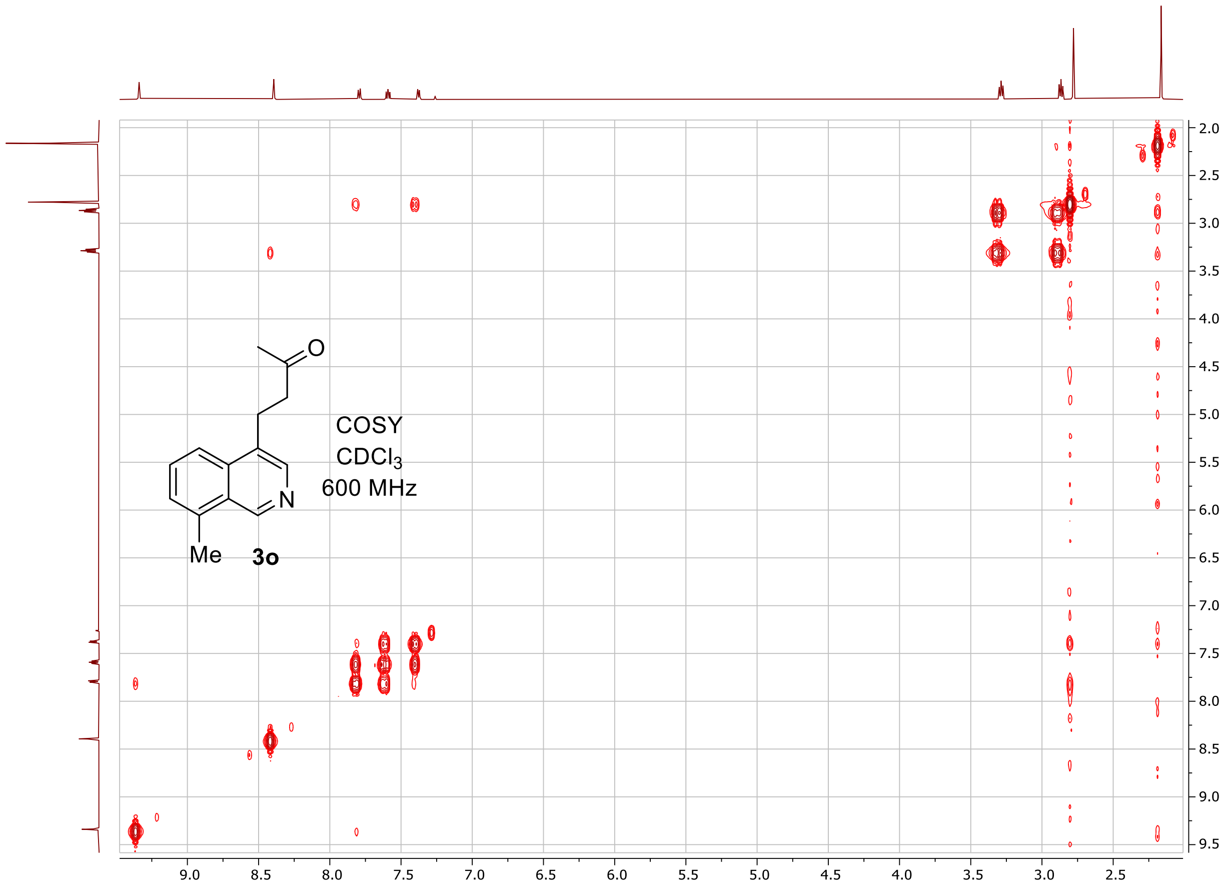
COSY
CDCl3
600 MHz
Me
3o

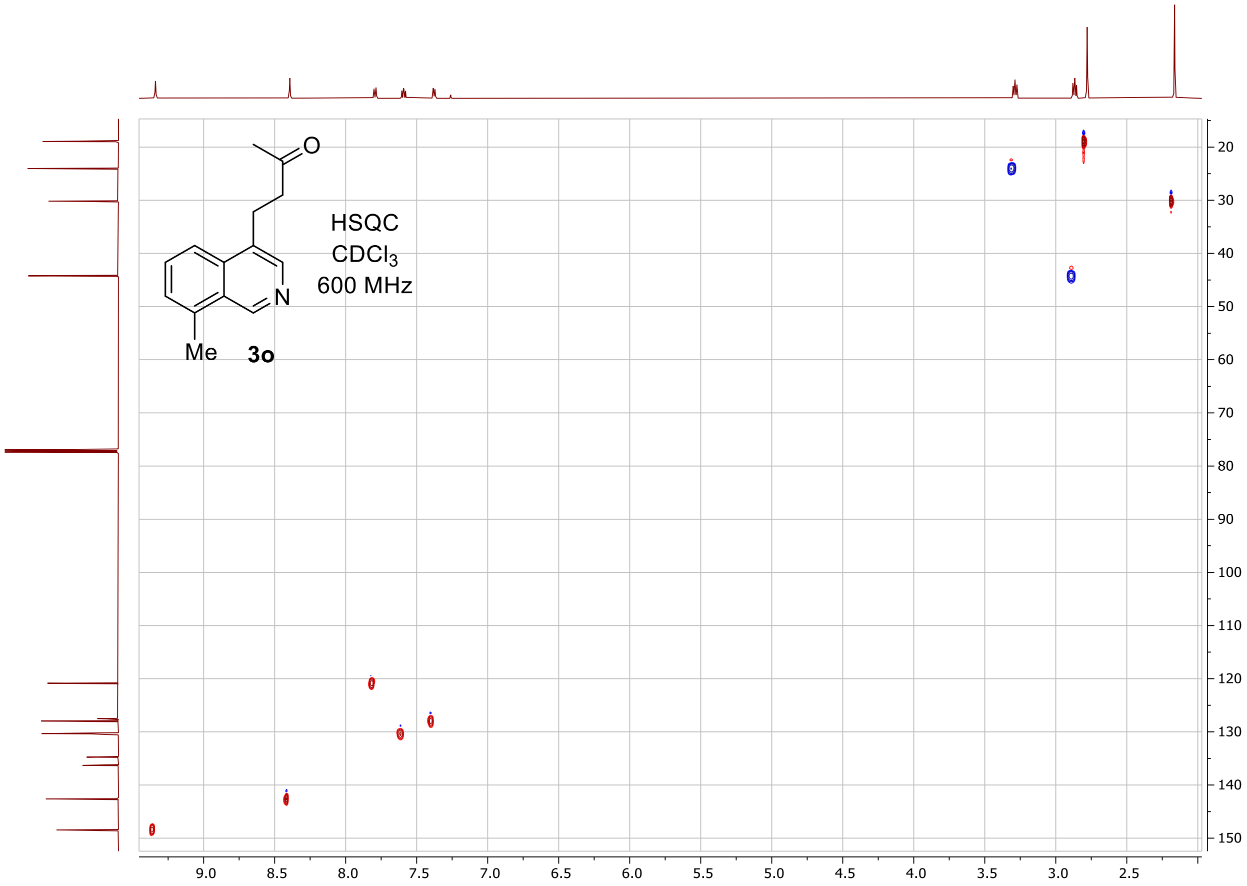
HSQC
CDCl3
600 MHz
Me
3o

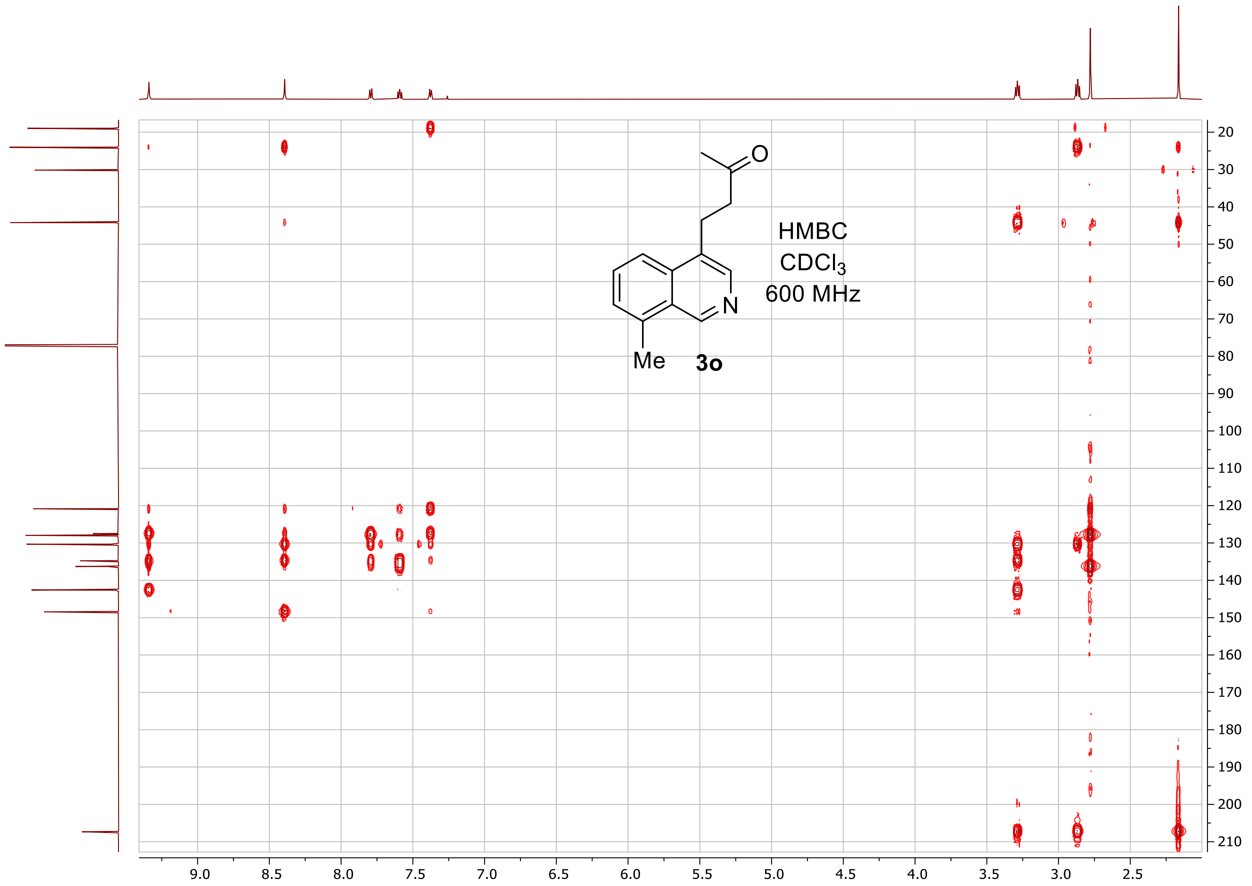
HMBC
CDCl3
600 MHz
Me
3o
O
N

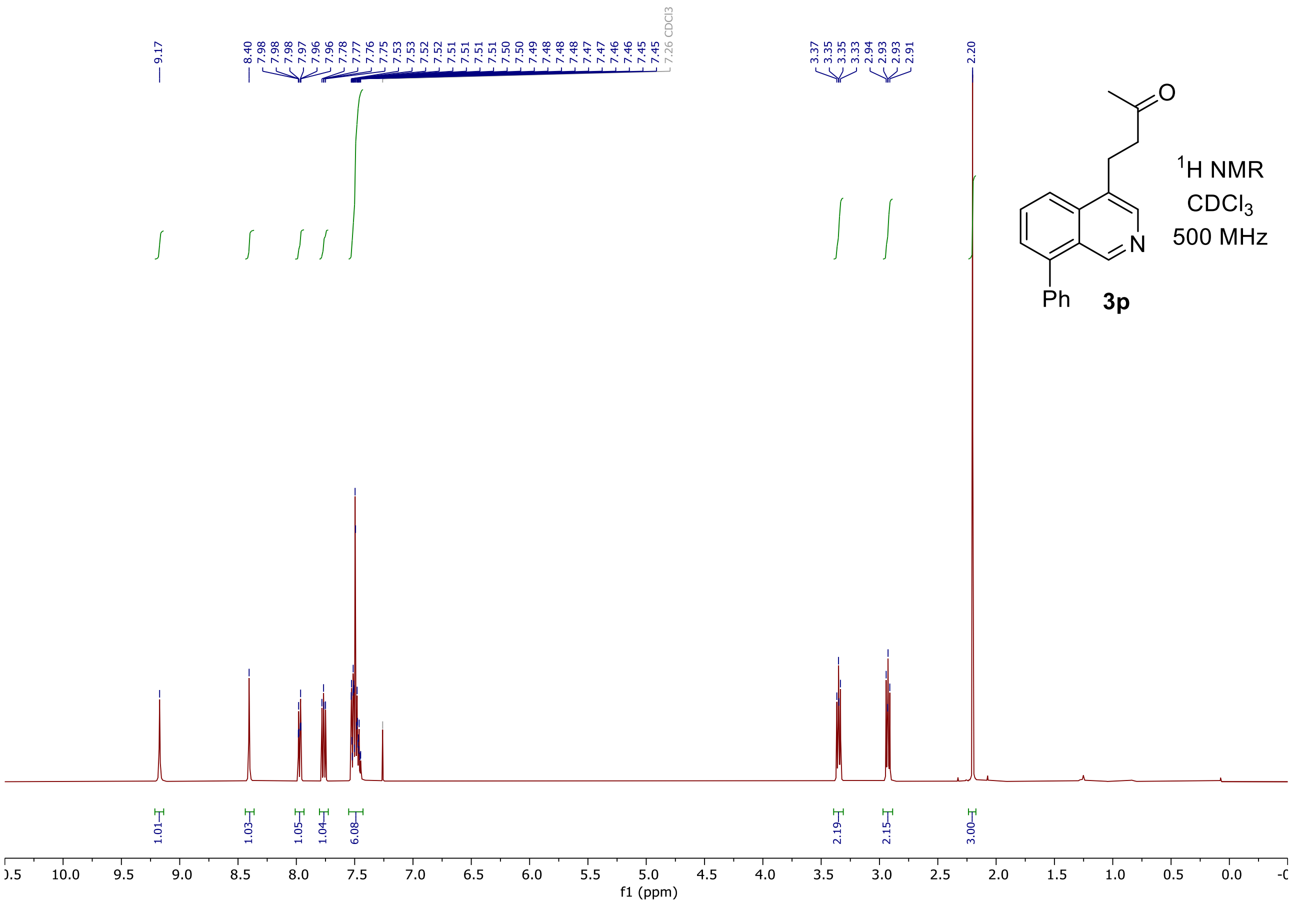

^{1}H NMR
$CDCl_3$
500 MHz
3p
f1 (ppm)

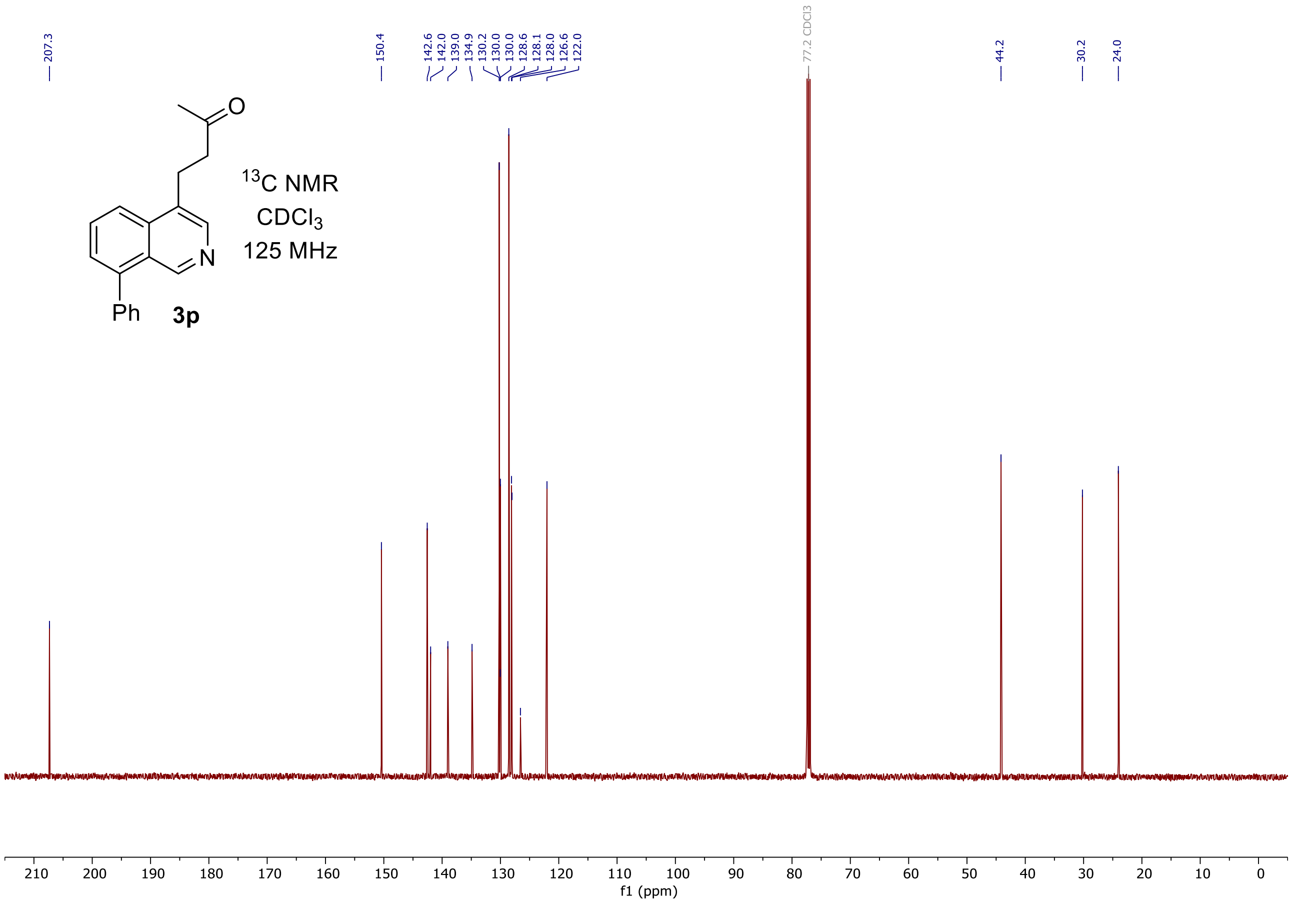

^{13}C NMR
$CDCl_3$
125 MHz
3p
f1 (ppm)

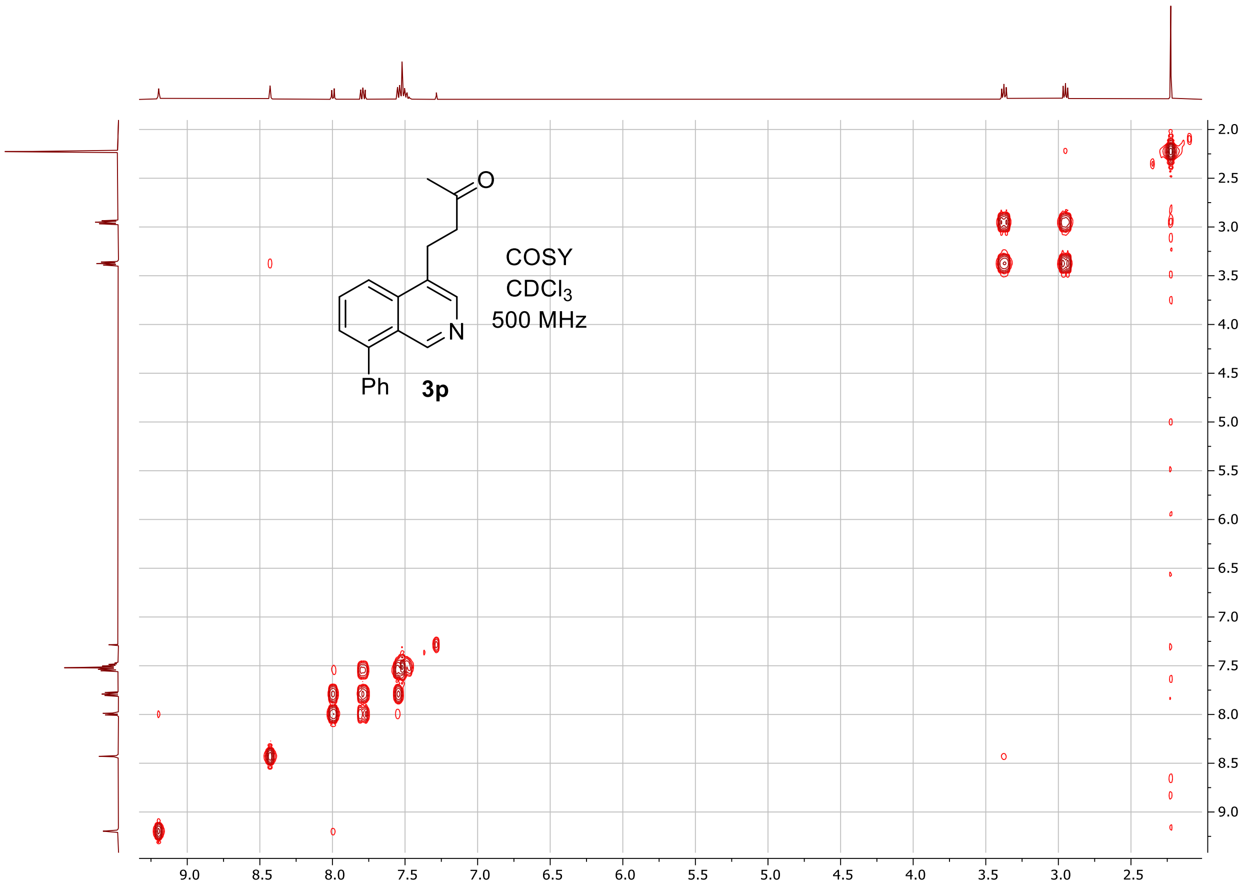

COSY
$CDCl_3$
500 MHz
Ph
3p

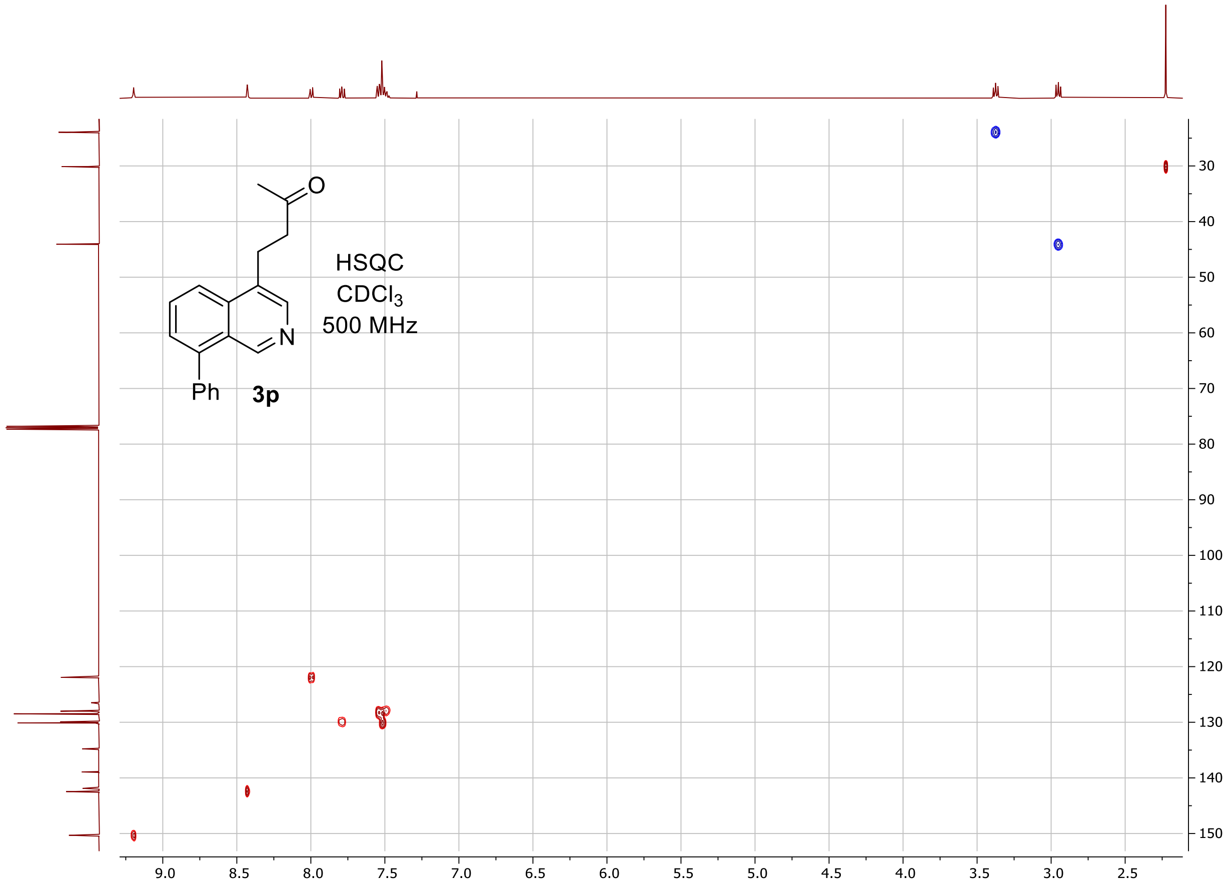

HSQC
$CDCl_3$
500 MHz
Ph
3p

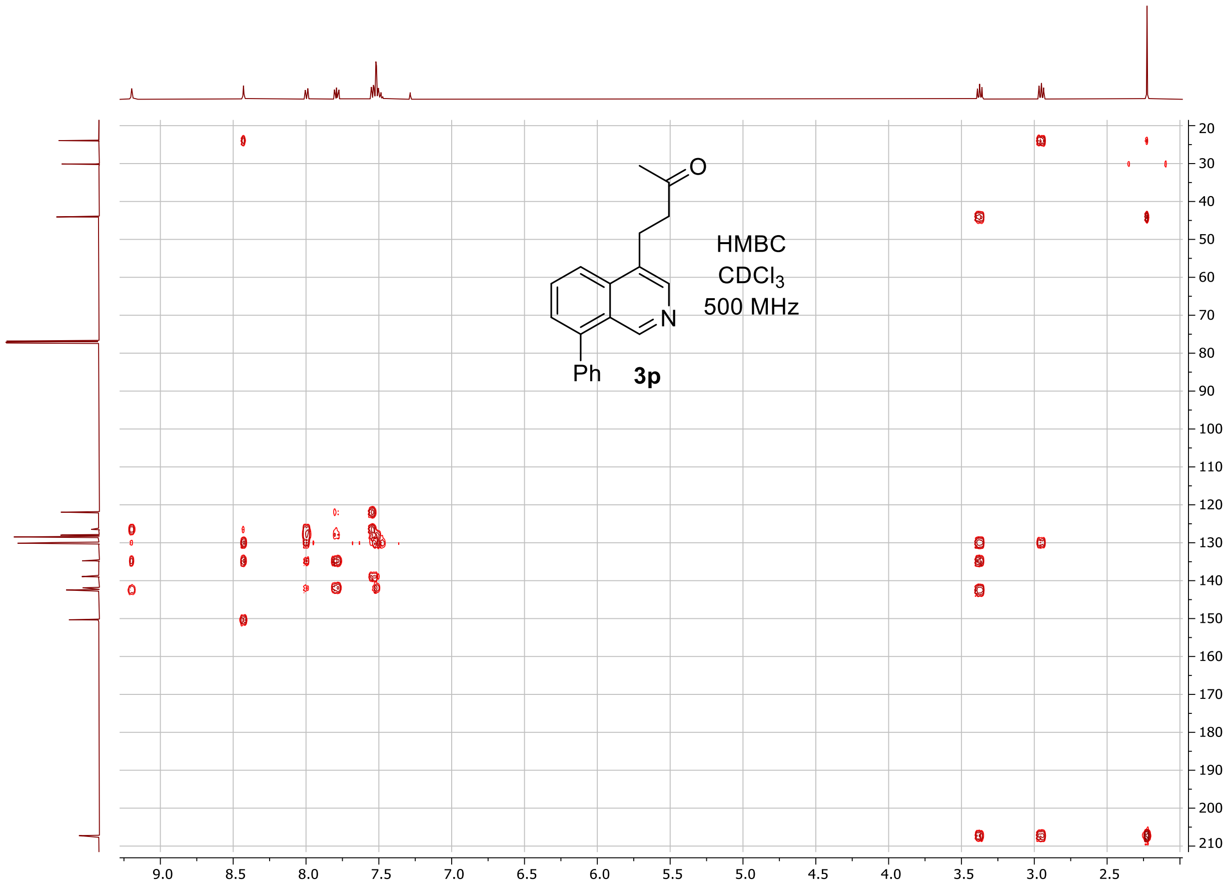

O
N
Ph
3p
HMBC
CDCl3
500 MHz

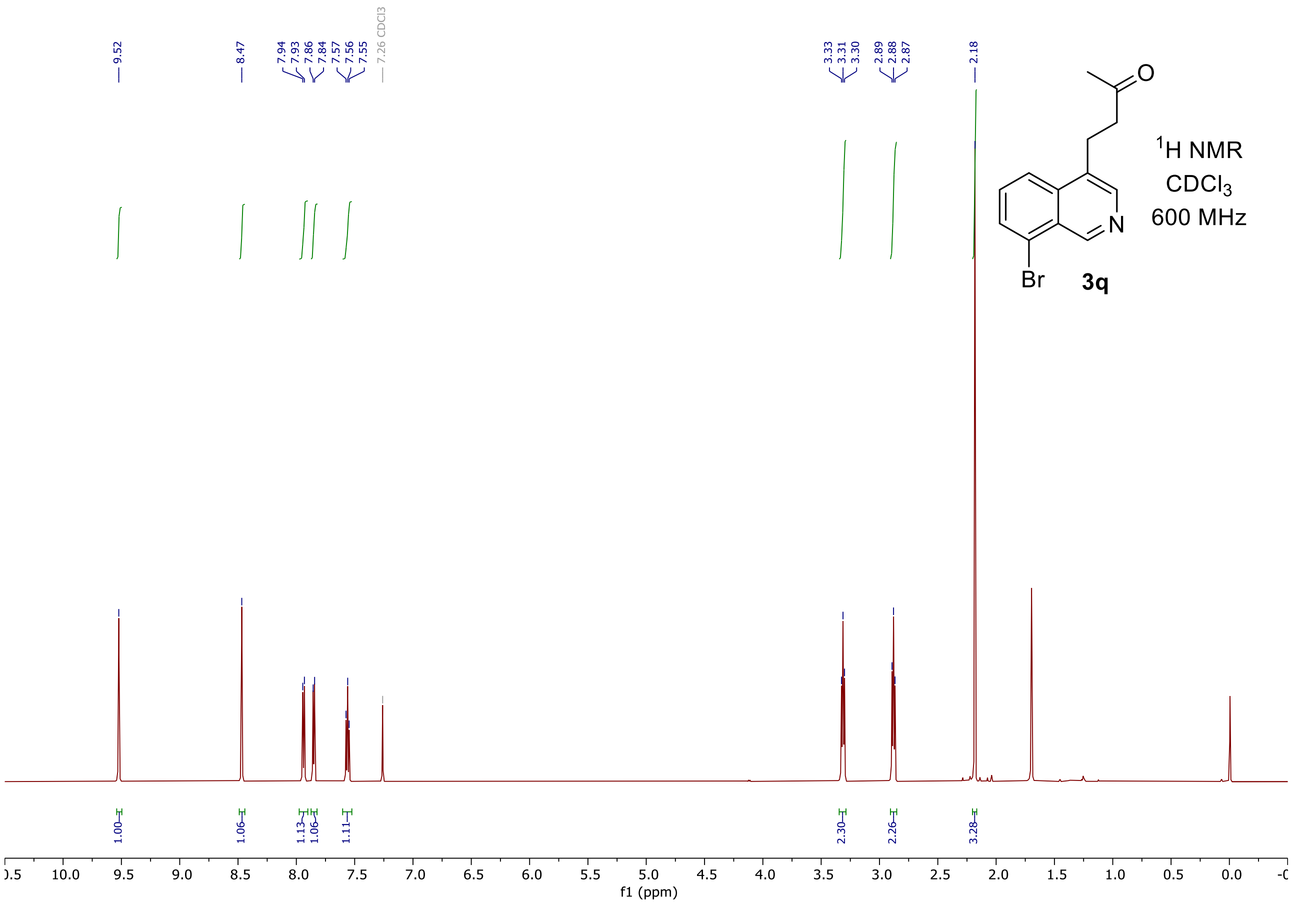

9.52
8.47
7.94
7.93
7.86
7.84
7.57
7.56
7.55
7.26 CDCl3
3.33
3.31
3.30
2.89
2.88
2.87
2.18
O
N
Br
3q
^{1}H NMR
$CDCl_3$
600 MHz
1.00
1.06
1.13
1.06
1.11
2.30
2.26
3.28
f1 (ppm)

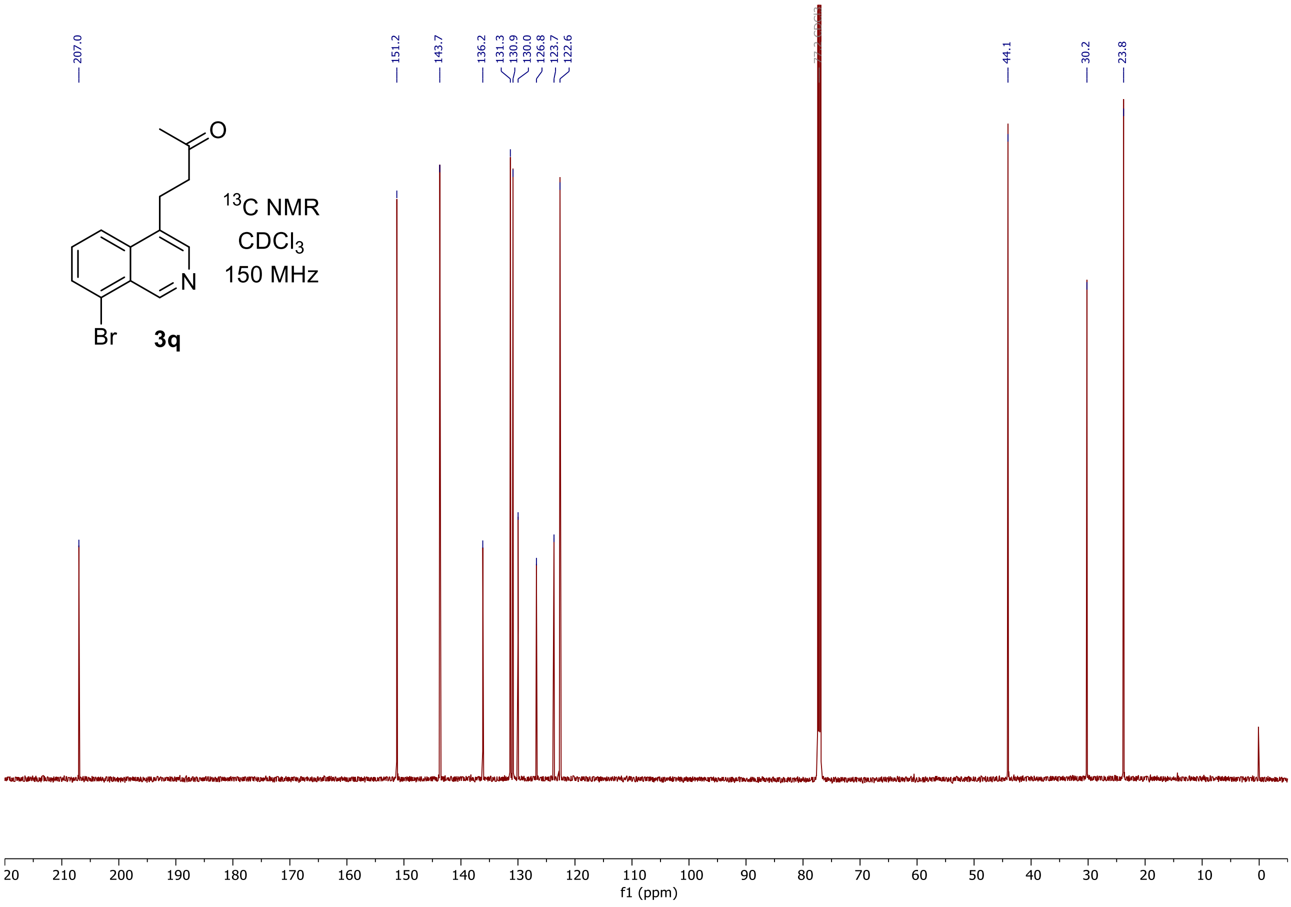

207.0
151.2
143.7
136.2
131.3
130.9
130.0
126.8
123.7
122.6
44.1
30.2
23.8
O
N
Br
3q
^{13}C NMR
$CDCl_3$
150 MHz
f1 (ppm)

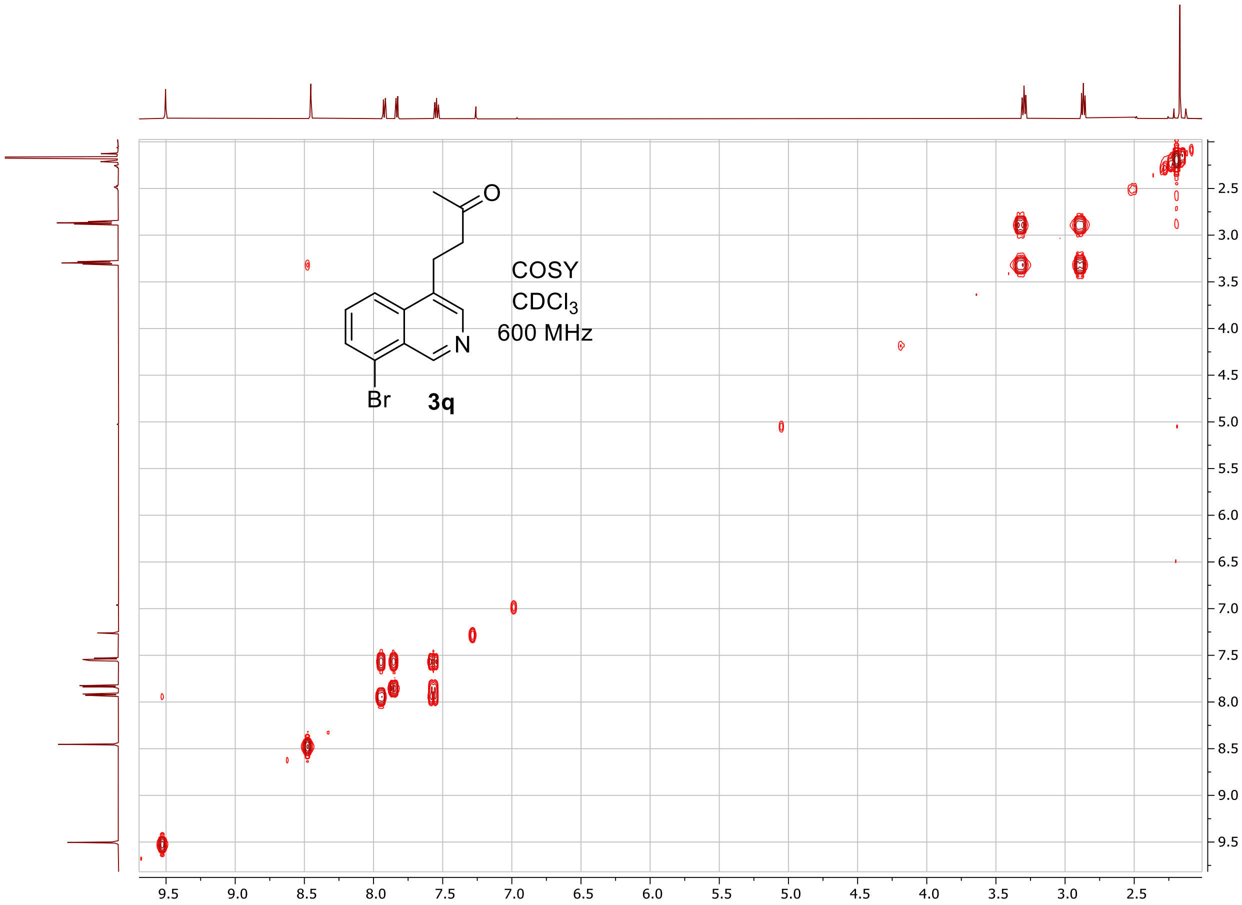
COSY
CDCl$_3$
600 MHz
3q

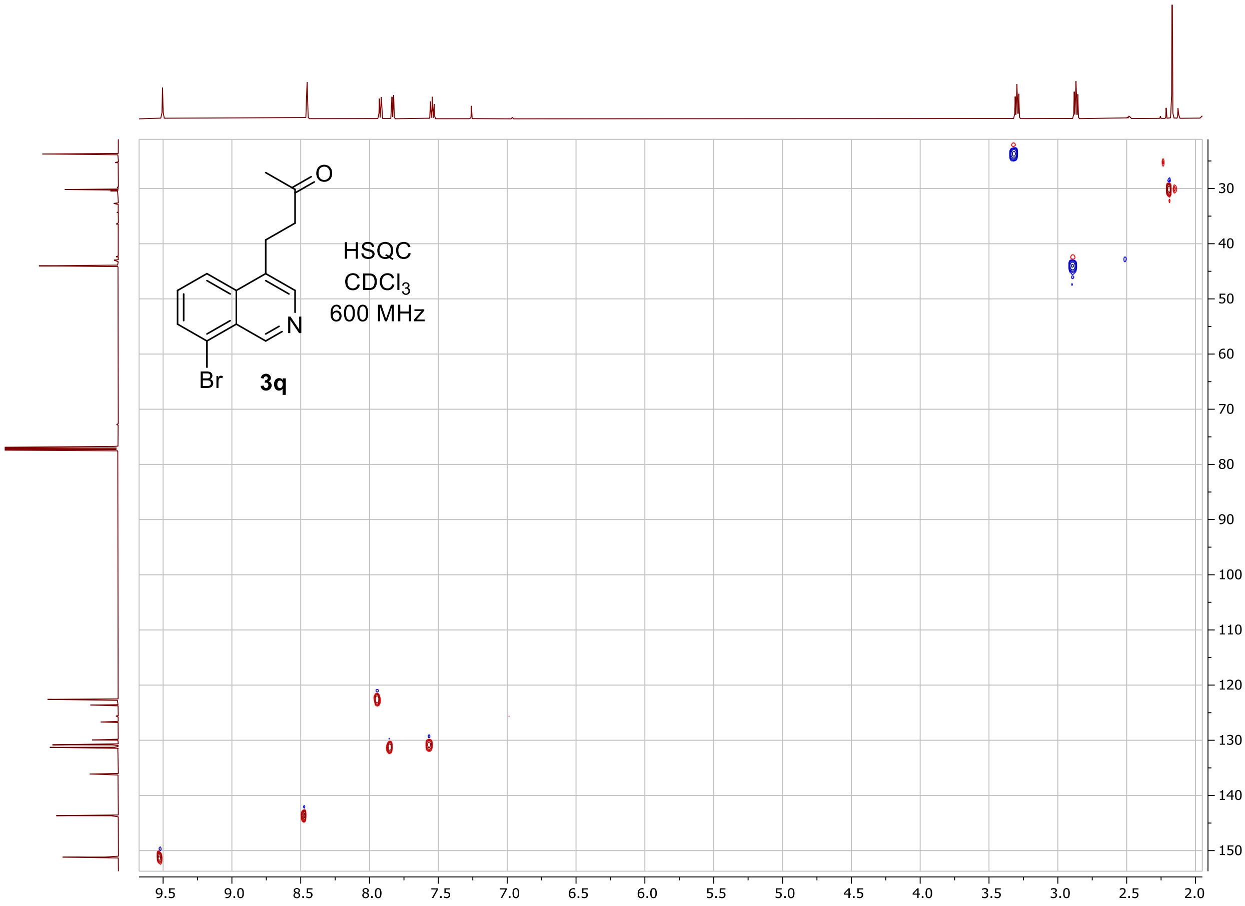
HSQC
CDCl$_3$
600 MHz
3q

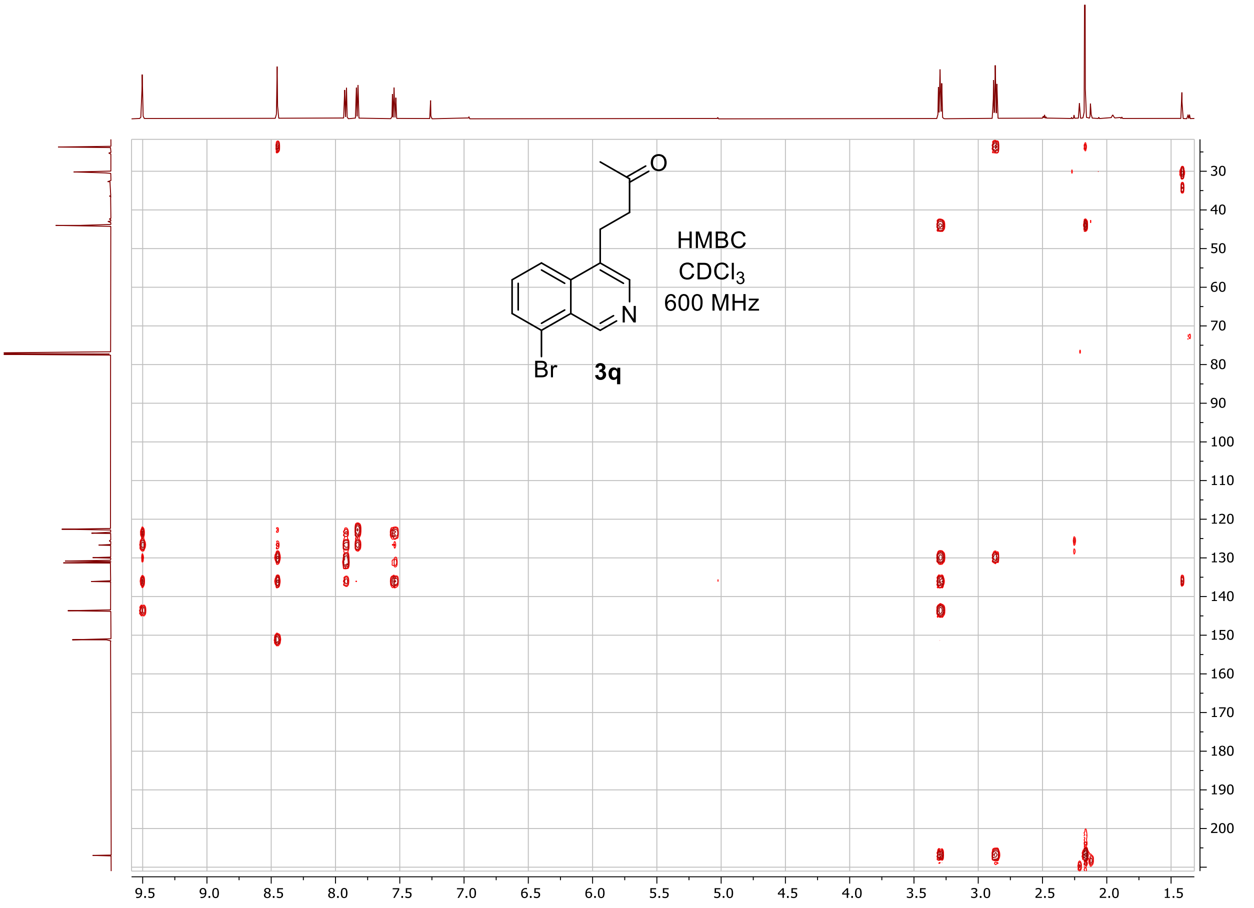

HMBC
$CDCl_3$
600 MHz
Br
3q
O
N

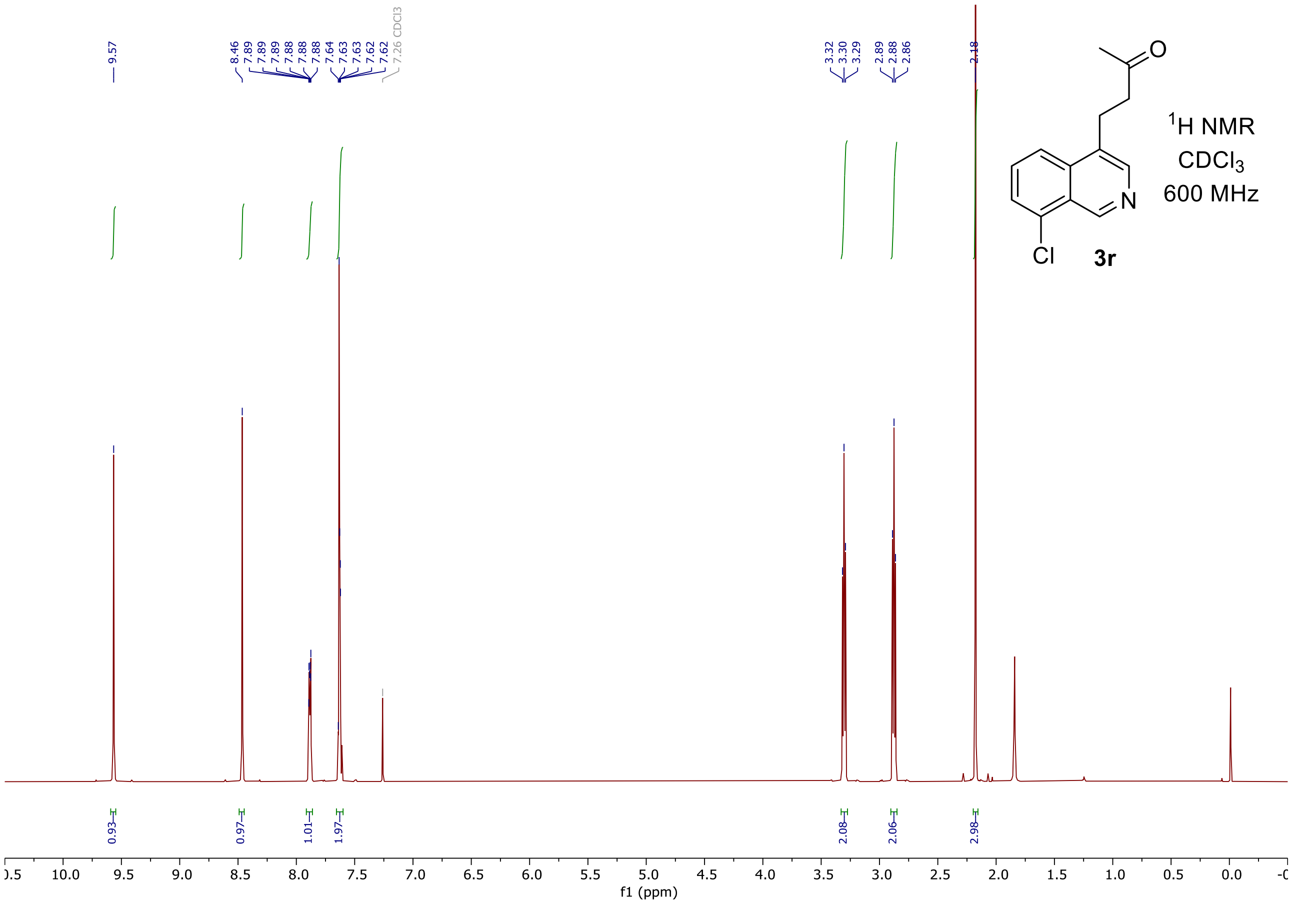
9.57
8.46
7.89
7.89
7.89
7.88
7.88
7.88
7.64
7.63
7.63
7.62
7.62
7.26 CDCl3
3.32
3.30
3.29
2.89
2.88
2.86
2.18
^{1}H NMR
$CDCl_3$
600 MHz
N
O
Cl
3r
0.93
0.97
1.01
1.97
2.08
2.06
2.98
f1 (ppm)

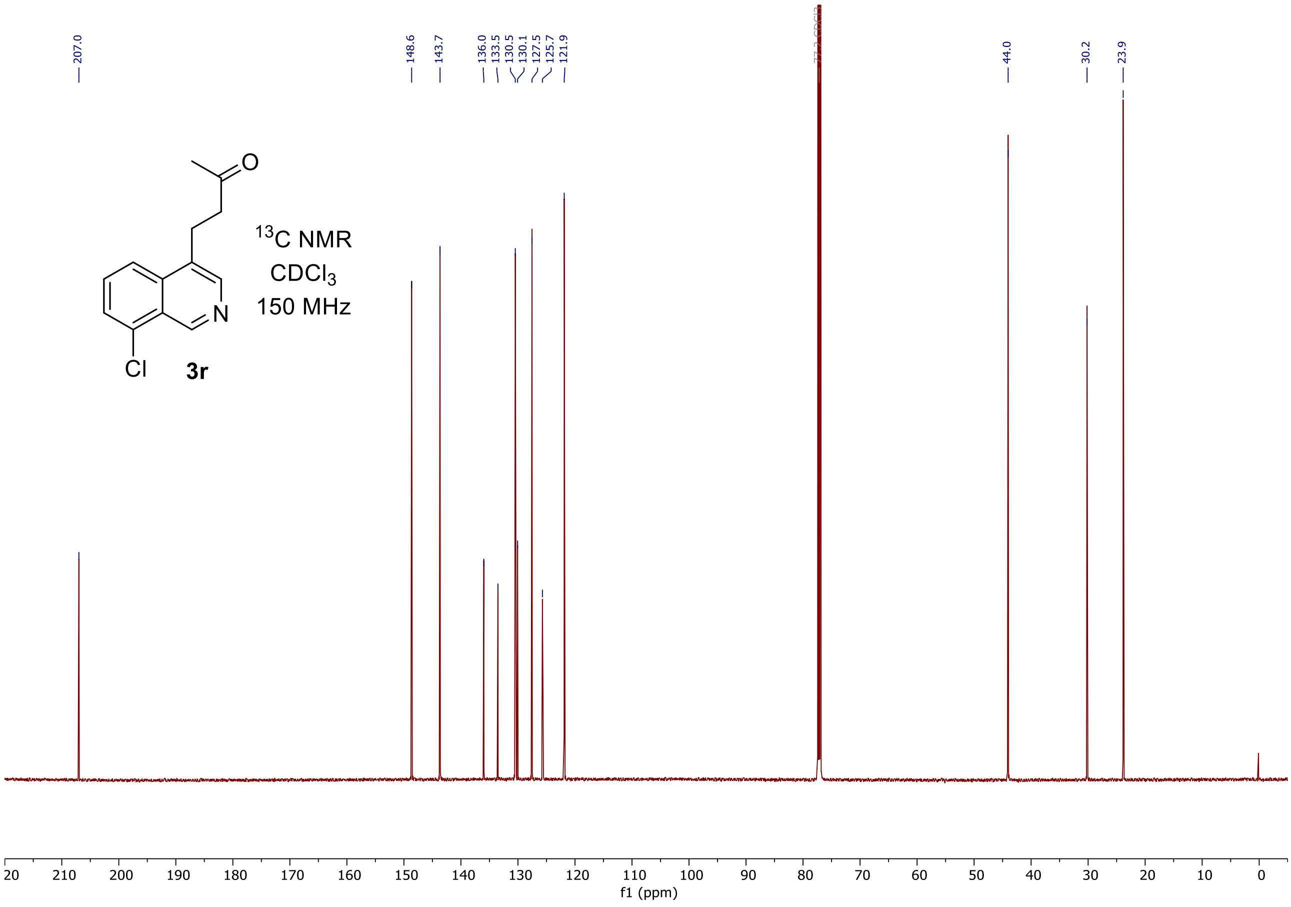
207.0
148.6
143.7
136.0
133.5
130.5
130.1
127.5
125.7
121.9
44.0
30.2
23.9
^{13}C NMR
$CDCl_3$
150 MHz
N
O
Cl
3r
f1 (ppm)

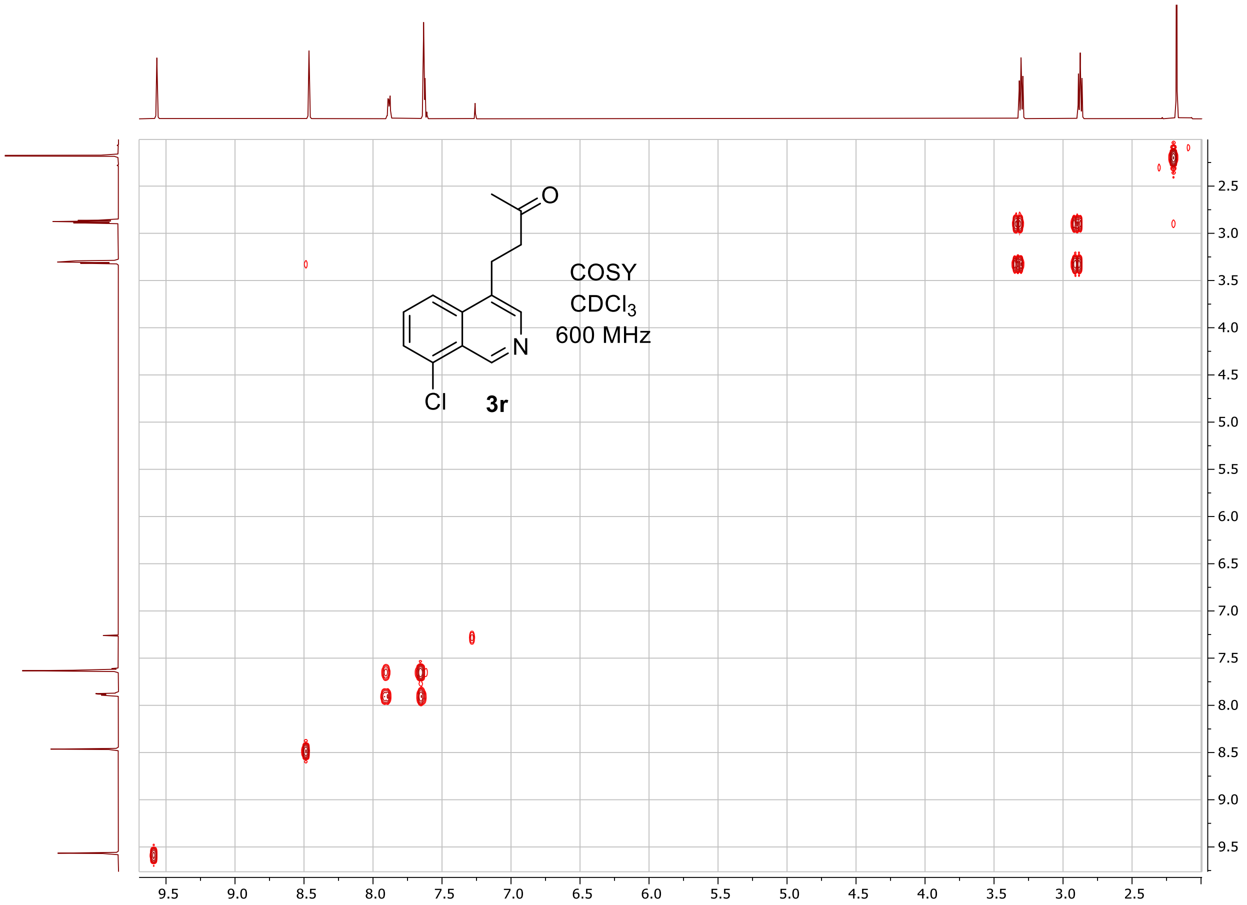
COSY
CDCl3
600 MHz
Cl
N
O
3r

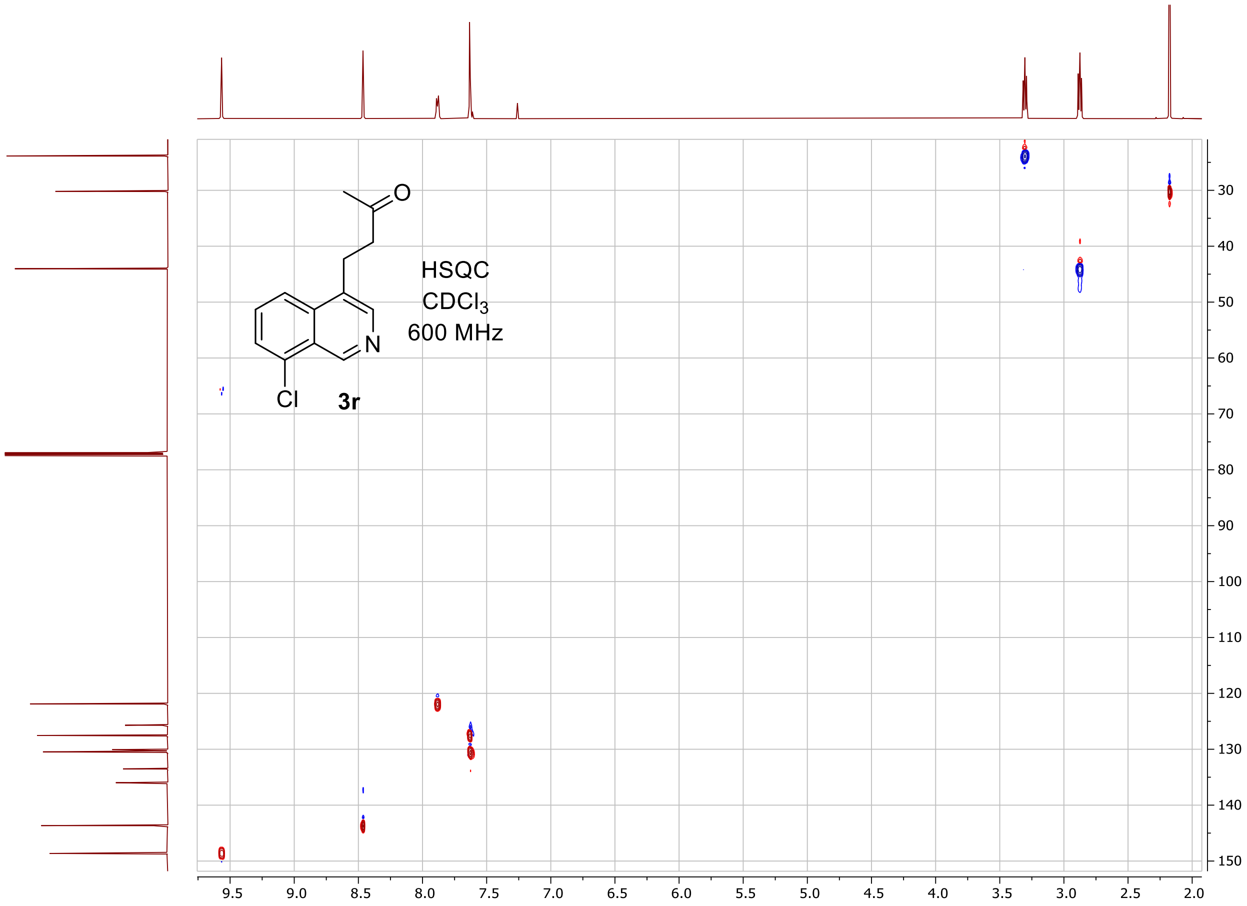
HSQC
CDCl3
600 MHz
Cl
N
O
3r

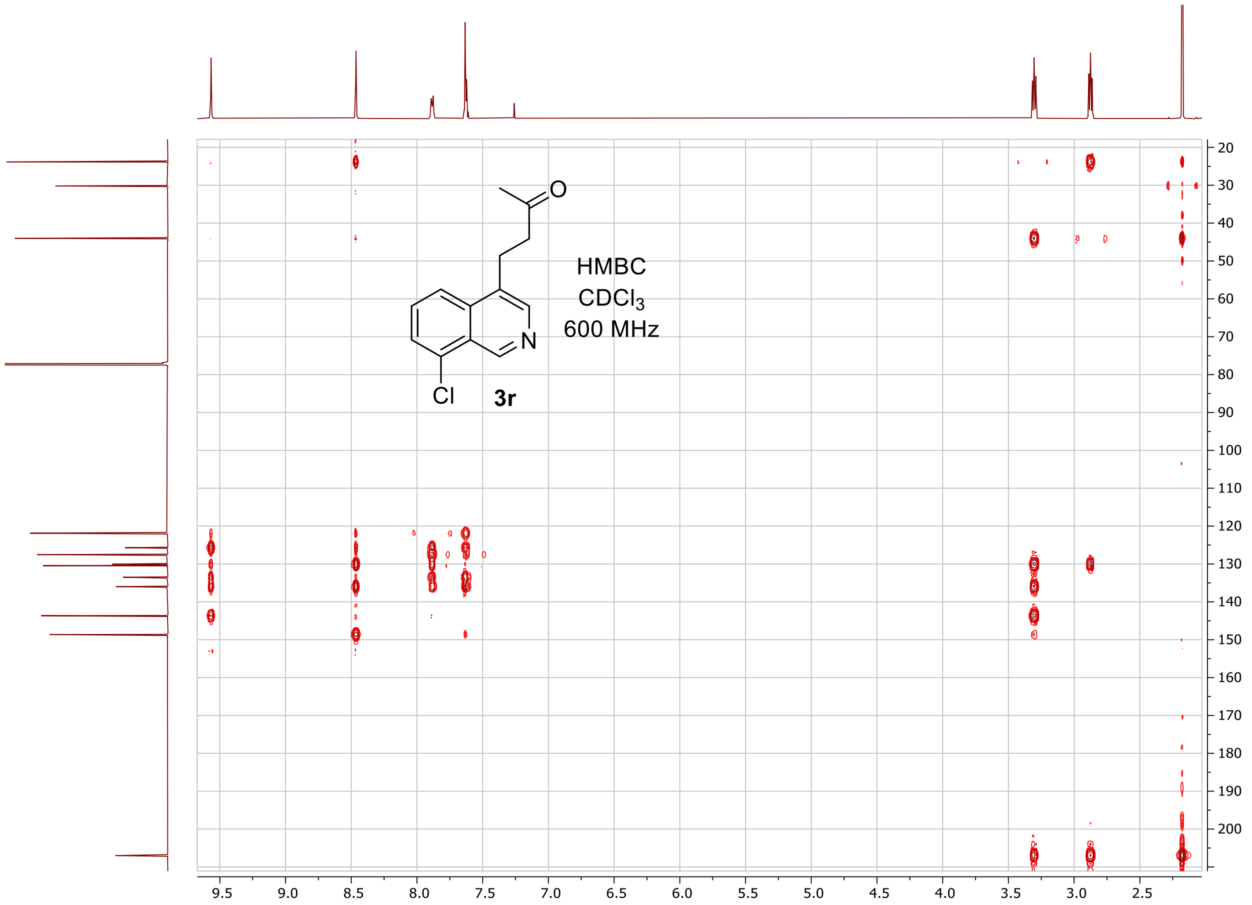

HMBC
CDCl3
600 MHz
3r

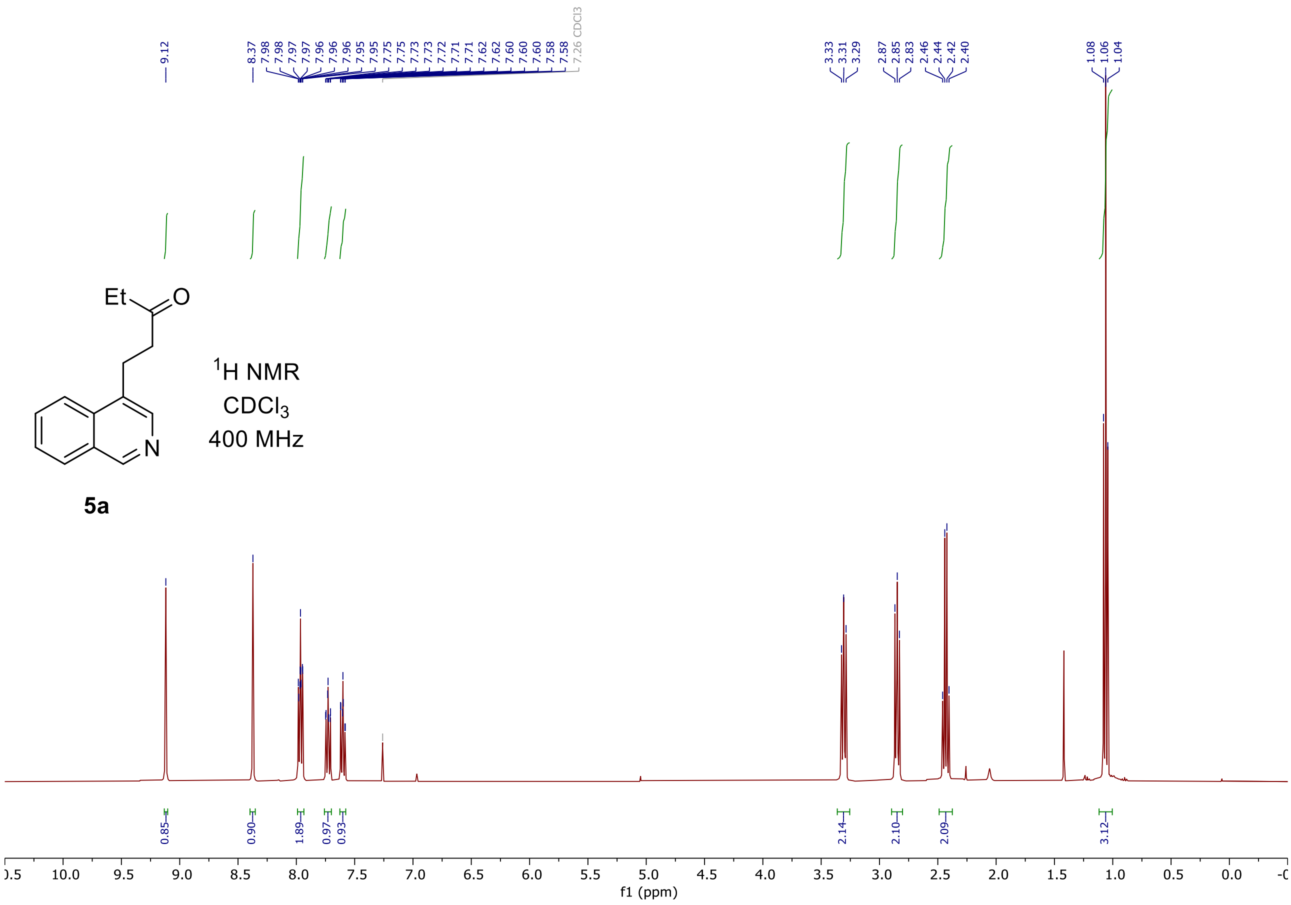
9.12
8.37
7.98
7.98
7.97
7.97
7.96
7.96
7.95
7.95
7.75
7.75
7.73
7.73
7.72
7.71
7.71
7.62
7.62
7.60
7.60
7.58
7.58
7.26 CDCl3
3.33
3.31
3.29
2.87
2.85
2.83
2.46
2.44
2.42
2.40
1.08
1.06
1.04
Et
O
N
1H NMR
CDCl3
400 MHz
5a
0.85
0.90
1.89
0.97
0.93
2.14
2.10
2.09
3.12
f1 (ppm)

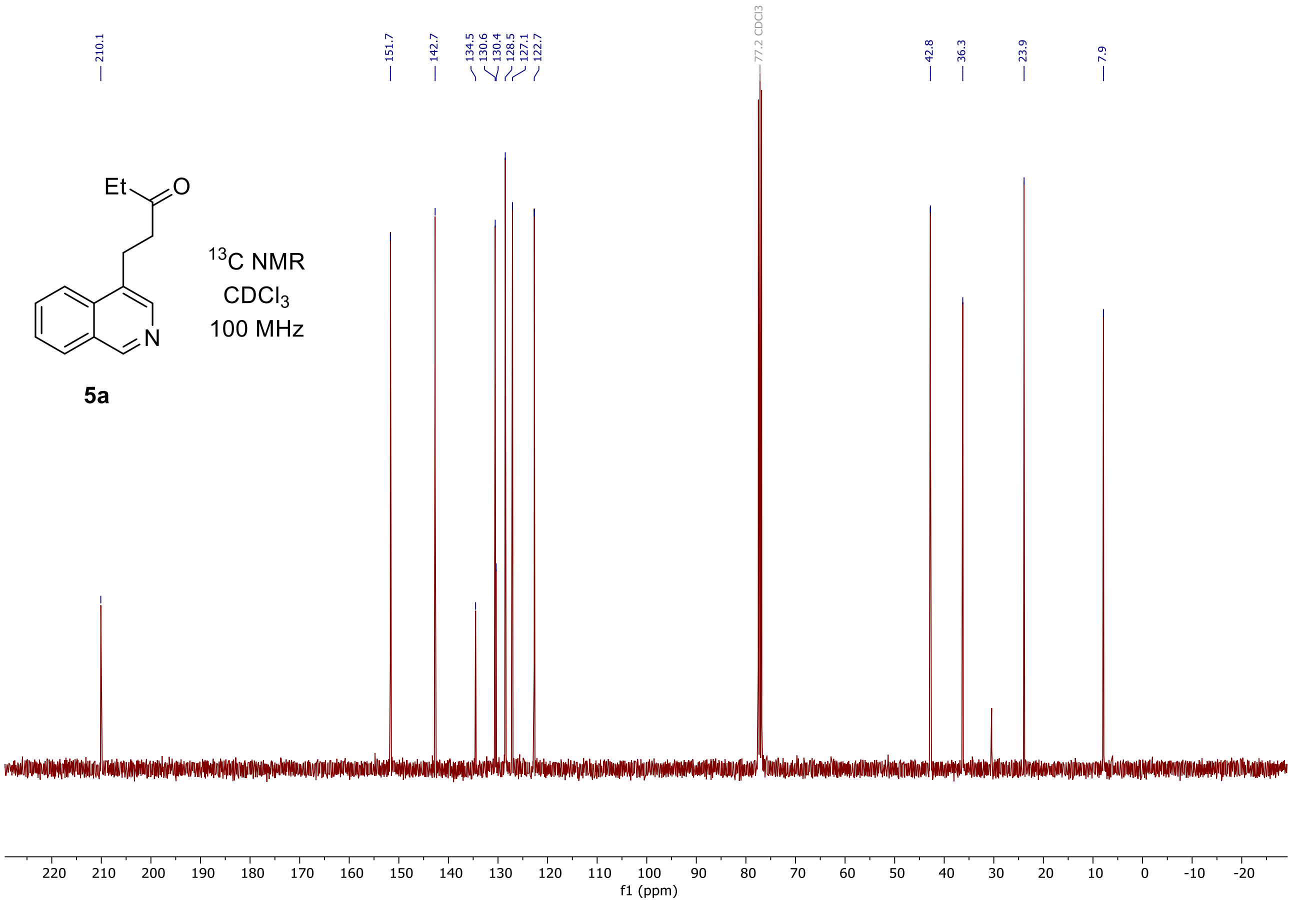
210.1
151.7
142.7
134.5
130.6
130.4
128.5
127.1
122.7
77.2 CDCl3
42.8
36.3
23.9
7.9
Et
O
N
13C NMR
CDCl3
100 MHz
5a
f1 (ppm)

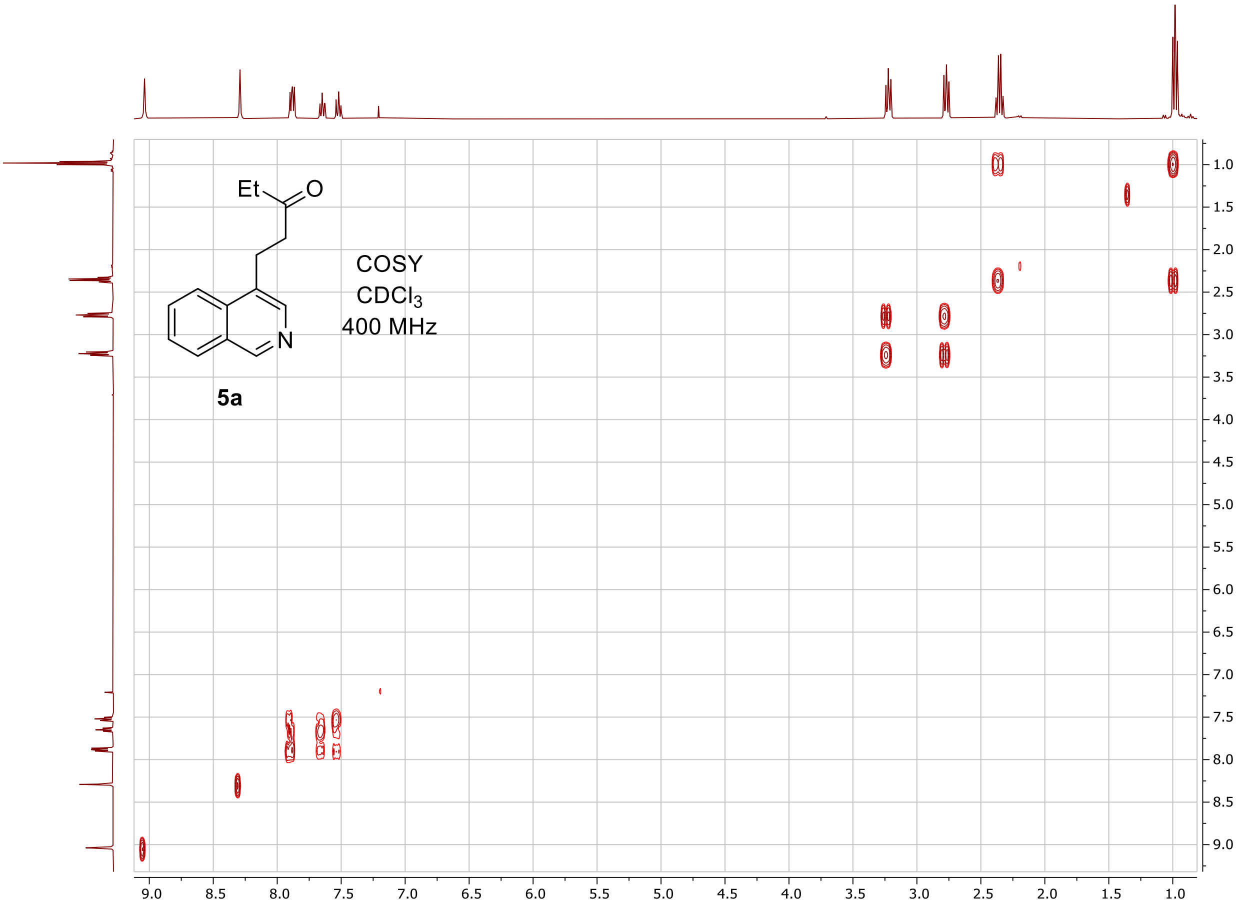

Et
O
N
5a
COSY
CDCl3
400 MHz

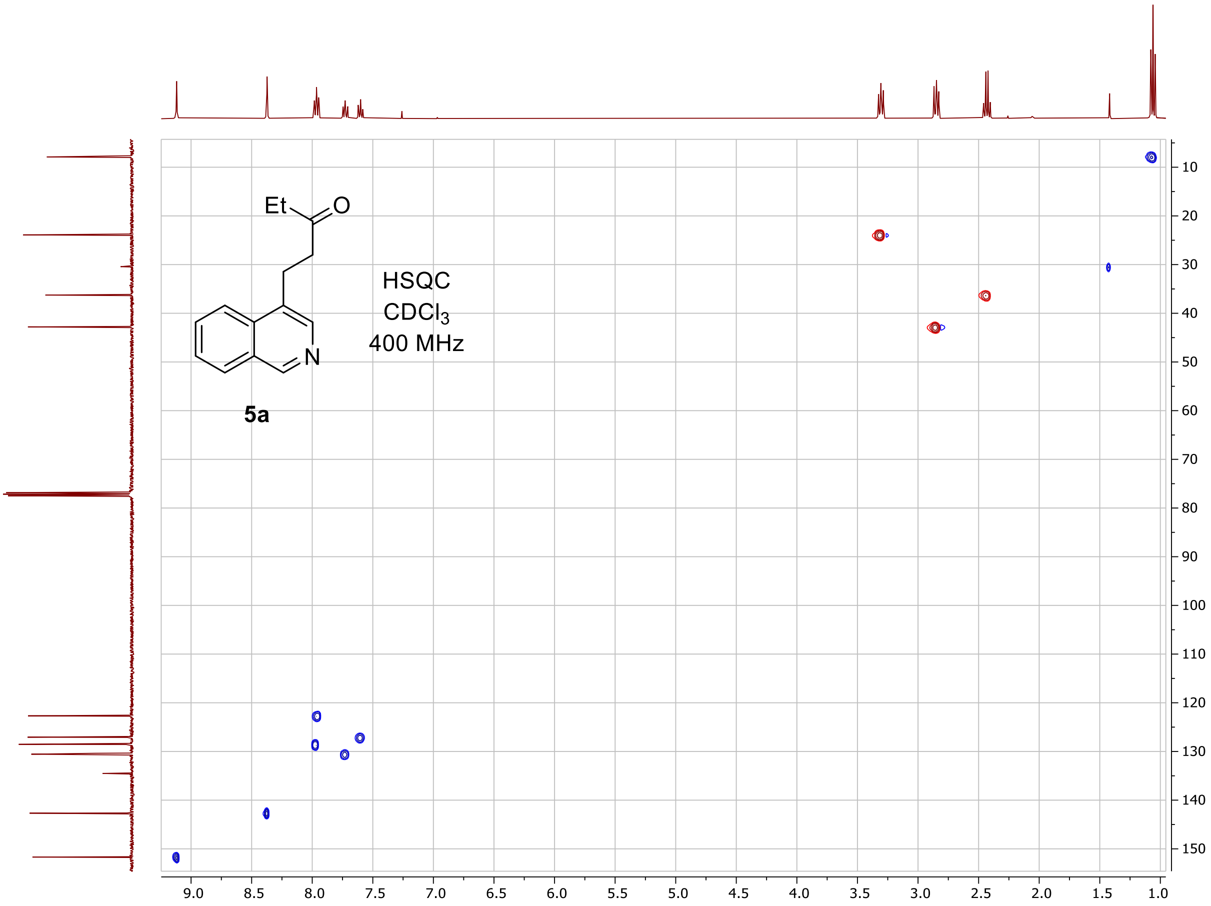

Et
O
N
5a
HSQC
CDCl3
400 MHz

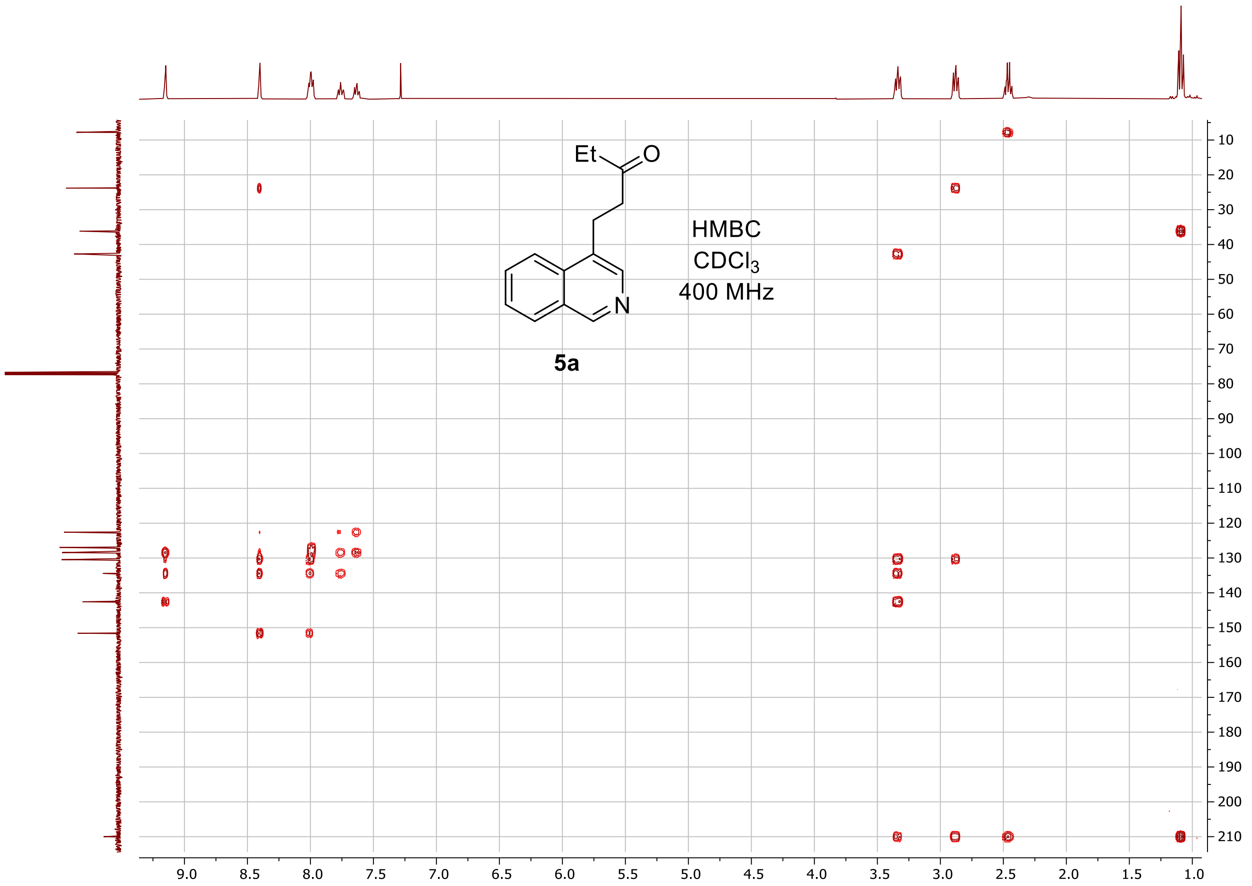

Et
O
N
5a
HMBC
CDCl3
400 MHz
10
20
30
40
50
60
70
80
90
100
110
120
130
140
150
160
170
180
190
200
210
9.0
8.5
8.0
7.5
7.0
6.5
6.0
5.5
5.0
4.5
4.0
3.5
3.0
2.5
2.0
1.5
1.0

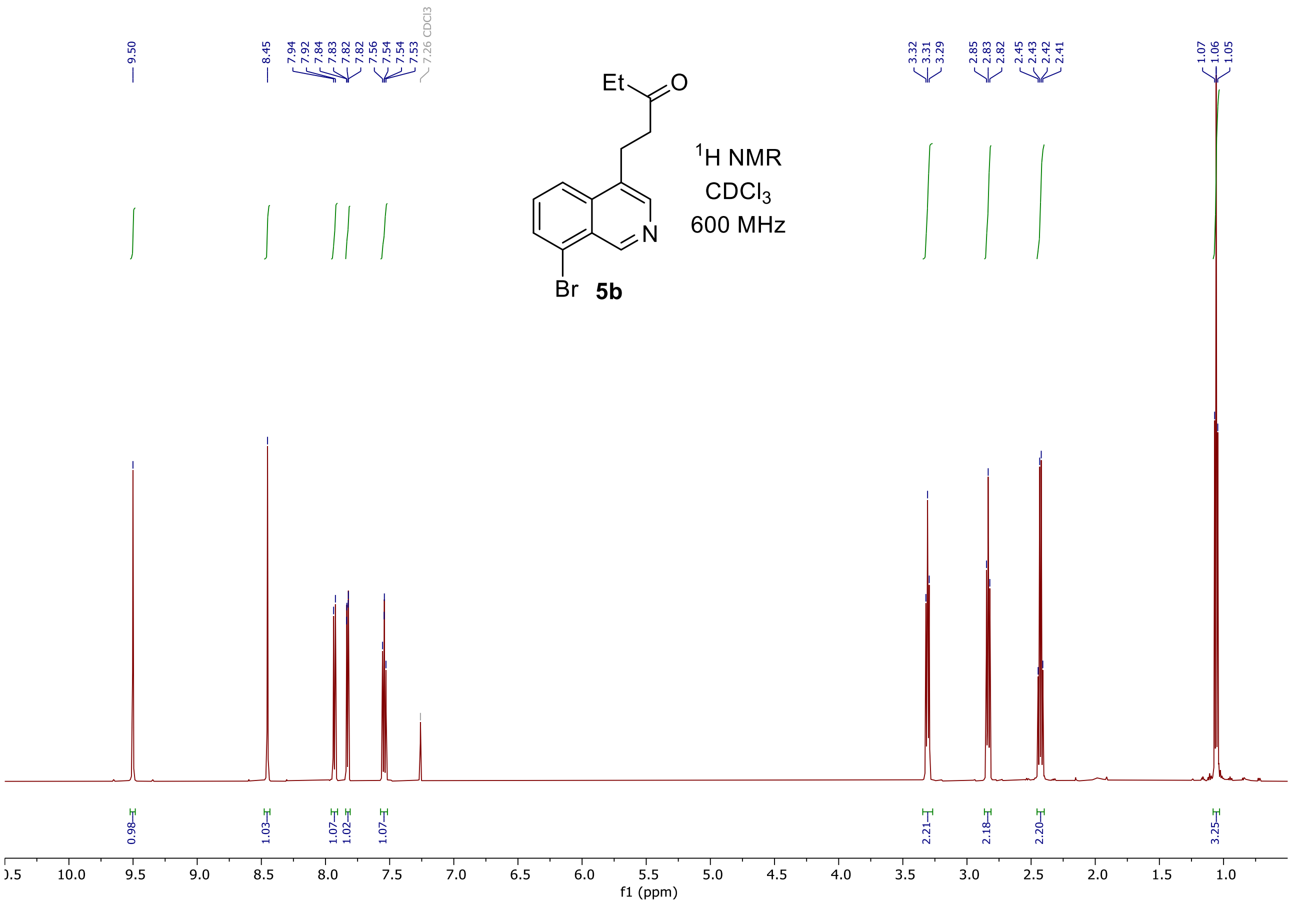
Et
O
N
Br
5b
1H NMR
CDCl3
600 MHz
f1 (ppm)

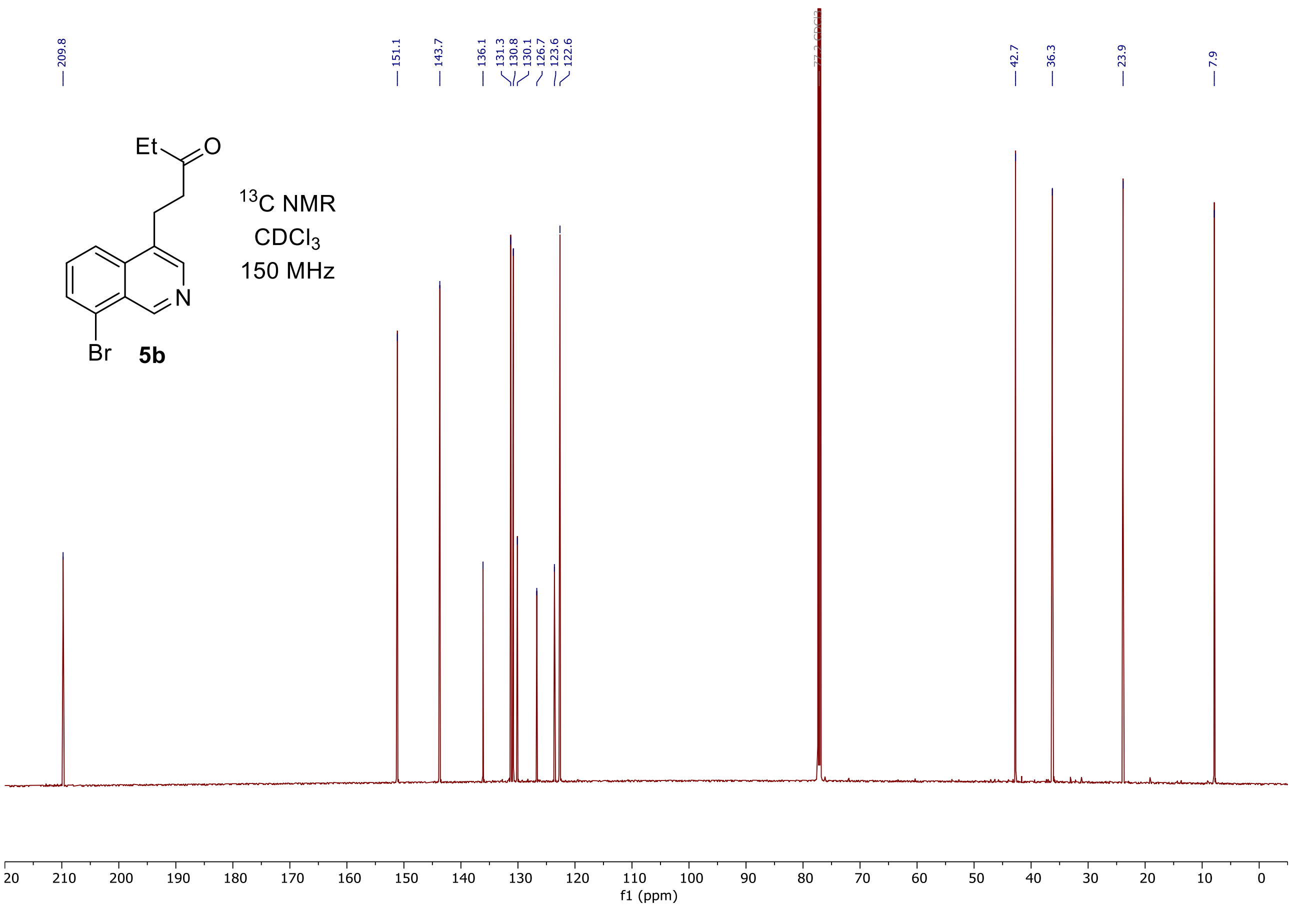
Et
O
N
Br
5b
13C NMR
CDCl3
150 MHz
f1 (ppm)

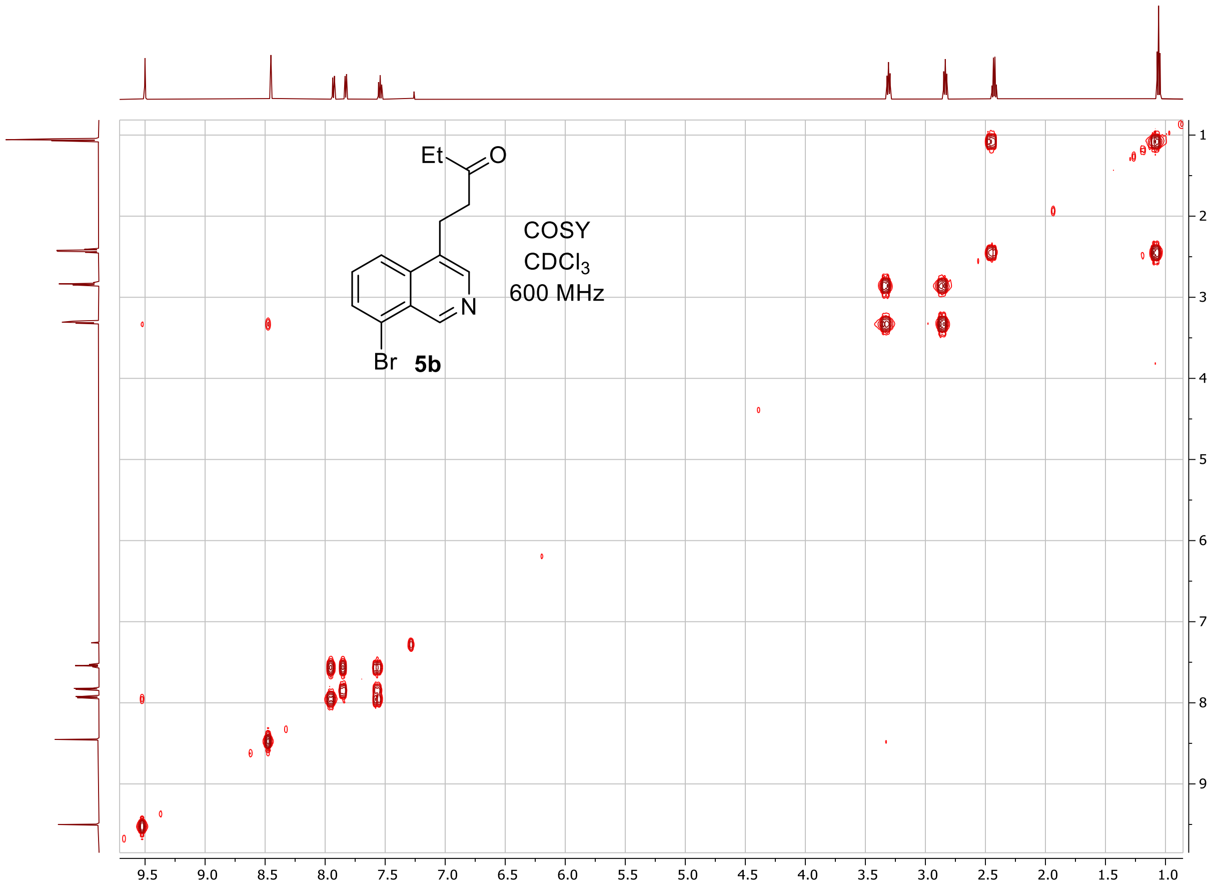
Et
O
N
Br
5b
COSY
CDCl3
600 MHz

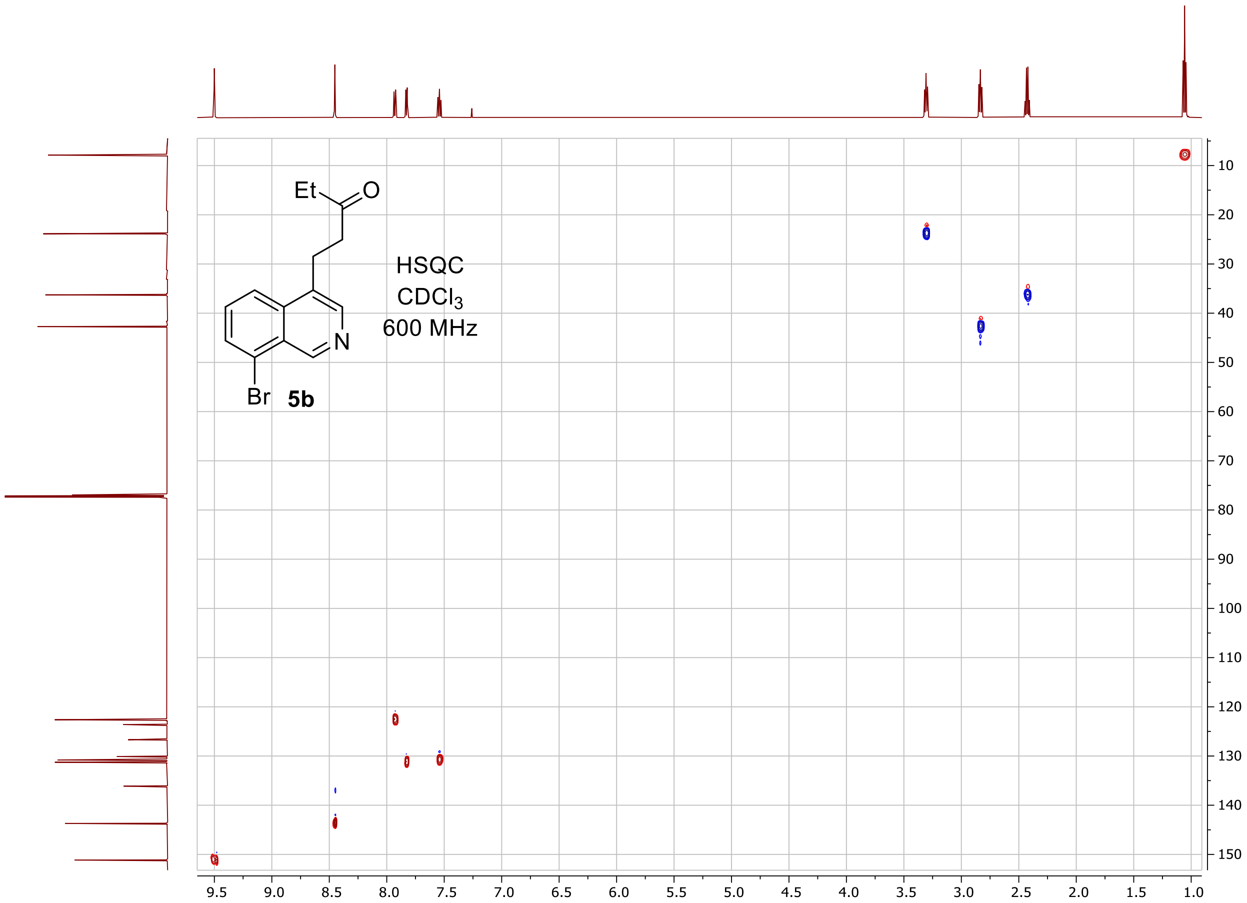
Et
O
N
Br
5b
HSQC
CDCl3
600 MHz

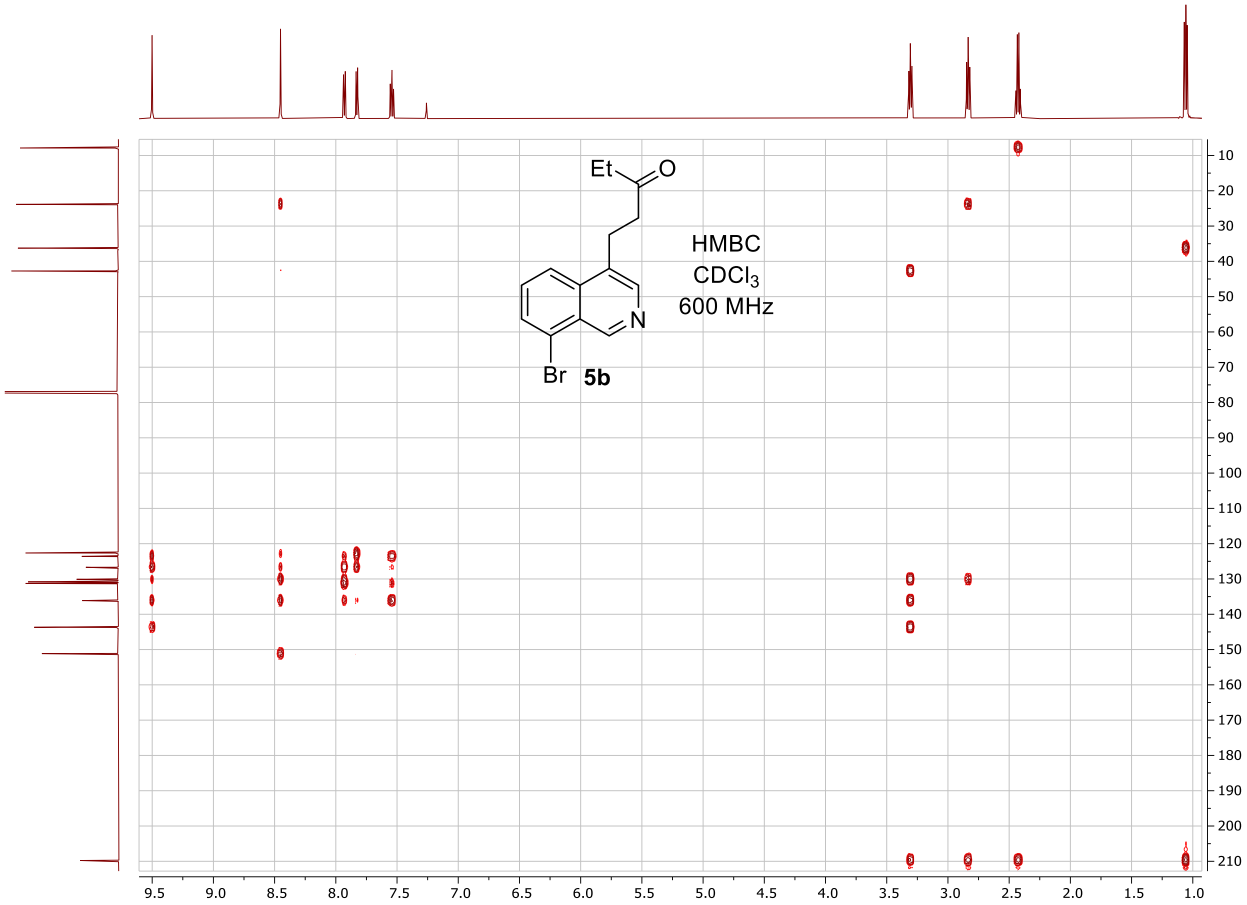
Et
O
N
Br
5b
HMBC
$CDCl_3$
600 MHz
10
20
30
40
50
60
70
80
90
100
110
120
130
140
150
160
170
180
190
200
210
9.5
9.0
8.5
8.0
7.5
7.0
6.5
6.0
5.5
5.0
4.5
4.0
3.5
3.0
2.5
2.0
1.5
1.0

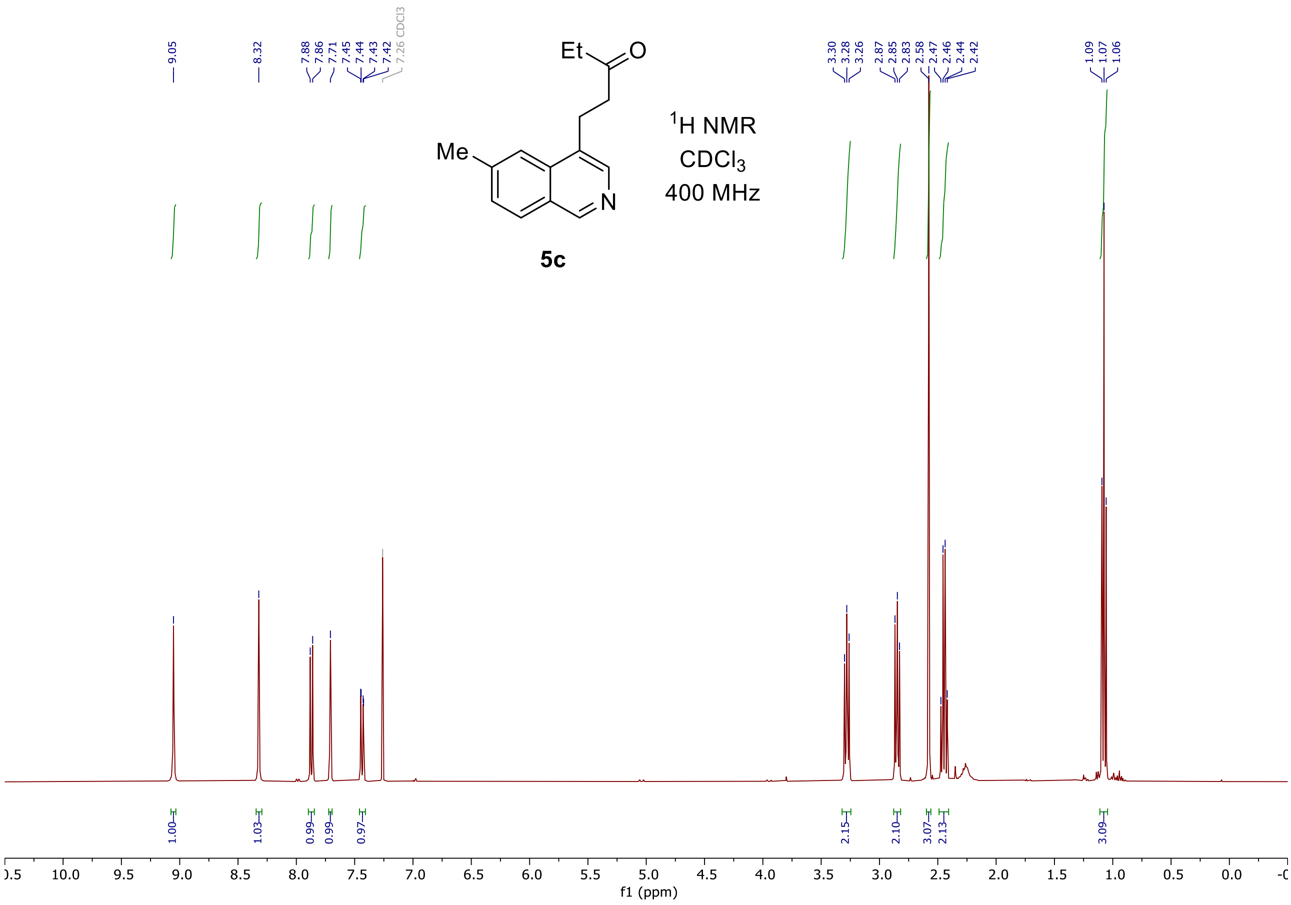
9.05
8.32
7.88
7.86
7.71
7.45
7.44
7.43
7.42
7.26 CDCl3
3.30
3.28
3.26
2.87
2.85
2.83
2.58
2.47
2.46
2.44
2.42
1.09
1.07
1.06
Et
O
Me
N
5c
1H NMR
CDCl3
400 MHz
1.00
1.03
0.99
0.99
0.97
2.15
2.10
3.07
2.13
3.09
f1 (ppm)

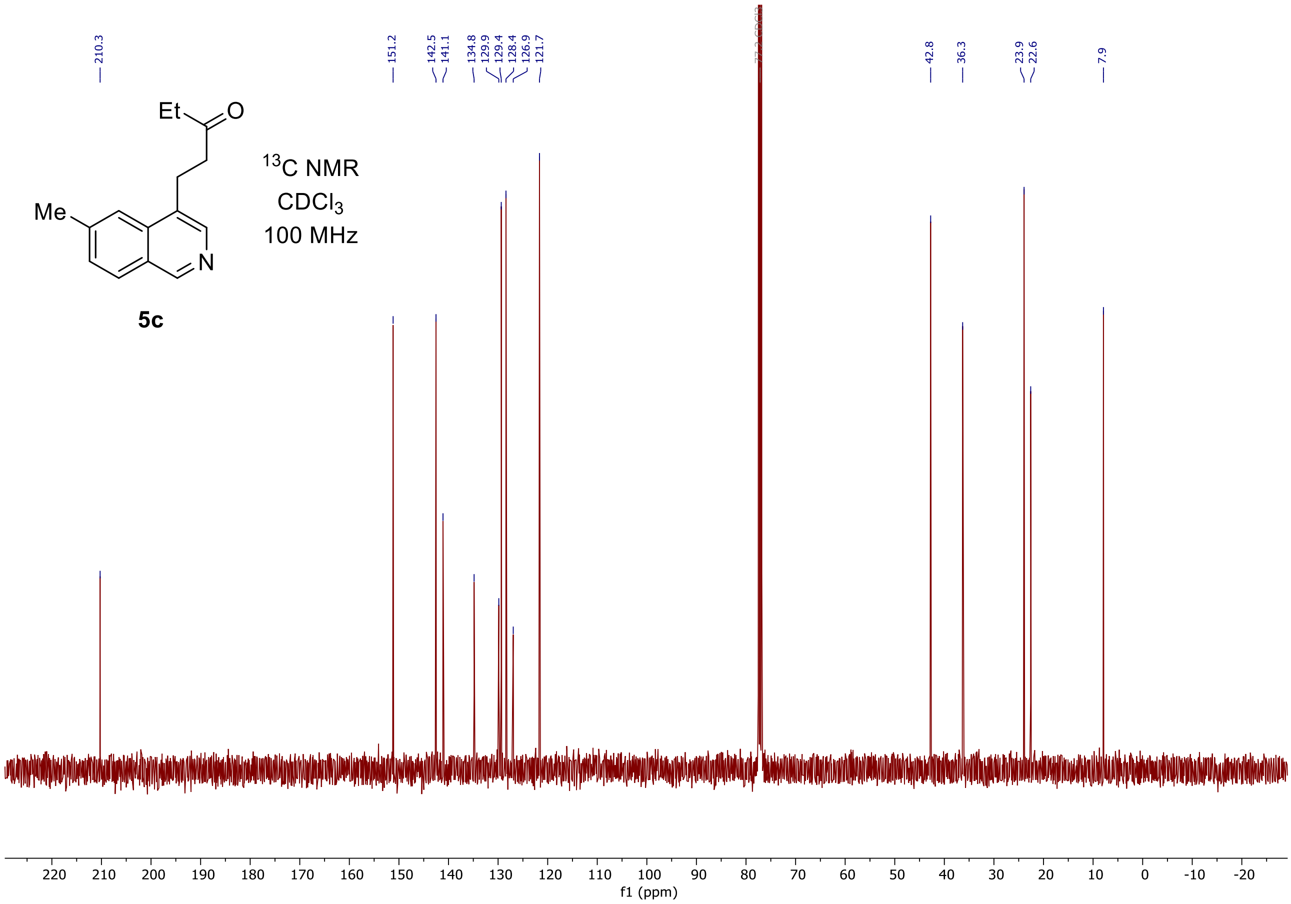
210.3
151.2
142.5
141.1
134.8
129.9
129.4
128.4
126.9
121.7
42.8
36.3
23.9
22.6
7.9
Et
O
Me
N
5c
13C NMR
CDCl3
100 MHz
f1 (ppm)

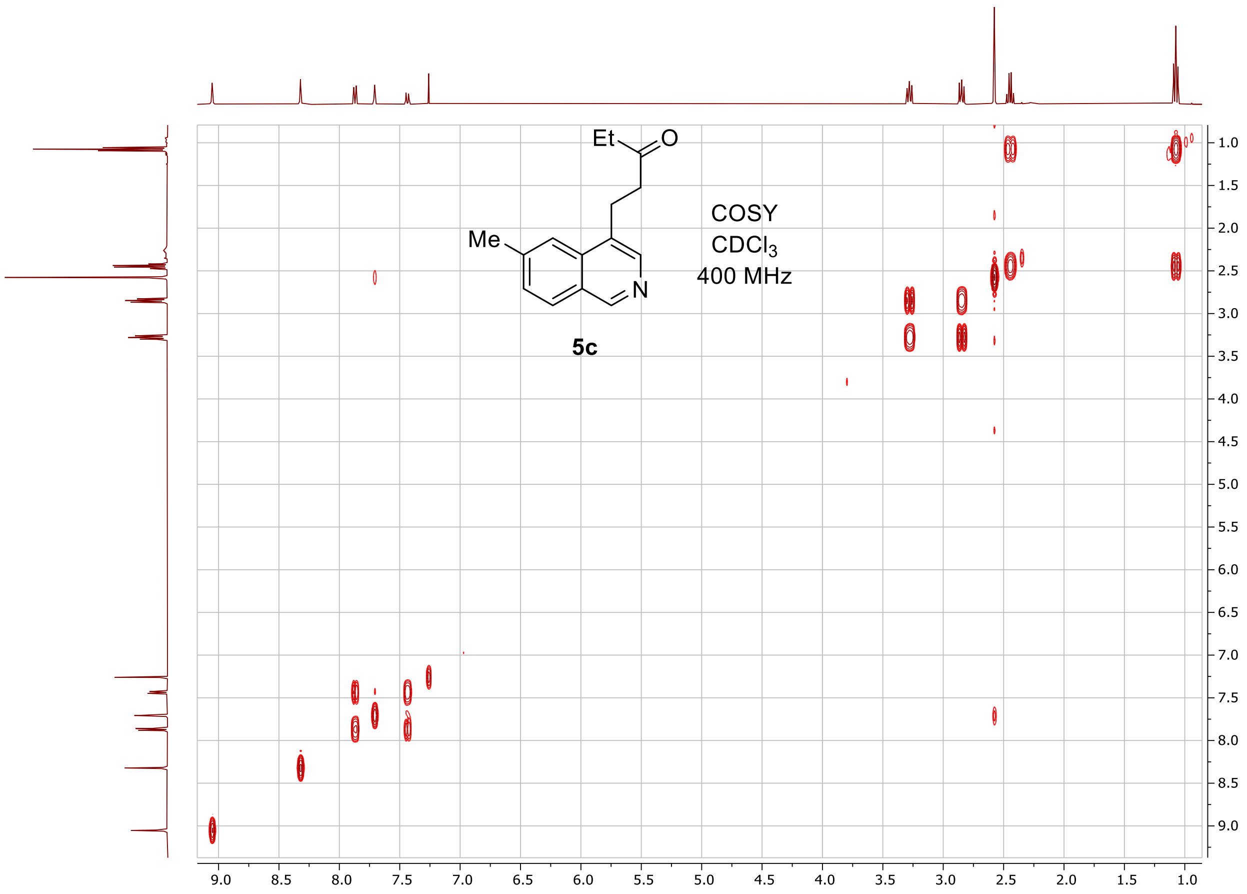
Et
O
Me
N
5c
COSY
CDCl3
400 MHz

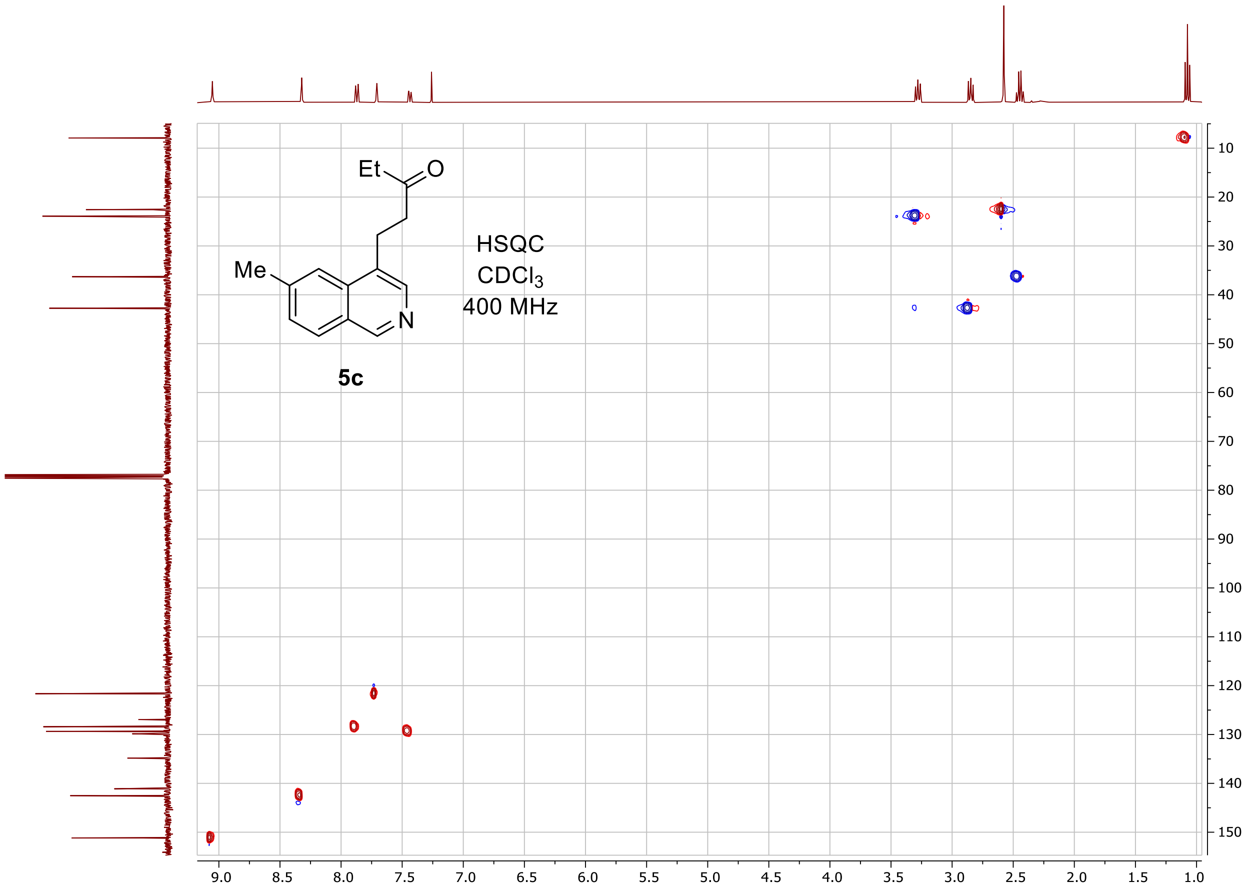
Et
O
Me
N
5c
HSQC
CDCl3
400 MHz

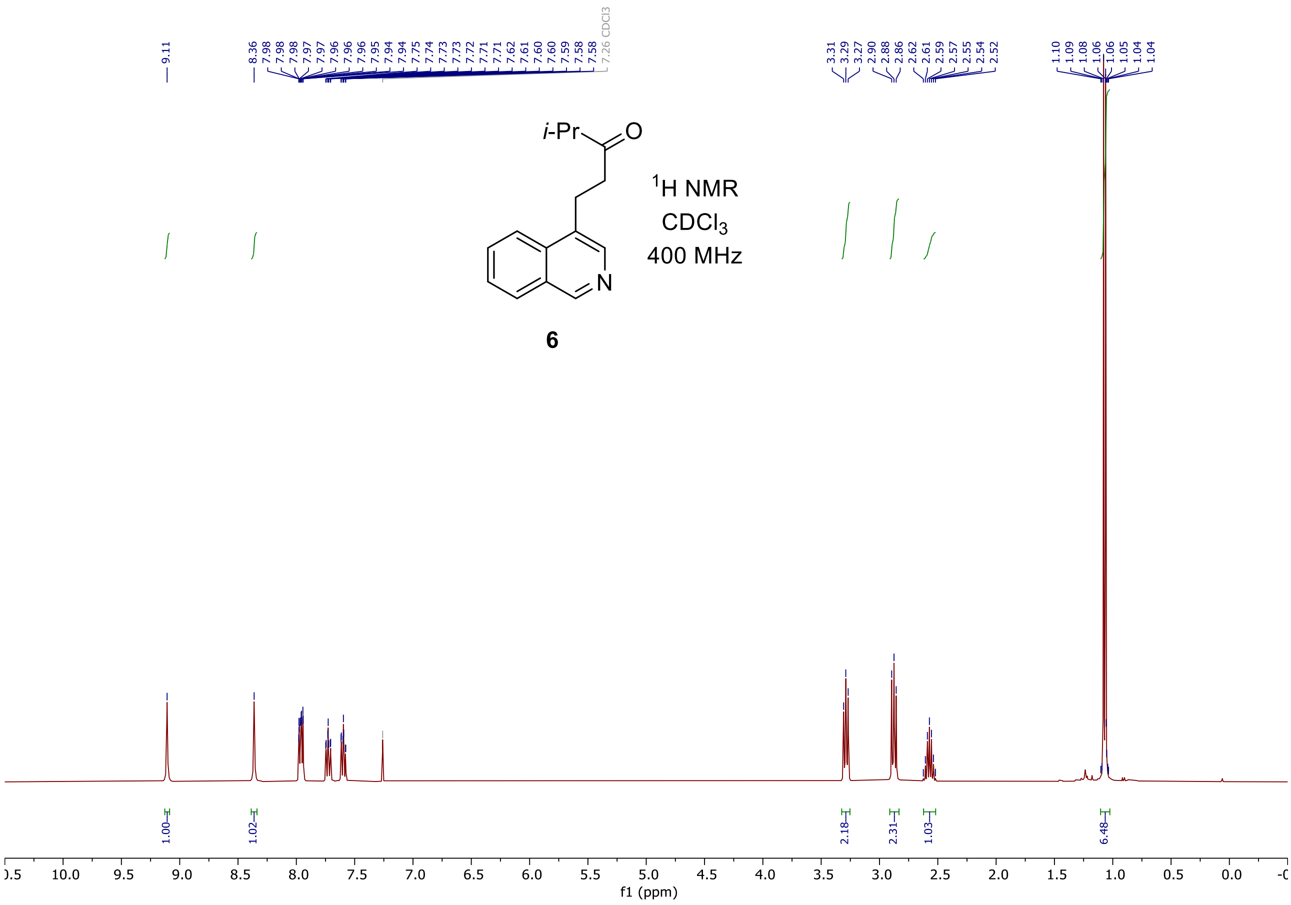

i-Pr
O
N
6
^{1}H NMR
$CDCl_3$
400 MHz
f1 (ppm)

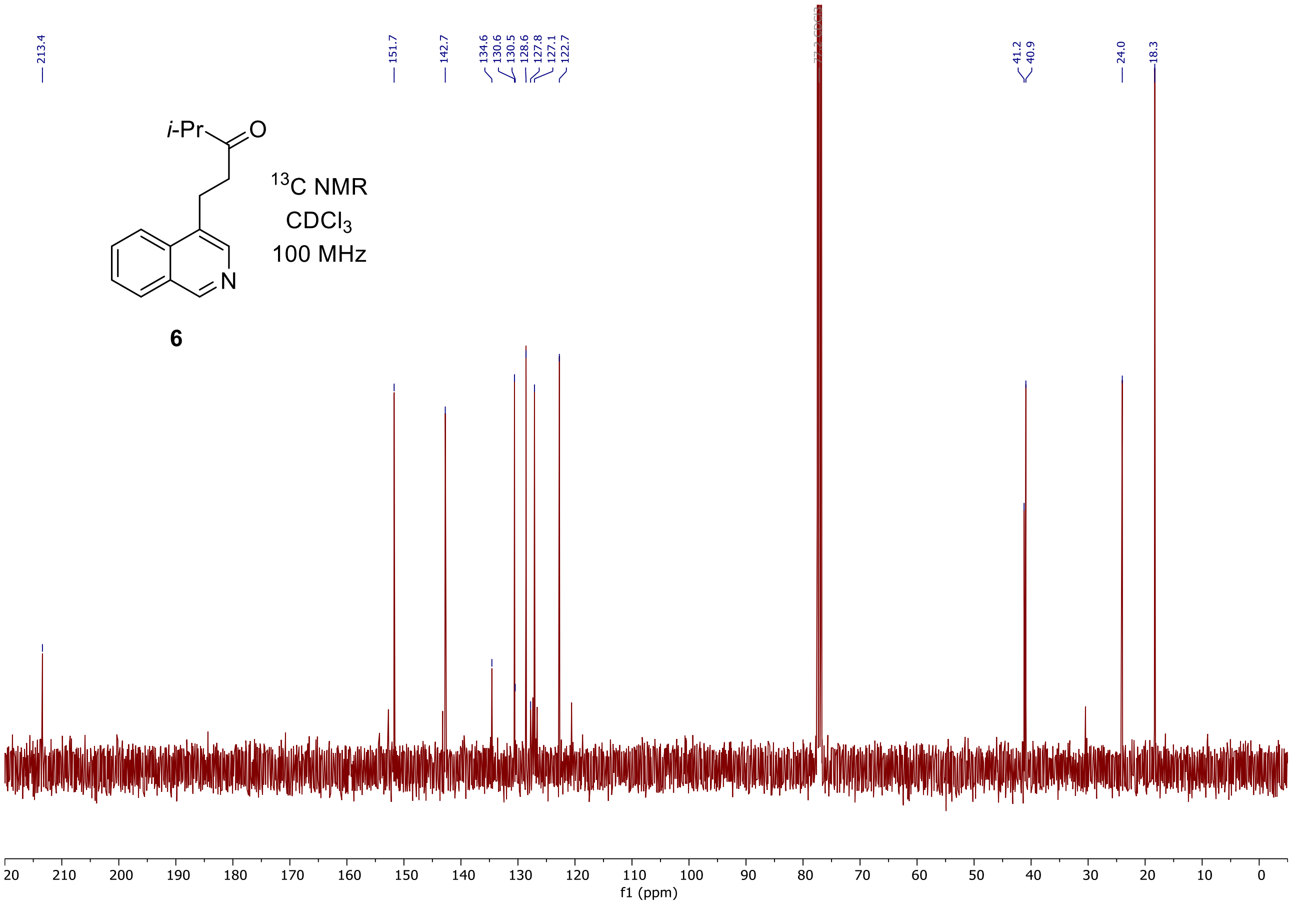

i-Pr
O
N
6
^{13}C NMR
$CDCl_3$
100 MHz
f1 (ppm)

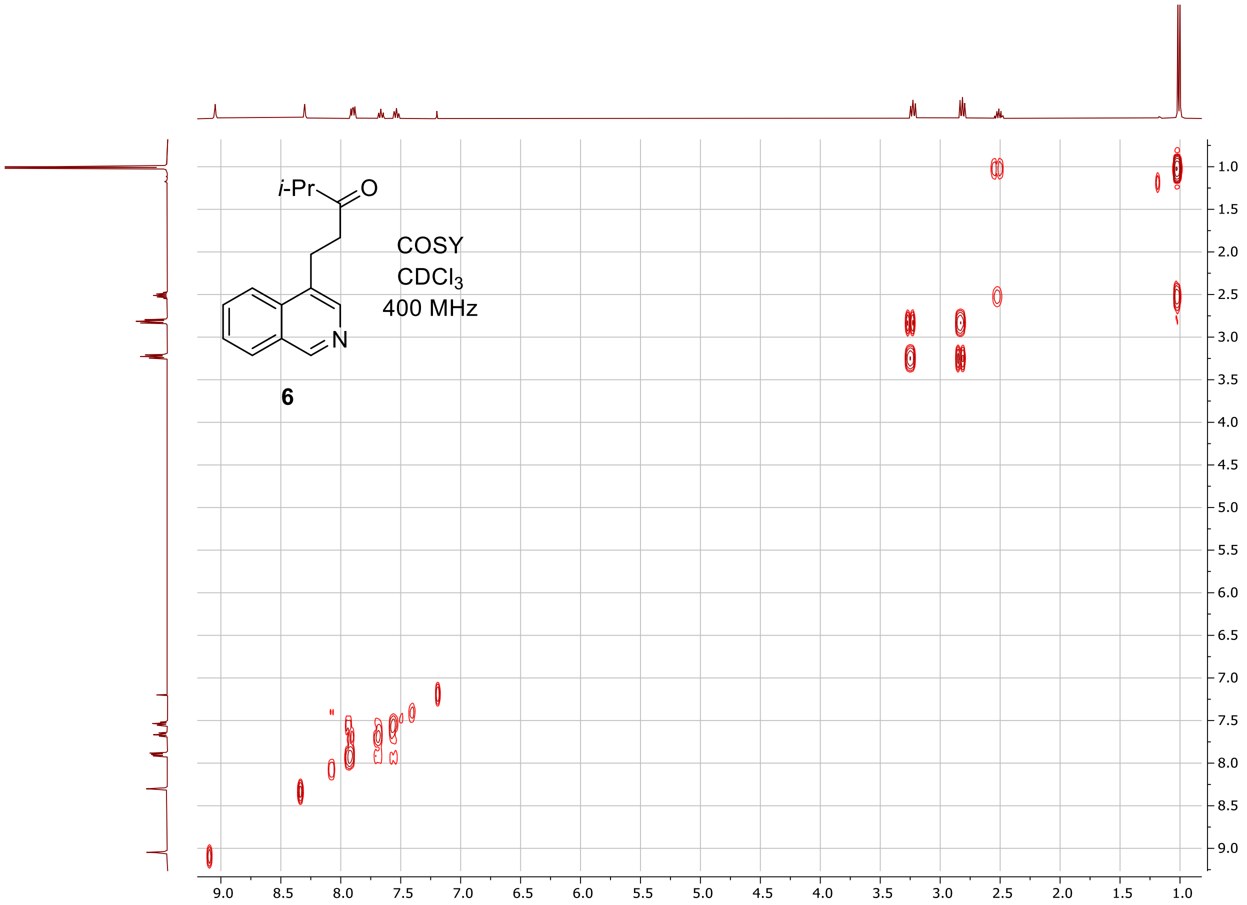
i-Pr
O
N
6
COSY
CDCl3
400 MHz

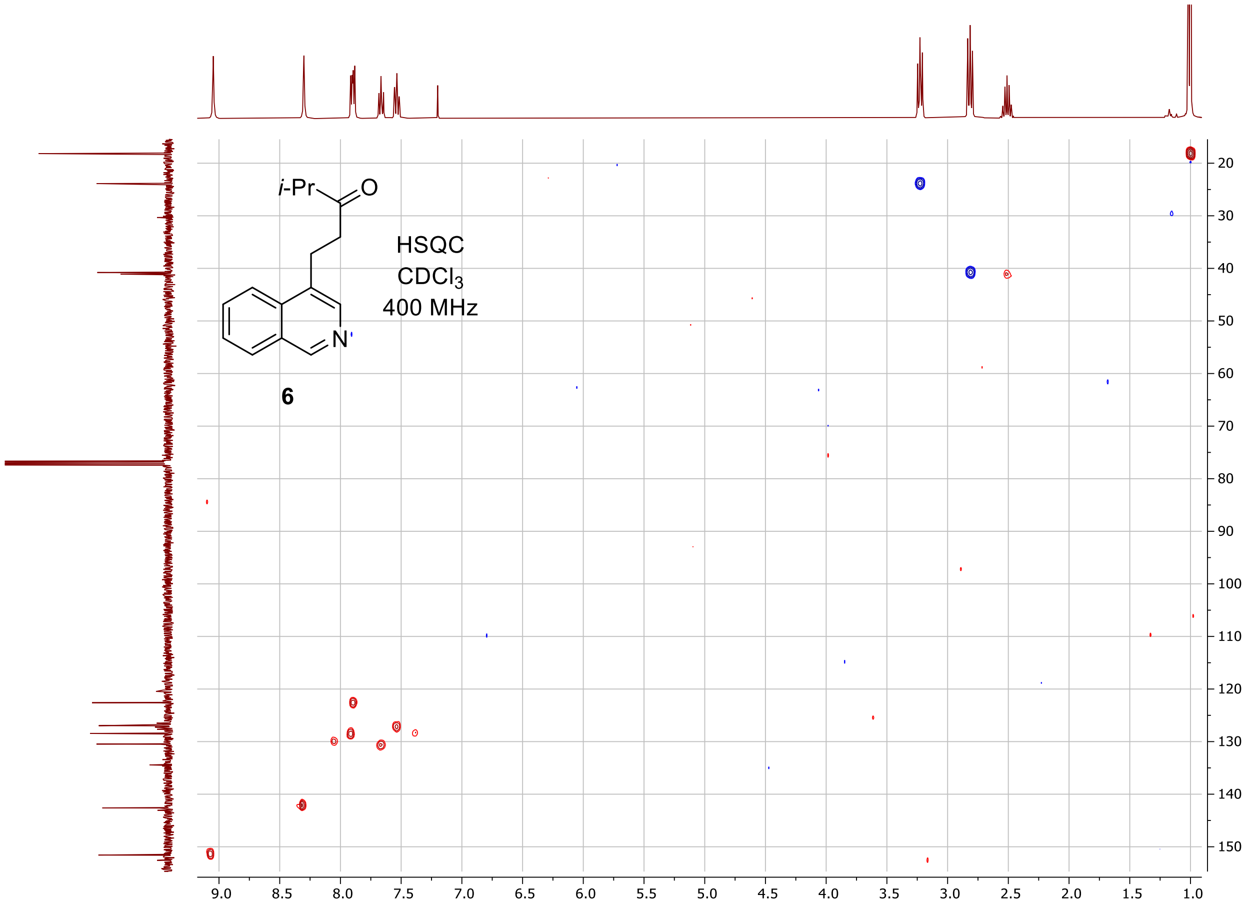
i-Pr
O
N
6
HSQC
CDCl3
400 MHz

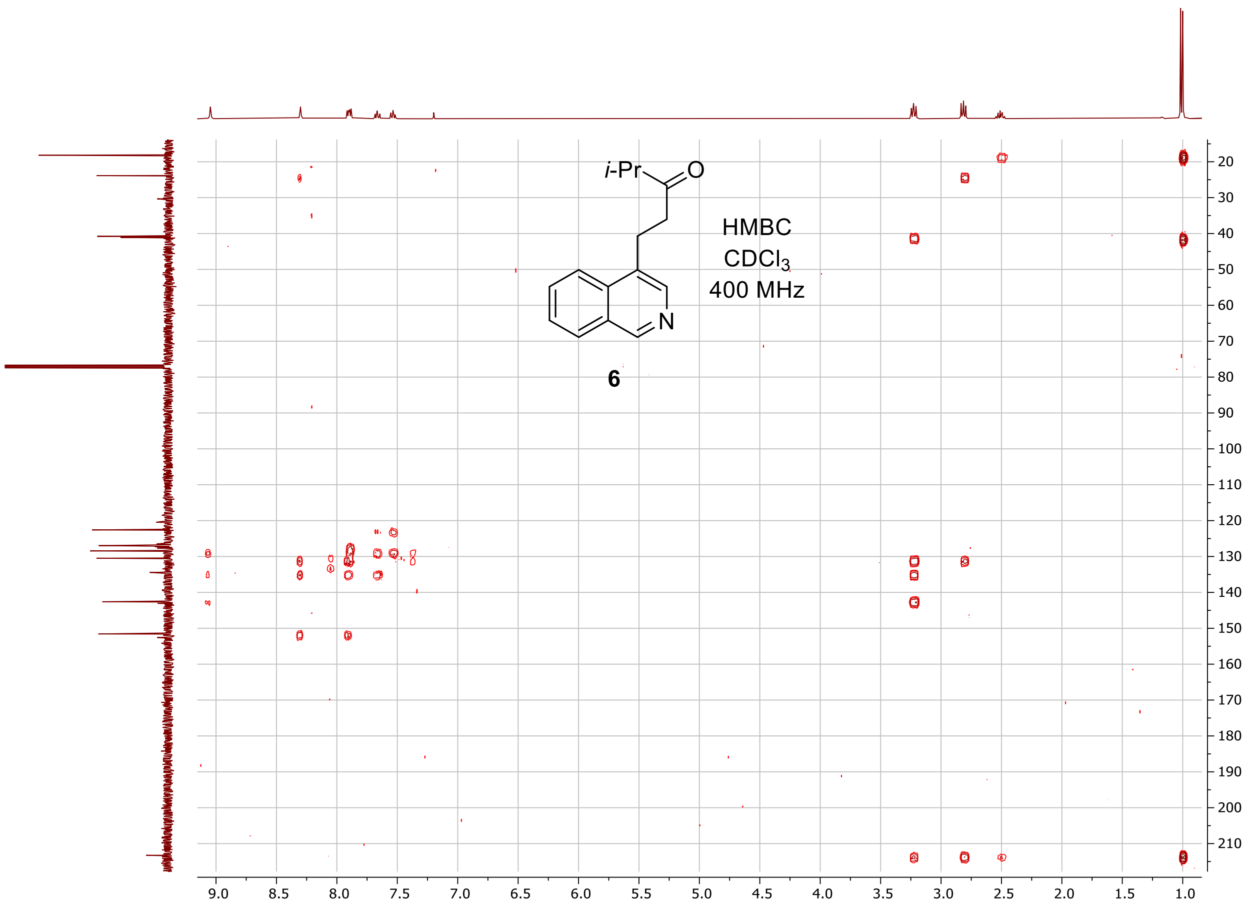
i-Pr
O
N
6
HMBC
CDCl3
400 MHz

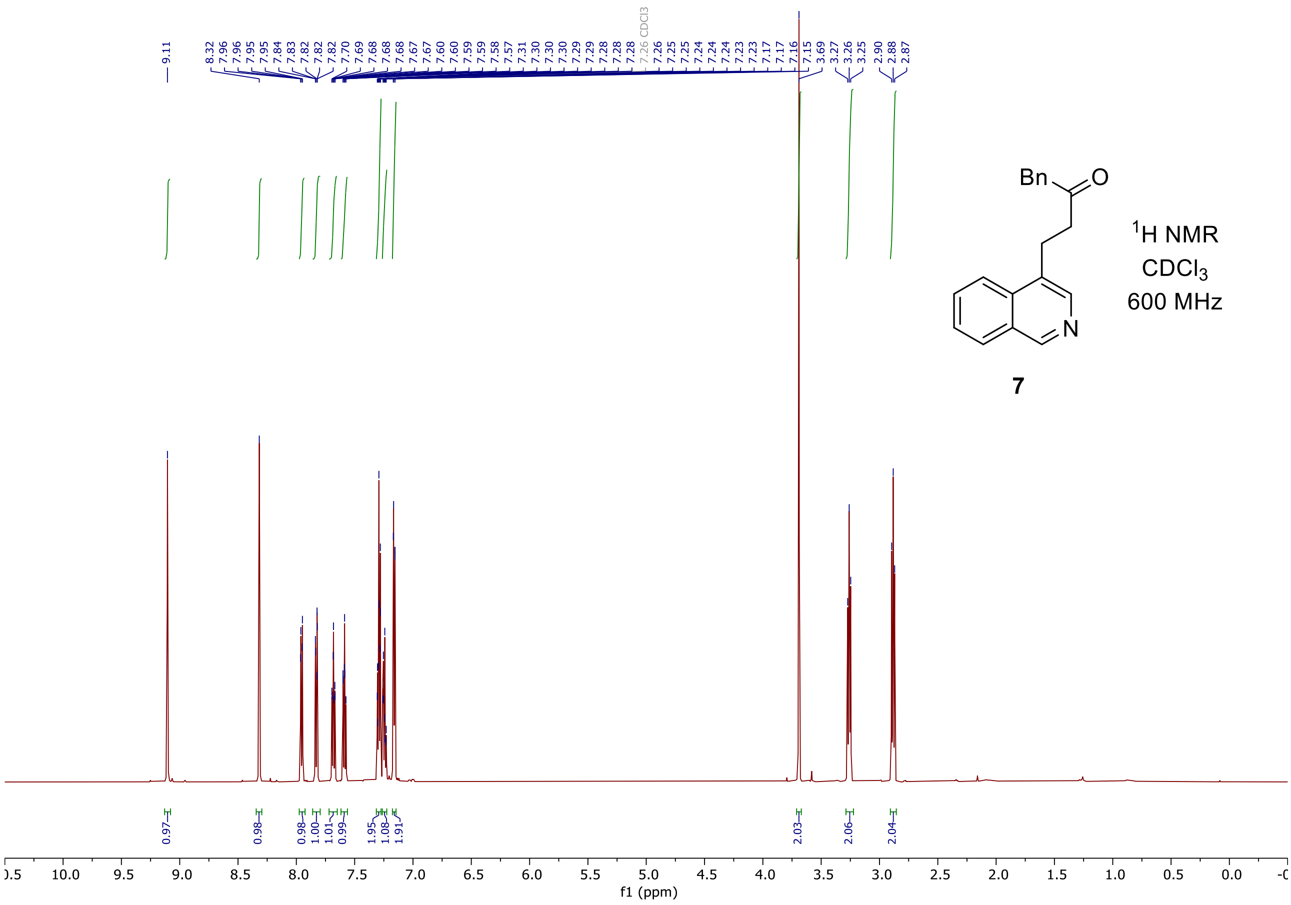

Bn
O
N
7
^{1}H NMR
$CDCl_3$
600 MHz
f1 (ppm)

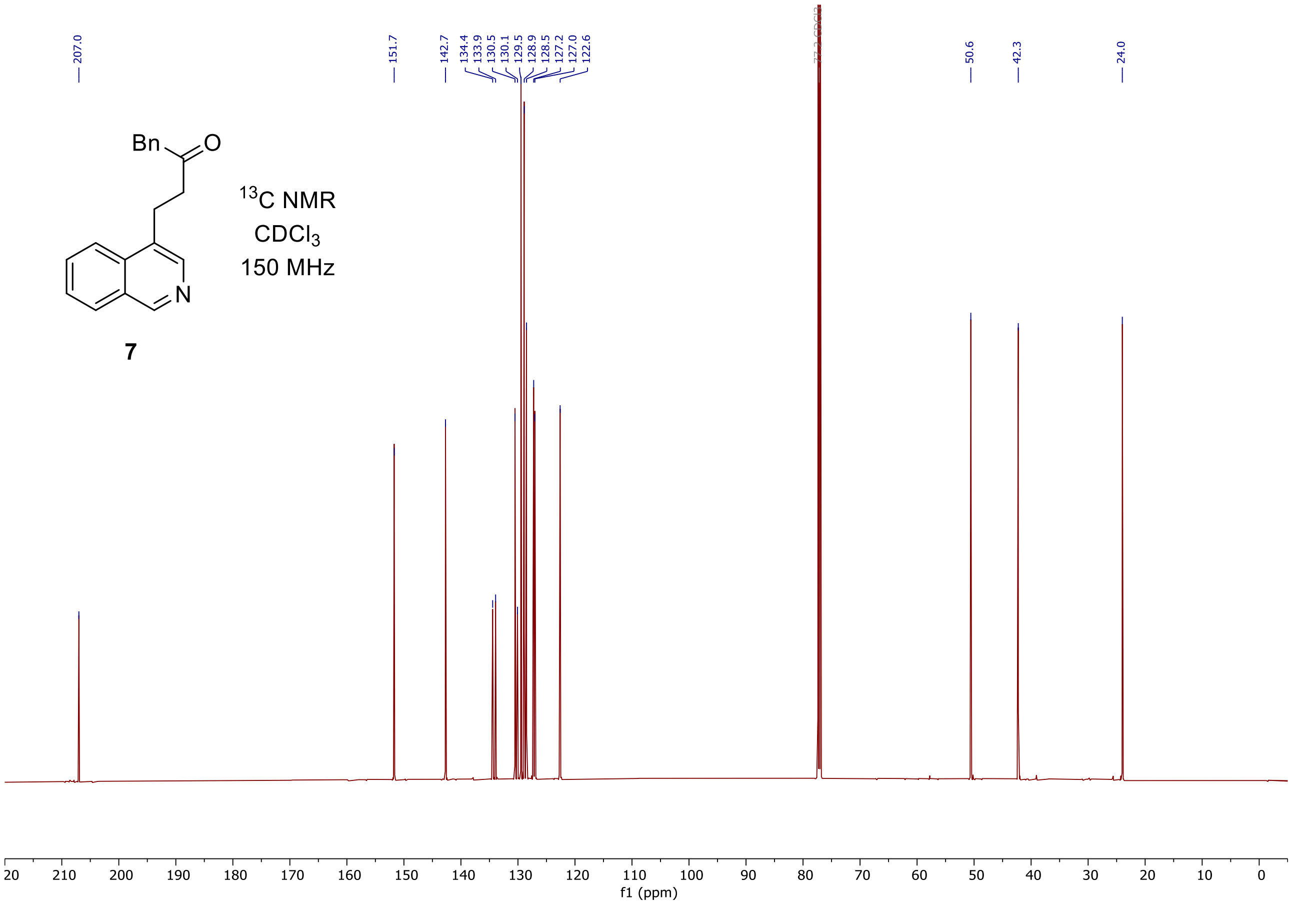

Bn
O
N
7
^{13}C NMR
$CDCl_3$
150 MHz
f1 (ppm)

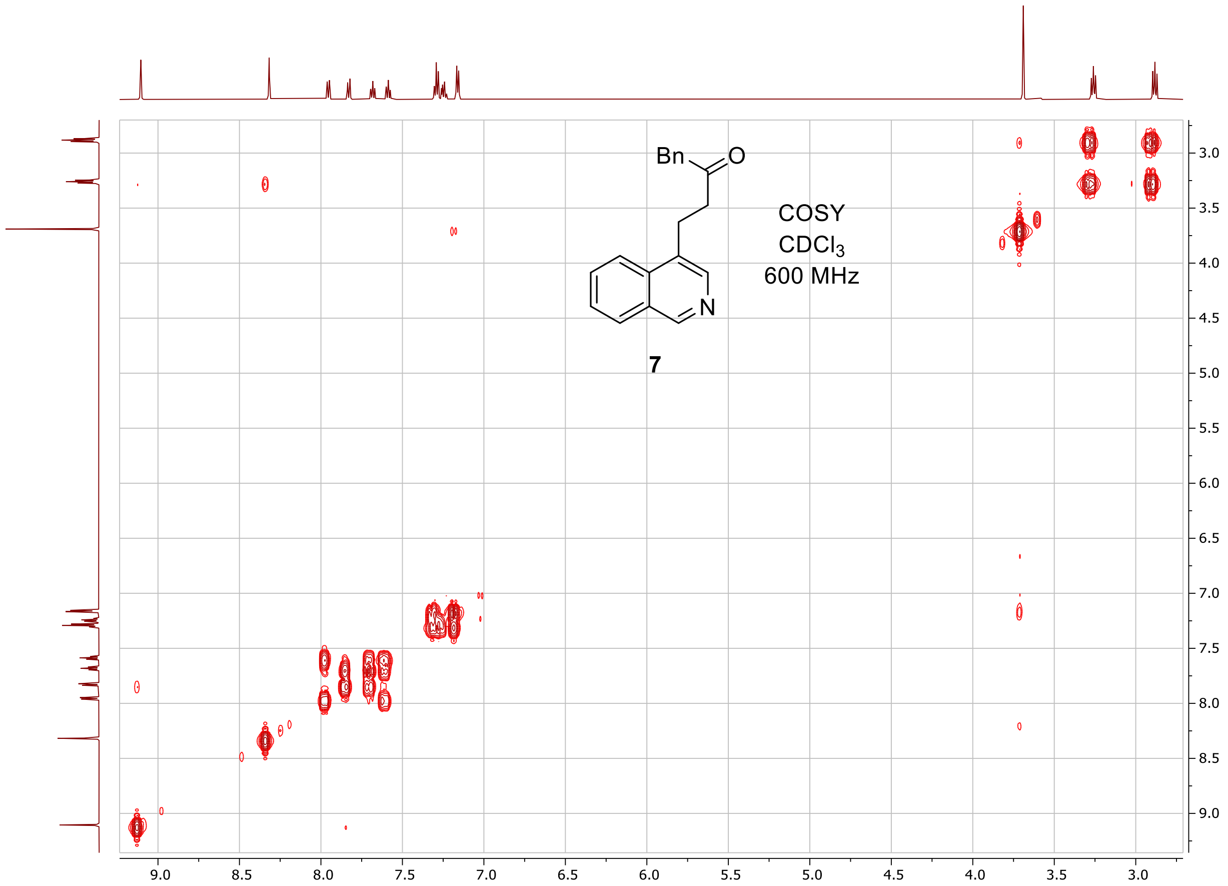

Bn
O
N
7
COSY
$CDCl_3$
600 MHz

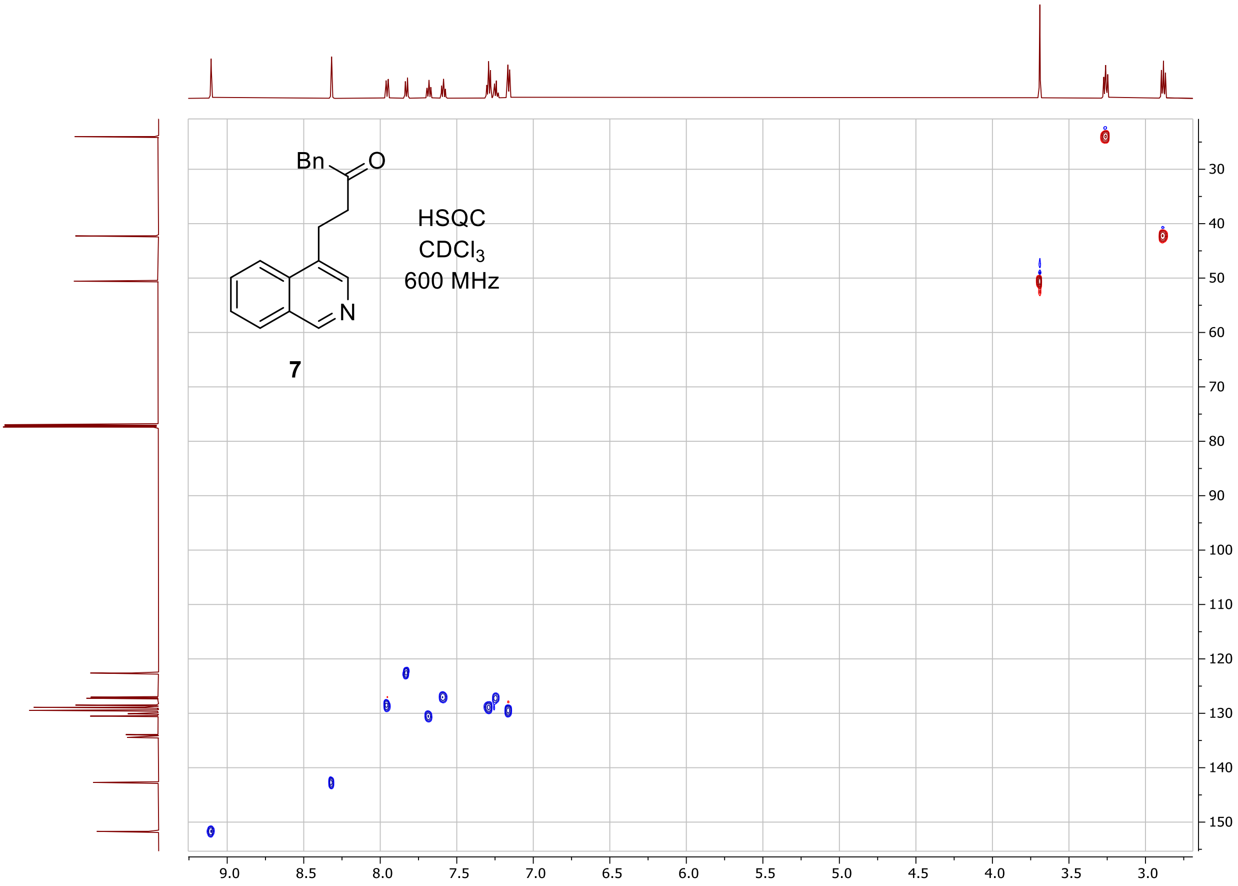

Bn
O
N
7
HSQC
$CDCl_3$
600 MHz

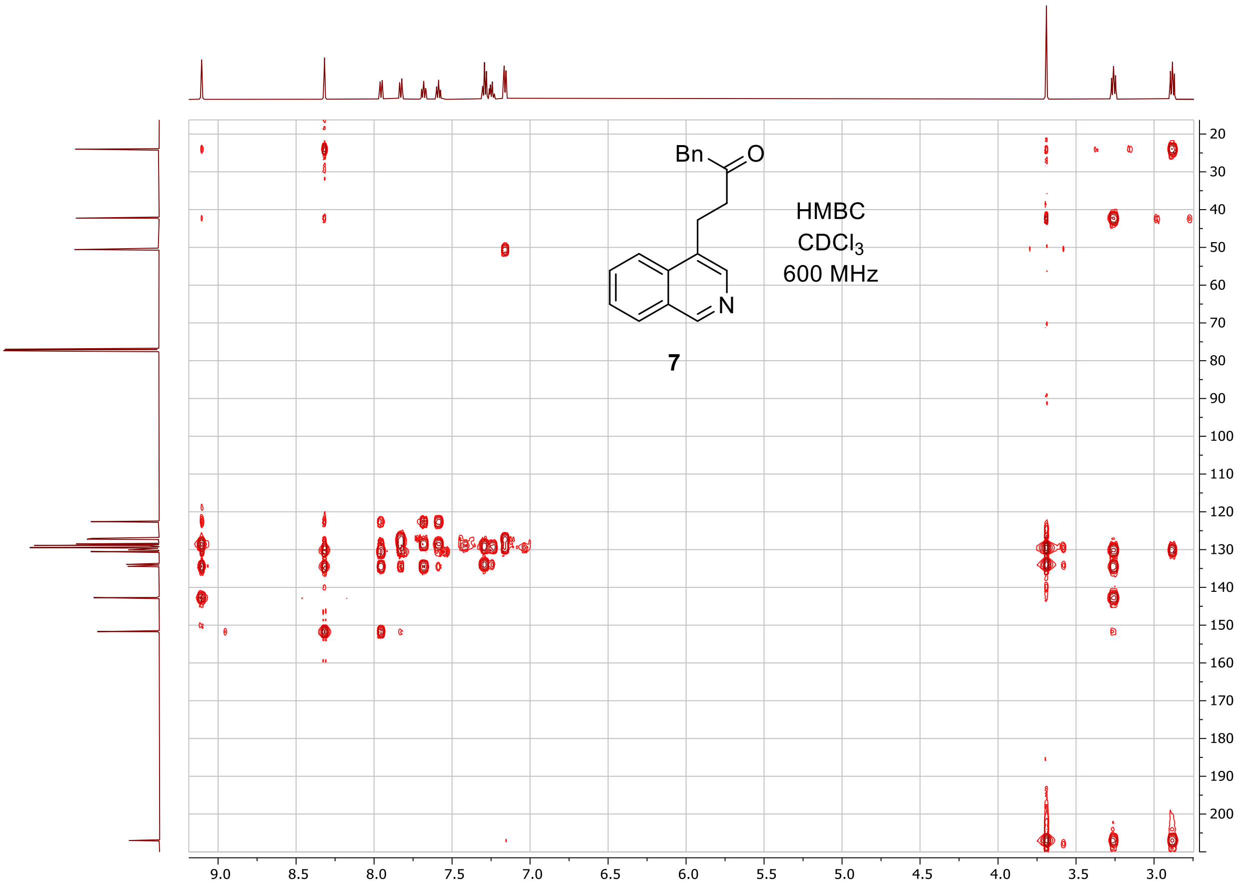
Bn
O
N
HMBC
CDCl3
600 MHz
9.0
8.5
8.0
7.5
7.0
6.5
6.0
5.5
5.0
4.5
4.0
3.5
3.0
20
30
40
50
60
70
80
90
100
110
120
130
140
150
160
170
180
190
200

7

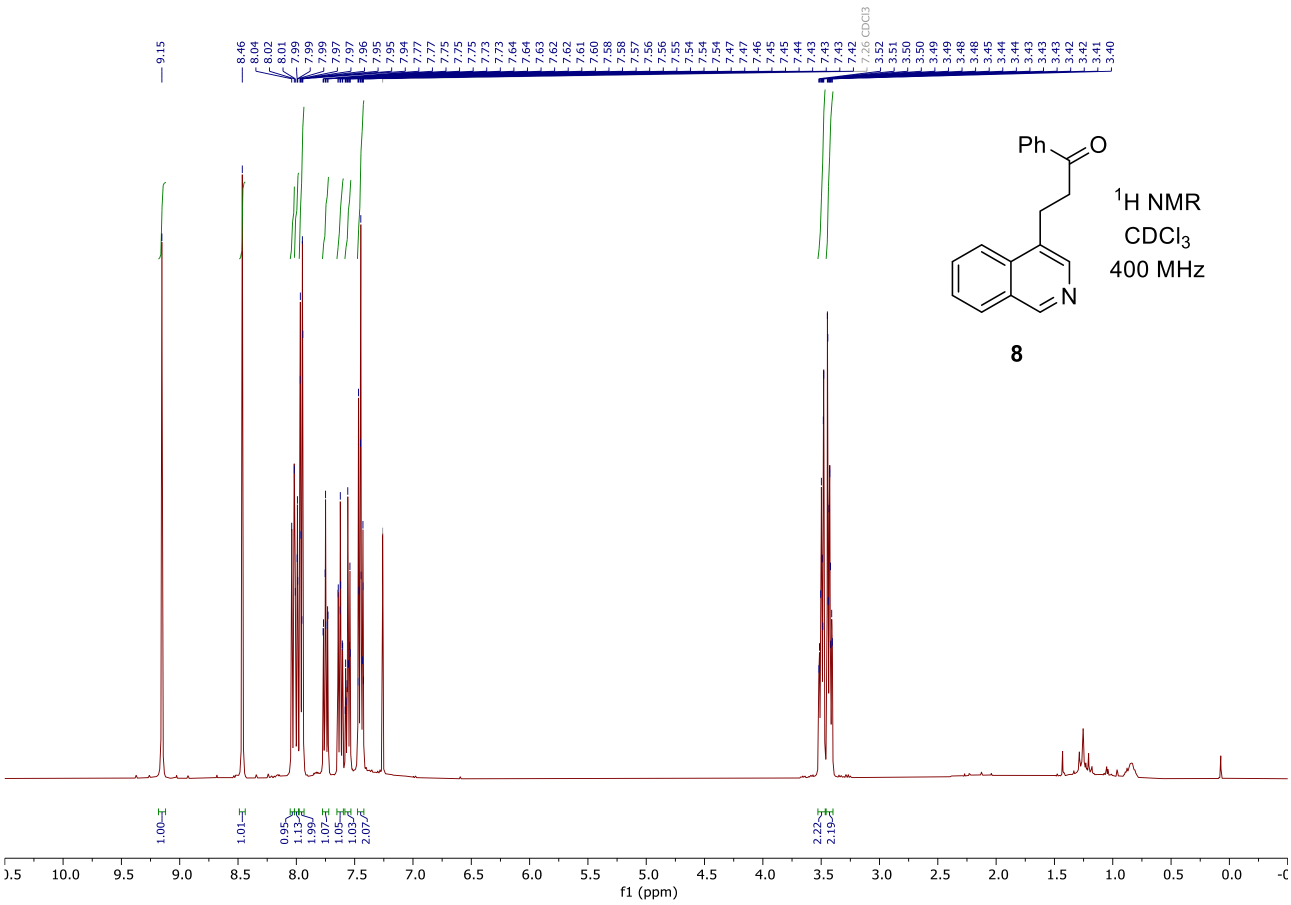

1H NMR
CDCl3
400 MHz
8
f1 (ppm)

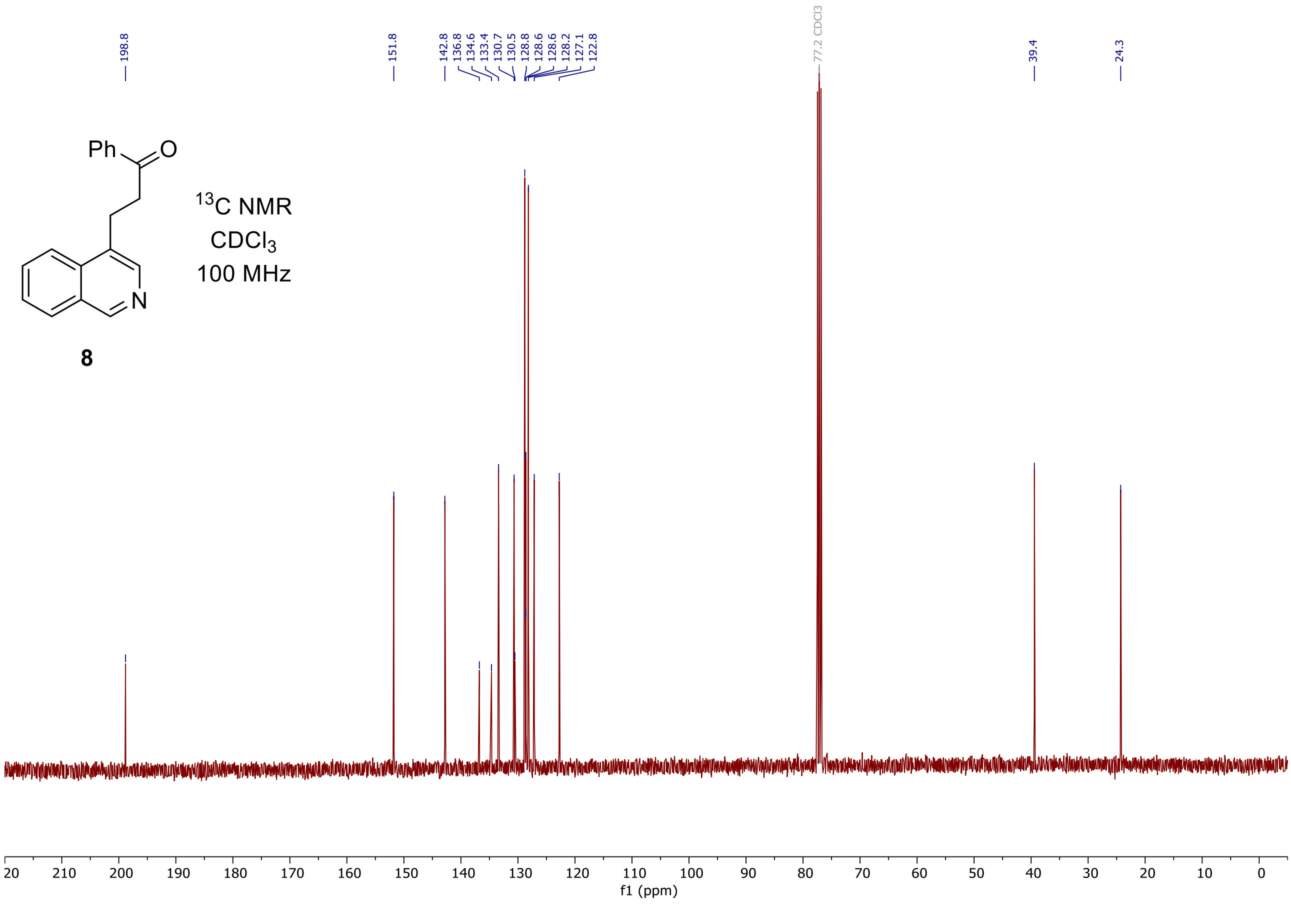

13C NMR
CDCl3
100 MHz
8
f1 (ppm)

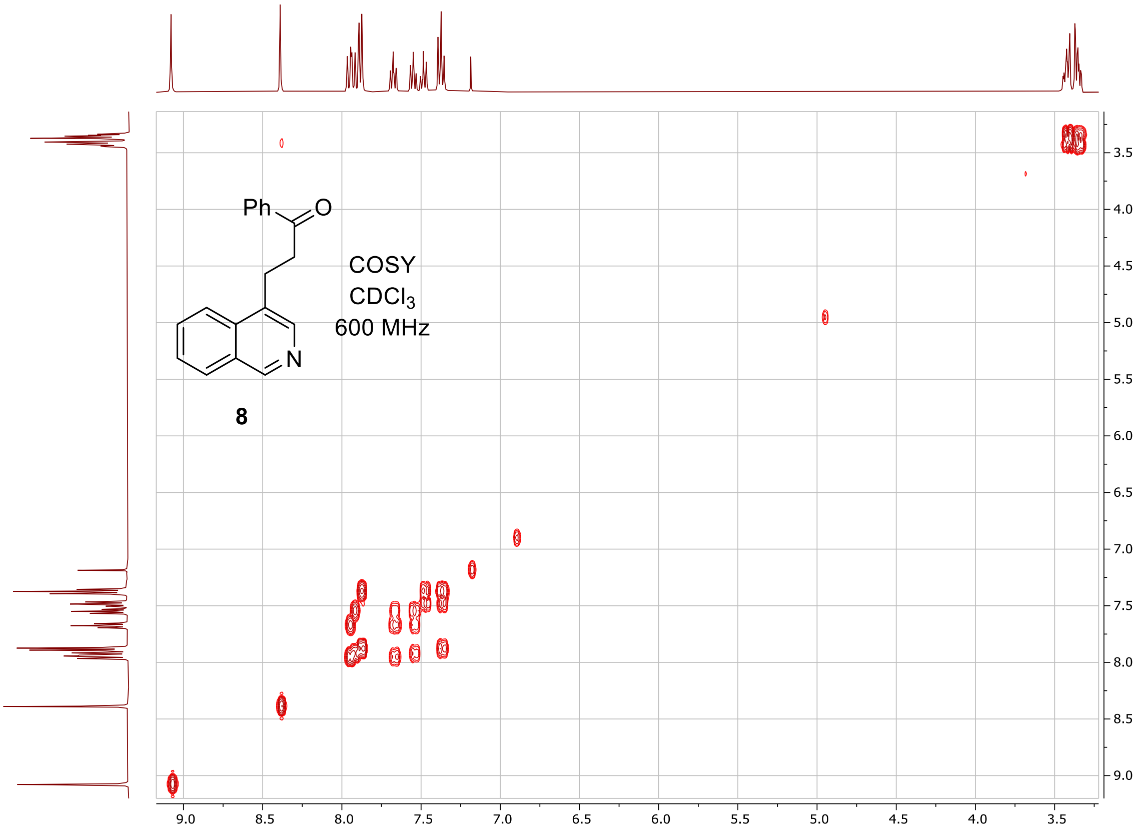
Ph
O
N
8
COSY
CDCl3
600 MHz
9.0 8.5 8.0 7.5 7.0 6.5 6.0 5.5 5.0 4.5 4.0 3.5
3.5 4.0 4.5 5.0 5.5 6.0 6.5 7.0 7.5 8.0 8.5 9.0

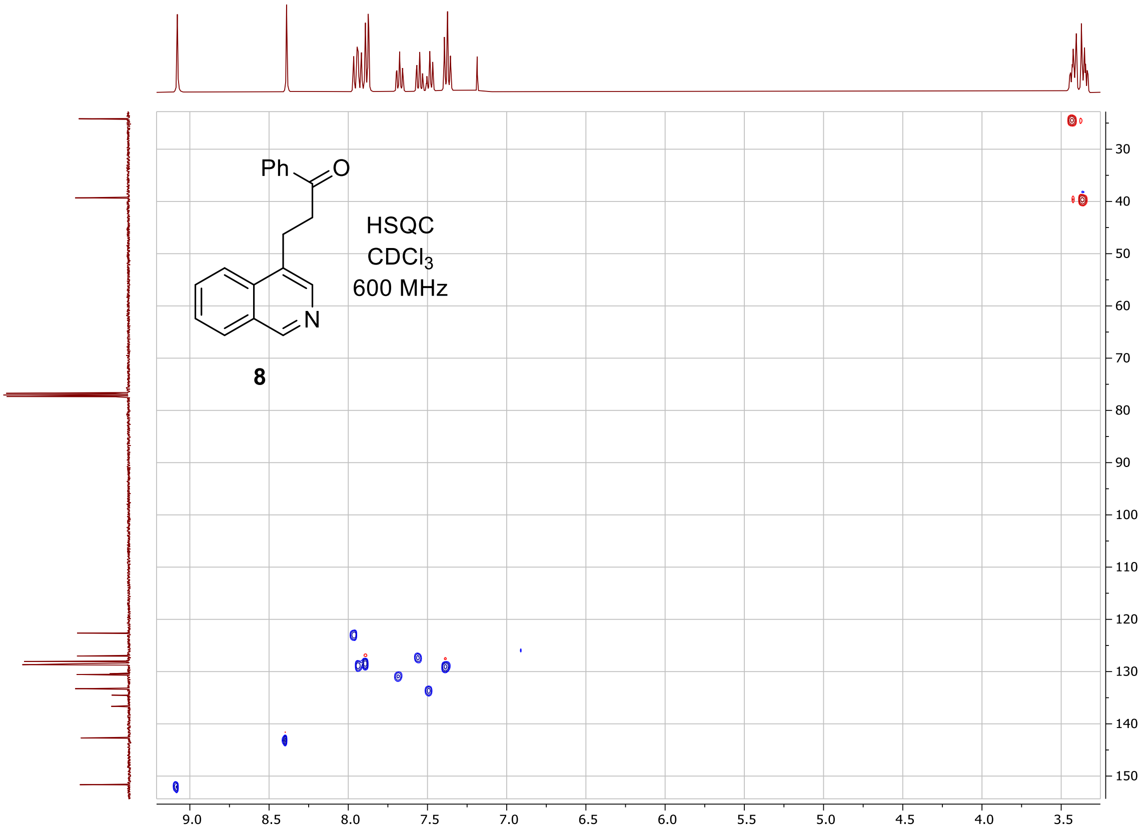
Ph
O
N
8
HSQC
CDCl3
600 MHz
9.0 8.5 8.0 7.5 7.0 6.5 6.0 5.5 5.0 4.5 4.0 3.5
30 40 50 60 70 80 90 100 110 120 130 140 150

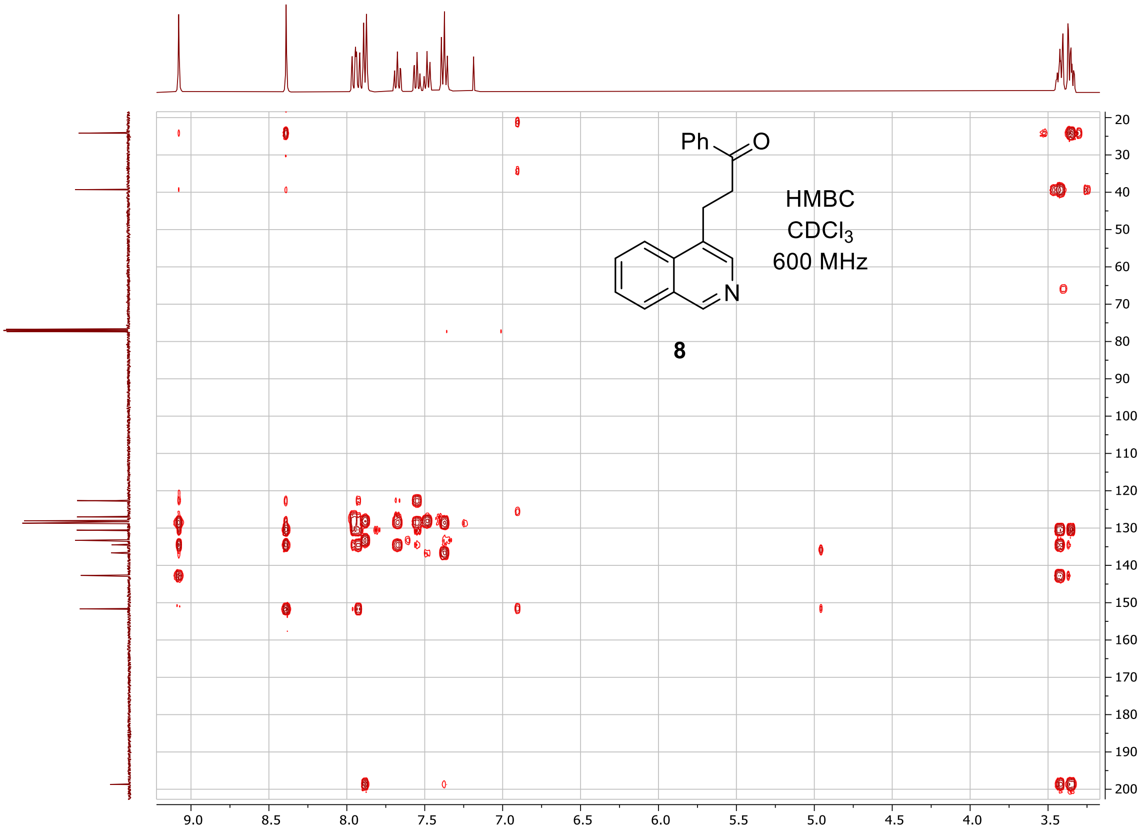
Ph
O
N
8
HMBC
CDCl3
600 MHz
9.0
8.5
8.0
7.5
7.0
6.5
6.0
5.5
5.0
4.5
4.0
3.5
20
30
40
50
60
70
80
90
100
110
120
130
140
150
160
170
180
190
200

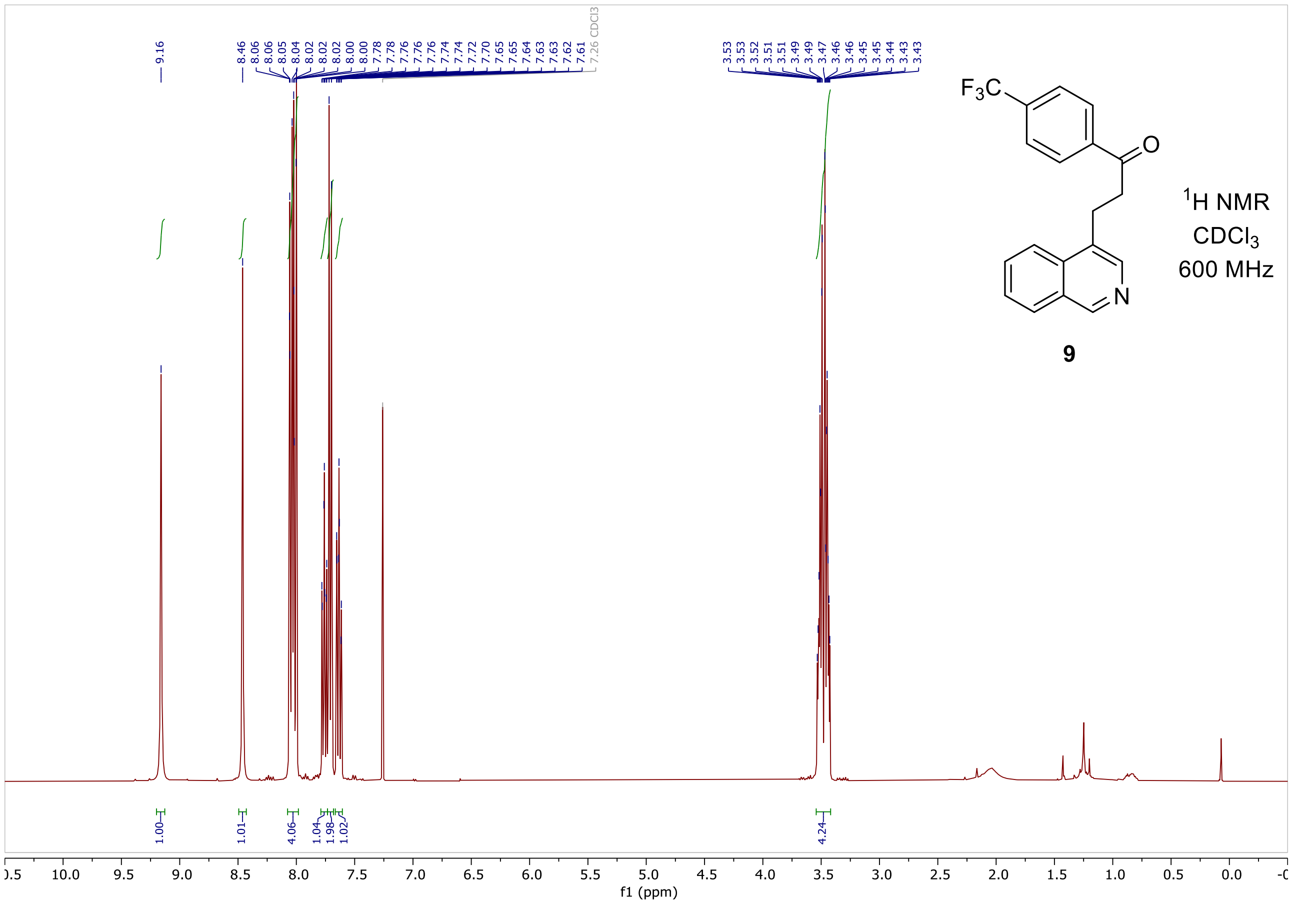

1H NMR
CDCl3
600 MHz
9
f1 (ppm)

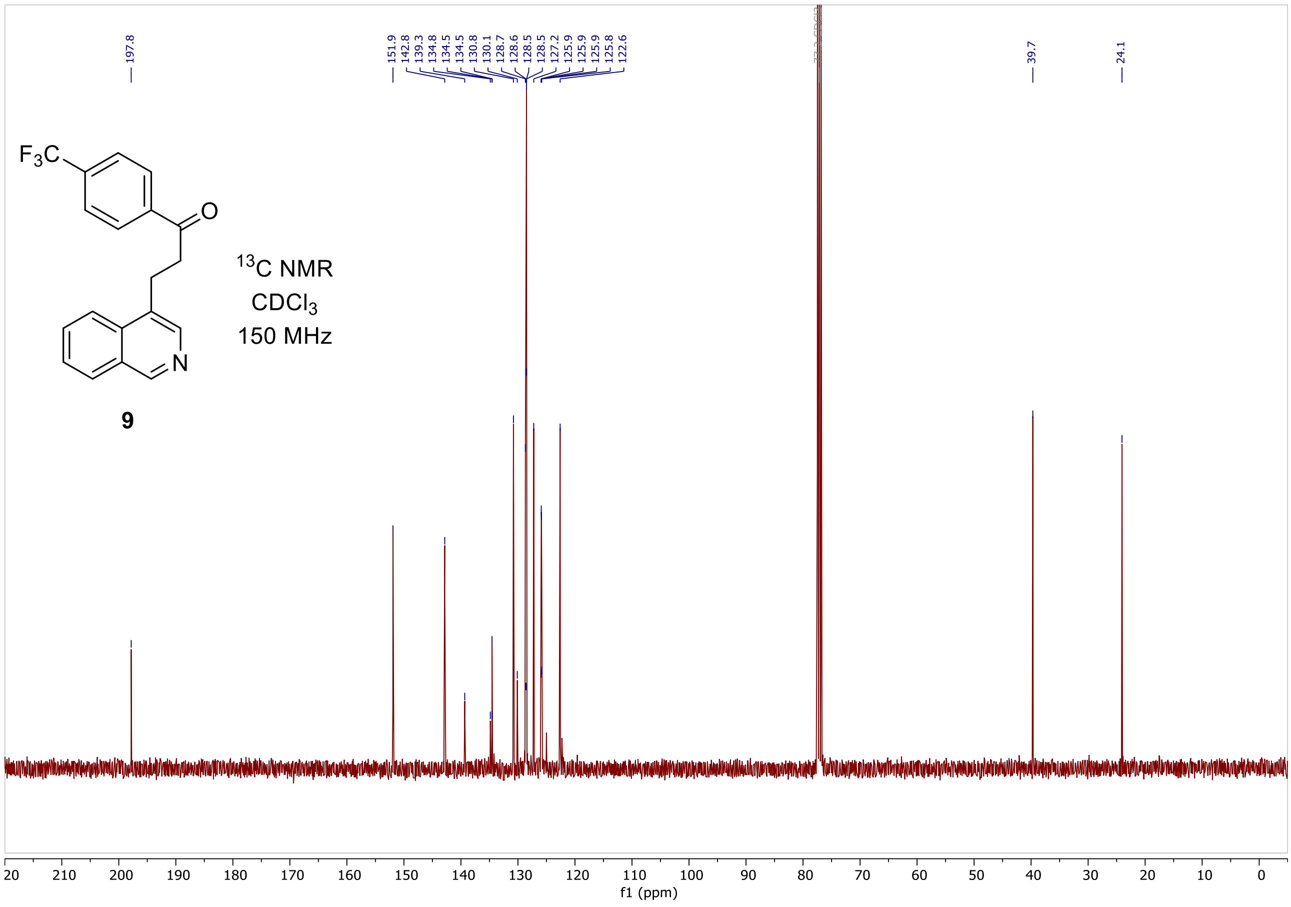

13C NMR
CDCl3
150 MHz
9
f1 (ppm)

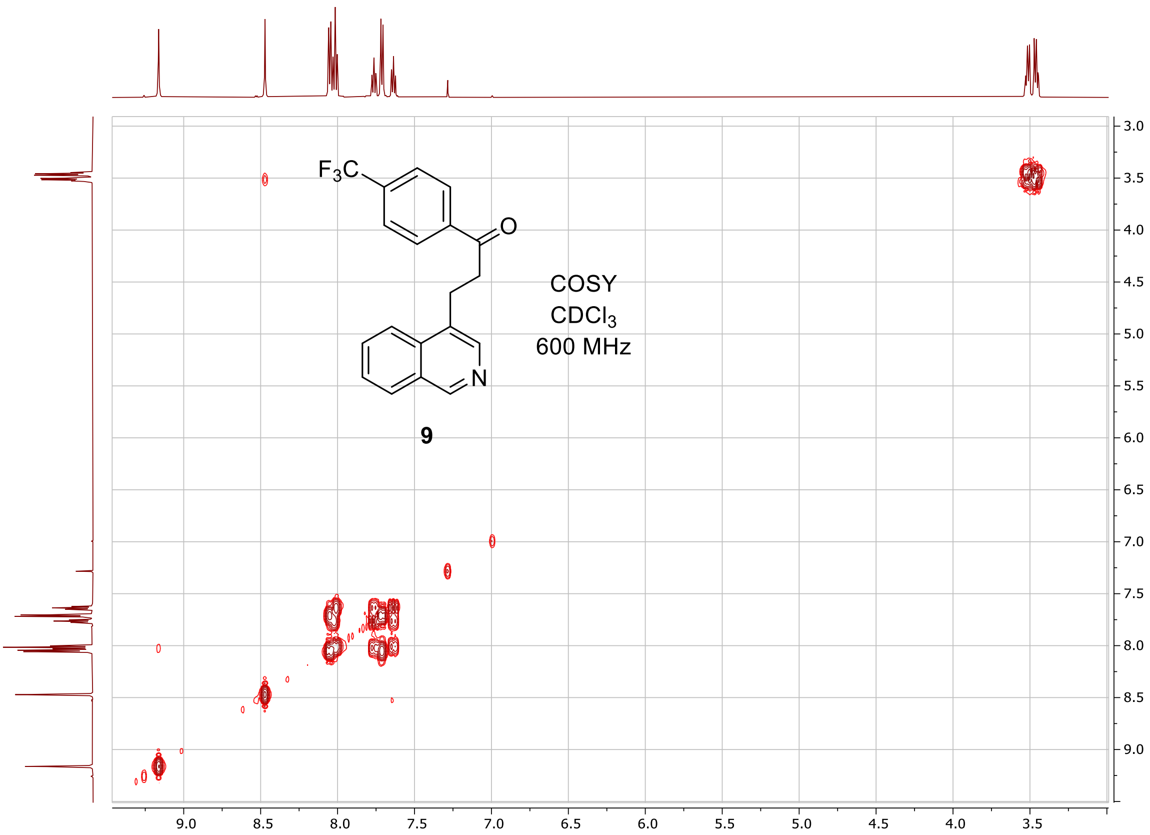
COSY
$CDCl_3$
600 MHz
9

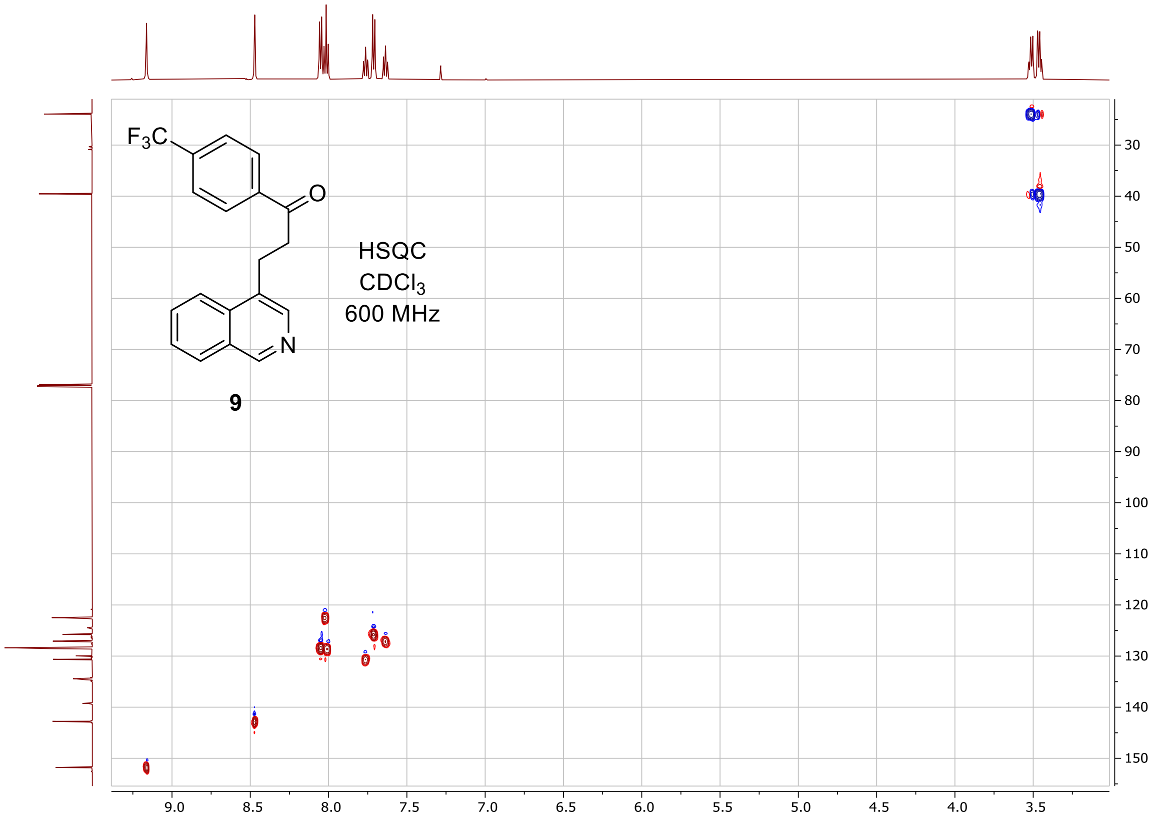
HSQC
$CDCl_3$
600 MHz
9

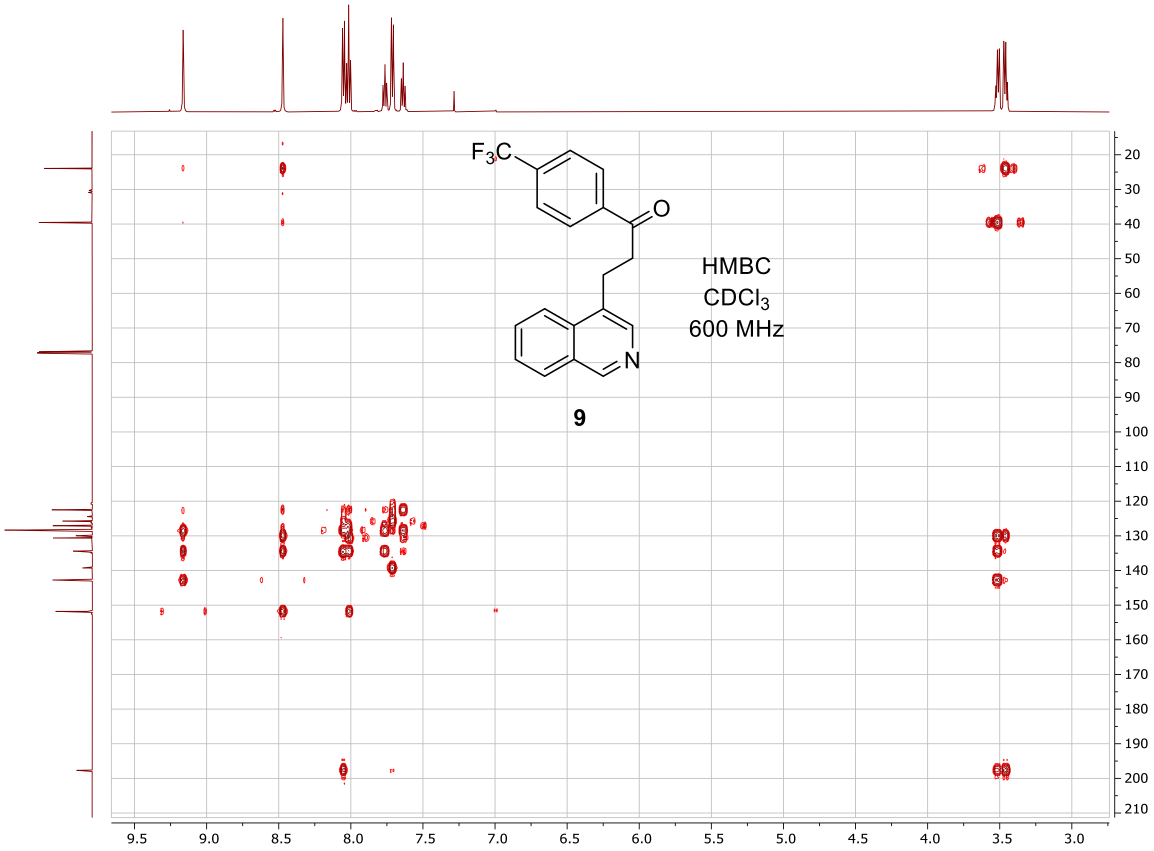
F3C
O
N
9
HMBC
CDCl3
600 MHz
9.5 9.0 8.5 8.0 7.5 7.0 6.5 6.0 5.5 5.0 4.5 4.0 3.5 3.0
20 30 40 50 60 70 80 90 100 110 120 130 140 150 160 170 180 190 200 210

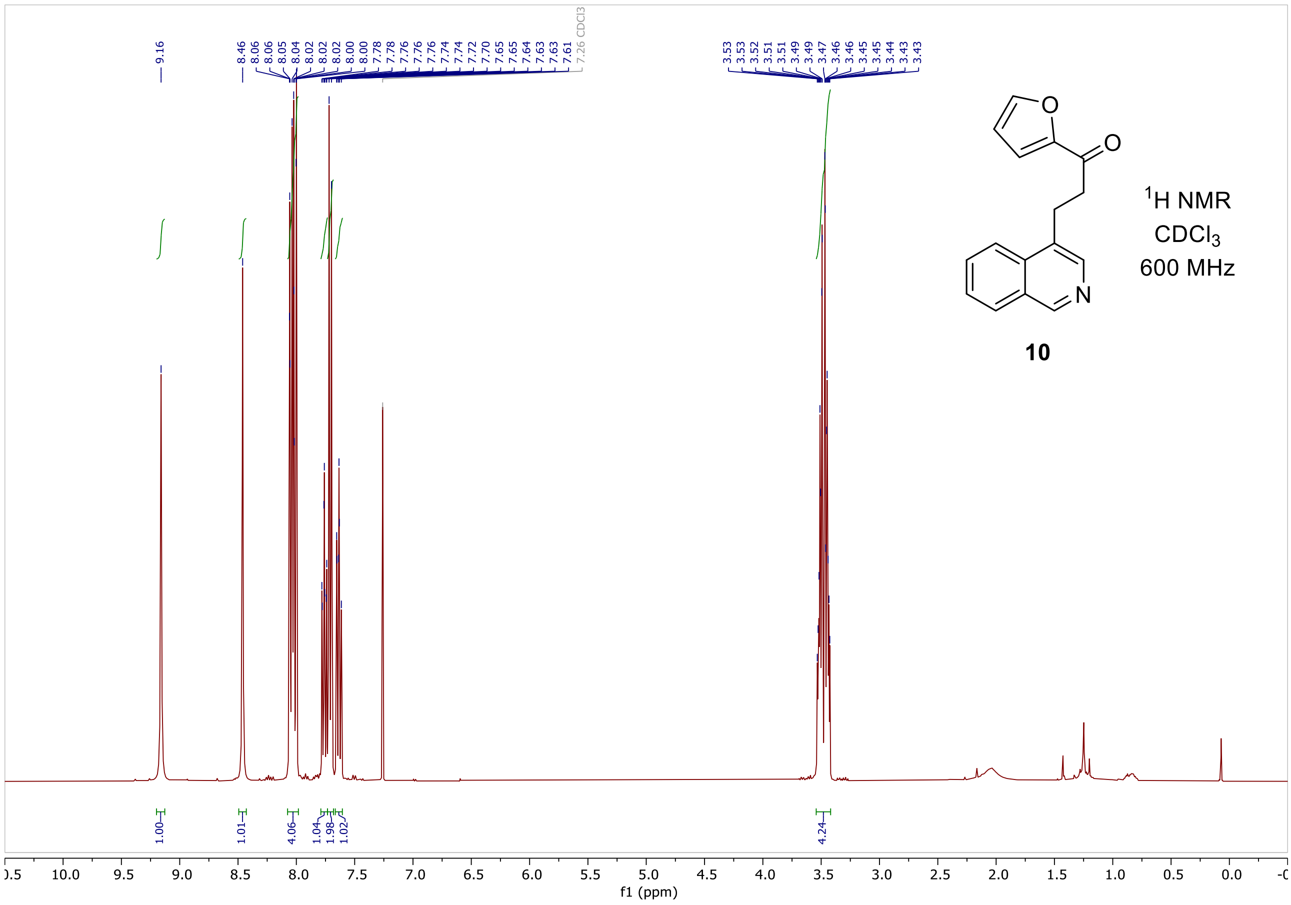
9.16
7.26 CDCl3
1H NMR
CDCl3
600 MHz
10
f1 (ppm)

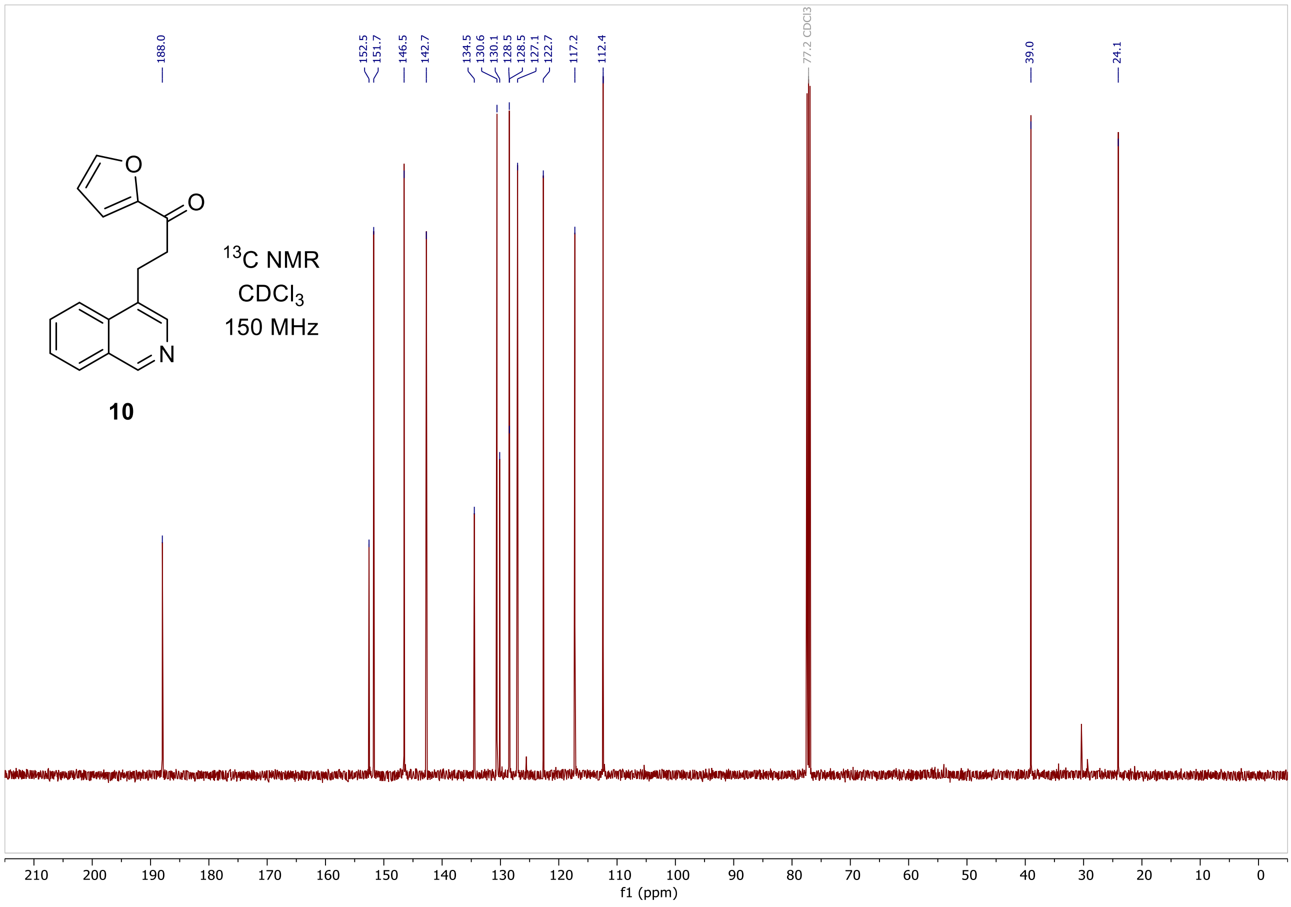
188.0
152.5
151.7
146.5
142.7
134.5
130.6
130.1
128.5
128.5
127.1
122.7
117.2
112.4
77.2 CDCl3
39.0
24.1
13C NMR
CDCl3
150 MHz
10
f1 (ppm)

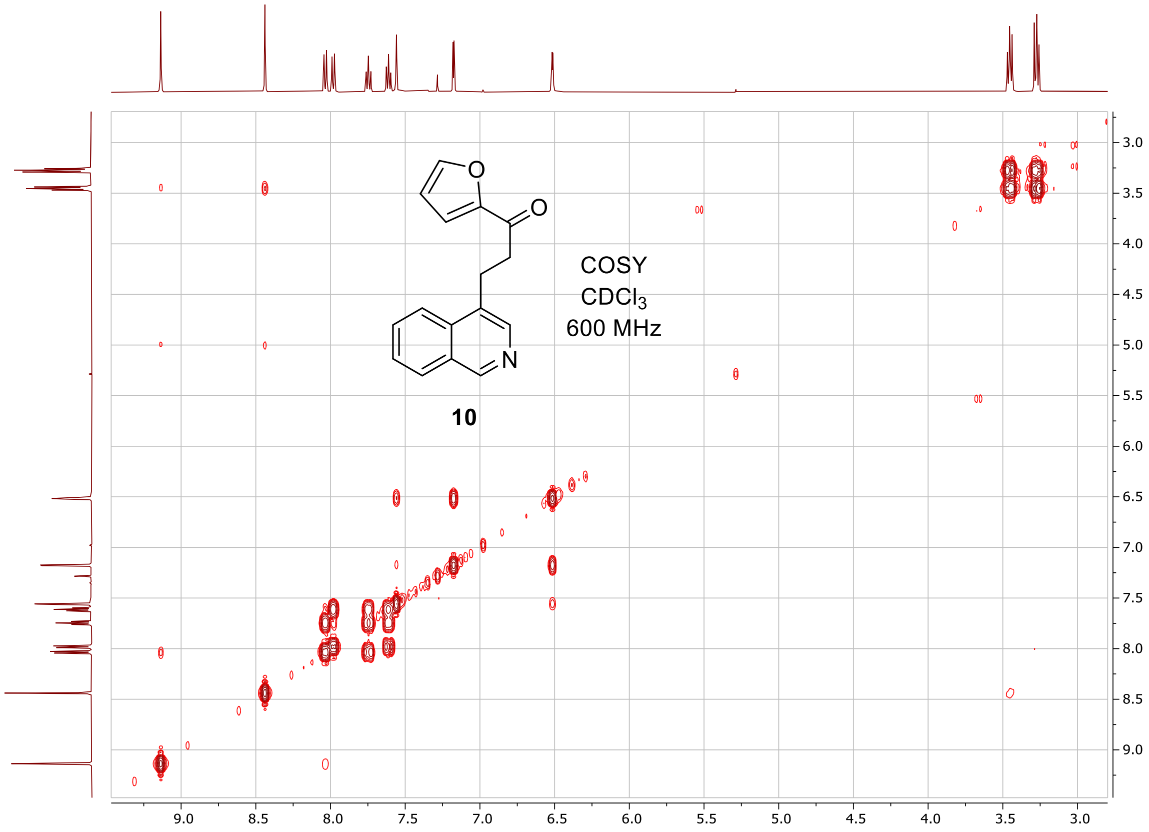

COSY
CDCl3
600 MHz
10
O
O
N
9.0
8.5
8.0
7.5
7.0
6.5
6.0
5.5
5.0
4.5
4.0
3.5
3.0

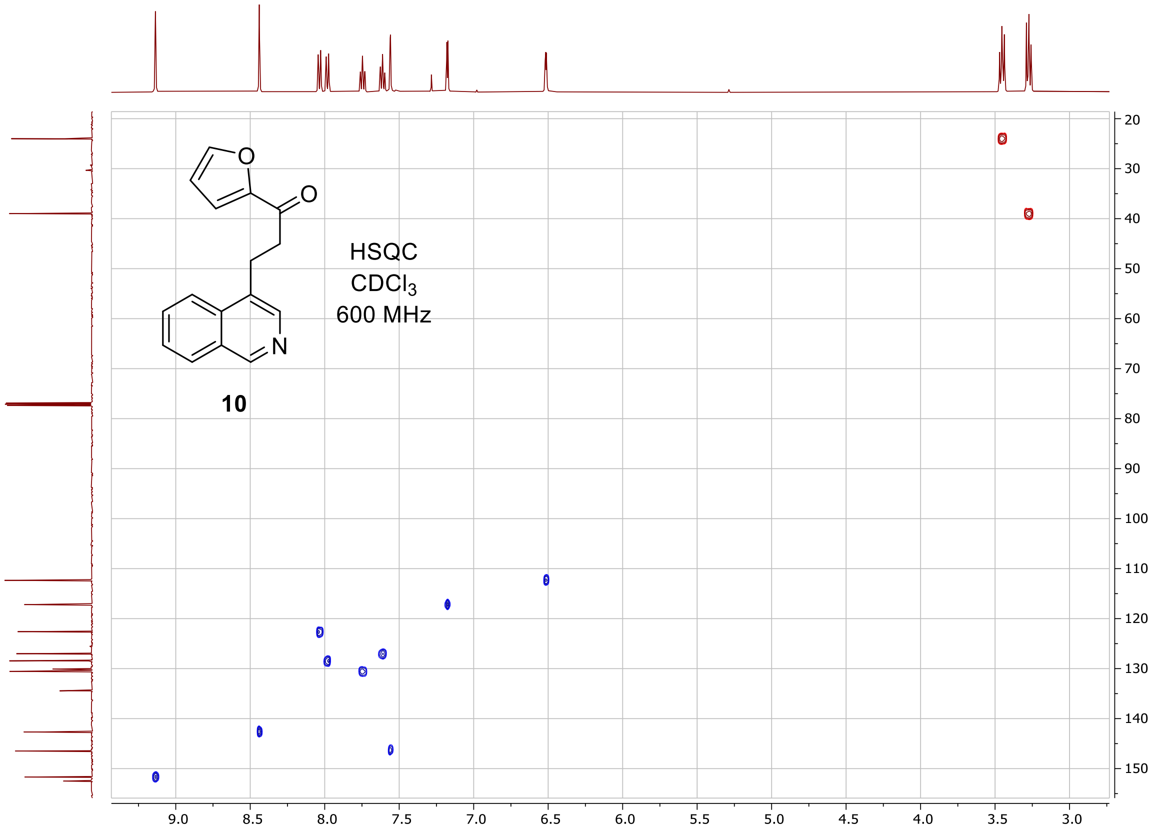

HSQC
CDCl3
600 MHz
10
O
O
N
20
30
40
50
60
70
80
90
100
110
120
130
140
150
9.0
8.5
8.0
7.5
7.0
6.5
6.0
5.5
5.0
4.5
4.0
3.5
3.0

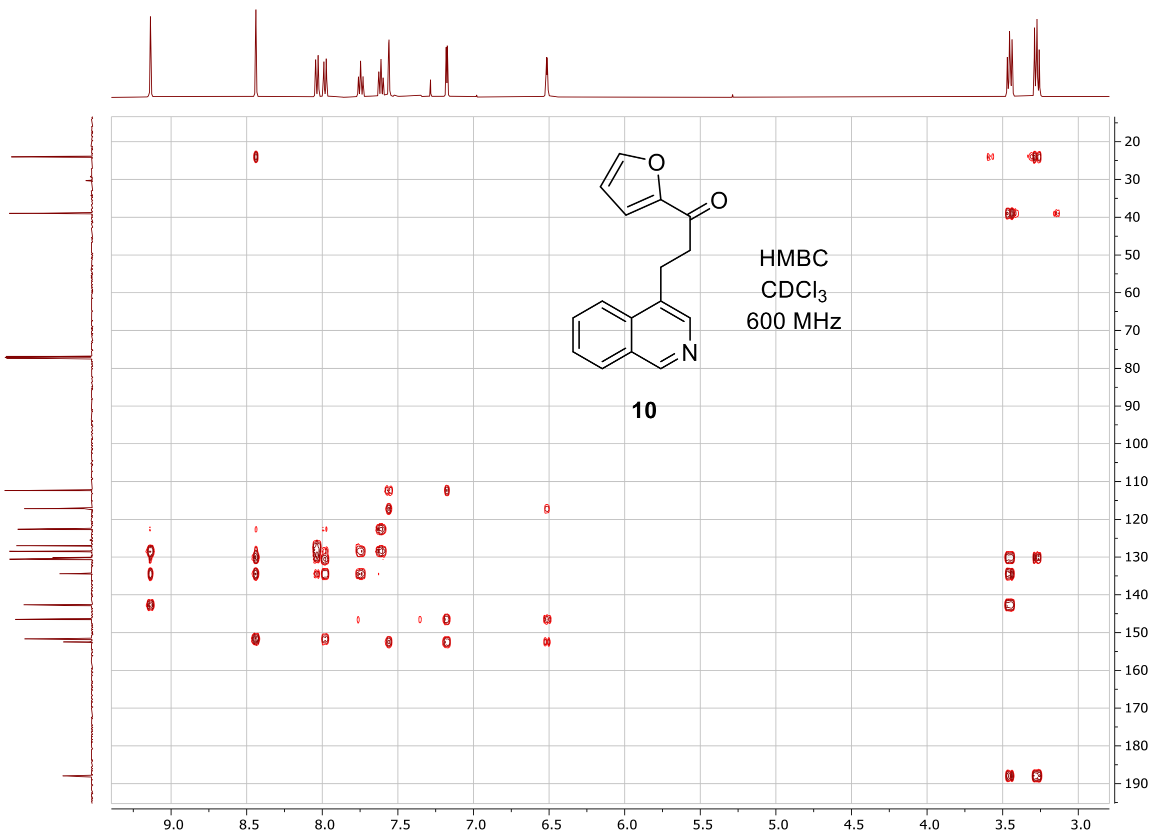
HMBC
CDCl3
600 MHz
10
N
O
O

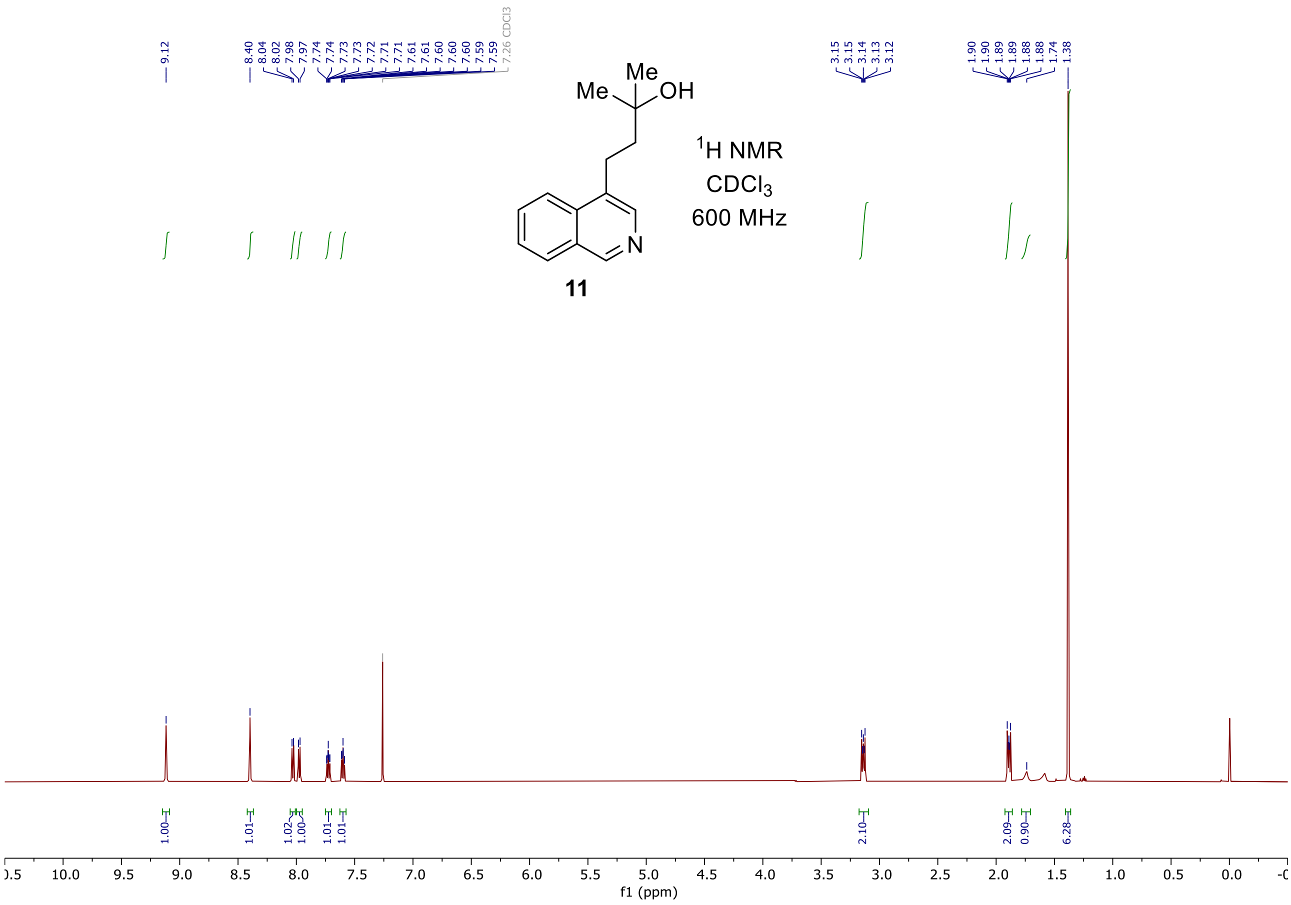

Me
Me
OH
N
11
^{1}H NMR
CDCl3
600 MHz
f1 (ppm)

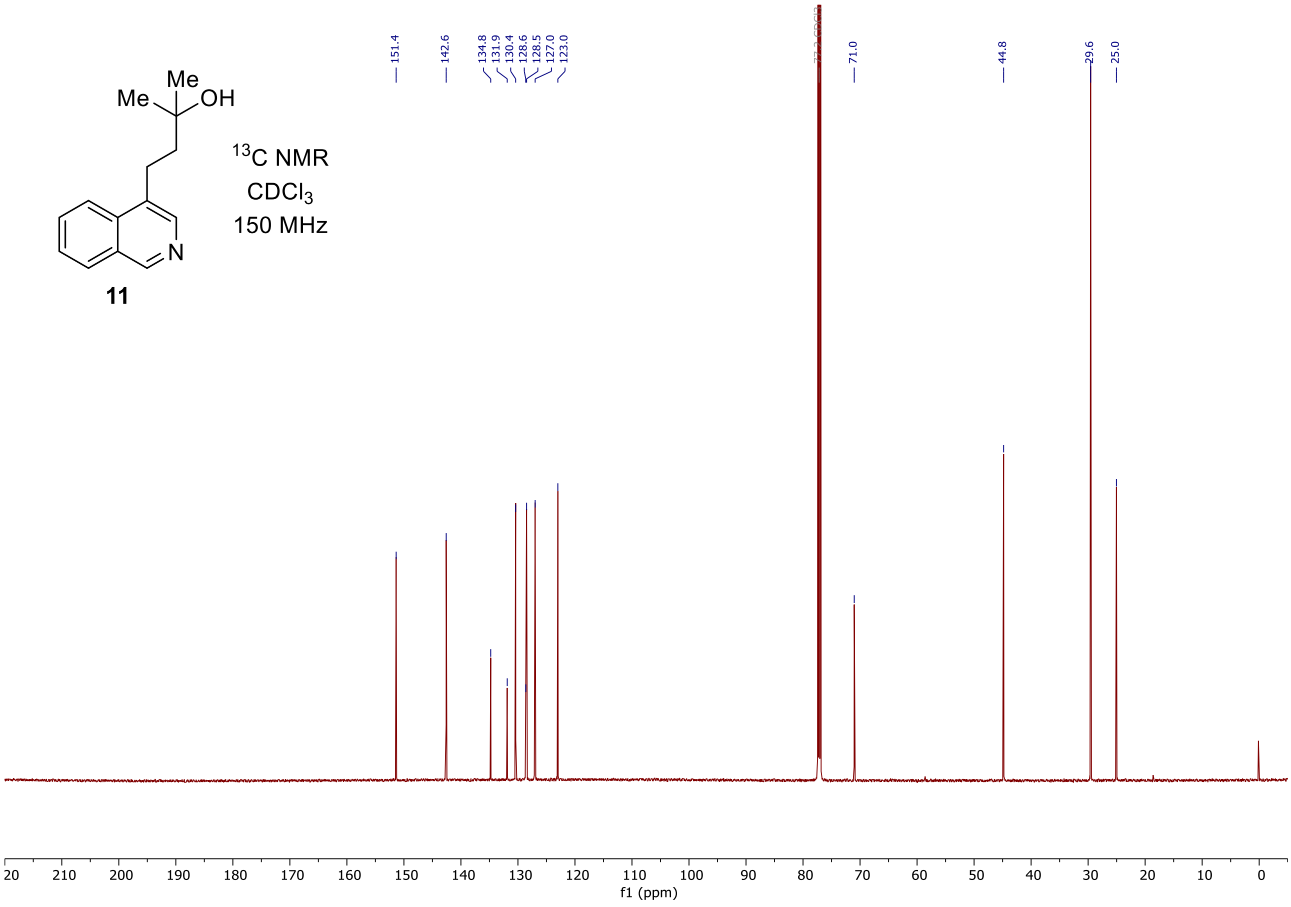

Me
Me
OH
N
11
^{13}C NMR
CDCl3
150 MHz
f1 (ppm)

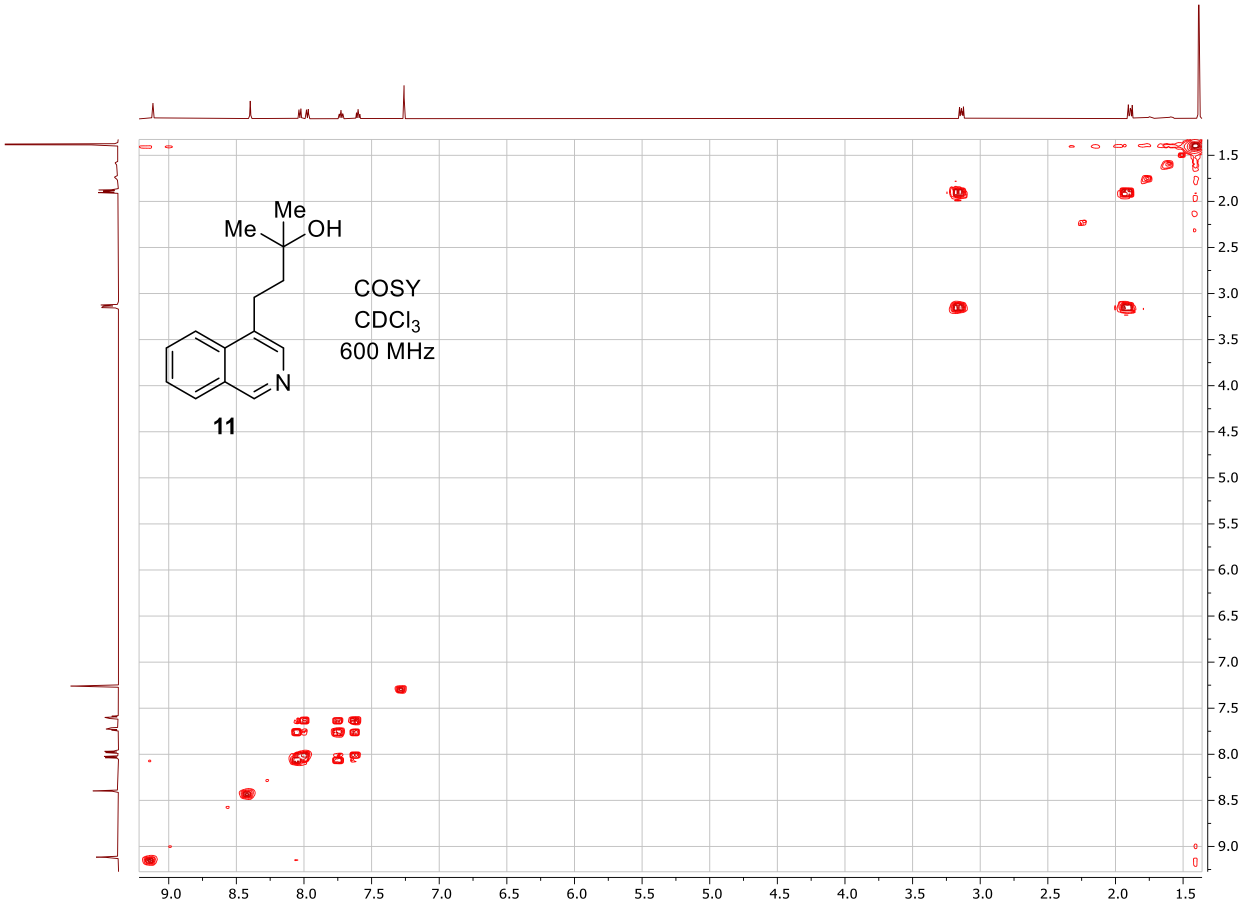
Me
Me
OH
N
11
COSY
CDCl3
600 MHz
9.0 8.5 8.0 7.5 7.0 6.5 6.0 5.5 5.0 4.5 4.0 3.5 3.0 2.5 2.0 1.5
1.5 2.0 2.5 3.0 3.5 4.0 4.5 5.0 5.5 6.0 6.5 7.0 7.5 8.0 8.5 9.0

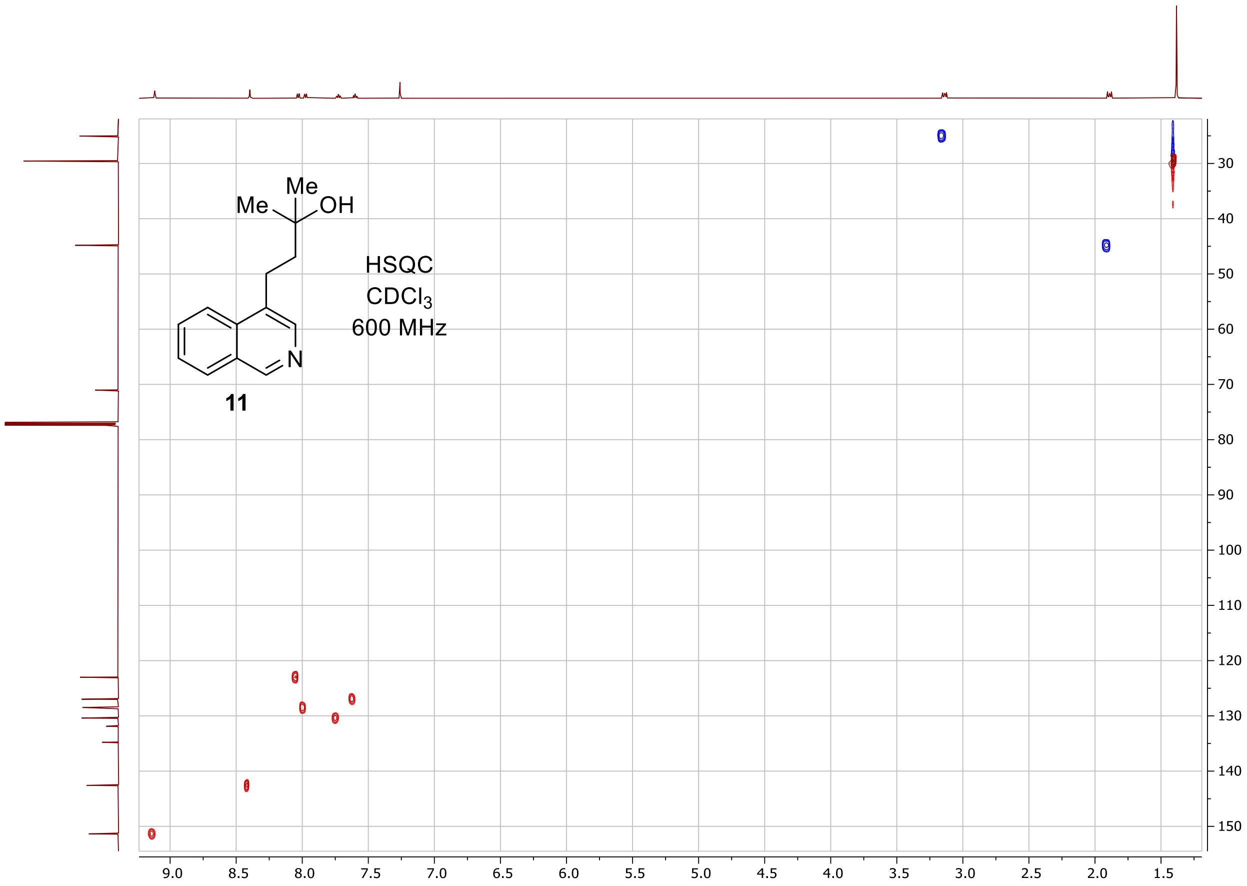
Me
Me
OH
N
11
HSQC
CDCl3
600 MHz
9.0 8.5 8.0 7.5 7.0 6.5 6.0 5.5 5.0 4.5 4.0 3.5 3.0 2.5 2.0 1.5
30 40 50 60 70 80 90 100 110 120 130 140 150

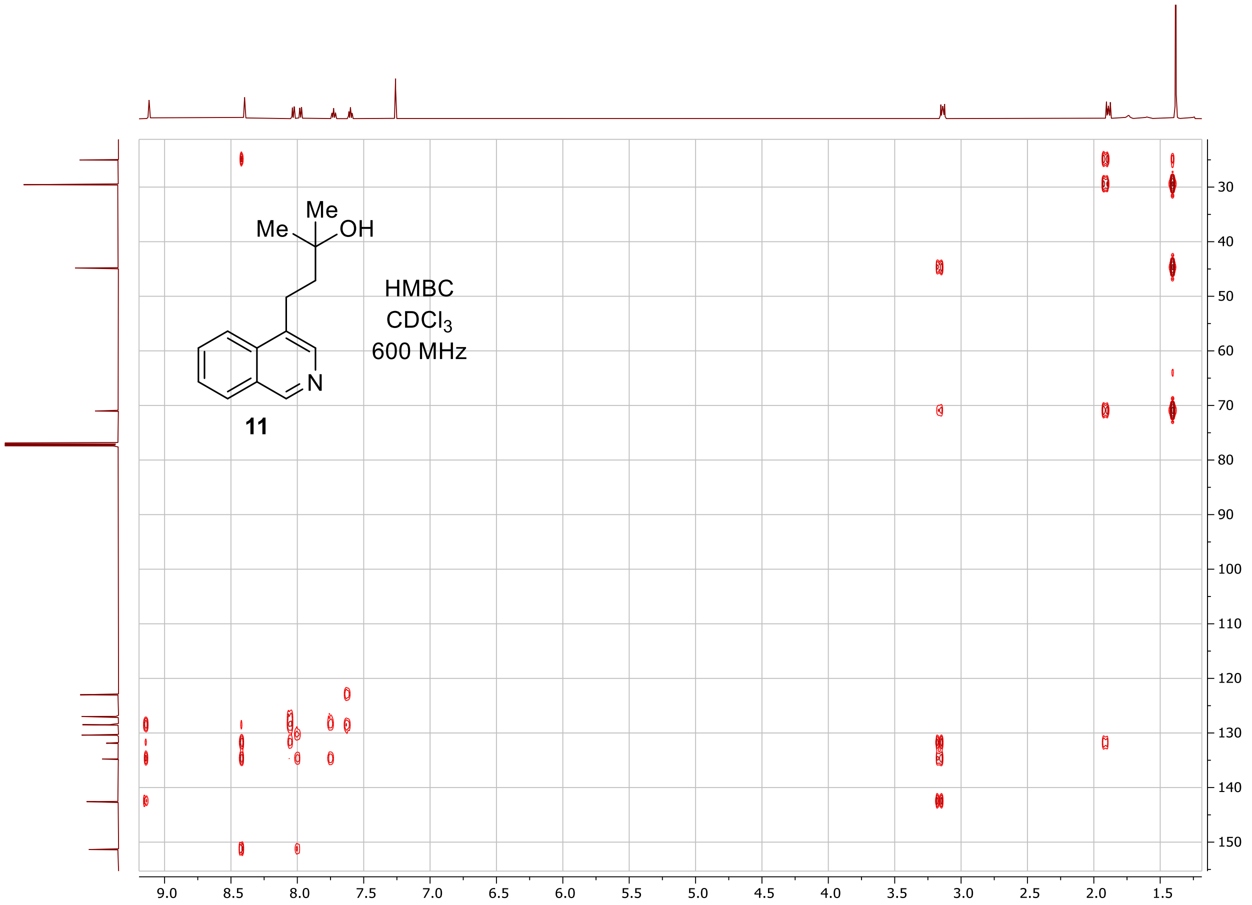

Me
Me
OH
N
11
HMBC
$CDCl_3$
600 MHz

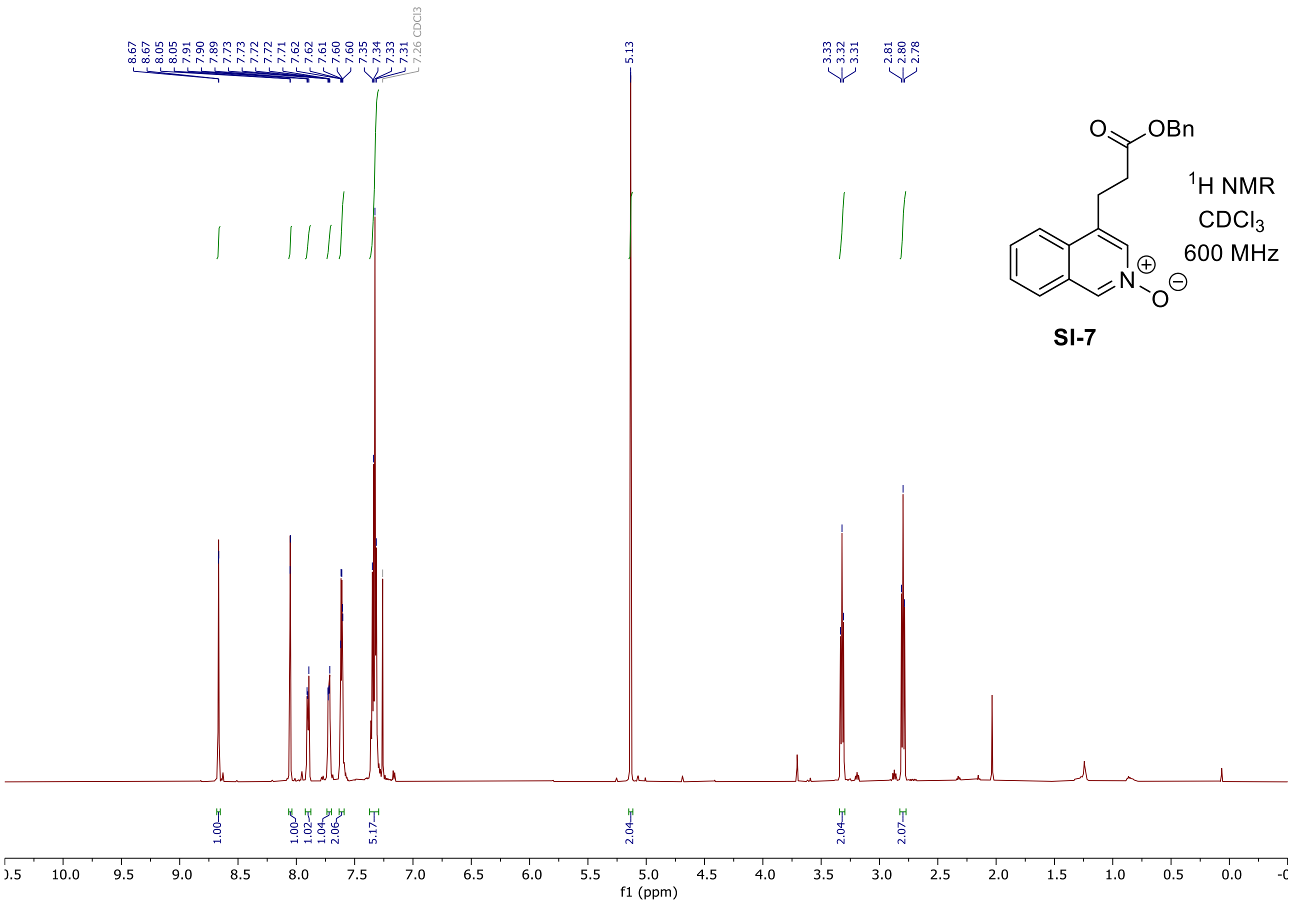

8.67
8.67
8.05
8.05
7.91
7.90
7.89
7.73
7.73
7.72
7.72
7.71
7.62
7.62
7.61
7.60
7.60
7.35
7.34
7.33
7.31
7.26 CDCl3
5.13
3.33
3.32
3.31
2.81
2.80
2.78
O
OBn
N
O
SI-7
1H NMR
CDCl3
600 MHz
1.00
1.00
1.02
1.04
2.06
5.17
2.04
2.04
2.07
f1 (ppm)

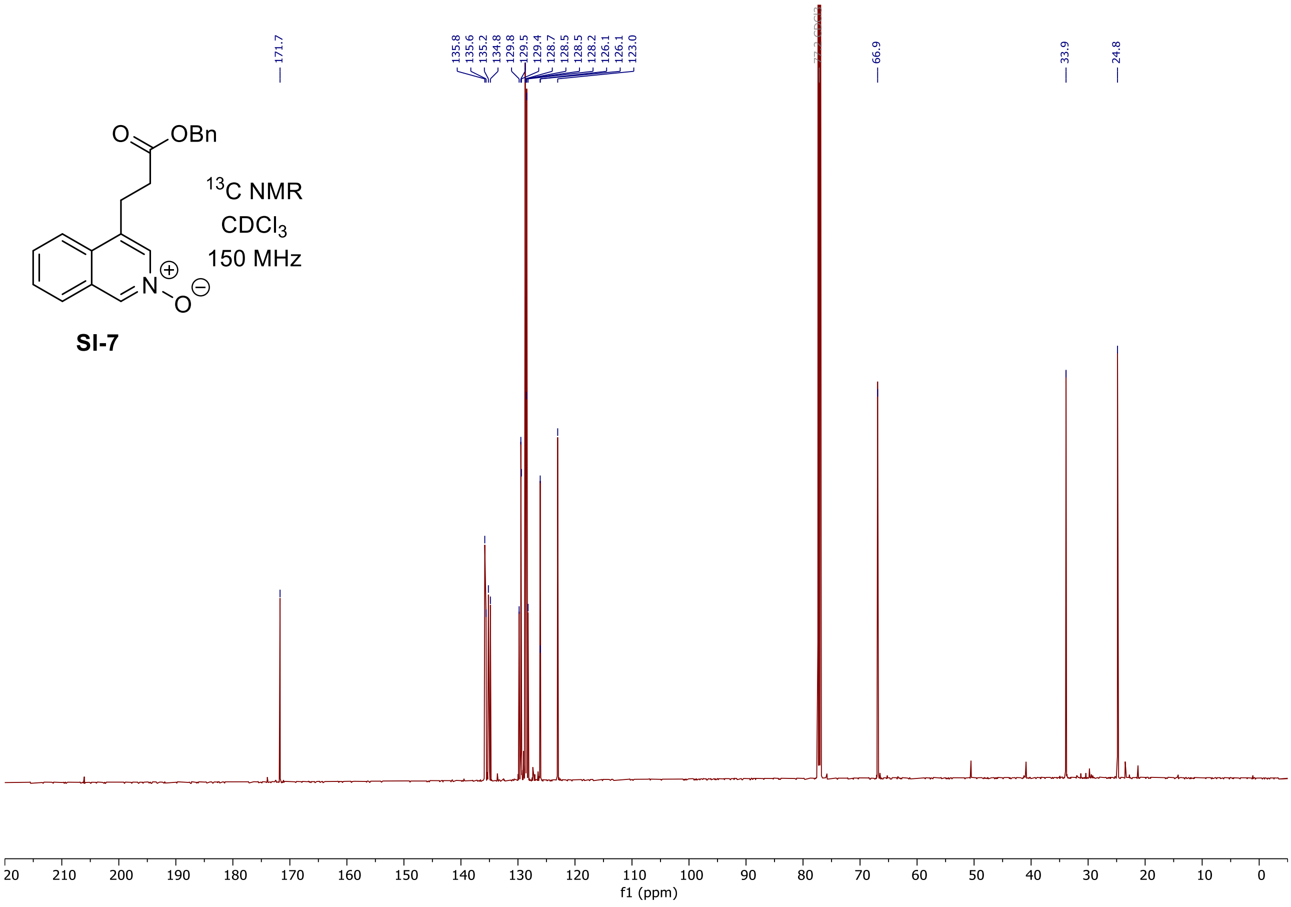

171.7
135.8
135.6
135.2
134.8
129.8
129.5
129.4
128.7
128.5
128.5
128.2
126.1
126.1
123.0
66.9
33.9
24.8
O
OBn
N
O
SI-7
13C NMR
CDCl3
150 MHz
f1 (ppm)

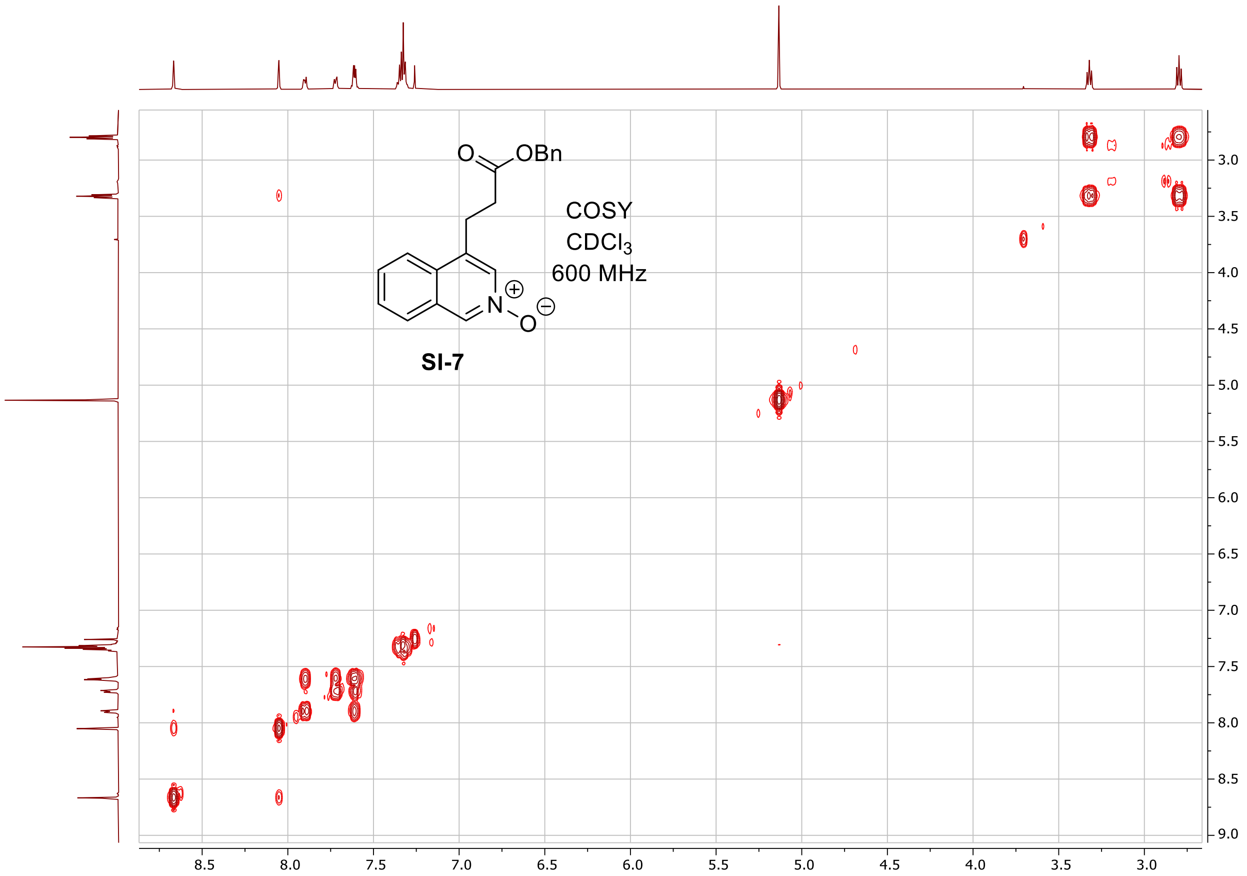

O
OBn
COSY
CDCl3
600 MHz
N
O
SI-7

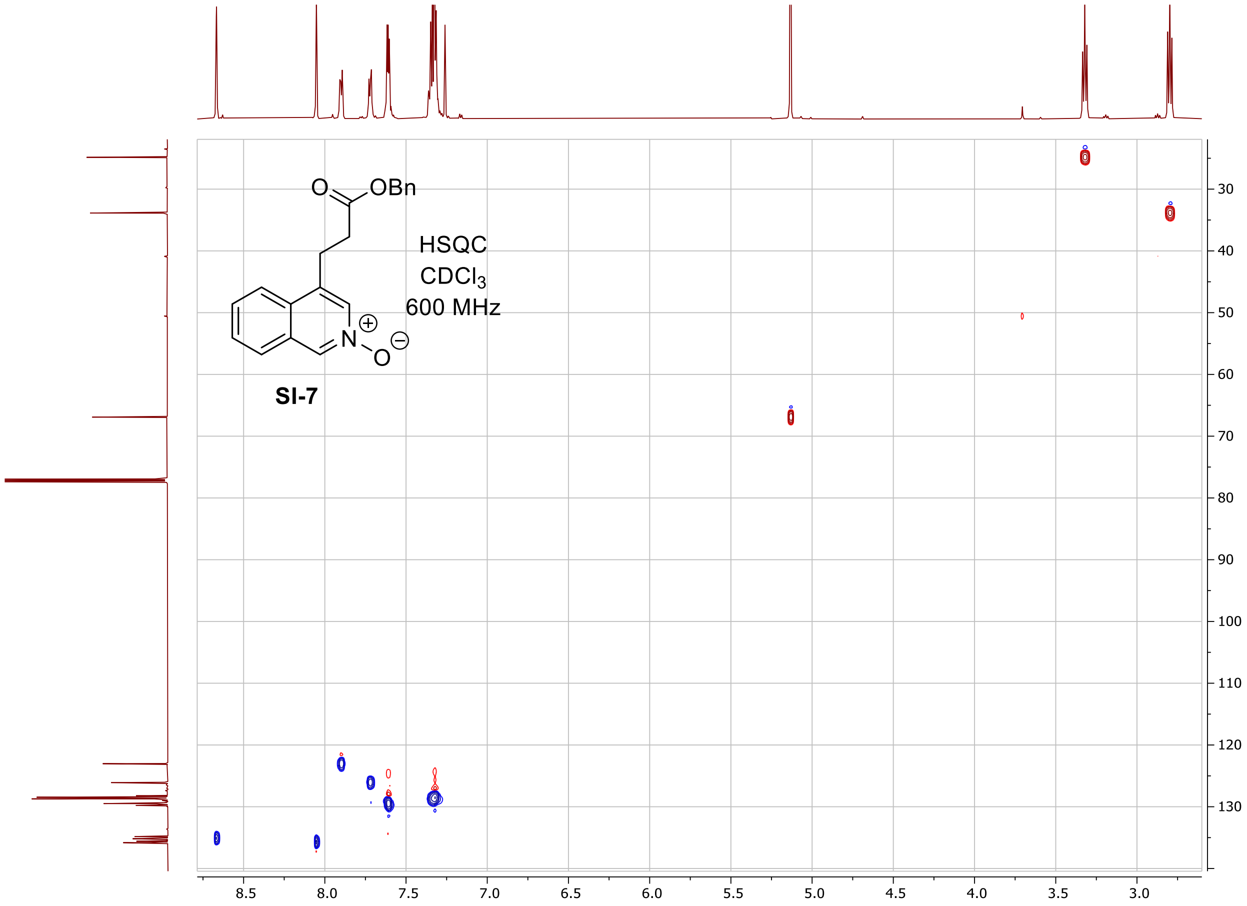

O
OBn
HSQC
CDCl3
600 MHz
N
O
SI-7

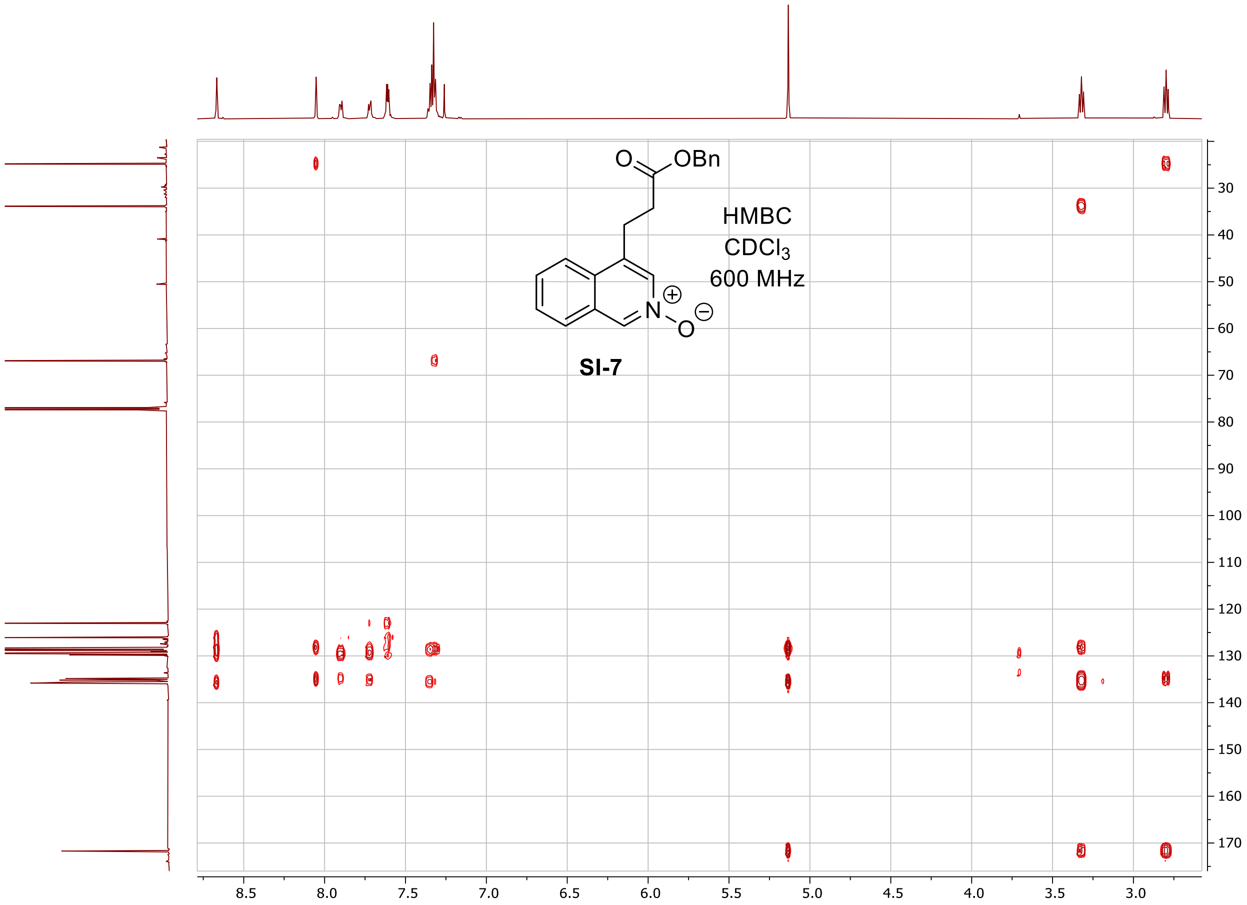

O
OBn
N
O
SI-7
HMBC
CDCl3
600 MHz

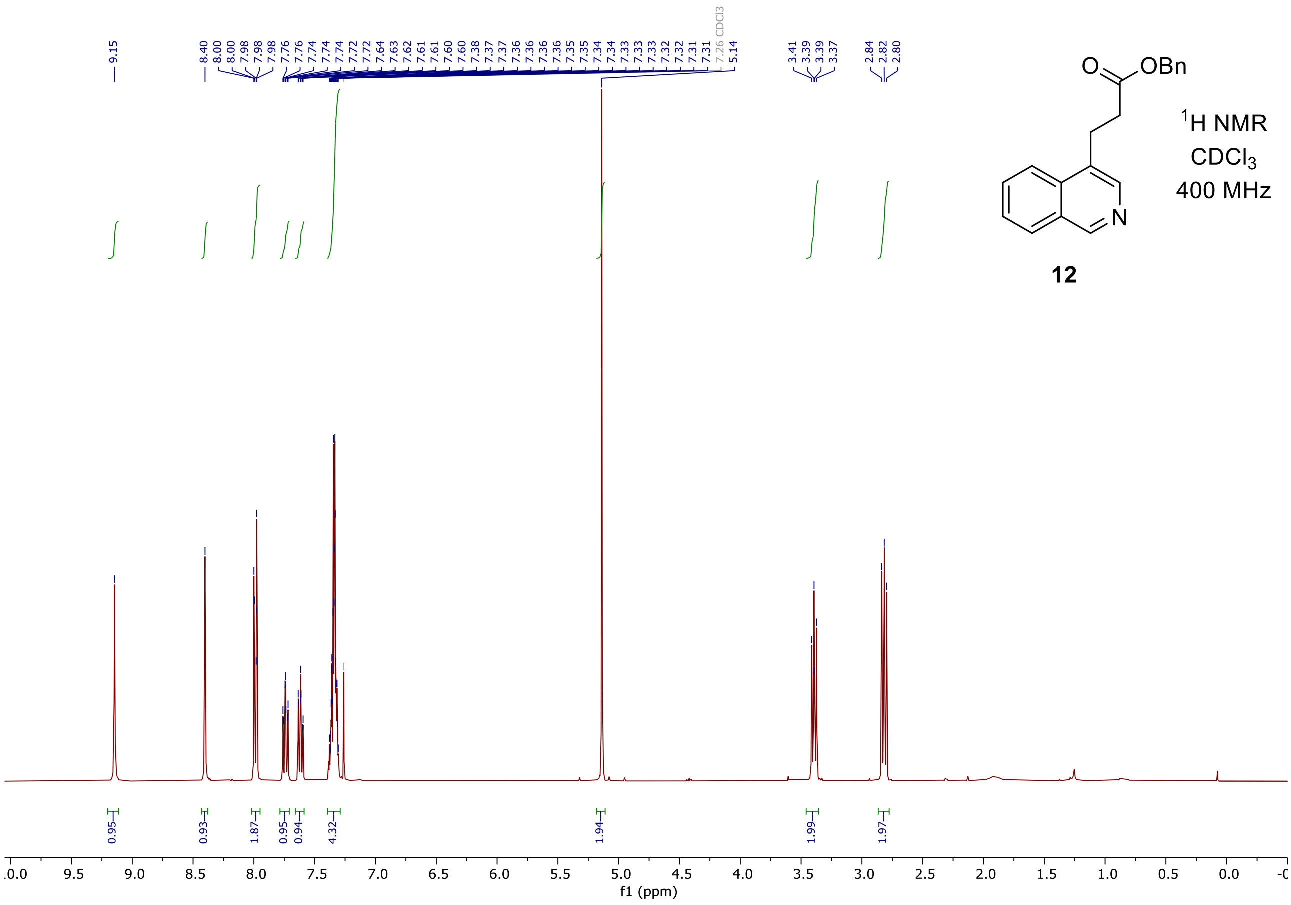

OBn
O
N
12
¹H NMR
CDCl3
400 MHz
f1 (ppm)

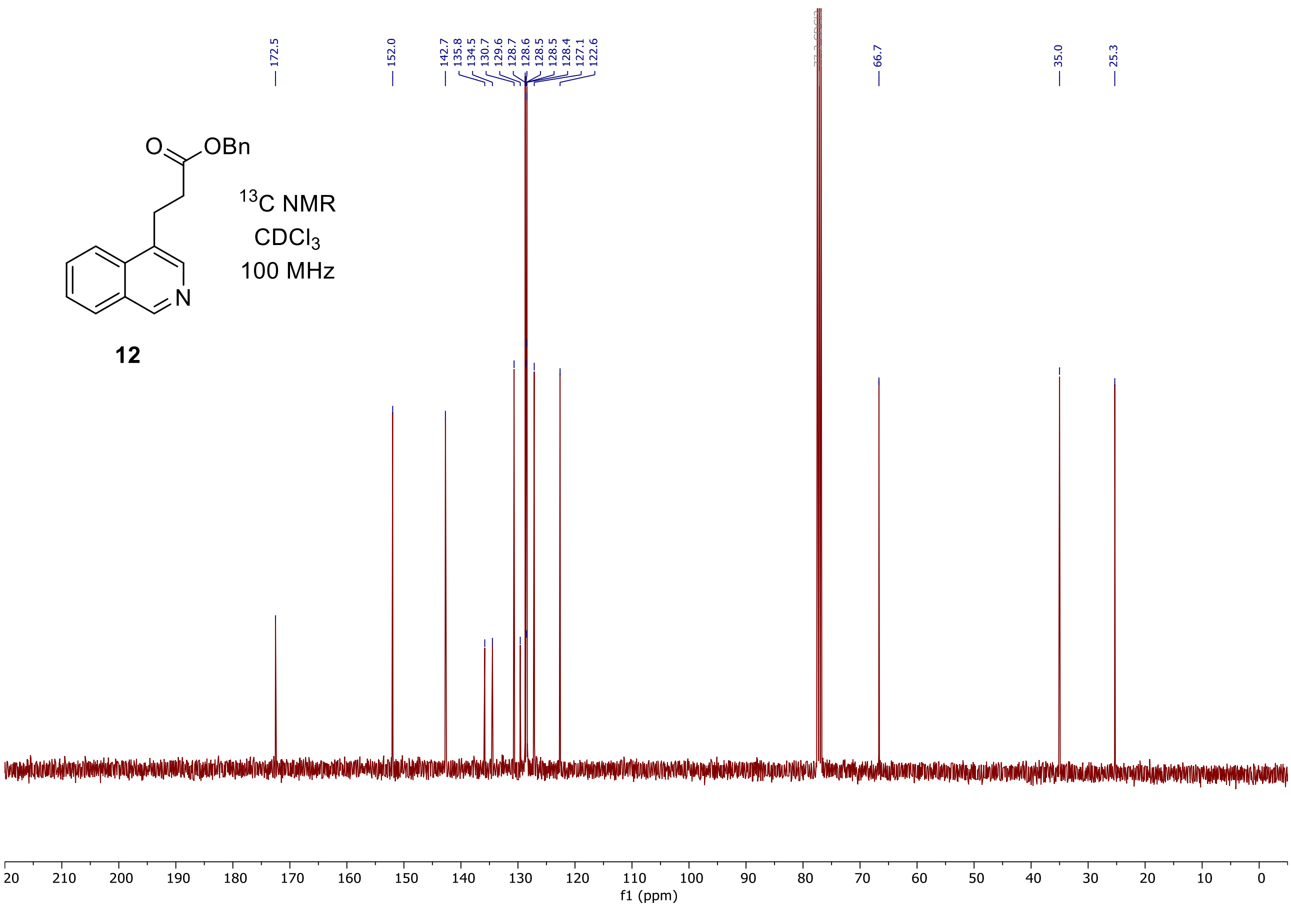

OBn
O
N
12
¹³C NMR
CDCl3
100 MHz
172.5
152.0
142.7
135.8
134.5
130.7
129.6
128.7
128.6
128.5
128.5
128.4
127.1
122.6
66.7
35.0
25.3
f1 (ppm)

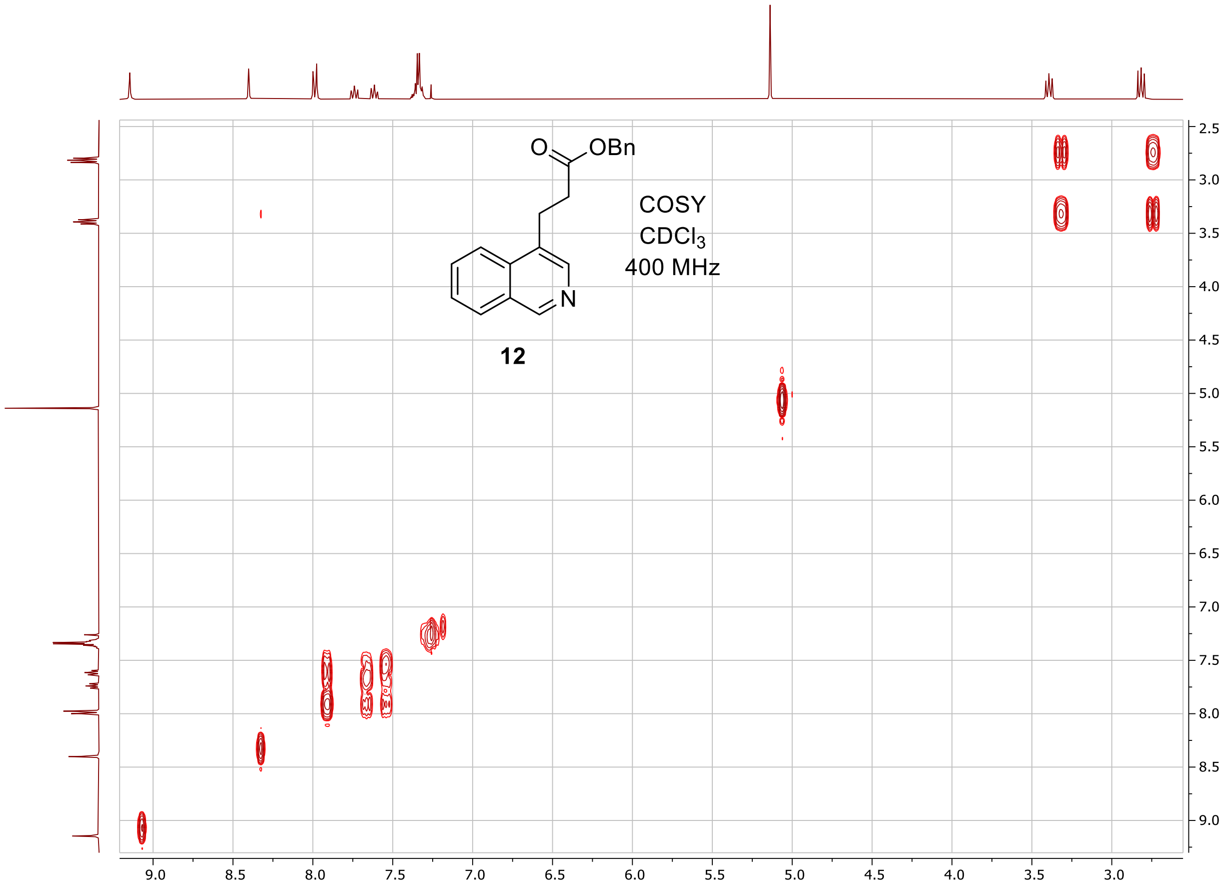
O
OBn
N
12
COSY
CDCl3
400 MHz

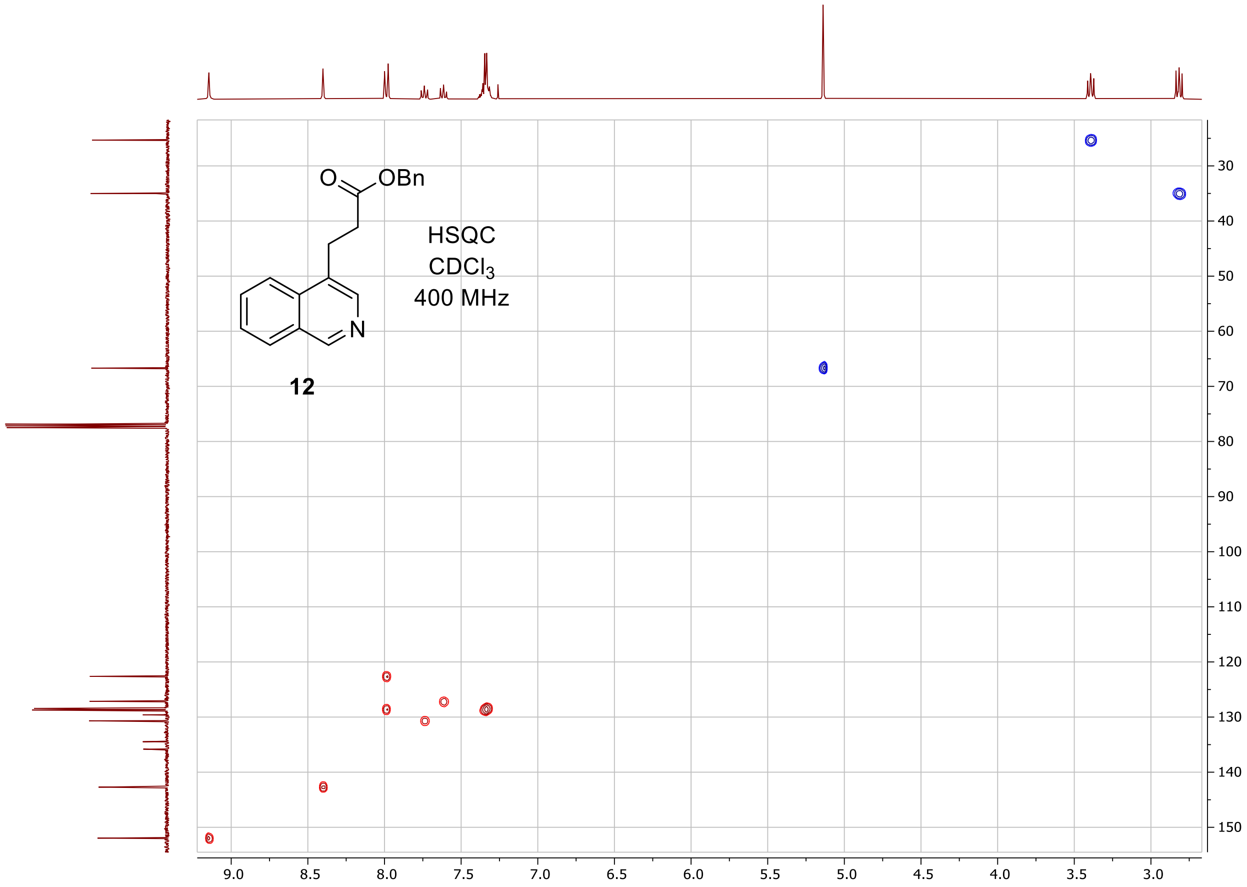
O
OBn
N
12
HSQC
CDCl3
400 MHz

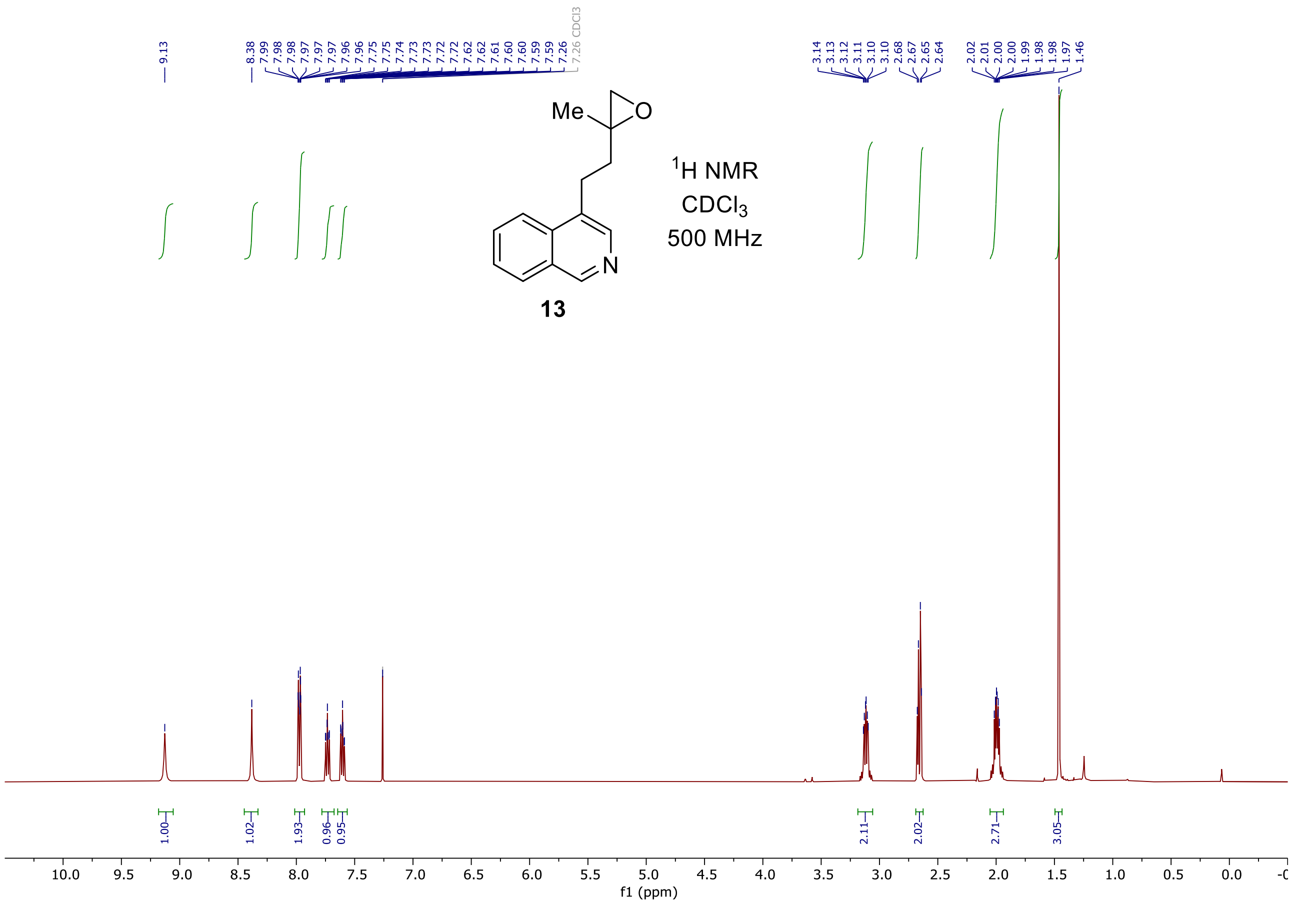

9.13
8.38
7.99
7.98
7.98
7.97
7.97
7.97
7.96
7.96
7.75
7.75
7.74
7.73
7.73
7.72
7.72
7.62
7.62
7.61
7.60
7.60
7.59
7.59
7.26 CDCl3
3.14
3.13
3.12
3.11
3.10
3.10
2.68
2.67
2.65
2.64
2.02
2.01
2.00
2.00
1.99
1.98
1.98
1.97
1.46
Me
O
N
13
1H NMR
CDCl3
500 MHz
1.00
1.02
1.93
0.96
0.95
2.11
2.02
2.71
3.05
f1 (ppm)

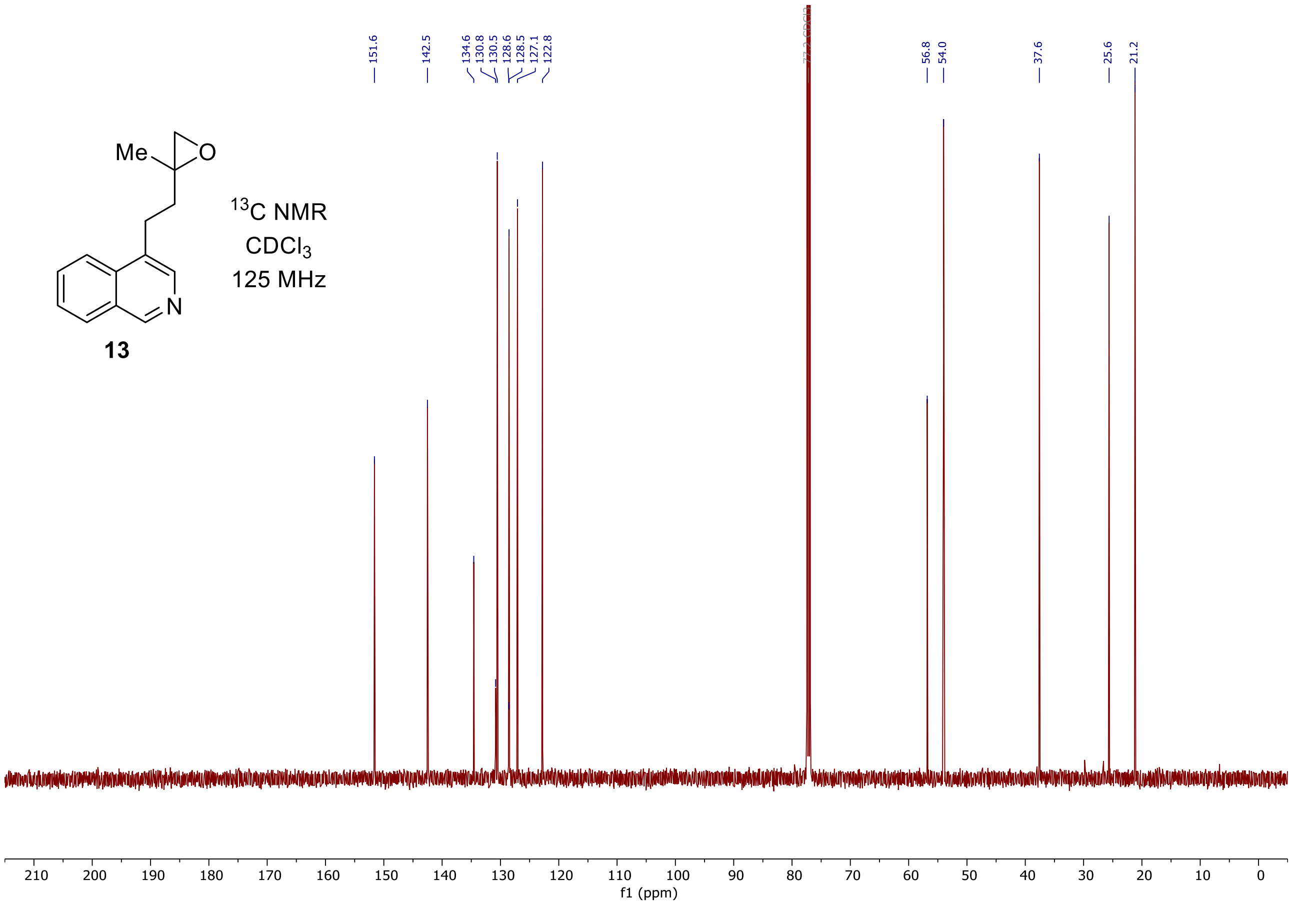

151.6
142.5
134.6
130.8
130.5
128.6
128.5
127.1
122.8
56.8
54.0
37.6
25.6
21.2
Me
O
N
13
13C NMR
CDCl3
125 MHz
f1 (ppm)

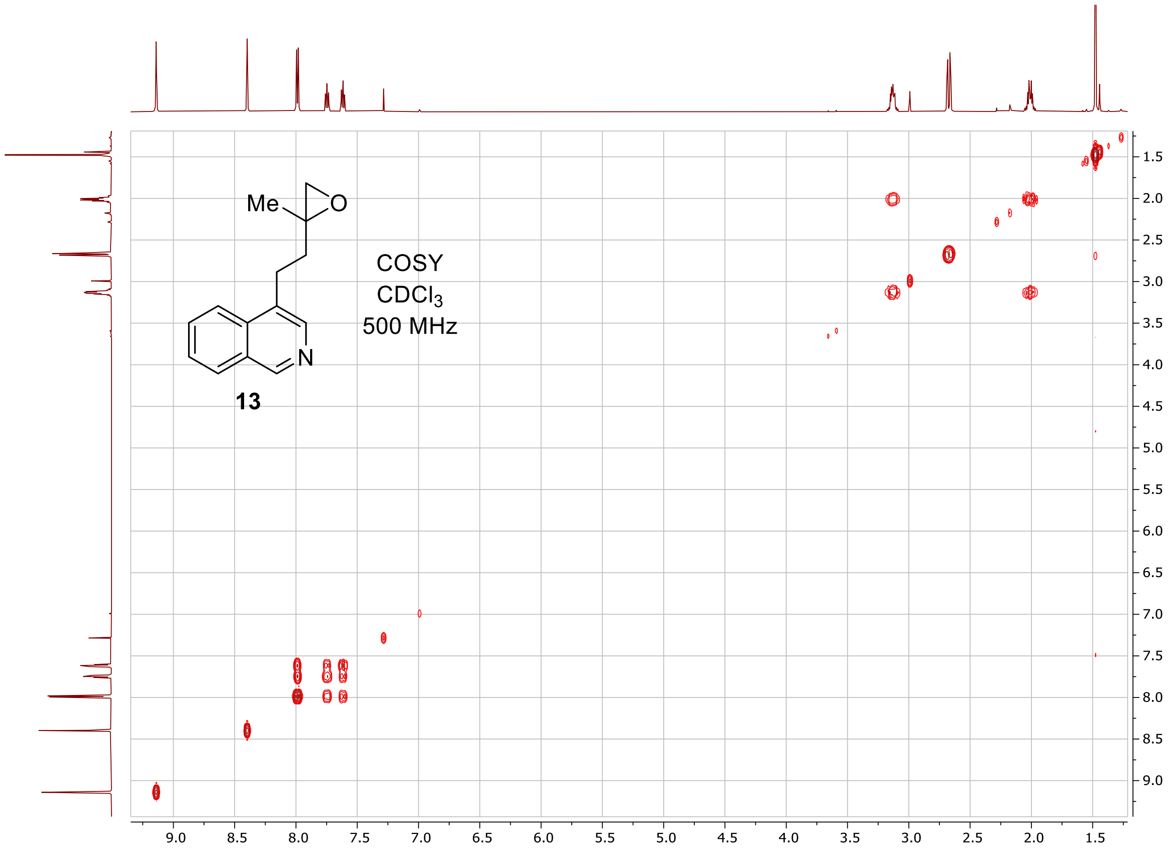

Me
O
N
13
COSY
CDCl$_3$
500 MHz

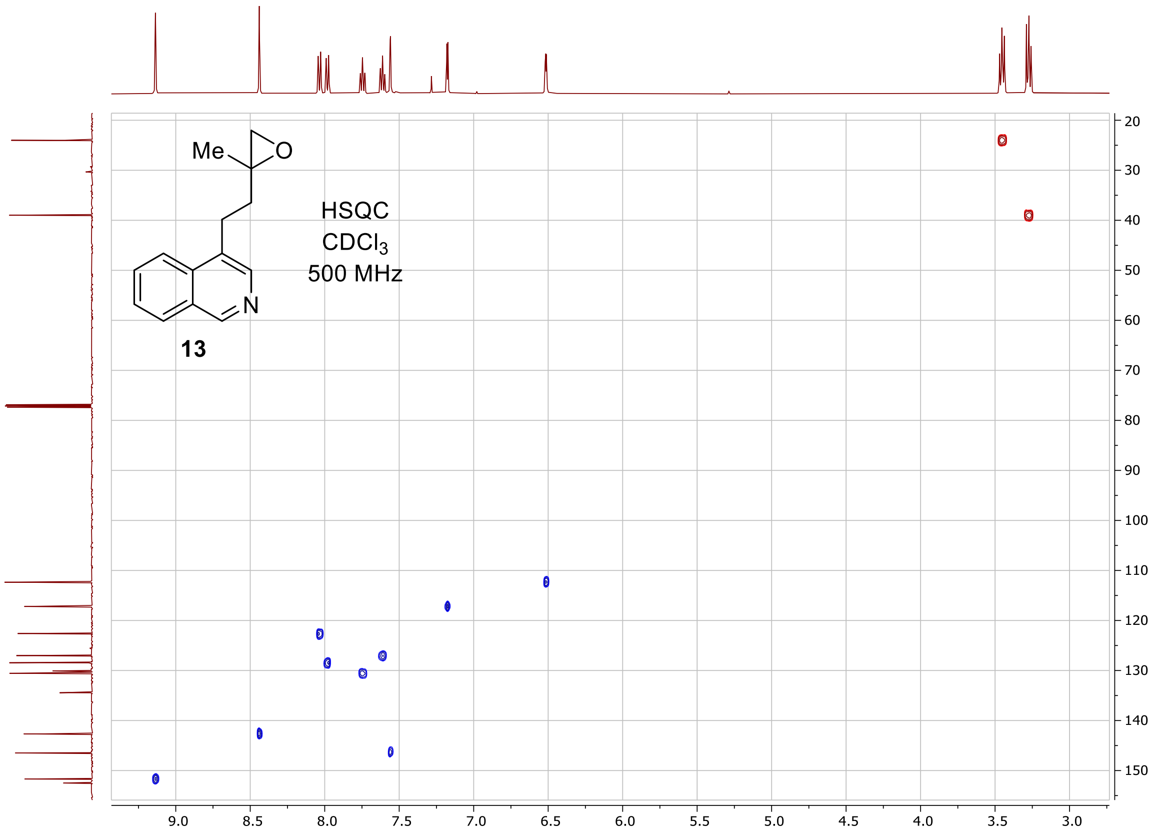

Me
O
N
13
HSQC
CDCl$_3$
500 MHz

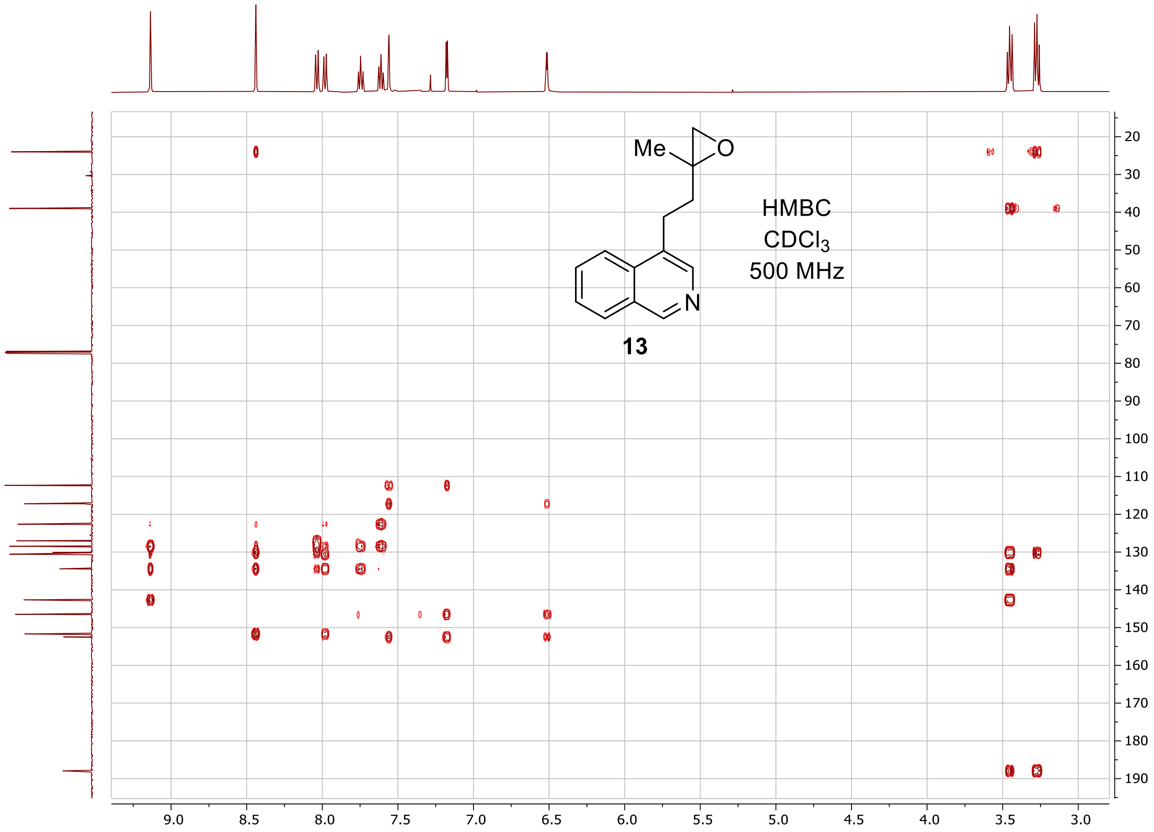

Me
O
N
13
HMBC
$CDCl_3$
500 MHz

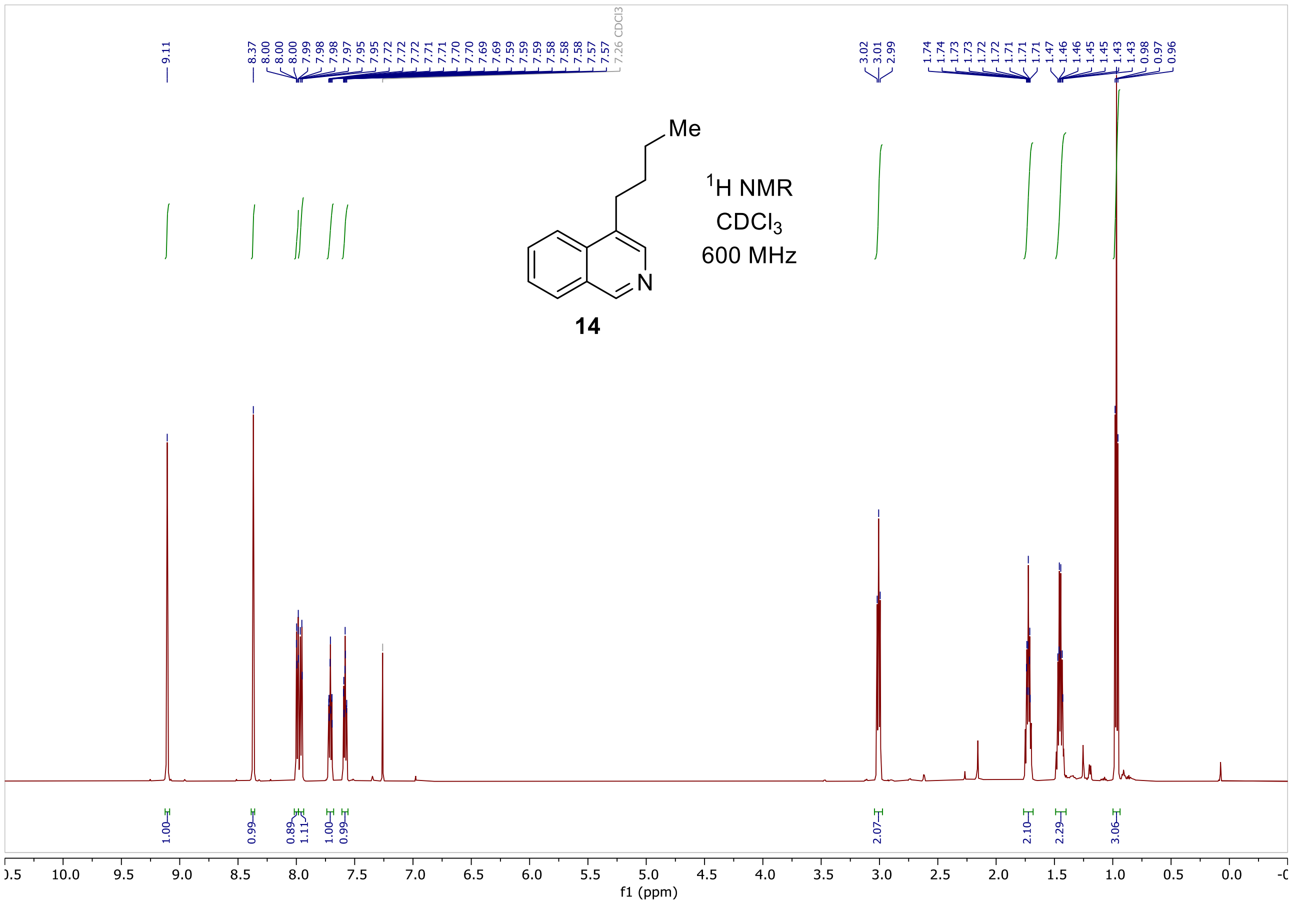
Me
N
14
1H NMR
CDCl3
600 MHz
9.11
8.37
8.00
8.00
7.99
7.98
7.98
7.97
7.95
7.95
7.72
7.72
7.72
7.71
7.71
7.70
7.69
7.69
7.59
7.59
7.59
7.58
7.58
7.57
7.57
7.26 CDCl3
3.02
3.01
2.99
1.74
1.74
1.73
1.73
1.72
1.72
1.71
1.71
1.71
1.47
1.46
1.46
1.45
1.45
1.43
1.43
0.98
0.97
0.96
1.00
0.99
0.89
1.11
1.00
0.99
2.07
2.10
2.29
3.06
f1 (ppm)

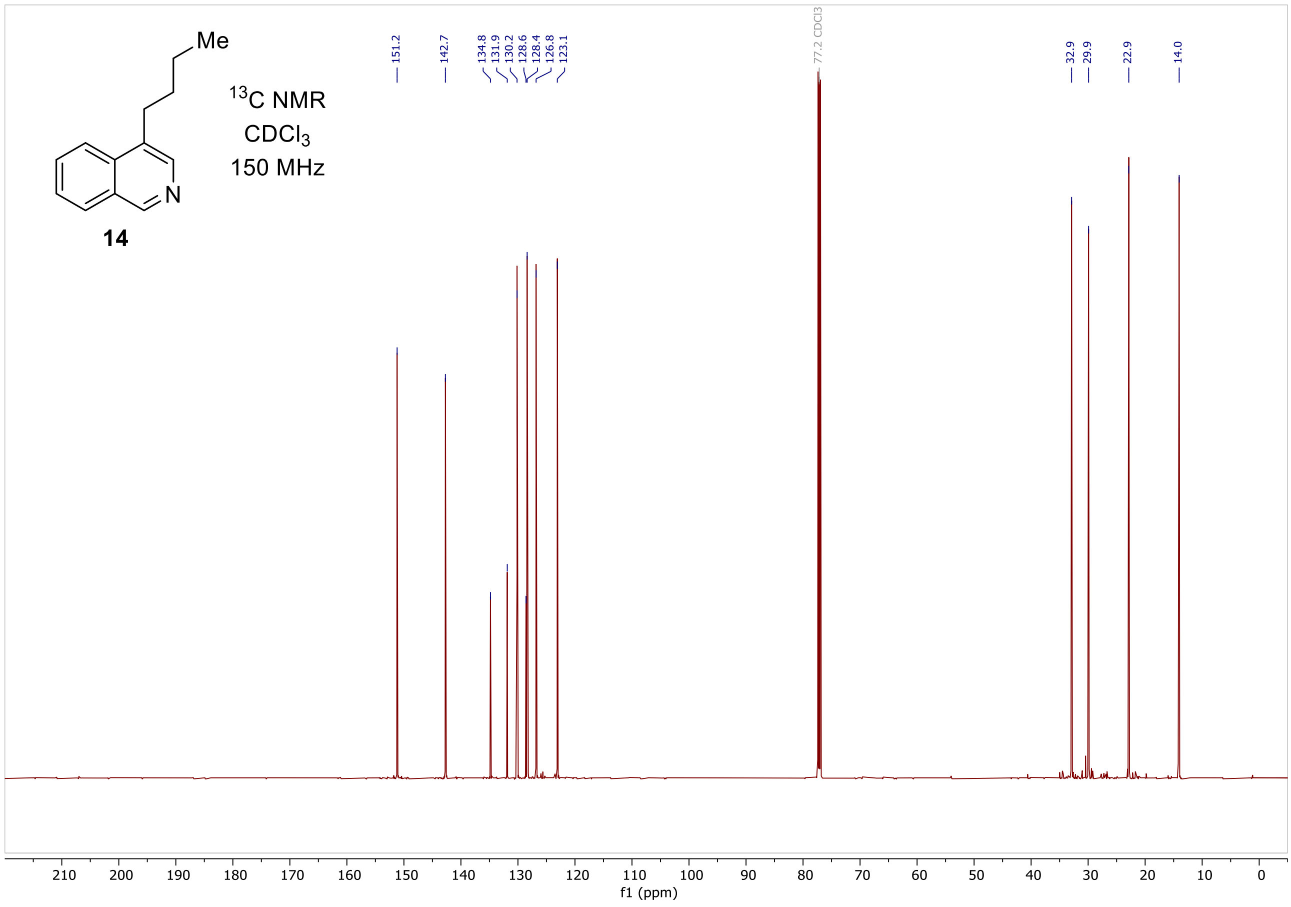
Me
N
14
13C NMR
CDCl3
150 MHz
151.2
142.7
134.8
131.9
130.2
128.6
128.4
126.8
123.1
77.2 CDCl3
32.9
29.9
22.9
14.0
f1 (ppm)

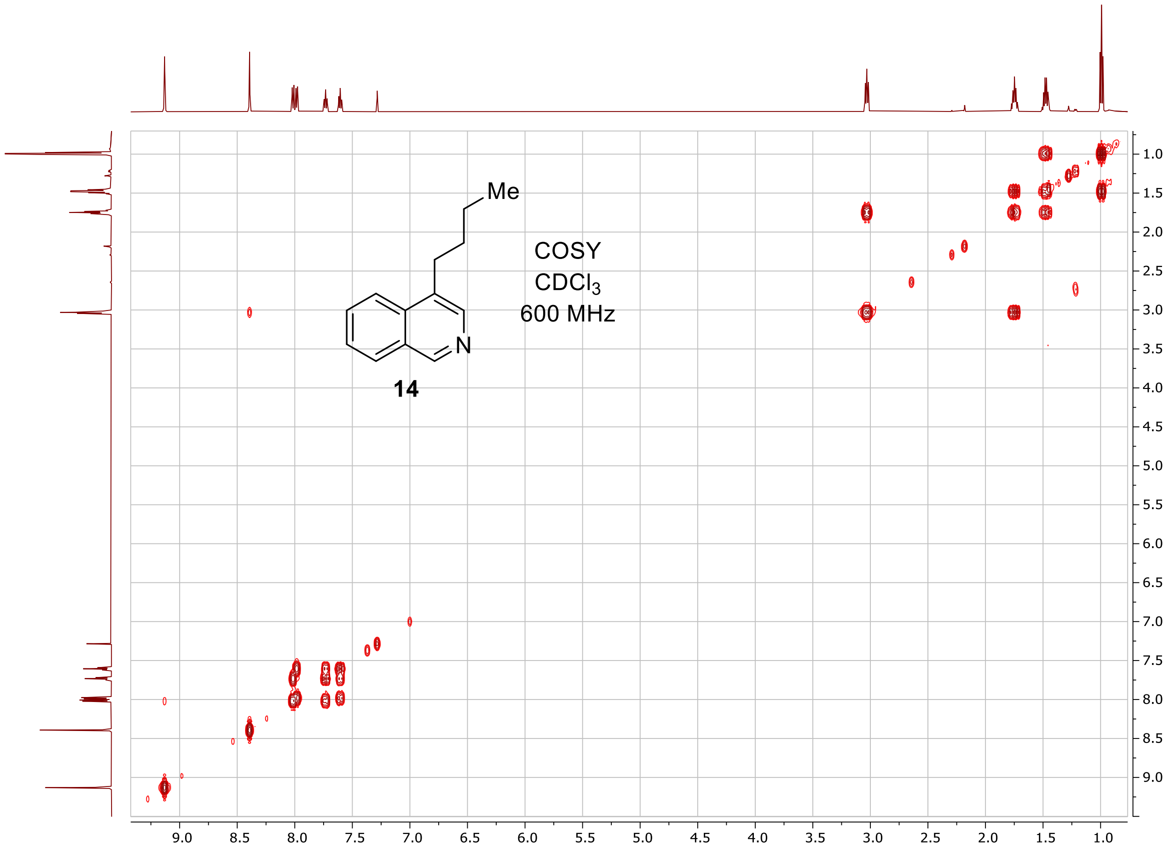
Me
COSY
CDCl3
600 MHz
N
14

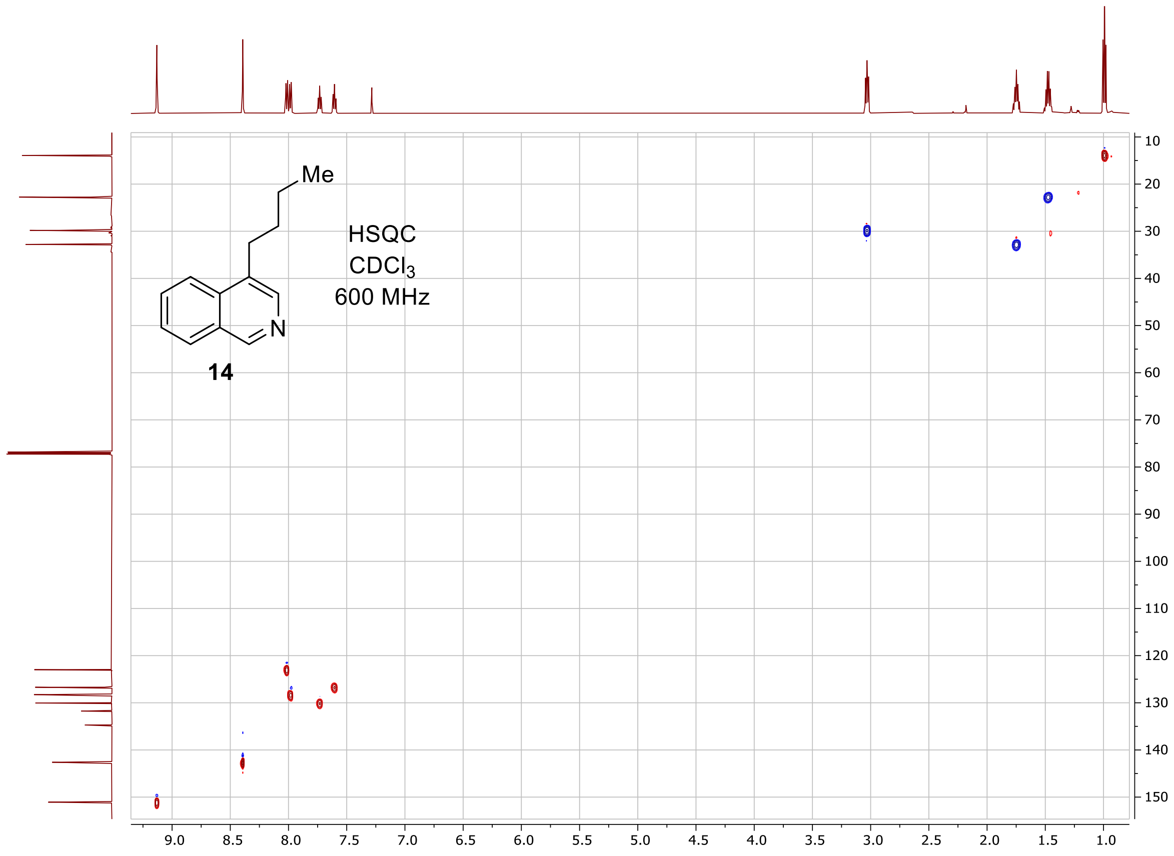
Me
HSQC
CDCl3
600 MHz
N
14

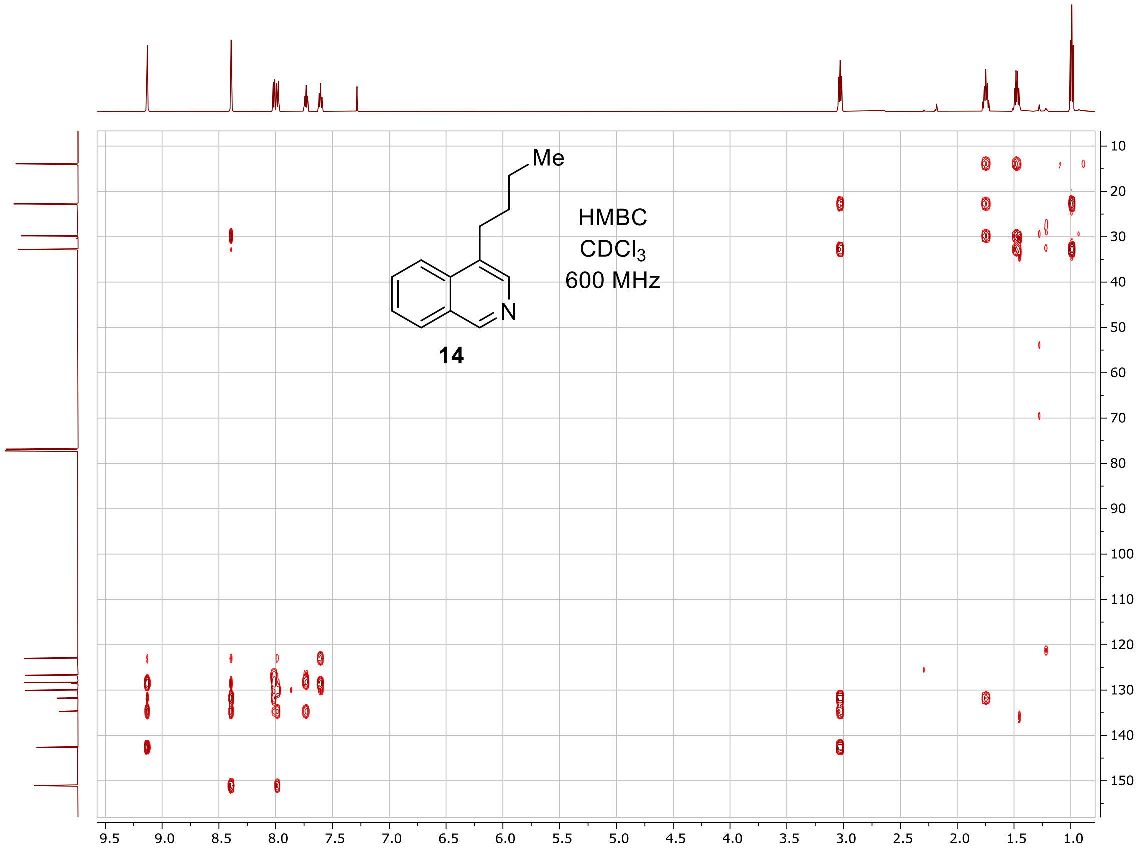
Me
N
14
HMBC
$CDCl_3$
600 MHz

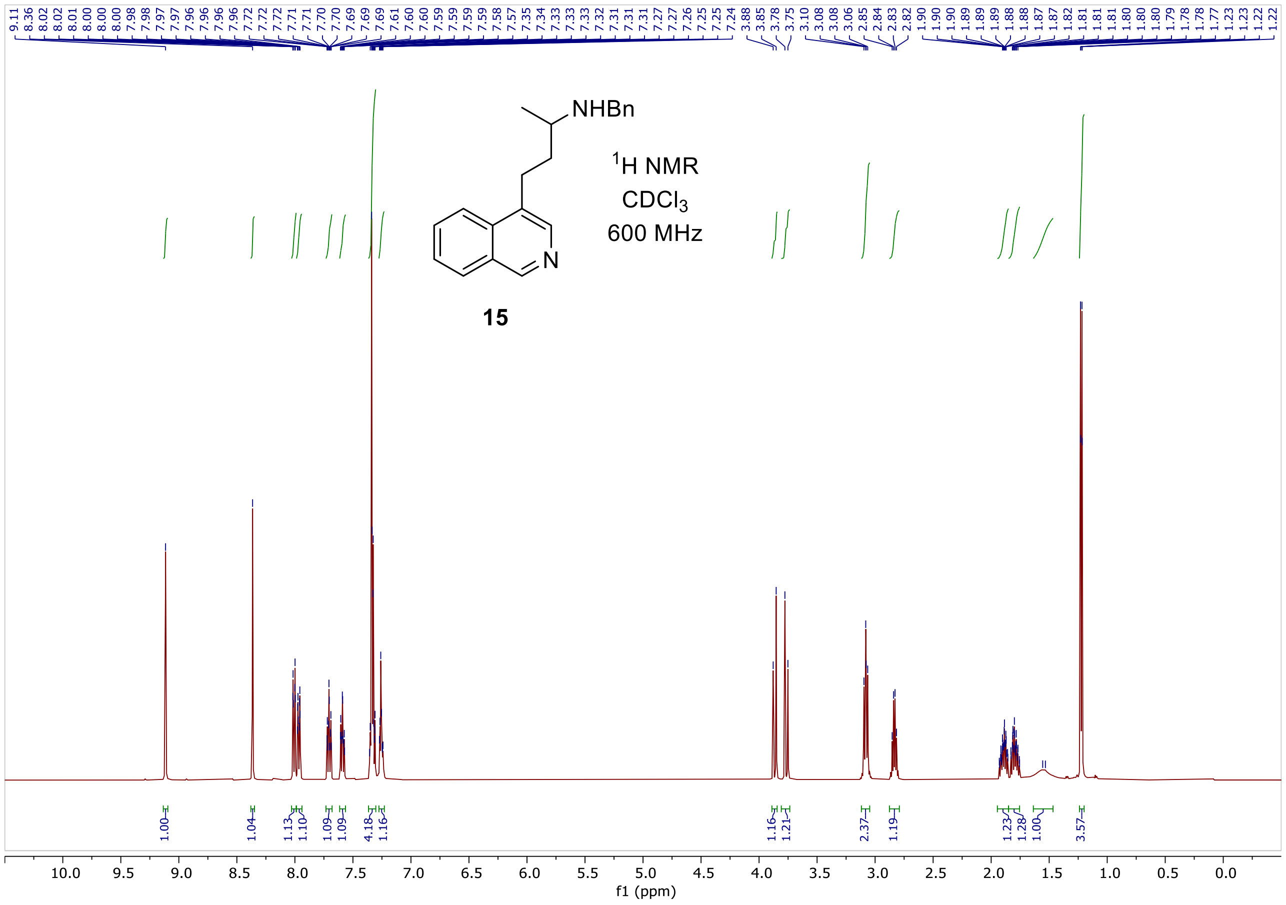
NHBn
N
15
^{1}H NMR
$CDCl_3$
600 MHz
f1 (ppm)

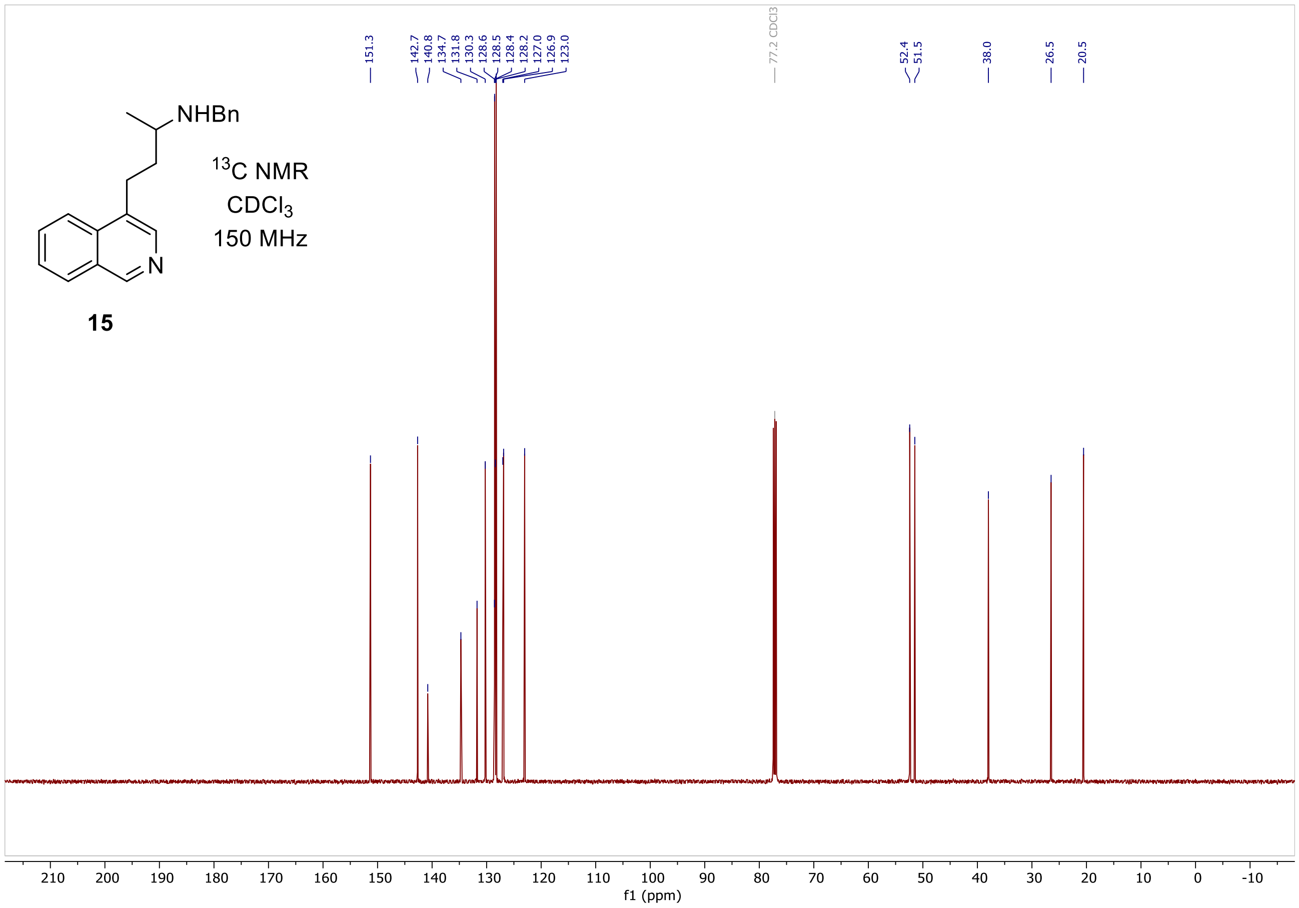
151.3
142.7
140.8
134.7
131.8
130.3
128.6
128.5
128.4
128.2
127.0
126.9
123.0
77.2 CDCl3
52.4
51.5
38.0
26.5
20.5
NHBn
N
15
^{13}C NMR
$CDCl_3$
150 MHz
f1 (ppm)

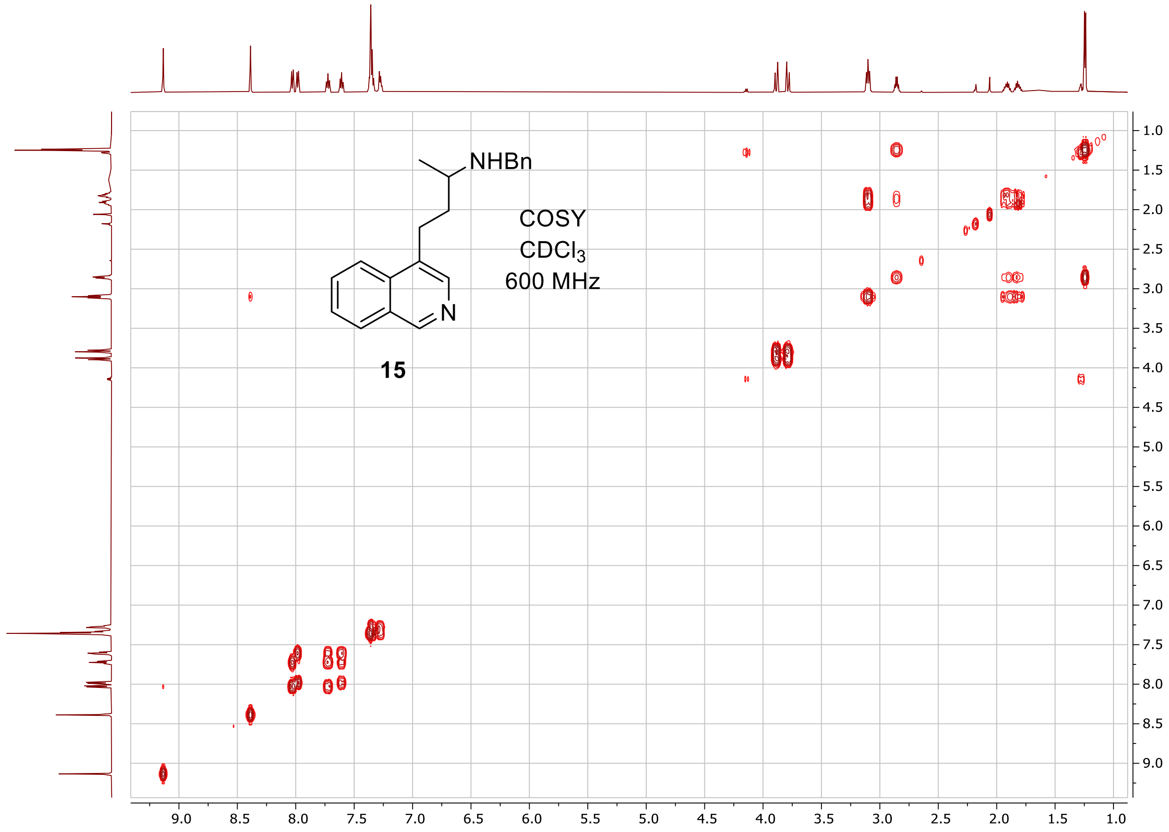
NHBn
COSY
CDCl3
600 MHz
15

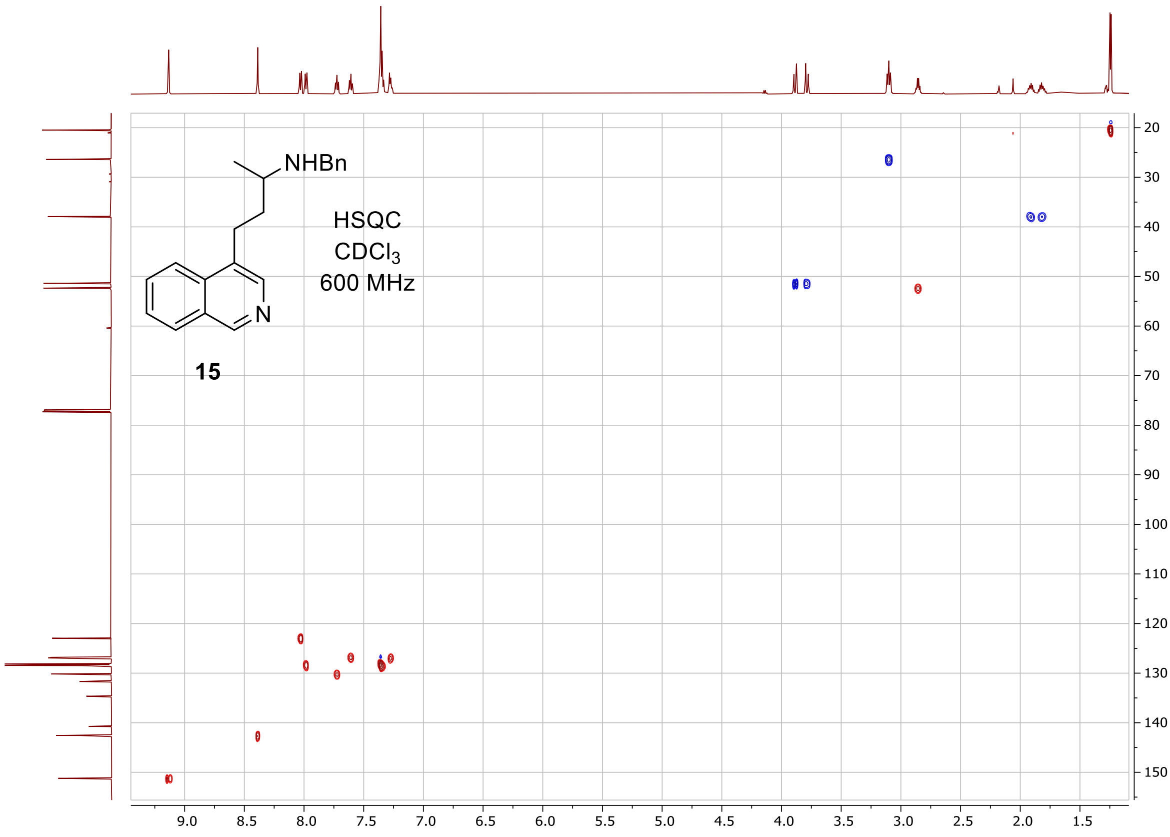
NHBn
HSQC
CDCl3
600 MHz
15

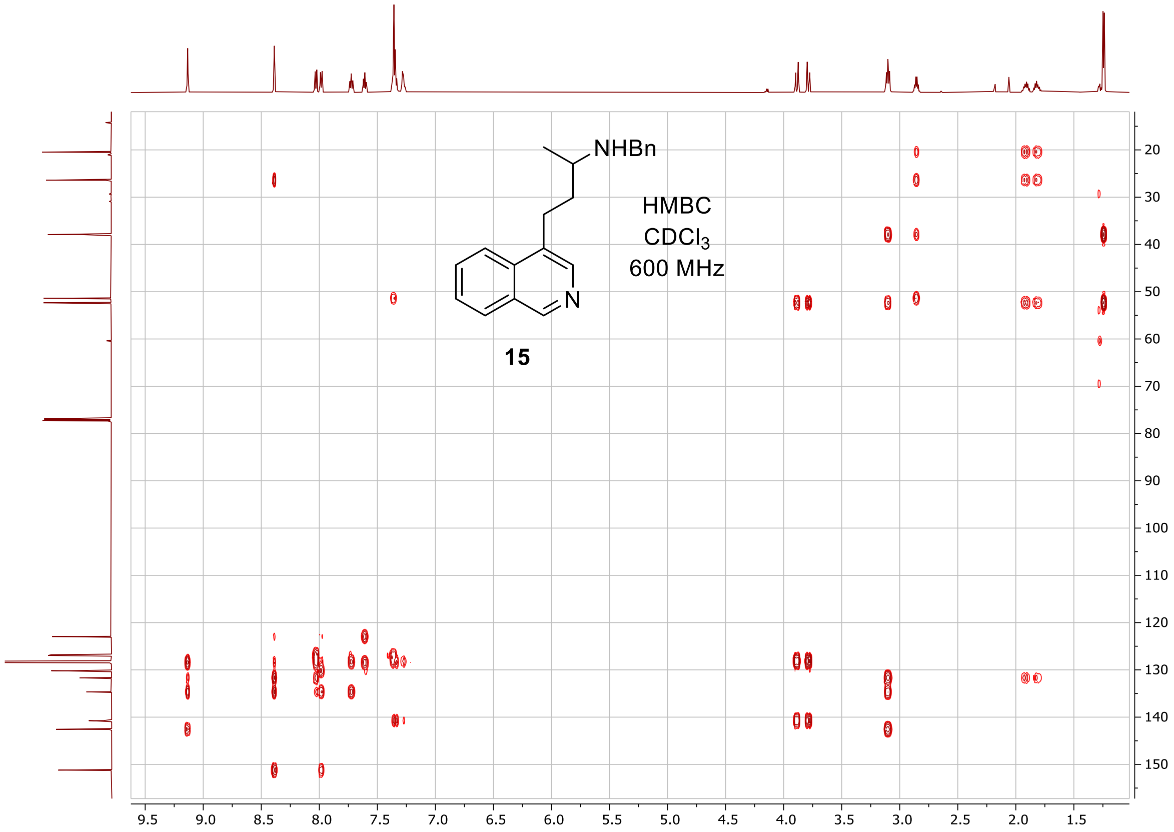
NHBn
N
15
HMBC
CDCl3
600 MHz
9.5
9.0
8.5
8.0
7.5
7.0
6.5
6.0
5.5
5.0
4.5
4.0
3.5
3.0
2.5
2.0
1.5
20
30
40
50
60
70
80
90
100
110
120
130
140
150

6. *References*

[1] A. B. Pangborn, M. A. Gairdello, R. H. Grubbs, R. K. Rosen, F. J. Timmers, *Organometallics*, **1996**, *15*, 1518.

[2] *Purification of Laboratory Chemicals*, 3rd edition. D.D. Perrin, W. L. F. Armarego, *Pergamon Press*, Oxford, **1988**.

[3] J. D. Firth, P. G. E. Craven, M. Lilburn, A. Pahl, S. P. Marsden, A. Nelson *Chem. Commun.* **2016**, *52*, 9837.

[4] Mainkar, P. S. Chippala, V. Chegondi, R. Chandrasekhar, S. *Synlett.* **2016**, *27* (13), 1969.

[5] Cooper, T.; Novak, A.; Humphreys, L. D.; Walker, M. D.; Woodward, S. *Adv. Synth. Catal.* **2006** *348*, 686.

[6] Jha, N.; Singh, R. P.; Saxena, P.; Kapur, M. *Org. Lett.* **2021**, *23*, 8694.

[7] Gupta, R. B.; Franck, R. W.; Onan, K. D.; Soll, C. E. *J. Org. Chem.* **1989**, *54*, 1097.

[8] Choudhury, A. R.; Mukherjee, S. *Chem. Sci.* **2016**, *7*, 6940.

[9] Jin, Y.; Makida, Y.; Uchida, T.; Kuwano, R. *J. Org. Chem.* **2018**, *83*, 3829.

[10] Rickerby, J.; Vallet, M.; Bernardinelli, G.; Viton, F.; Kundig, E. P. *Chem. Eur. J.* **2007**, *13*, 3354.

[11] Bera, N.; Samanta, S.; Sarkar, D. *J. Org. Chem.* **2021**, *86*, 16395.

[12] Zhang, N. Zhng, C. Hu, X. Xie, X. Liu, Y. *Org. Lett.* **2021**, *23* (15), 6004-6009.

[13] Sauza, A. Morales-Serna, J. A. García-Molina, M. Gavino, R. Cárdenas, *Synthesis* **2012**, *2*, 272.

[14] Cárdenas, J. Gavino, R. García-Ríos, E. Rios-Ruiz, L. Puello-Cruz, A. C. Morales-Serna, F. N. Gómez, S. López-Torres, A. Morales-Serna, J. A. *RSC Adv.* **2021**, *11*, 20278.

[15] Chen, D. Xu, G. Zhou, Q. Chung, L. W. Tang, W. *J. Am. Chem. Soc.* **2017**, *139* (29), 9767.
